# Exploring the temporal statistical relationship between commercial cerebral near-infrared spectroscopy signals and intracranial pressure in traumatic brain injury

**DOI:** 10.1186/s40635-026-00964-8

**Published:** 2026-08-27

**Authors:** Noah Silvaggio, Kevin Y. Stein, Makenna Coldwell, Vuk Roca, Amanjyot Singh Sainbhi, Isuru Herath, Tobias Bergmann, Rakibul Hasan, Mansoor Hayat, Jaewoong Moon, Frederick A. Zeiler

**Affiliations:** 1 Department of Human Anatomy and Cell Science, Rady Faculty of Health Sciences, University of Manitoba, Winnipeg, Canada; 2 Graduate Program in Biomedical Engineering, Price Faculty of Engineering, University of Manitoba, Winnipeg, Canada; 3 Department of Biosystems Engineering, Price Faculty of Engineering, University of Manitoba, Winnipeg, Canada; 4 Undergraduate Engineering, Price Faculty of Engineering, University of Manitoba, Winnipeg, MB Canada; 5 Section of Neurosurgery, Department of Surgery, Rady Faculty of Health Sciences, University of Manitoba, Winnipeg, Canada; 6 Department of Clinical Neuroscience, Karolinska Institutet, Stockholm, Sweden; 7 Pan Am Clinic Foundation, Winnipeg, MB Canada

**Keywords:** Intracranial pressure, ICP, Cerebral near-infrared spectroscopy, NIRS, Regional oxygen saturation, RSO_2_

## Abstract

**Background:**

Monitoring of intracranial pressure (ICP) is important for not only assessing neurological status of patients following traumatic brain injury (TBI), but also for guiding therapeutic interventions for the prevention of secondary neurological injury. Although current ICP monitoring techniques are highly temporally accurate, they come with some significant limitations. These limitations include the invasive nature, expertise required for placement of these devices, high cost associated with maintenance, and poor spatial resolution. Cerebral near-infrared spectroscopy (NIRS) offers a non-invasive alternative technique for cerebral physiological monitoring. This modality eliminates the need for surgical expertise, as well as decreasing the financial cost associated with monitoring cerebral physiology. Preliminary evidence has pointed to a potential link between the NIRS variable, regional oxygen saturation (rSO_2_), and invasively obtained ICP. Despite this, there are very few studies that exist that investigate the direct statistical relationship between NIRS variables, such as rSO_2_, and invasively obtained ICP. As such, this study set out to characterize and compare the temporal statistical properties of commercial NIRS-based rSO_2_, ICP, and mean arterial pressure (MAP) signals.

**Results:**

Utilizing retrospective high-frequency physiologic data from a population of 118 TBI patients across different temporal resolutions and data window sizes, we evaluated independent statistical signal structure of rSO_2_ and ICP using variance analysis and ARIMA modelling. Following normalization, variance metrics remained significantly different between all physiologic variable comparison groups, across all temporal resolutions and window sizes examined. In contrast, median optimal ARIMA model orders converged, with no significant differences observed between any of the included physiological variables. Next, Pearson correlation, cross-correlation, Granger causality, and VARIMA IRF analysis continued to show increasing temporal similarity between ICP and rSO_2_ as temporal resolution decreased and window size increased. In contrast, throughout the majority of the analyses, the relationship between AMP and rSO_2_ remained not only consistently weaker, but also more inconsistent across the majority of the analyses.

**Conclusion:**

Findings from this study demonstrate convergence in the temporal statistical behaviour of ICP and rSO_2_ at lower temporal resolutions, providing a foundation for future predictive non-invasive ICP monitoring approaches. Further investigation into direct statistical relationships and development of reliable non-invasive ICP prediction models using NIRS-derived data remains necessary for clinical utility.

**Supplementary Information:**

The online version contains supplementary material available at https://doi.org/10.1186/s40635-026-00964-8.

## Introduction

Live-time continuous monitoring of cerebral physiologic processes often requires measurement using invasive modalities [1]. In particular, parenchymal, or cerebrospinal fluid-based devices must be surgically implanted to measure intracranial pressure (ICP). ICP is the pressure within the cranial vault, determined by factors including the cerebral tissue, surrounding blood, and cerebrospinal fluid [2]. In certain cases mass lesions, such as hematomas or tumors can further increase ICP through intracranial volume expansion [2]. In a clinical setting, continuous monitoring of ICP is crucial particularly when involving patients who have suffered traumatic brain injury (TBI). After TBI, the risk for secondary neurological injury such as hemorrhage or edema is much higher, signifying the importance of monitoring ICP in the intensive care unit (ICU) [3]. In both of these cases, increases in ICP can occur, leading to intracranial hypertension (IH) [4]. If left untreated, IH can result in an array of different symptoms, including but not limited to headaches, vison impairments, nausea, seizures, coma, and can contribute to long-term morbidity and even mortality [4].

Invasive ICP monitors, whether they are external ventricular drains (EVDs) or parenchymal pressure transducers, come with some significant limitations [5]. EVDs are typically placed in the ventricular system and are shown to have a high degree of accuracy; however, they do come with the risk of both hemorrhage and infection [5]. Parenchymal pressure transducers such as epidural, subdural, and parenchymal devices can be easier to implant into their desired locations, particularly in cases of severe brain swelling where locating the ventricles may be too cumbersome [5]. A limitation of these parenchymal pressure transducers is the fact that once placed into the cerebral tissue, these devices cannot be re-calibrated which could lead to inaccuracies and issues with drift [5]. Despite their high accuracy, these invasive ICP monitors have several other important limitations that should be addressed, including the risk of neurological injury during placement, specialized expertise and surgical skills required, high financial costs for maintenance, and have limited spatial resolution [6, 7].

Cerebral near-infrared spectroscopy (NIRS) technology is a tool that was first described in 1977 by Franz Jöbsis as a way to non-invasively monitor cerebral oxygenation [8]. With the ability to measure the absorption of near-infrared (NIR) light by oxygenated hemoglobin (O_2_Hb) and deoxygenated hemoglobin (HHb), NIRS systems can estimate regional cerebral oxygen (rSO_2_) from the ratio of O_2_Hb to tHb [9–11]. Due to the characteristics of NIR light, as well as the NIRS system itself, NIRS technology has been proposed as a method to continuously and non-invasively monitor cerebral physiology. As NIRS systems use optodes that stick directly onto an individual’s scalp, typically bifrontally on the forehead, the need for surgical expertise is eliminated [12]. Furthermore, NIRS technology is also more cost-effective when compared to other forms of cerebral monitors, in particular invasive methods. The combination of a more cost-effective and non-invasive method to monitoring cerebral physiology increases the spatial resolution as multiple cerebral locations can be easily monitored.

Previous studies have suggested a potential relationship between ICP and NIRS-derived variables because both may reflect aspects of pulsatile CBV [13, 14]. However, the limited number of direct comparisons between invasively measured ICP and commercial NIRS-derived rSO_2_ have generally demonstrated weak or inconsistent relationships [13, 14]. A recent systemically conducted scoping review set out to identify all of the current literature that explored a direct statistical relationship between invasively obtained ICP and NIRS-based signals [15]. Through this systemic scoping review, it became clear that few studies exist that document an objective comparison between ICP and NIRS-based signals [15]. Among the studies that were identified, results do point to a potential positive correlation amongst the signals sources; However, further research into exploring the temporal dynamics of this relationship is required [15]. Previous studies have not examined whether this relationship changes across different temporal resolutions and data window sizes, despite both of these physiological variables being continuous in nature. Characterizing these temporal statistical properties is an important step in determining whether commercial rSO_2_ contains information that can contribute to non-invasive ICP estimation in the future. Therefore, this study set out to characterize and compare the independent temporal statistical properties of commercial NIRS-based rSO_2_ signals and ICP, as well as exploring the temporal relationship between mean arterial pressure (MAP), rSO_2_, and ICP across multiple temporal resolutions and data window sizes.

## Materials and methods

### Study design and human populations

**Fig. 1:**
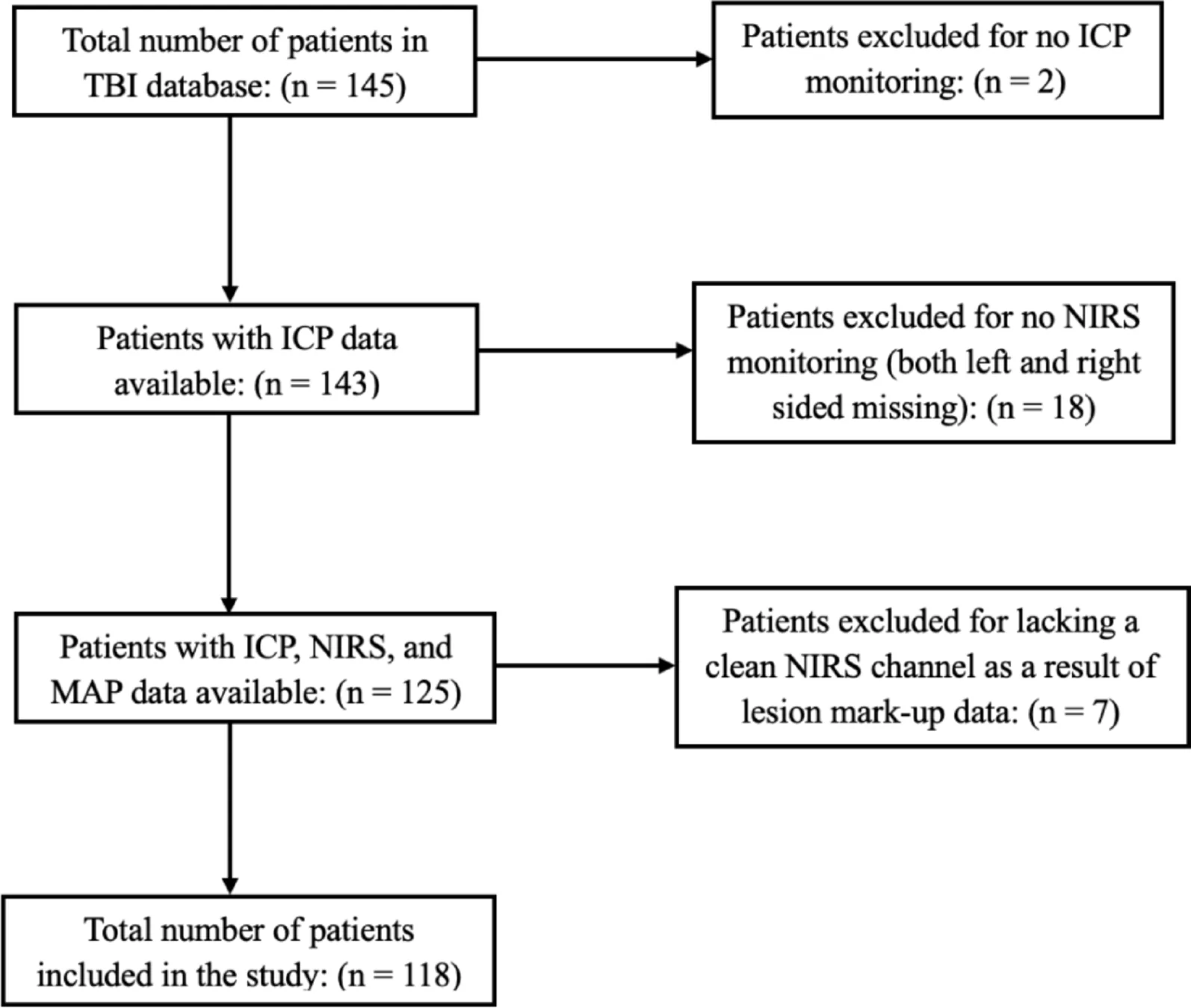
A flowchart depicting the total number of patients included and screened in this study, and the reasons for exclusion of some patients. Note that when discussing a clean NIRS channel, patients were included if they had either a left or right sided channel not accompanied with either a frontal contusion or scalp hematoma on the same side

High frequency cerebral physiologic data collected between early 2019 and June 2025 from patients who had suffered acute moderate/severe TBI was retrospectively accessed for this study. This data originated from an on-going prospectively maintained TBI database consisting of existing de-identified archived data collected from adult moderate/severe TBI patients admitted to Winnipeg Health Sciences Centre (HSC) Surgical Intensive Care Unit (SICU; Shared Health Manitoba) who required invasive ICP monitoring for clinical purposes based on Brain Trauma Foundation guidelines [16]. Details related to this database can also be seen in other recent studies [17–19]. Patients within this cohort have also received an array of other invasive and non-invasive cerebral physiologic monitoring devices depending on the unique clinical situations and the decisions of the treating team at the time of admission to the ICU. More specifically, NIRS monitoring was performed when clinically available and at the discretion of the treating clinical team. Only those TBI patients were included who underwent invasive ICP monitoring and had concurrent MAP and NIRS monitoring available. For this retrospective study, the patient population consisted of 118 individuals with MAP, ICP and rSO_2_ monitoring (Fig. 1).

### Ethical considerations

This is a retrospective cohort study of an existing prospectively maintained TBI database by our lab group. Ethical approval for this analysis has been secured and is covered under the University of Manitoba Heath Research Ethics Board and Shared Health Research Impact Committee - H2024:266 (SH2024-140) and H2024:217 (SH2024-114).

### Data collection

In this study, four high-resolution physiologic data streams were used including MAP, ICP, and rSO_2_ (both left and right frontal lobes). Invasive ICP monitoring was completed using Codman ICP Express or Cerelink from Integra Life Sciences (Princeton, NJ). Additionally, as a part of the standard monitoring within the SICU, the arterial blood pressure (ABP) of patients was monitored for the duration of their ICU stay. Monitoring of ABP was done invasively through arterial lines (Baxter Healthcare Corp. CardioVascular Group, Irvine, CA). In order to obtain values for rSO_2_, bifrontal NIRS monitoring was measured continuously over the course of the ICU stay (INVOS 5100 C or 7100, Medtronic, Minneapolis, MN). Sensors were placed on both the left and the right side of the forehead to monitor rSO_2_ data from the right and left frontal lobes when possible, at a sampling rate of 1 Hz. When both left- and right-sided NIRS channels were available, channels were analyzed separately, and as their own reading for rSO_2_.

All of the physiological data was recorded using Intensive Care Monitoring “Plus” (ICM +) software (Cambridge Enterprise Ltd, Cambridge, UK, http://www.neurosurg.cam.ac.uk/icmplus) which was connected to the SICU monitors. Signals were digitized using analog to digital signal converters (DT9803 or DT9826; Data Translation, Marlboro, MA). Physiologic signals were sampled at a frequency of 100 Hz (Hz) in order to capture full waveforms, where applicable.

Archived lesion mark-up data was reviewed in order to identify any hematomas, brain contusions, and scalp concerns that may impact NIRS channel recordings from the bifrontal sensors. Entire channels were excluded when ipsilateral lesions or scalp conditions including either frontal contusions or scalp hematomas were present, as these could compromise signal quality from that sensor (i.e. no data were used from such channels). When only one channel was deemed non-viable, the contralateral channel was still retained for analysis and patients were included in the study. In total, 7 patients were excluded as a result of the lesion-mark up data, as these patients did not have a clean signal from either the left or right sided sensor (Fig. 1).

Additionally, demographic data which was utilized for subgroup analysis was also included in the TBI database. This included ICP monitor location, age, biologic sex, decompressive craniotomy (DC) timing (if present), Marshal computed tomography (CT) score, admission Glasgow Coma Scale (GCS), admission pupil exam, and Rotterdam CT score.

### Physiologic data cleaning and processing

Prior to any further processing or analysis, signal artifacts from the archived data were removed manually by qualified personnel using ICM+ software who were blinded from the objectives of this present study. During this process, artifacts were identified through instances in which the magnitude of the signal in question is physiologically implausible (outside of the range of 0–100 mmHg for ICP, and 0–300 mmHg for arterial blood pressure and where there has been a clear loss of waveform within the signals [20, 21]. For example, a clear loss of waveform can be as a result of sensor disconnect, and hence an artifactual segment. In order to determine the pulse amplitude of ICP (AMP), the fundamental Fourier amplitude of the ICP pulse waveform was calculated over a 10-second window, and updated every 10 s [22, 23]. MAP was utilized throughout the subsequent analysis, which was obtained by averaging the patients ABP signals for the duration of a cardiac cycle. For all of the recorded signals: ICP, AMP, ABP, and rSO_2_ (both left and right sides), 10-second moving averages were exported from ICM + in order to focus on the slow-wave oscillatory behaviour of the parent signals, reducing the influence of higher-frequency cardiac and respiratory oscillations and isolating the frequency to the range associated with cerebral autoregulation (CA) [24, 25]. This processing also allowed for the comparison between signals acquired at different sampling frequencies, as commercial NIRS-derived rSO_2_ is sampled at 1 Hz, whereas ICP is sampled at 100 Hz. Further processing of the included data streams was done to remove values that were likely to be artifact. MAP values lower than 30 mmHg and greater than 200 mmHg, ICP values greater than 100 mmHg, AMP values greater than 5 mmHg, and rSO_2_ values less than 10% were all removed prior to any analysis [26–30]. These values were chosen, as a true physiologic value outside of this range is unlikely.

### Down sampling of included data streams

Following preprocessing within ICM+, physiologic signals were exported as 1-second and 10-second time-series. The 10-second decimated data for these continuous physiologic signals was then further down-sampled using non-overlapping moving average filters in order to generate additional lower temporal resolution datasets (aside from the 10-second frequency; 1-minute and 10-minute intervals). This down sampling occurred independently for each signal. These temporal resolutions were selected as they represent commonly encountered data export rates from commercially available software, or commonly recorded data frequencies in bedside patient charts. Figure 1 highlights the general impact of down sampling on data structure. Furthermore, window sizes were created through the utilization of non-overlapping intervals, such that each window represents a distinct segment of the desired signal. In the case where a patient may have had a monitoring period shorter than the current analysis window being examined, data was not included in the analysis in order to ensure the appropriate number of data points were included in each time period being analysed.

In this study, both temporal resolutions and window sizes were utilized. Temporal resolution refers to the sampling interval of the time-series, such that a value for the physiological variable is available every 1 s, 10 s, 1 min, or 10 min. Window size refers to the duration of each non-overlapping segment for the calculation of the statistic, including 1-minute, 5-minute, 15-minute, 30-minute, and 1-hour windows.

**Fig. 2:**
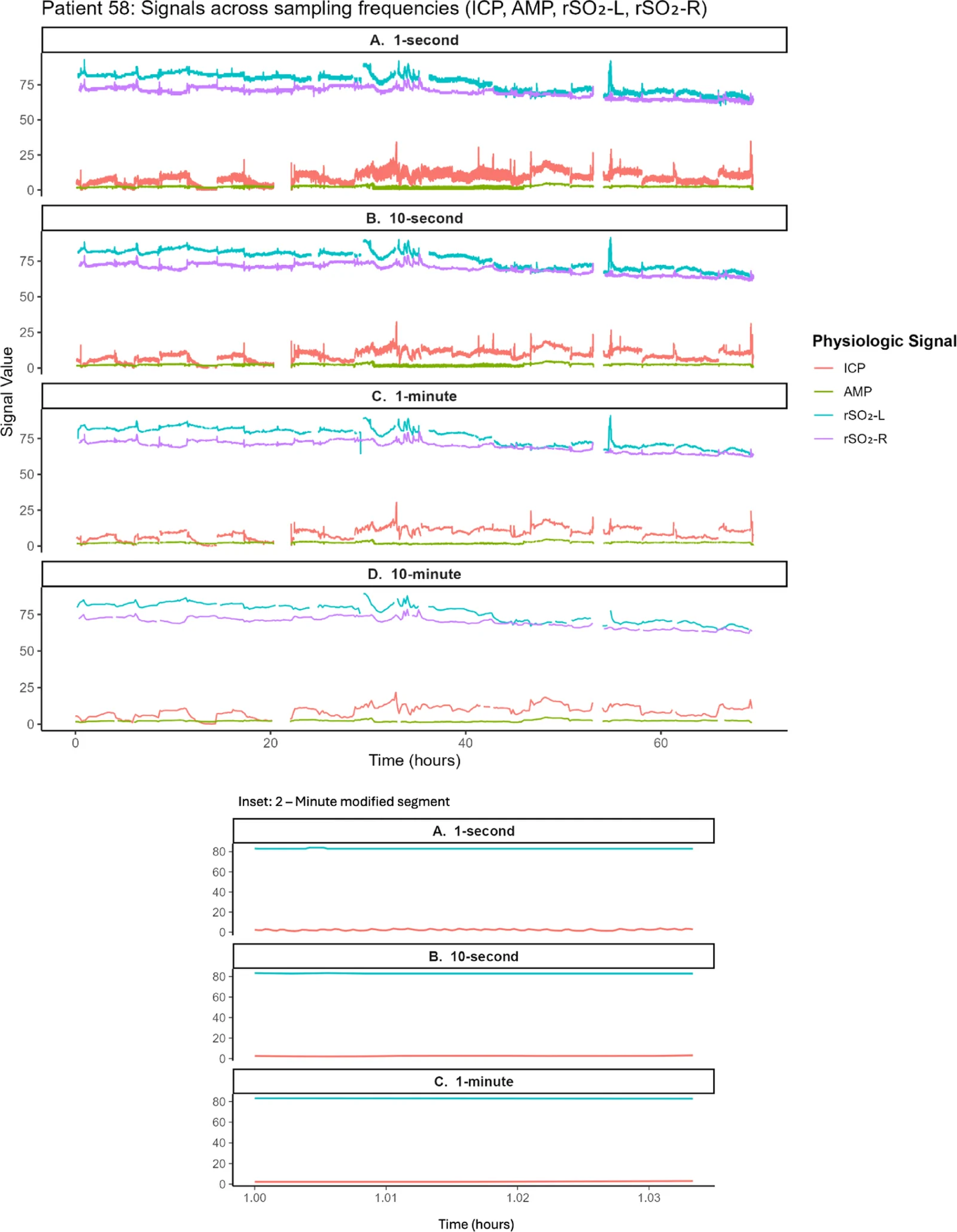
A patient example of downsampling the signals intracranial pressure (ICP), pulse amplitude of intracranial pressure (AMP), and both left and right regional oxygen saturation (rSO_2_). Signals at the 10-second temporal resolution were downsampled to lower temporal resolutions, with the lowest being 10-minute. Panel **A** displays the exported ICM + 1-second signals, panel **B** shows the 10-second temporal resolution directly obtained from ICM+ software, panel **C** displays the 1-minute downsampled signals, and panel **D** displays the 10-minute downsampled signals. Both ICP and AMP are recorded in millimetres of mercury (mmHg), while rSO_2_ is recorded as a percentage since it is the ratio of deoxygenated hemoglobin to the total hemoglobin concentration. Also included is a inset depicting a 2-minute time segment in order to illustrate the effect of temporal downsampling on ICP and rSO_2_ signal morphology

### Statistical data analysis

The data analysis was performed in this study using R statistical software (Version 4.5.0, R Foundation for Statistical Computing, Vienna, Austria), utilizing the following packages: *tidyverse*,* forecast*,* zoo*,* doParallel*,* vars*,* openxlsx*, and *foreach.* All statistical analyses were performed with an alpha of 0.05 for statistical significance. Subsequent correction for multiple comparisons was conducted using Bonferroni methodology, with p-values maintaining significance after correction denoted with an asterisk within the reported results and appendices.

#### Evaluation and comparison of independent statistical features of ICP, AMP, and rSO_2_

##### Variance calculations

First, we independently evaluated differences in basic signal structure variance between ICP/AMP and rSO_2_. At three different temporal resolutions (1-second, 10-second, and 1-minute), non-overlapping data windows were made for each of the included signals ICP, AMP, and rSO_2_. The included window sizes were 1-minute, 5-minute, 15-minute, 30-minute, and 1-hour. Within each of the different windows the mean absolute error (MAE), mean squared error (MSE), and the root mean squared error (RMSE) were calculated for each physiologic signal using the mean signal value within that widow size as the reference value. To account for differences in the scale and units of the physiologic variables, variance metrics were normalized by the corresponding window mean prior to statistical comparison. For each patient, temporal resolution, and signal, median values of these variance metrics across the employed windows were calculated and used for population level comparisons. Bonferroni correction was applied to adjust the significance threshold for multiple comparisons performed across temporal frequencies, window sizes, physiologic signals, and variance metrics. Wilcoxon signed-rank testing was conducted to assess whether MAE, MSE, and RMSE distributions differed between signal types across window sizes and temporal resolutions.

##### Autoregressive integrated moving average (ARIMA) modelling

Second, to further explore the independent statistical signal structures of ICP/AMP and rSO_2_, a formal assessment of ARIMA time-series structures was conducted. ICP, AMP, and rSO_2_ data streams were analyzed at all the included temporal frequencies. Prior to modelling and differencing, artefacts were removed from the data streams. Before constructing the optimal time-series ARIMA model, it was necessary to determine the stationarity of each time-series. Both Augmented Dickey-Fuller (ADF) and Kwiatkowski-Phillips-Schmidt-Shin (KPSS) tests were used to perform stationarity analysis for each physiological signal at the individual patient level [31]. Differencing orders of 0, 1, 2, 3, and 4 were tested for each patient to determine the optimal differencing order to reach stationarity. After differencing, ARIMA models were then constructed for each patient and signal across the included temporal resolutions. The optimal autoregressive (p-order) and moving average (q-order) orders ranging from 0 to 10 were tested. The optimal ARIMA model was primarily selected by minimizing the Akaike Information Criterion (AIC), with Bayesian Information Criterion (BIC) and log-likelihood (LL) also recorded and used as secondary measures in order to confirm model suitability without unnecessary complexity. The optimal ARIMA parameters for each signal and subject were recorded. At each of the included temporal resolutions, a median optimal ARIMA model order was determined for each signal for population level comparisons between ICP, AMP, and rSO_2_. This is in keeping with prior works from our group [19, 32–35]. Differences in median optimal ARIMA orders were then compared between ICP/AMP and rSO_2_ via Wilcoxon signed-rank testing. Further Bonferroni correction was utilized to adjust the significance threshold for multiple comparisons within each of the reported temporal resolutions.

##### Subgroup analysis – variance

Third, a subgroup analysis using the included patient demographic data was completed to test to see if any differences in the calculated variance metrics existed between different subgroups. The categories included are as follows: Total population, ICP monitor on the same side as NIRS, ICP monitor on the opposite side of NIRS, primary DC, secondary DC, no DC, GCS of 8 or less, GCS of 9–12, bilateral reactive pupil response, bilateral unreactive pupil response, unilateral unreactive pupil response, Rotterdam CT Grade of 1, Rotterdam CT Grade of 2, Rotterdam CT Grade of 3, Rotterdam CT Grade of 4, Rotterdam CT Grade of 5, Rotterdam CT Grade of 6, Marshall CT Grade of II, Marshall CT Grade of III, Marshall CT Grade of IV, Marshall CT Grade of V, age under 40, age between 40 and 60, age over 60, male, and female.

#### Evaluation of temporal interdependencies of ICP, AMP, MAP and rSO_2_

##### Correlation analysis – ICP/AMP and rSO_2_

Next, to facilitate evaluating temporal interdependency of ICP/AMP and rSO_2_, Pearson correlation analysis was performed at 1-second, 10-second, 1-minute, and 10-minute temporal resolutions using varying window sizes in order to quantify a linear association between the following pairs of physiologic signals: ICP and rSO_2_ (left and right) and AMP and rSO_2_ (left and right). Correlation analyses were performed independently for each patient, with both the Pearson correlation coefficient (r) and corresponding p-value being obtained for every patient at each temporal resolution and window size. Patient specific correlation coefficients were then subsequently summarized at the population level. Bonferroni correction was then applied within each of the reported temporal resolutions.

##### Cross-correlation, covariance, and granger causality analysis – ICP/AMP and rSO_2_

To further characterized the temporally resolved interdependencies between ICP/AMP and rSO_2_, cross-correlation functions (CCF) were computed for different pairs of signals at 1-second, 10-second, 1-minute, and 10-minute temporal resolutions and different window sizes. The CCF were computed for the following pairs: ICP to rSO_2_ (left and right), and AMP to rSO_2_ (left and right). For each patient, CCF between the signals was calculated in order to assess the similarity of the signal pairs as a function of time lag, allowing identification of whether changes in one signal preceded or followed changes in the other. For each patient, the strength of the relationship (r) and the temporal delay between the variables (lag) were recorded. Subsequent population-level analysis occurred using median and IQR values.

Covariance analysis was performed using the same temporal resolutions, window sizes, and pairs of signals, as listed above. For each patient, the covariance between the signals was determined, giving insight into the degree of shared variability among the signal pairs. Similarly, median and IQR values were then calculated in order to perform population-level analysis.

Using the same temporal resolutions, window sizes, and signal pairs, as listed above, a bidirectional Granger causality test was performed in order to assess the predictive relationships between the signals. For each signal pairing, patient, and temporal resolution, F-statistics and p-values were calculated to determine the causal direction. These values were then summarized at the population level to give a percentage of the F-statistic values that were statistically significant for each of the relationships.

##### Vector autoregressive integrated moving average (VARIMA) models and impulse response function (IRF)

Finally, to evaluate the temporal relationship between systemic driving pressures for pulsatile CBV changes (i.e. MAP) and the recorded cranial physiology that is often interpreted as a surrogate for CBV (i.e. ICP/AMP and rSO_2_), formal VARIMA IRF analysis was conducted. Similar to methodology utilized for ARIMA analysis, prior to the VARIMA modelling, stationarity was assessed utilizing ADF and KPSS testing, with first-order differencing being applied before model fitting to reach stationarity, and confirmed with both ADF and KPSS testing. The ICP, AMP, MAP, and rSO_2_ data streams were further analyzed at all of the included temporal frequencies. During the ARIMA model construction, stationarity of each of the variables had been assessed, and subsequently a first-order differenced series was utilized for VARIMA model construction to remove non-stationary trends and stabilize the variables over time. Unlike ARIMA which models the temporal behaviour of an individual signal, VARIMA evaluates how signal pairs interact and influence one another over time [36, 37]. VARIMA models were constructed for the following pairs of variables: MAP and ICP, MAP and AMP, MAP and rSO_2_ (left and right), ICP and rSO_2_ (left and right), and AMP and rSO_2_ (left and right). In this case, MAP was included in the analysis as a common variable to examine how ICP and rSO_2_ respond to the same driving pressure signal. The optimal autoregressive and moving average orders for each VARIMA model were determined by testing candidate p-order and q-order combinations within a range that was constrained by the previously identified ARIMA orders for each signal [36, 37]. Final model selection was primarily based on minimizing the BIC, with AIC and LL also being recorded and utilized as secondary measures [36, 37]. The optimal VARIMA parameters for each signal and subject were recorded. At each of the included temporal resolutions, a median optimal VARIMA model order was determined for each signal pairing for population level comparisons. IRFs were then generated to assess the temporal direction and dependence of each of the relationships.

##### Subgroup analysis – correlation, cross-correlation, co-variance, and granger causality

Third, a subgroup analysis using the included patient demographic data was completed to test to see if any differences in the results from the tests performed existed between different subgroups. The categories included are as follows: Total population, ICP monitor on the same side as NIRS, ICP monitor on the opposite side of NIRS, primary DC, secondary DC, no DC, GCS of 8 or less, GCS of 9–12, bilateral reactive pupil response, bilateral unreactive pupil response, unilateral unreactive pupil response, Rotterdam CT Grade of 1, Rotterdam CT Grade of 2, Rotterdam CT Grade of 3, Rotterdam CT Grade of 4, Rotterdam CT Grade of 5, Rotterdam CT Grade of 6, Marshall CT Grade of II, Marshall CT Grade of III, Marshall CT Grade of IV, Marshall CT Grade of V, age under 40, age between 40 and 60, age over 60, male, and female.

## Results

### Patient population demographics

In total, there were 118 patients included in this study. The demographic data of this patient population is summarized in Table 1. It is important to note that when combined, the percentages of ICP location being on the same side as the NIRS signals and the percentages of ICP location being on the opposite side as the NIRS signals are over 100%. This is because some of the patients had viable data from both the right and left NIRS sensors, meaning they fall into both categories.

**Table 1:** Cohort demographics

Variable		Median (IQR) or Number (%)
Age		41 (28–54)
Male Patients		94 (79.7)
ICP Monitor Location	Same Side as NIRS	113 (95.8)
	Opposite Side as NIRS	117 (99.2)
Decompressive Craniotomy	None	82 (69.5)
	Primary	29 (24.6)
	Secondary	7 (5.9)
Admission GCS	Total	6 (4–8)
Admission Pupil Exam	Bilaterally Unreactive	15 (12.7)
	Unilaterally Unreactive	26 (22)
	Bilaterally Reactive	77 (65.3)
Rotterdam CT Score	Total	4 (4–5)
Admission Marshall CT Score	II	3 (2.5)
	III	38 (32.2)
	IV	18 (15.3)
	V	59 (50)

CT computed tomography, GCS Glasgow Coma Scale, ICP intracranial pressure, IQR interquartile range, N number of subjects, NIRS near infrared spectroscopy

### Evaluation and comparison of independent statistical features of ICP, AMP, and rSO_2_

#### Variance analysis across temporal resolution and data window size

Variance characteristics of comparisons between different physiologic variables were compared across three different temporal resolutions and five different window sizes using the metrics MAE, MSE, and RMSE (Appendix A, Table A). The variable comparison groups consisted of ICP vs. rSO_2__left (L), ICP vs. rSO_2__right (R), AMP vs. rSO_2__L, and AMP vs. rSO_2__R. During this analysis temporal resolutions of 1-second, 10-second, and 1-minute were used while the window sizes consisted of 1-minute, 5-minute, 15-minute, 30-minute, and 1-hour.

**Fig. 3:**
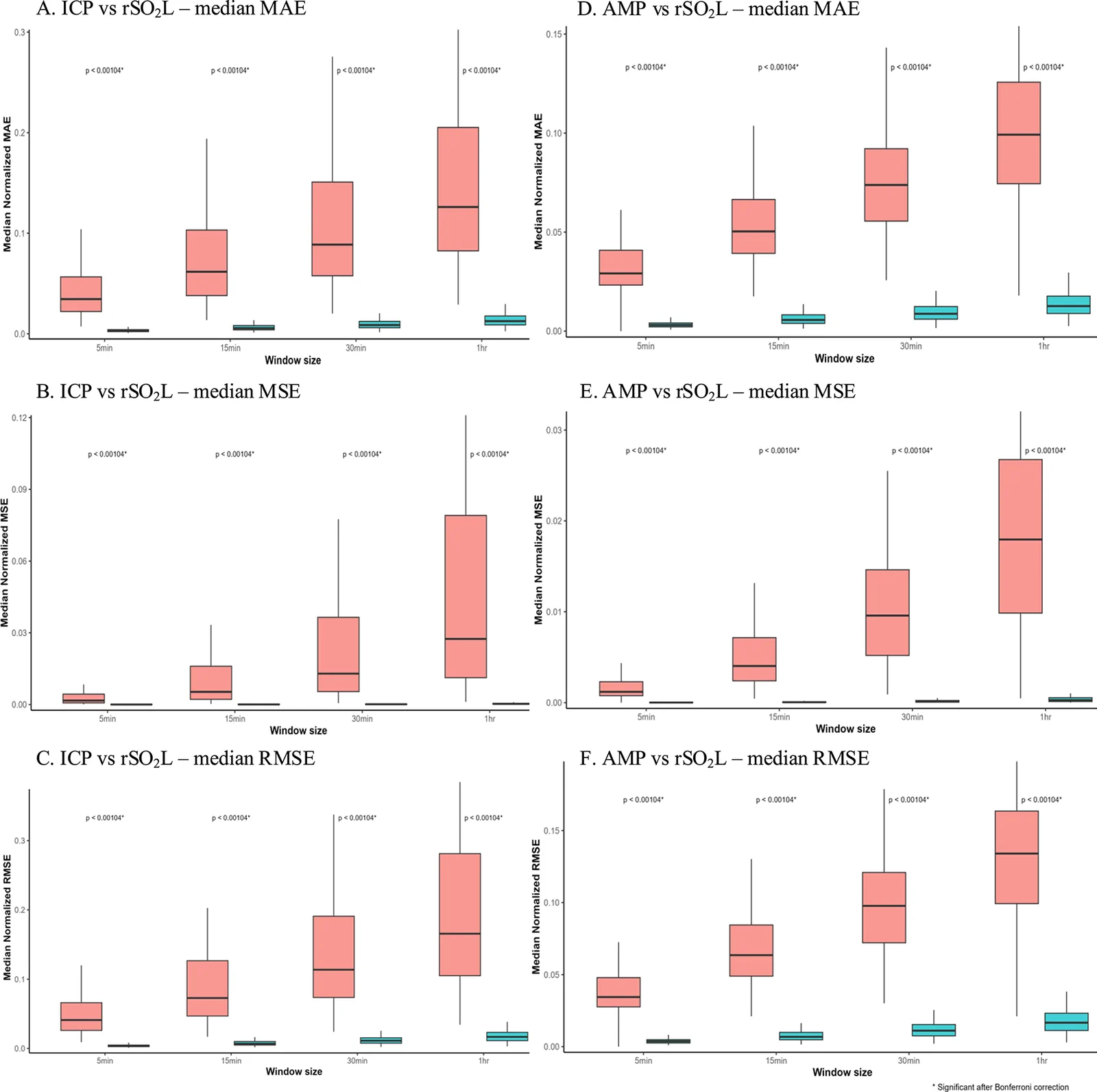
**A**-**C** show the median variance metrics of ICP, displayed in pink, compared to the median variance metrics of left sided rSO_2_, displayed in blue. Panel A compares the median mean average error (MAE), panel B compares the median mean squared error (MSE), and panel C compares the median root mean squared error (RMSE) between the two signals. **D**-**F** show the median variance metrics of AMP, displayed in pink, compared to the median variance metrics of left sided rSO_2_, displayed in blue. Panel D compares the MAE, panel E compares the MSE, and panel F compares the RMSE between the two signals

Following normalization of the variance metrics, Wilcoxon signed-rank testing demonstrated statistically significant differences between all physiologic variable comparison groups. Furthermore, this was demonstrated across every temporal resolution, window size, and variance metric examined. These differences remained significant following Bonferroni correction (Appendix A, Table A). Figure 3 illustrates the distributions of the normalized MAE, MSE, and RMSE across window sizes at the 1-minute resolution for all included variable comparison groups.

Although statistically significant differences remained throughout the analyses, the magnitude of these differences became progressively smaller at lower temporal resolutions and longer window sizes (Appendix A, Fig. 3). This suggests that there is an increasingly similar normalized variance behaviour as the temporal resolution decreases and window size increases.

#### ARIMA statistical features of ICP and rSO_2_ signals

The optimal ARIMA models were identified for different physiologic variables at temporal frequencies of 1-second, 10-second, and 1-minute. The physiologic variables investigated included ICP, AMP, rSO_2__L, and rSO_2__R. Population level summaries of the optimal autoregressive (p-order) and moving average (q-order) orders are presented in Table 2.

**Table 2:** Median optimal ARIMA orders and Wilcoxon Sign Rank test comparing the p-order and q-order between ICP and rSO_2_ and AMP and rSO_2_, with differencing (d-order) orders held at 1

Physiologic Comparison	Optimal *p* Order			Optimal q Order		
	ICP/AMP Median	rSO_2_ Median	*p*-value	ICP/AMP Median	rSO_2_ Median	*p*-value
**1-Second Temporal Resolution**						
**ICP vs.** rSO_2_**_L**	9	5	< 0.001*	9	6	< 0.001*
**ICP vs.** rSO_2_**_R**	9	5	< 0.001*	9	6	< 0.001*
**AMP vs.** rSO_2_**_L**	8	5	< 0.001*	8	6	0.075
**AMP vs.** rSO_2_**_R**	8	5	< 0.001*	8	6	0.544
**10-Second Temporal Resolution**						
**ICP vs.** rSO_2_**_L**	4	2	< 0.001*	4	3	0.837
**ICP vs.** rSO_2_**_R**	4	2	**0.027**	4	3	0.660
**AMP vs.** rSO_2_**_L**	2	2	0.485	3	3	**0.038**
**AMP vs.** rSO_2_**_R**	2	2	0.702	3	3	0.095
**1-Minute Temporal Resolution**						
**ICP vs.** rSO_2_**_L**	1	1	0.764	2	2	0.320
**ICP vs.** rSO_2_**_R**	1	1	0.271	2	2	0.746
**AMP vs.** rSO_2_**_L**	1	1	0.255	2	2	0.467
**AMP vs.** rSO_2_**_R**	1	1	0.159	2	2	0.042

AMP pulse amplitude of intracranial pressure, ICP intracranial pressure, rSO_2__L left sided regional oxygen saturation, rSO_2__Rright sided regional oxygen saturation

Bolded values denote significant p-values prior to Bonferroni analysis, and an asterisk denotes p-values which remain significant post Bonferroni analysis

Prior to ARIMA model construction, signals from all four of the physiologic variables needed to be differenced in order to achieve stationarity. For the vast majority of the patients, first-order differencing (d-order = 1) was sufficient in achieving stationarity across the signals and temporal resolutions. This was determined by using both ADF and KPSS testing, as seen in Appendix B (Tables B.1.1 – B.1.9).

After differencing, optimal ARIMA models were identified per patient across signals and temporal resolutions by testing p-orders and q-orders of 0–10. Optimal model selection was based primarily on minimization of AIC, with BIC and LL being used as secondary measures of model suitability. A patient-level example on the selection process for optimal ARIMA models, as well as the corresponding autocorrelation function (ACF) and partial autocorrelation function (PACF) plots which confirm appropriate model fitting can be seen in Appendix B (Tables B.2.1 – B.2.4).

In general, at the 1-second temporal resolution, median p-orders were higher for ICP (9) when compared to both rSO_2__L and rSO_2__R (5) (Table 2). Similarly median q-orders were also higher for ICP (9) when compared to rSO_2_ (6) (Table 2). When a Wilcoxon signed-rank test was used, it was determined that differences in both the p-orders and q-orders between ICP and rSO_2_ were statistically significant (Table 2). When looking at the relationship between AMP and rSO_2_, median p-orders were higher for AMP (8) than p-orders for rSO_2_ (5) (Table 2). Similar findings were found among the q-orders, with higher values being found for AMP (8) when compared to rSO_2_ (6) (Table 2). After the completion of a Wilcoxon signed-rank test, it was determined that differences in the p-orders of AMP and rSO_2_ were statistically significant; however, it was determined that there was no statistical significance in the difference in q-orders between AMP and rSO_2_ (Table 2).

At the 10-second temporal resolution, it was also found that median p-orders were higher for ICP (4) when compared to both rSO_2__L and rSO_2__R (2) (Table 2). Median q-orders were slightly higher for ICP (4) when compared to rSO_2_ (3) (Table 2). After the Wilcoxon signed-rank test, it was determined that differences in the p-orders between ICP and rSO_2_ were statistically significant (Table 2). Despite this, the Wilcoxon signed-rank test also showed that differences in the q-orders between ICP and rSO_2_ were not statistically significant (Table 2). When looking at the relationship between AMP and rSO_2_, median p-orders were very similar between AMP (2) and p-orders for both rSO_2__L and rSO_2__R (2) (Table 2). Similar findings were found among the q-orders. Furthermore, the Wilcoxon signed-rank test determined that differences in the p-orders and q-orders of AMP and rSO_2_ were not statistically significant, except with regards to the q orders in the AMP to rSO_2__L relationship (Table 2).

At the 1-minute temporal resolution, it was found that the median p-orders converged across ICP (1), AMP (1), rSO_2__L (1), and rSO_2__R (1) (Table 2). Similarly, it was found that the median q-orders also converged across ICP (2), AMP (2), rSO_2__L (2), and rSO_2__R (2) (Table 2). The subsequent Wilcoxon signed-rank test determined that there were no statistically significant differences in the optimal p-orders and q-orders between any of the physiological variables (Table 2).

#### Variance sub-group analysis

Sub-group analysis was performed in order to determine if the calculated variance metrics differed amongst the following categories: age, biologic sex, admission Marshall CT grade, Rotterdam CT score, pupil reactivity, admission GCS, and ICP monitor location. Across all of the subgroups, there were no consistent differences in the observed variance metrics across physiological variables, window sizes, or the temporal frequencies that were examined. Sub-group analysis results can be found in Appendix C.

### Evaluation of temporal interdependencies of ICP, AMP, MAP, and rSO_2_

#### Correlation analysis – ICP/AMP and rSO_2_

Pearson correlation coefficients were calculated in order to quantify the linear relationship between physiologic signal pairs across four different temporal resolutions and six different window sizes (Appendix D, Table D). Comparisons were performed between ICP and rSO_2__L, ICP and rSO_2__R, AMP and rSO_2__L, and AMP and rSO_2__R using temporal resolutions of 1-second, 10-seconds, 1-minute, and 10-minutes, with window sizes of 1-minute, 5-minutes, 15-minutes, 30-minutes, 1-hour, and 6-hours.

At the 1-second temporal resolution, it was observed that throughout all of the window sizes, there was a weak correlation between both ICP and rSO_2_ as well as AMP and rSO_2_ signals (Appendix D, Table D). For example, at the 30-minute window size, the correlation between ICP and rSO_2_ was determined to be 0.048 (Appendix D, Table D). Although many of these correlations were not statistically significant at smaller window sizes, as the window sizes increased more of the Pearson correlation values became significant.

At both the 10-second and 1-minute temporal resolutions, the strength of the relationship between ICP and rSO_2_ signals began to increase and became moderately positive (Appendix D, Table D). Furthermore, it was determined that at the 30-minute window size and 10-second resolution Pearson correlation values became significant (0.094 for the linear relationship between ICP and rSO_2__R) (Appendix D, Table D). Within these two temporal resolutions, it was also noted that AMP displayed less consistent and weaker correlations with rSO_2_ signals across all of the window sizes (Appendix D, Table D).

At the 10-minute temporal resolution, moderate correlations between values of ICP and values of rSO_2_ continued to be seen across all of the included window sizes. However, these relationships were not deemed statistically significant (Appendix D, Table D). For example, at the 10-minute temporal resolution and 3-hour window size, the Pearson correlation was determined to be 0.139 between ICP and rSO_2__R, however this was not deemed to be statistically significant (*p* = 0.123) (Appendix D, Table D). Similar to what was seen with other temporal resolutions, AMP continued to display less consistent and weaker correlations with rSO_2_ signals across all of the window sizes when compared to the ICP vs. rSO_2_ relationship (Appendix D, Table D).

#### Cross correlation analysis - ICP/AMP and rSO_2_

Cross-correlation functions (CCF) were computed in order to investigate both the strength of the relationship and the lag between the following sets of physiologic variables: ICP vs. rSO_2__L, ICP vs. rSO_2__R, AMP vs. rSO_2__L, and AMP vs. rSO_2__R. This was completed across the same four temporal resolutions and seven window sizes. The population summary table can be seen in Appendix F.

It was observed that at the 1-second temporal resolution, the values for the median maximum CCF values were fairly low and inconsistent across all of the investigated window sizes for both ICP vs. rSO_2_ and AMP vs. rSO_2_ (Appendix F, Table F). Additionally, the median lag displayed variability in not only the relationship examined, but also the particular window size (Appendix F, Table F).

At the 10-second temporal resolution, values for the cross correlation for both ICP vs. rSO_2_ and AMP vs. rSO_2_ began to increase; however, these did end up decreasing as the window sizes got larger (Appendix F, Table F). It was also determined that several of the investigated relationships demonstrated peak cross correlation at extended negative lag values, with some reaching lag − 10 (Appendix F, Table F), suggesting that one physiologic signal consistently preceded the other across multiple sampling intervals.

Lastly, at both the 1-minute and 10-minute temporal resolutions, the cross-correlation strength between ICP and rSO_2_ was moderately strong and remained fairly consistent across all of the window sizes tested (Appendix F, Table F). Moreover, the median lag amongst the ICP vs. rSO_2_ relationship was consistently calculated to be lag 0 across all of the window sizes for both temporal resolutions (Appendix F, Table F). Despite this, when looking at the cross correlation strength between AMP and rSO_2_, it was determined to be both weaker as well as having many inconsistencies across the window sizes (Appendix F, Table F).

#### Granger causality analysis - ICP/AMP and rSO_2_

Bidirectional Granger causality testing was performed in order to investigate the predictive relationships between ICP and rSO_2_ as well as AMP and rSO_2_. This was done across the same four temporal resolutions and seven window sizes as previously discussed. A population level summary on the proportion of patients that displayed a statistically significant Granger predictive result can be seen in Table 3.

**Table 3:** Population summary of Granger causality analysis across different temporal resolutions, window sizes, and physiologic relationships

		**Proportion Significant in Population (p < 0.05)**						
**Physiologic Relationship**	**Direction**	**1min**	**5min**	**15min**	**30min**	**1hr**	**3hr**	**6hr**
**1-Second Temporal resolution**								
**ICP vs ** **rSO**_**2**_ **_L**	**ICP→ ** **rSO**_**2**_ **_L**	10.3%	26.5%	48.4%	61.6%	71.8%	82.4%	86.4%
	**rSO**_**2**_ **_L ** **→** **ICP**	10.2%	12.5%	20.8%	29.7%	41.2%	59.7%	67.6%
**ICP vs ** **rSO**_**2**_ **_R**	**ICP ** **→** **rSO**_**2**_ **_R**	10.5%	27.0%	49.4%	63.5%	73.8%	83.5%	86.5%
	**rSO**_**2**_ **_R** **→** ** ICP**	10.1%	12.3%	20.3%	30.5%	42.6%	60.4%	69.2%
**AMP vs ** **rSO**_**2**_ **_L**	**AMP ** **→** **rSO**_**2**_ **_L**	21.5%	36.7%	50.9%	59.8%	68.3%	77.2%	81.8%
	**rSO**_**2**_ **_L ** **→** ** AMP**	13.4%	14.0%	20.8%	29.2%	39.5%	56.0%	63.2%
**AMP vs ** **rSO**_**2**_ **_R**	**AMP ** **→** **rSO**_**2**_ **_R**	21.8%	37.1%	51.5%	60.9%	68.5%	77.7%	81.0%
	**rSO**_**2**_ **_R** **→** ** AMP**	13.3%	13.8%	20.6%	28.6%	40.0%	57.0%	64.2%
**10-Second Temporal resolution**								
**ICP vs ** **rSO**_**2**_ **_L**	**ICP ** **→** **rSO**_**2**_ **_L**	–	16.3%	54.6%	64.7%	73.6%	82.8%	86.6%
	**rSO**_**2**_ **_L** **→** ** ICP**	–	14.5%	23.0%	28.6%	37.2%	54.2%	60.8%
**ICP vs ** **rSO**_**2**_ **_R**	**ICP ** **→** **rSO**_**2**_ **_R**	–	16.0%	55.9%	65.6%	74.0%	81.7%	84.9%
	**rSO**_**2**_ **_R** **→** ** ICP**	–	14.5%	23.6%	28.5%	36.5%	53.2%	60.9%
**AMP vs ** **rSO**_**2**_ **_L**	**AMP ** **→** **rSO**_**2**_ **_L**	–	15.9%	46.1%	54.6%	62.6%	73.0%	77.0%
	**rSO**_**2**_ **_L** **→** ** AMP**	–	14.4%	20.4%	24.9%	32.0%	45.4%	55.6%
**AMP vs ** **rSO**_**2**_ **_R**	**AMP ** **→** **rSO**_**2**_ **_R**	–	16.1%	47.2%	56.3%	64.9%	74.1%	79.4%
	**rSO**_**2**_ **_R** **→** ** AMP**	–	14.4%	20.7%	25.3%	32.1%	46.5%	54.7%
**1-Minute Temporal resolution**								
**ICP vs ** **rSO**_**2**_ **_L**	**ICP ** **→** **rSO**_**2**_ **_L**	–	–	14.2%	15.9%	34.1%	50.9%	58.7%
	**rSO**_**2**_ **_L** **→** ** ICP**	–	–	12.3%	15.8%	29.2%	45.4%	52.5%
**ICP vs ** **rSO**_**2**_ **_R**	**ICP ** **→** **rSO**_**2**_ **_R**	–	–	14.2%	16.8%	33.7%	49.8%	57.5%
	**rSO**_**2**_ **_R** **→** ** ICP**	–	–	11.9%	15.6%	29.2%	45.4%	52.5%
**AMP vs ** **rSO**_**2**_ **_L**	**AMP** **→** **rSO**_**2**_ **_L**	–	–	13.1%	16.4%	30.3%	45.4%	54.3%
	**rSO**_**2**_ **_L ** **→** ** AMP**	–	–	10.8%	15.6%	24.6%	37.8%	44.0%
**AMP vs ** **rSO**_**2**_ **_R**	**AMP ** **→** **rSO**_**2**_ **_R**	–	–	13.1%	16.0%	29.5%	44.5%	54.2%
	**rSO**_**2**_ **_R** **→** ** AMP**	–	–	11.0%	15.4%	23.3%	36.5%	43.4%
**10-Minute Temporal resolution**								
**ICP vs ** **rSO**_**2**_ **_L**	**ICP ** **→** **rSO**_**2**_ **_L**	–	–	–	–	–	16.7%	22.9%
	**rSO**_**2**_ **_L ** **→** ** ICP**	–	–	–	–	–	15.8%	21.4%
**ICP vs ** **rSO**_**2**_ **_R**	**ICP ** **→** **rSO**_**2**_ **_R**	–	–	–	–	–	18.4%	27.1%
	**rSO**_**2**_ **_R** **→** ** ICP**	–	–	–	–	–	14.1%	20.9%
**AMP vs ** **rSO**_**2**_ **_L**	**AMP ** **→** **rSO**_**2**_ **_L**	–	–	–	–	–	15.0%	21.1%
	**rSO**_**2**_ **_L ** **→** ** AMP**	–	–	–	–	–	15.4%	23.0%
**AMP vs ** **rSO**_**2**_ **_R**	**AMP** **→** **rSO**_**2**_ **_R**	–	–	–	–	–	14.7%	21.8%
	**rSO**_**2**_ **_R** **→** **AMP**	–	–	–	–	–	14.4%	20.7%

AMP pulse amplitude of intracranial pressure, ICP intracranial pressure, rSO_2__L left sided regional oxygen saturation, rSO_2__R right sided regional oxygen saturation

In general, it was determined that for both of the relationships, ICP and rSO_2_ as well as AMP vs. rSO_2_, a smaller proportion of the patient population demonstrated significant Granger causality results when compared to larger window sizes at the same frequency (Table 3). This was most notable at the 1-second and 10-second temporal resolutions. For example, when looking at the ICP → rSO_2__L relationship at the 1-second temporal resolution, 10.3% of the patient population displayed a significant Granger predictive result in the 1-minute window size (Table 3). This increased significantly as by the 6-hour window size, 86.4% of the patient population displayed a significant Granger predictive result (Table 3). Although significant bidirectional predictive relationships were observed in some patients, significant ICP → rSO_2_ and AMP → rSO_2_ predictive relationships were observed slightly more frequently when compared to the reverse direction (Table 3). Lastly, throughout the window sizes and temporal resolutions investigated, a higher proportion of the patient population displayed a significant Granger predictive result for the ICP → rSO_2_ relationship than the AMP → rSO_2_ relationship (Table 3).

#### VARIMA analysis

Similar to the ARIMA analysis, VARIMA modelling was performed for sets of different physiologic variables to assess the temporal direction and dependence between the variables at three different temporal resolutions; 1-second, 10-second, and 1-minute. The sets of physiologic variables that were investigated included MAP and ICP, MAP and AMP, MAP and rSO_2_, ICP and rSO_2_, and AMP and rSO_2_. After the use of first-order differencing, determined in the ARIMA analysis, optimal VARIMA models were utilized to generate an IRF analysis. Population level summaries of the peak response magnitude and peak lag for each of the physiological relationships investigated are presented in Table 4.

**Table 4:** Population summary of Impulse Response Function analysis across temporal resolutions of 1-second, 10-second, and 1-minute

Impulse	Response	Median Peak Response (IQR)	Median Peak Lag (IQR)
**1-Second Temporal Resolution**			
**MAP**	**ICP**	0.398 (0.218–0.597)	0.0 (0.0–0.0)
**MAP**	**rSO**_**2**_ **_L**	0.002 (−0.004–0.006)	3 (1.0–4.0)
**MAP**	**rSO**_**2**_ **_R**	0.002 (−0.003–0.005)	1.0 (1.0–3.0)
**MAP**	**AMP**	0.003 (0–0.005)	3.0 (2.0–3.0)
**ICP**	**MAP**	0.33 (−0.534–0.664)	2.0 (1.0–4.0)
**ICP**	**rSO**_**2**_ **_L**	0.002 (−0.006–0.007)	3.0 (1.0–5.0)
**ICP**	**rSO**_**2**_ **_R**	0.004 (−0.004–0.008)	3.0 (1.0–5.0)
**AMP**	**MAP**	−0.104 (−0.212–0.108)	1.0 (0.0–3.0)
**AMP**	**rSO**_**2**_ **_L**	0.004 (0.001–0.009)	3.0 (1.0–4.0)
**AMP**	**rSO**_**2**_ **_R**	0.004 (0.001–0.012)	3.0 (1.0–4.0)
**rSO**_**2**_ **_L**	**MAP**	−0.039 (−0.073 - −0.015)	1.0 (1.0–3.0)
**rSO**_**2**_ **_L**	**ICP**	−0.011 (−0.018 - −0.006)	1.0 (1.0–3.0)
**rSO**_**2**_ **_L**	**AMP**	0 (−0.001–0.001)	4.0 (2.0–5.0)
**rSO**_**2**_ **_R**	**MAP**	−0.041 (−0.077–0.008)	1.0 (1.0–3.0)
**rSO**_**2**_ **_R**	**ICP**	−0.01 (−0.02 - −0.002)	1.0 (1.0–3.75)
**rSO**_**2**_ **_R**	**AMP**	−0 (−0.001–0.001)	3.0 (2.0–4.0)
**10-Second Temporal Resolution**			
**MAP**	**ICP**	−0.08 (−0.347–0.228)	1.0 (0.0–1.0)
**MAP**	**rSO**_**2**_ **_L**	0.027 (−0.044–0.055)	1.0 (0.75–2.0)
**MAP**	**rSO**_**2**_ **_R**	0.028 (−0.039–0.064)	1.0 (0.0–2.0)
**MAP**	**AMP**	−0.023 (−0.051 - −0.008)	1.0 (1.0–1.0)
**ICP**	**MAP**	0.193 (0.099–0.366)	1.0 (1.0–2.0)
**ICP**	**rSO**_**2**_ **_L**	0.049 (−0.04–0.093)	1.0 (1.0–2.0)
**ICP**	**rSO**_**2**_ **_R**	0.052 (−0.032–0.106)	1.0 (1.0–2.0)
**AMP**	**MAP**	0.208 (−0.159–0.363)	0.0 (0.0–1.0)
**AMP**	**rSO**_**2**_ **_L**	0.01 (−0.048–0.045)	2.0 (1.0–2.0)
**AMP**	**rSO**_**2**_ **_R**	−0.009 (−0.055–0.049)	1.0 (1.0–2.0)
**rSO**_**2**_ **_L**	**MAP**	0.042 (−0.08–0.088)	2.0 (1.0–4.0)
**rSO**_**2**_ **_L**	**ICP**	−0.022 (−0.06–0.015)	1.0 (1.0–3.0)
**rSO**_**2**_ **_L**	**AMP**	−0.004 (−0.01–0.003)	1.0 (1.0–3.0)
**rSO**_**2**_ **_R**	**MAP**	0.038 (−0.056–0.082)	2.0 (1.0–3.0)
**rSO**_**2**_ **_R**	**ICP**	−0.02 (−0.051–0.014)	1.0 (1.0–3.0)
**rSO**_**2**_ **_R**	**AMP**	−0.004 (−0.01–0.002)	1.0 (1.0–3.0)
**1-Minute Temporal Resolution**			
**MAP**	**ICP**	0.134 (−0.103–0.325)	0.0 (0.0–1.0)
**MAP**	**rSO**_**2**_ **_L**	0.104 (0.048–0.183)	0.0 (0.0–1.0)
**MAP**	**rSO**_**2**_ **_R**	0.097 (0.028–0.165)	0.0 (0.0–1.0)
**MAP**	**AMP**	−0.01 (−0.018–0.005)	1.0 (1.0–2.0)
**ICP**	**MAP**	0.241 (0.087–0.373)	1.0 (1.0–1.0)
**ICP**	**rSO**_**2**_ **_L**	0.102 (−0.036–0.156)	0.0 (0.0–1.0)
**ICP**	**rSO**_**2**_ **_R**	0.091 (0.026–0.159)	0.0 (0.0–1.0)
**AMP**	**MAP**	−0.325 (−0.653–0.231)	0.0 (0.0–0.0)
**AMP**	**rSO**_**2**_ **_L**	−0.023 (−0.105–0.072)	1.0 (0.0–1.0)
**AMP**	**rSO**_**2**_ **_R**	0.007 (−0.105–0.101)	0.0 (0.0–1.0)
**rSO**_**2**_ **_L**	**MAP**	−0.081 (−0.163–0.132)	2.0 (1.0–3.0)
**rSO**_**2**_ **_L**	**ICP**	−0.061 (−0.12 - −0.024)	1.0 (1.0–2.0)
**rSO**_**2**_ **_L**	**AMP**	−0.006 (−0.015–0.002)	1.0 (1.0–2.0)
**rSO**_**2**_ **_R**	**MAP**	−0.081 (−0.165–0.153)	1.0 (1.0–3.0)
**rSO**_**2**_ **_R**	**ICP**	−0.064 (−0.132 - −0.023)	1.0 (1.0–2.0)
**rSO**_**2**_ **_R**	**AMP**	−0.006 (−0.014–0.001)	1.0 (1.0–2.0)

AMP pulse amplitude of intracranial pressure, ICP intracranial pressure, MAP mean arterial pressure, rSO_2__L left sided regional oxygen saturation, rSO_2__Rright sided regional oxygen saturation

At all of the included temporal resolutions, MAP impulses produced more consistent responses to both ICP and rSO_2_ when compared to AMP (Table 4). This suggests a closer temporal similarity between MAP and both ICP and rSO_2_ behaviour when compared to AMP. At the 1-second temporal resolution, the median impulse response of MAP to ICP was 0.398 IQR: (0.218–0.597), which had a median lag of 0 associated with the result (Table 4). By comparison, MAP-related responses in AMP were both weaker and less consistent across all of the included temporal resolutions (Table 4). For example, in the 1-second temporal resolution, the median impulse response of MAP to AMP was 0.003 IQR: (0.000–0.005) with a median lag of 3, while in the 1-minute temporal resolution, the median impulse of MAP to AMP was observed to be −0.01 IQR: (−0.018- 0.005) with a median lag of 1 (Table 4).

As the temporal resolution decreased, the MAP to rSO_2_ relationships became more similar to the MAP to ICP relationships. Although there were some differences in the median impulse response, at the 10-second resolution both the MAP to rSO_2_ and MAP to ICP responses occurred within lag 1 (Table 4). These similarities continued to the 1-minute resolution, as the MAP to rSO_2_ and MAP to ICP relationships produced similar responses. Here, the median impulse response of MAP to ICP was 0.134, while the median impulse response of MAP to rSO_2__L was 0.104, with both of these occurring at lag values of 0 (Table 4).

When comparing the behaviours of ICP and AMP to the behaviour of rSO_2_, ICP demonstrated more consistent similarity to rSO_2_ across the included temporal resolutions when compared to AMP. At the 1-minute temporal resolution, ICP impulses produced median impulse responses of 0.102 and 0.091 for rSO_2__R and rSO_2__L respectively (Table 4). Both of these occurred at a lag value of 0, indicating an immediate response (Table 4). In contrast, at the 1-minute resolution, AMP impulses not only resulted in a weaker response by rSO_2_, but responses also occurred at higher lag values, indicating a less direct relationship between the two (Table 4). For example, in the lower temporal resolutions (1-second), the rSO_2__L→AMP relationship had a median impulse response of 0, with this value being accompanied by lag value of 4 (Table 4).

#### Cross-correlation, co-variance, and granger causality sub-group analysis

Sub-group analysis was performed in order to determine if the cross-correlation, granger causality, or Pearson correlation analysis differed amongst the following categories: Age, biologic sex, admission Marshall CT grade, Rotterdam CT score, pupil reactivity, admission GCS, and ICP monitor location. Across all of the subgroups, there were no consistent differences in the observed results of the statistical tests across physiological variables, window sizes, or the examined temporal resolutions.

## Discussion

In order to characterize and compare the temporal statistical properties of NIRS-based rSO_2_ signals and invasively obtained ICP signals, an array of different statistical methods were employed including variance analysis between the signals, computing the optimal ARIMA and VARIMA orders for each of the included variables, as well as the completion of correlation, cross-correlation, co-variance and granger causality analysis. There are several interesting insights that can be drawn from the analysis of this TBI patient population. Overall, analyses evaluating temporal dependence, including ARIMA modelling, correlation analyses, Granger causality and VARIMA, demonstrated increasing similarity between ICP and rSO_2_ at lower temporal resolutions and longer window sizes. In comparison, AMP and rSO_2_ relationships were generally weaker and less consistent across the same analyses. In contrast to the temporal dependence testing, normalized variance analysis showed that the absolute variability of the two signals remained statistically distinct across all comparisons.

Population level variance analysis demonstrated significant differences in normalized variance metrics between both ICP and rSO2 across every temporal resolution and window size examined (Appendix A, Fig. 3). These findings indicate that the magnitude of normalized signal variability differs between ICP, AMP, and rSO2 even following a Bonferroni correction. Despite this, normalized variance distributions demonstrated that the magnitude of these differences generally become smaller at lower temporal resolutions and longer window sizes. This trend is consistent with the findings observed in the original, non-normalized variance analysis, in which lower temporal resolutions and longer window sizes resulted in increasingly similar variance behaviour between ICP and rSO2. Together, these observations suggest there to be an overall trend toward increasingly similar variance behaviour between ICP and rSO2 at lower temporal resolutions and longer window sizes. Persistent differences in normalized variance may reflect intrinsic physiological differences between ICP and rSO2, as the two signals represent distinct aspects of cerebral physiology. Additionally, these differences may be impacted by the sampling frequencies of the monitoring technologies, with ICP acquired at 100 Hz and commercial NIRS at 1 Hz. Even after downsampling, the ICP signal may retain greater temporal variability than rSO2 because of its higher original sampling frequency.

Results observed during the population level variance analysis were complemented by determining the optimal ARIMA models for the physiologic variables ICP, AMP, and rSO_2_. Unlike the variance analysis, which characterizes differences in signal variability, ARIMA modelling evaluates the temporal statistical structure of each signal. Here, unlike differences in their normalized variance, there was an increasing similarity in optimal ARIMA orders observed at lower temporal resolutions and window sizes. At the 1-second temporal resolution, it was shown that at the population level, there was a statistical difference between optimal p-orders and q-orders of ICP and rSO_2_ (Table 2). At, the 10-second resolution, only the differences in the optimal p-orders between ICP and rSO_2_ were statistically significant, as differences between the optimal q-orders for the variables were deemed not statistically significant through the Wilcoxon signed rank test (Table 2). Furthermore, at the lowest temporal resolution analysed (1-minute), differences were not statistically significant between optimal p-orders and q-orders for ICP, and optimal p-orders and q-orders for rSO_2_ (Table 2). Interestingly, at the lower temporal resolutions tested (10-second and 1-minute), differences in both the optimal p-orders and q-orders between AMP and rSO_2_ were deemed not statistically significant through Wilcoxon signed rank testing (Table 2). These ARIMA results suggest that ICP and rSO_2_ exhibit similar temporal statistical characteristics at lower temporal resolutions.

It is important to note that increased temporal averaging smooths the time-series of each physiological signal by reducing high-frequency variability. As such, some of the convergence that was seen in the ARIMA model structure at lower temporal resolutions may, in part, reflect the mathematical effects of temporal averaging, at lower temporal resolutions, trends from common clinical practices and therapeutic interventions can be accentuated. However, previous investigations of multi-model cerebral physiologic data streams have demonstrated that progressive temporal averaging does not necessarily result in the convergence of optimal ARIMA model orders. Instead, optimal ARIMA structures generally remain both patient and signal-specific despite increased temporal averaging [19, 34]. Therefore, while the temporal averaging may have contributed to the observed convergence in ARIMA model structure, it can’t fully explain these findings. As such, the observed convergence in ARIMA structure between ICP and rSO2 at lower temporal resolutions may reflect similarities in the temporal statistical behaviour, although it is not definitive evidence of direct physiological coupling.

The completion of correlational analysis as well as causal and cross dependence testing further supported the idea that ICP and rSO_2_ signals are related to one another. Pearson correlation analysis indicated a moderate correlation between ICP and rSO_2_ at lower temporal resolution (1-minute and 10-minute) and mid-range window sizes (Appendix D). It is important to note that Pearson correlations were calculated independently for each patient and subsequently summarized at the population level. While Pearson correlations provide a simple measure of linear association, it does not account for the repeated nature of physiologic measurements within individual patient. Therefore, Pearson correlation was interpreted alongside the cross-correlation, Granger causality, and VARIMA analyses, providing more information towards the temporal and directional relationships between the signals. The results from the CCF analysis indicated that as temporal resolution decreased, the strength of the relationship between ICP and rSO_2_ continued to strengthen and became more consistent across the included window sizes (Appendix F). Additionally, these correlational relationships were accompanied with a lag value of 0, indicating almost no time-delay between the two signals (Appendix F). Furthermore, Granger causality testing indicated that although some bidirectional predictability was identified, the ICP → rSO_2_ relationship was favoured among the population level results (Table 3). Here it was determined that as window sizes became larger, a higher proportion of the population displayed significant Granger predictive results in the relationship of ICP → rSO_2_ (Table 3). It is also important to note that throughout the majority of these tests, the AMP vs. rSO_2_ relationship displayed much weaker and less consistent results when compared to the ICP vs. rSO_2_ relationship. Collectively, results from the correlation and Granger analyses indicate that ICP and rSO_2_ not only share statistical association, but also demonstrate directional temporal predictability, particularly at lower temporal resolutions.

Through IRF analysis using VARIMA modelling, we were able to further demonstrate the temporal relationship between MAP, rSO_2_, and ICP. Similar to the other analysis performed, it was noted that the impulse responses increased as the particular temporal resolution decreased (Table 4). Furthermore, as the temporal resolution decreased, these responses began occurring at smaller lag values, indicating a that physiologic variable were more tightly coupled to one another at lower temporal resolutions (Table 4). Notably, in the 1-minute temporal resolution, it was found that the impulse of MAP on ICP and the impulse of MAP on rSO_2_ were very similar (Table 4). Additionally, the impulse of ICP on rSO_2_ continued to increase as temporal resolution decreased, with ICP impulses resulting in an almost instant response in rSO_2_ at the 1-minute temporal resolution (Table 4). On the other hand, it is important to note that across the different temporal resolutions investigated, the impulse response of AMP to rSO_2_ was not only weaker, but less consistent and occurring at higher lag values when compared to both ICP and MAP (Table 4). These results further support the presence of shared temporal behaviour between ICP and rSO_2_, particularly at lower temporal resolutions. The inclusion of MAP within the VARIMA analysis demonstrated that both ICP and rSO_2_ exhibited similar responses to changes in systemic arterial pressure. However, this finding may reflect the influence of a common systemic driver that acts on both the ICP and rSO_2_ signal, rather than a direct physiological relationship between the two. As such, these results should be interpreted as evidence of shared temporal dynamics, and not proof of a direct relationship between ICP and rSO_2_.

Finally, subgroup analysis grouped by age, biologic sex, admission Marshall CT grade, Rotterdam CT score, pupil reactivity, admission GCS, and ICP monitor location did not indicate any significant differences in the obtained results. This suggests that the observed relationship between ICP and rSO_2_ signals is consistent across the current TBI patient population utilized in this study, regardless of variations in patient demographics.

## Limitations

There are a few limitations with this study that need to be mentioned. First, this study was a retrospective analysis of a relatively large TBI population (largest single center population with co-registered commercial cerebral NIRS, ICP, and ABP data to our knowledge); however, these findings should be considered exploratory in nature. Amongst the included TBI patient population, there was heterogeneity in injury severity and specific therapeutic interventions administered, which may introduce variability that was not captured by the subgroup analysis. Second, the TBI patient population included in this analysis is from a single Canadian center. Different regional factors could limit the generalizability of these findings when compared to other regions and patient populations where injury management may differ. Third, artefacts can arise through numerous causes in a clinical setting, including but not limited to subject movement, equipment malfunctions, environmental factors, and sensor adherence problems. Although physiologic signals were cleaned prior to the analysis, and further thresholding was applied, artefactual segments may still have been retained in the data used for subsequent analysis. Fourth, bias could have been introduced in several ways. One potential source of bias could have been introduced through NIRS recordings, as only two different models of the same commercial device were utilized to gather measurements of rSO_2_. Other commercially available NIRS systems could have slight differences in applicability, which was not tested in this analysis. Another way that bias could have been introduced in this analysis was through the employed statistical analyses. The large number of comparisons between variables, across both frequencies and window sizes, could have resulted in the addition of bias. Additionally, because the utilized NIRS devices sample at 1 Hz, rSO_2_ measurements may not capture higher-frequency cerebrovascular oscillations that the invasive ICP monitors capture. This may influence statistical relationships observed at higher temporal resolutions and shorter window sizes, highlighting the need for more high-frequency research-grade NIRS datasets with co-registered ICP recordings. As such, these findings should be interpreted as describing the shared low-frequency temporal behaviour, as the complete spectrum of 100 Hz ICP data was not included. The consistently weaker relationship observed between AMP and rSO_2_ compared with ICP and rSO_2_ warrants further consideration. AMP reflects the pulsatile changes in ICP that are closely associated with cerebral blood volume [38]. As discussed, the maximum 1 Hz sampling frequency of the commercial NIRS device utilized may limit its ability to adequately characterize cardiac-frequency oscillations, and thereby reducing the association between AMP and rSO_2_ that we have observed. However, an additional explanation may relate to the underlying physiology of the signals examined. While AMP primarily reflets pulsatile changes in ICP, ICP represents a broader measure of intracranial physiology, influenced by CBV, tissue compliance, edema, and cerebral perfusion [2, 38]. Since rSO_2_ reflects oxygen saturation, its relationship with ICP may be driven more strongly by these slower metabolic and ischemic processes rather than by pulsatile CBV alone. This may help explain why ICP consistently demonstrated stronger temporal associations with rSO_2_, when compared to the relationship between rSO_2_ and AMP. Finally, another limitation was that the commercial NIRS system only output the calculated rSO_2_ using their proprietary algorithm, and do not output other physiologic variables such as measured O_2_Hb, HHb, and tHb. With only having access to one of the physiologic variables, generalization of our results from the investigation into the relationship between invasive ICP signals and NIRS signals are limited. Hence, it is unknown whether the observed relationship can be extended to other NIRS-based physiologic variables such as O_2_Hb, HHb, and tHb.

## Future directions

Observations demonstrated in this study provide a framework for developing future predictive models that combine NIRS with additional physiological variables, in this case involving rSO_2_ and ICP. It’s important to note that the present findings do not demonstrate real-time non-invasive estimation of ICP, and instead identify the temporal resolutions over which commercial rSO_2_ and invasively obtained ICP exhibit similar statistical behaviour. Before being able to estimate ICP signals utilizing NIRS technology, further research into the direct relationship between these signals needs to be completed. One idea is to examine this relationship using a larger multi-institutional patient population, which would further strengthen in the findings of this study by improving the generalizability of the results. Another idea would be to look at the relationship between ICP and other NIRS variables, such as concentrations of O_2_Hb and HHb, as well as the tHb. By looking into these other relationships, our understanding of the relationship between NIRS-based signals and invasively obtained ICP-based signals can be strengthened. Additionally, future studies regarding cerebral physiology should aim to model ICP using NIRS-based indices such as rSO_2_. Work through the current study has shown a relationship between ICP and rSO_2_, in particular at lower temporal resolution and longer window sizes. Through the modelling of ICP using a variable such as rSO_2_, we can gain confidence in the ability to use NIRS technology in a clinical setting as a non-invasive alternative to obtain measurements of ICP-based cerebral physiology. This carries a few benefits including a lesser financial burden for resources, more accessibility as the need for surgical intervention to monitor ICP would not be needed, and an overall higher spatial resolution by being able to monitor multiple brain regions. As it stands, similarities between the signal characteristics of rSO_2_ and ICP occur at lower temporal resolutions, meaning more research into the relationship at higher frequencies is necessary to allow for the real-time estimation of ICP in a clinical setting. Future studies should also utilize full waveform NIRS data, meaning utilizing NIRS systems with higher temporal resolution. This may help to improve the characterization of pulsatile cerebral physiologic changes, providing insight into the ability to reconstruct the full ICP pulse waveform using NIRS-derived signals. Artefacts remain one of the main limitations of this technology. Cleaning these signals by hand is both time consuming and cumbersome. In the future, the development of a real-time artefact management system would be beneficial. This would allow for the ability to clean and manage the artefacts in real time, not only improving efficiency, but also allowing the data to be used in real-time.

## Conclusion

In this study, a cerebral physiologic data from a population of 118 TBI patients was utilized in order to both characterize and compare the temporal statistical properties of rSO_2_ and ICP, and demonstrate the temporal relationship between the three physiologic signals MAP, rSO_2_ and ICP. Across the analyses performed, ICP and rSO_2_ demonstrated stronger statistical associations primarily at lower temporal resolutions and longer window sizes. While normalized variance analysis indicated that differences in signal variability persisted across all temporal resolutions and window sizes, ARIMA modelling, correlation analysis, Granger causality, and VARIMA impulse response analysis demonstrated that ICP and rSO_2_ share constancy in their statistical and temporal relatedness with one another. These findings demonstrate convergence between ICP and rSO_2_ signal characteristics at lower temporal resolutions, more specifically around the 1-minute temporal resolution. This starts to provide the foundation for future development and evaluation of predictive non-invasive ICP monitoring approaches; however, further research into modelling ICP using rSO_2_ can further strengthen this idea.

## Supplementary Information


Supplementary Material 1


## Data Availability

The datasets used and analysed during the current study are available from the corresponding author on reasonable request.

## Abbreviations

ACF: Autocorrelation function
ADF: Augmented dickey-fuller
AIC: Akaike information criterion
AMP: Pulse amplitude of intracranial pressure
BIC: Bayesian information criterion
CBV: Cerebral blood volume
CCF: Cross correlation function
CT: Computed tomography
GCS: Glasgow Coma Scale
HSC: Health Sciences Centre
ICP: Intracranial pressure
IQR: Interquartile range
KPSS: Kwiatkowski-phillips-schmidt-shin
LL: Log-likelihood
MAE: Mean absolute error
MAP: Mean arterial pressure
MSE: Mean squared error
N: Number of subjects
NIRS: Near infrared spectroscopy
PACF: Partial autocorrelation function
RMSE: Root mean squared error
rSO_2__L: Left sided regional oxygen saturation
rSO_2__R: Right sided regional oxygen saturation
