# Supplementary material for: Exploring the temporal statistical relationship between commercial cerebral near-infrared spectroscopy signals and intracranial pressure in traumatic brain injury
Source: Intensive Care Med Exp. 2026 Aug 27;14:114. doi: 10.1186/s40635-026-00964-8 (PMC13522304; doi:10.1186/s40635-026-00964-8)
Supplement: Supplementary file 1 [file 40635_2026_964_MOESM1_ESM.docx]

**Table of Contents:**

**Appendix A.** Variance Metric comparison between physiologic variables ---------------------------------------------------------------------------------------- 2

**Appendix B.**

**B.1.1.** Population summary of ADF results for non-differenced data. ------------------------------------------------------------------------------------ 13

**B.1.2.** Population summary of ADF results for first-order differenced data. ----------------------------------------------------------------------------- 14

**B.1.3.** Population summary of ADF results for second-order differenced data. ------------------------------------------------------------------------- 14

**B.1.4.** Population summary of ADF results for third-order differenced data. ---------------------------------------------------------------------------- 15

**B.1.5.** Population summary of ADF results for fourth-order differenced data. -------------------------------------------------------------------------- 15

**B.1.6.** Population summary of ADF results for non-differenced data. ------------------------------------------------------------------------------------ 16

**B.1.7.** Population summary of ADF results for first-order differenced data. ----------------------------------------------------------------------------- 17

**B.1.8.** Population summary of ADF results for second-order differenced data. ------------------------------------------------------------------------- 17

**B.1.9.** Population summary of ADF results for third-order differenced data. ---------------------------------------------------------------------------- 18

**B.1.10.** Population summary of ADF results for fourth-order differenced data. ------------------------------------------------------------------------- 19

**B.2.1.** Patient Example – AIC, while fitting ARIMA models (1-minute resolution) ------------------------------------------------------------------- 19

**B.2.2.** Patient Example – BIC, while fitting ARIMA models (1-minute resolution) -------------------------------------------------------------------- 23

**B.2.3.** Patient Example – LL, while fitting ARIMA models (1-minute resolution) --------------------------------------------------------------------- 26

**B.2.4.** Patient example – Choosing ARIMA based on AIC, BIC, and LL at 1-minute Resolution --------------------------------------------------- 30

**Figure B.1.** Patient example of ACF and PACF for ICP --------------------------------------------------------------------------------------------------- 31

**Figure B.2.** Patient example of ACF and PACF for AMP ------------------------------------------------------------------------------------------------- 32

**Figure B.3.** Patient example of ACF and PACF for rSO_2__L ---------------------------------------------------------------------------------------------- 33

**Figure B.4.** Patient example of ACF and PACF for rSO_2__R ---------------------------------------------------------------------------------------------- 34

**Appendix C.** Sub-group analysis for variance metrics across 1-second, 10-second, and 1-minute temporal resolutions. --------------------------------- 40

**Appendix D.** Population summary of Pearson correlation analysis ------------------------------------------------------------------------------------------------ 93

**Appendix E.** Population summary of cross correlation function analysis ---------------------------------------------------------------------------------------- 95

**Appendix A.**

*AMP* pulse amplitude of intracranial pressure, *ICP* intracranial pressure, *MAE* mean absolute error, *MSE* mean squared error, *RMSE* root mean squared error, *rSO_2__L* left sided regional oxygen saturation, rSO_2_*_R* right sided regional oxygen saturation.

**Table A.** Variance metric comparison between physiologic variables using Wilcoxon Signed rank tests through different combinations of temporal frequencies and widow sizes. Bolded values denote significant p-values prior to Bonferroni analysis, and an asterisk denotes p-values which remain significant post Bonferroni analysis.

| **Physiologic** | **Variance Metric** | **Wilcoxon Sign Rank Test Results** | | | | | | | | | | | |
| --- | --- | --- | --- | --- | --- | --- | --- | --- | --- | --- | --- | --- | --- |
| **Variable Comparison** |  | **1-Minute Window Size and 1-Second Temporal Frequency** | | | | | | | | | | | |
|  |  | **Mean** | | | **SD** | | | **Median** | | | **IQR** | | |
|  |  | **Median ICP/AMP** | **Median** rSO_2_ | **p-value** | **Median ICP/AMP** | **Median** rSO_2_ | **p-value** | **Median ICP/AMP** | **Median** rSO_2_ | **p-value** | **Median ICP/AMP** | **Median** rSO_2_ | **p-value** |
| **ICP vs. rSO_2__L** | **MSE** | 0.032775 | 8.99E-05 | **1.94E-20*** | 0.0796218 | 0.000433 | **1.94E-20*** | 0.0096004 | 3.39E-05 | **1.94E-20*** | 0.0161865 | 4.49E-05 | **1.94E-20*** |
|  | **RMSE** | 0.1338946 | 0.0062568 | **1.94E-20*** | 0.107041 | 0.0061642 | **1.94E-20*** | 0.0979817 | 0.0058252 | **1.94E-20*** | 0.0752452 | 0.0051103 | **1.94E-20*** |
|  | **MAE** | 0.1108266 | 0.0050502 | **1.94E-20*** | 0.0828571 | 0.004742 | **1.94E-20*** | 0.0800582 | 0.0049127 | **1.94E-20*** | 0.0610032 | 0.0049367 | **1.94E-20*** |
| **ICP vs. rSO_2__R** | **MSE** | 0.032775 | 8.20E-05 | **6.07E-20*** | 0.0796218 | 0.0004913 | **7.54E-20*** | 0.0096004 | 3.17E-05 | **6.07E-20*** | 0.0161865 | 4.38E-05 | **6.07E-20*** |
|  | **RMSE** | 0.1338946 | 0.005877 | **6.07E-20*** | 0.107041 | 0.0064551 | **6.07E-20*** | 0.0979817 | 0.0056313 | **6.07E-20*** | 0.0752452 | 0.0048954 | **6.07E-20*** |
|  | **MAE** | 0.1108266 | 0.0048039 | **6.07E-20*** | 0.0828571 | 0.0048961 | **6.07E-20*** | 0.0800582 | 0.0045458 | **6.07E-20*** | 0.0610032 | 0.0048517 | **6.07E-20*** |
| **AMP vs. rSO_2__L** | **MSE** | 0.0170764 | 8.99E-05 | **1.94E-20*** | 0.0690081 | 0.000433 | **1.94E-20*** | 0.0033259 | 3.39E-05 | **1.94E-20*** | 0.0072145 | 4.49E-05 | **1.94E-20*** |
|  | **RMSE** | 0.0858132 | 0.0062568 | **1.94E-20*** | 0.0973542 | 0.0061642 | **1.94E-20*** | 0.0576706 | 0.0058252 | **1.94E-20*** | 0.055903 | 0.0051103 | **1.94E-20*** |
|  | **MAE** | 0.0685939 | 0.0050502 | **1.94E-20*** | 0.0745431 | 0.004742 | **1.94E-20*** | 0.0476023 | 0.0049127 | **1.94E-20*** | 0.0451824 | 0.0049367 | **1.94E-20*** |
| **AMP vs. rSO_2__R** | **MSE** | 0.0170764 | 8.20E-05 | **6.07E-20*** | 0.0690081 | 0.0004913 | **6.07E-20*** | 0.0033259 | 3.17E-05 | **6.07E-20*** | 0.0072145 | 4.38E-05 | **6.07E-20*** |
|  | **RMSE** | 0.0858132 | 0.005877 | **6.07E-20*** | 0.0973542 | 0.0064551 | **6.07E-20*** | 0.0576706 | 0.0056313 | **6.07E-20*** | 0.055903 | 0.0048954 | **6.07E-20*** |
|  | **MAE** | 0.0685939 | 0.0048039 | **6.07E-20*** | 0.0745431 | 0.0048961 | **6.07E-20*** | 0.0476023 | 0.0045458 | **6.07E-20*** | 0.0451824 | 0.0048517 | **6.07E-20*** |
|  | | **5-Minute Window Size and 1-Second Temporal Frequency** | | | | | | | | | | | |
| **ICP vs. rSO_2__L** | **MSE** | 0.048597 | 0.0001901 | **1.94E-20*** | 0.0994543 | 0.0005866 | **1.94E-20*** | 0.0157944 | 5.47E-05 | **1.94E-20*** | 0.024582 | 7.01E-05 | **1.94E-20*** |
|  | **RMSE** | 0.165412 | 0.0098015 | **1.94E-20*** | 0.1332721 | 0.008868 | **1.94E-20*** | 0.1256755 | 0.0073981 | **1.94E-20*** | 0.0935146 | 0.0050131 | **1.94E-20*** |
|  | **MAE** | 0.129095 | 0.0079008 | **1.94E-20*** | 0.0974507 | 0.0073341 | **1.94E-20*** | 0.0989352 | 0.0063651 | **1.94E-20*** | 0.0714959 | 0.0049952 | **1.94E-20*** |
| **ICP vs. rSO_2__R** | **MSE** | 0.048597 | 0.0001882 | **6.07E-20*** | 0.0994543 | 0.0005691 | **6.07E-20*** | 0.0157944 | 4.83E-05 | **6.07E-20*** | 0.024582 | 6.79E-05 | **6.07E-20*** |
|  | **RMSE** | 0.165412 | 0.0094597 | **6.07E-20*** | 0.1332721 | 0.0088982 | **6.07E-20*** | 0.1256755 | 0.0069517 | **6.07E-20*** | 0.0935146 | 0.0051263 | **6.07E-20*** |
|  | **MAE** | 0.129095 | 0.0074948 | **6.07E-20*** | 0.0974507 | 0.0074564 | **6.07E-20*** | 0.0989352 | 0.0060408 | **6.07E-20*** | 0.0714959 | 0.0051271 | **6.07E-20*** |
| **AMP vs. rSO_2__L** | **MSE** | 0.0248299 | 0.0001901 | **1.94E-20*** | 0.0689149 | 0.0005866 | **1.94E-20*** | 0.0061963 | 5.47E-05 | **1.94E-20*** | 0.0130067 | 7.01E-05 | **1.94E-20*** |
|  | **RMSE** | 0.1156271 | 0.0098015 | **1.94E-20*** | 0.1021369 | 0.008868 | **1.94E-20*** | 0.0787164 | 0.0073981 | **1.94E-20*** | 0.0704814 | 0.0050131 | **1.94E-20*** |
|  | **MAE** | 0.0846093 | 0.0079008 | **1.94E-20*** | 0.0756115 | 0.0073341 | **1.94E-20*** | 0.0622849 | 0.0063651 | **1.94E-20*** | 0.0538983 | 0.0049952 | **1.94E-20*** |
| **AMP vs. rSO_2__R** | **MSE** | 0.0248299 | 0.0001882 | **6.07E-20*** | 0.0689149 | 0.0005691 | **6.07E-20*** | 0.0061963 | 4.83E-05 | **6.07E-20*** | 0.0130067 | 6.79E-05 | **6.07E-20*** |
|  | **RMSE** | 0.1156271 | 0.0094597 | **6.07E-20*** | 0.1021369 | 0.0088982 | **6.07E-20*** | 0.0787164 | 0.0069517 | **6.07E-20*** | 0.0704814 | 0.0051263 | **6.07E-20*** |
|  | **MAE** | 0.0846093 | 0.0074948 | **6.07E-20*** | 0.0756115 | 0.0074564 | **6.07E-20*** | 0.0622849 | 0.0060408 | **6.07E-20*** | 0.0538983 | 0.0051271 | **6.07E-20*** |
|  | | **15-Minute Window Size and 1-Second Temporal Frequency** | | | | | | | | | | | |
| **ICP vs. rSO_2__L** | **MSE** | 0.0662026 | 0.0003267 | **1.94E-20*** | 0.1287716 | 0.000748 | **1.94E-20*** | 0.0209722 | 9.97E-05 | **1.94E-20*** | 0.0404737 | 0.0001841 | **1.94E-20*** |
|  | **RMSE** | 0.1892067 | 0.0142691 | **1.94E-20*** | 0.1522684 | 0.0113208 | **1.94E-20*** | 0.144818 | 0.0099852 | **1.94E-20*** | 0.1276363 | 0.0081516 | **1.94E-20*** |
|  | **MAE** | 0.1478375 | 0.0111529 | **1.94E-20*** | 0.114033 | 0.0088931 | **1.94E-20*** | 0.113629 | 0.0081754 | **1.94E-20*** | 0.0937797 | 0.0069551 | **1.94E-20*** |
| **ICP vs. rSO_2__R** | **MSE** | 0.0662026 | 0.0003201 | **6.07E-20*** | 0.1287716 | 0.0006954 | **6.07E-20*** | 0.0209722 | 8.48E-05 | **6.07E-20*** | 0.0404737 | 0.0001832 | **6.07E-20*** |
|  | **RMSE** | 0.1892067 | 0.0133013 | **6.07E-20*** | 0.1522684 | 0.011506 | **6.07E-20*** | 0.144818 | 0.0092072 | **6.07E-20*** | 0.1276363 | 0.0080784 | **6.07E-20*** |
|  | **MAE** | 0.1478375 | 0.0103534 | **6.07E-20*** | 0.114033 | 0.0092011 | **6.07E-20*** | 0.113629 | 0.0075778 | **6.07E-20*** | 0.0937797 | 0.0066011 | **6.07E-20*** |
| **AMP vs. rSO_2__L** | **MSE** | 0.0347007 | 0.0003267 | **1.94E-20*** | 0.062294 | 0.000748 | **1.94E-20*** | 0.0109467 | 9.97E-05 | **1.94E-20*** | 0.024483 | 0.0001841 | **1.94E-20*** |
|  | **RMSE** | 0.1475265 | 0.0142691 | **1.94E-20*** | 0.1059704 | 0.0113208 | **1.94E-20*** | 0.1046212 | 0.0099852 | **1.94E-20*** | 0.0978704 | 0.0081516 | **1.94E-20*** |
|  | **MAE** | 0.1041493 | 0.0111529 | **1.94E-20*** | 0.0746418 | 0.0088931 | **1.94E-20*** | 0.0799461 | 0.0081754 | **1.94E-20*** | 0.0677457 | 0.0069551 | **1.94E-20*** |
| **AMP vs. rSO_2__R** | **MSE** | 0.0347007 | 0.0003201 | **6.07E-20*** | 0.062294 | 0.0006954 | **6.07E-20*** | 0.0109467 | 8.48E-05 | **6.07E-20*** | 0.024483 | 0.0001832 | **6.07E-20*** |
|  | **RMSE** | 0.1475265 | 0.0133013 | **6.07E-20*** | 0.1059704 | 0.011506 | **6.07E-20*** | 0.1046212 | 0.0092072 | **6.07E-20*** | 0.0978704 | 0.0080784 | **6.07E-20*** |
|  | **MAE** | 0.1041493 | 0.0103534 | **6.07E-20*** | 0.0746418 | 0.0092011 | **6.07E-20*** | 0.0799461 | 0.0075778 | **6.07E-20*** | 0.0677457 | 0.0066011 | **6.07E-20*** |
|  | | **30-Minute Window Size and 1-Second Temporal Frequency** | | | | | | | | | | | |
| **ICP vs. rSO_2__L** | **MSE** | 0.0830557 | 0.00046 | **1.94E-20*** | 0.1479495 | 0.0007444 | **2.84E-20*** | 0.0289056 | 0.0001676 | **1.94E-20*** | 0.0602696 | 0.0003656 | **1.94E-20*** |
|  | **RMSE** | 0.2220512 | 0.0176174 | **1.94E-20*** | 0.1590966 | 0.0121601 | **2.84E-20*** | 0.170016 | 0.0129469 | **1.94E-20*** | 0.1482875 | 0.0119722 | **1.94E-20*** |
|  | **MAE** | 0.164438 | 0.0137048 | **1.94E-20*** | 0.111934 | 0.0100349 | **2.84E-20*** | 0.1290547 | 0.0100196 | **1.94E-20*** | 0.1066661 | 0.0089509 | **1.94E-20*** |
| **ICP vs. rSO_2__R** | **MSE** | 0.0830557 | 0.000465 | **6.07E-20*** | 0.1479495 | 0.0008621 | **6.24E-20*** | 0.0289056 | 0.0001632 | **6.07E-20*** | 0.0602696 | 0.00035 | **6.07E-20*** |
|  | **RMSE** | 0.2220512 | 0.0171398 | **6.07E-20*** | 0.1590966 | 0.0127818 | **6.07E-20*** | 0.170016 | 0.0127759 | **6.07E-20*** | 0.1482875 | 0.0119218 | **6.07E-20*** |
|  | **MAE** | 0.164438 | 0.0131311 | **6.07E-20*** | 0.111934 | 0.0099344 | **6.07E-20*** | 0.1290547 | 0.0101312 | **6.07E-20*** | 0.1066661 | 0.008662 | **6.07E-20*** |
| **AMP vs. rSO_2__L** | **MSE** | 0.0421848 | 0.00046 | **1.94E-20*** | 0.0582723 | 0.0007444 | **2.84E-20*** | 0.0176384 | 0.0001676 | **1.94E-20*** | 0.0314939 | 0.0003656 | **1.94E-20*** |
|  | **RMSE** | 0.1745558 | 0.0176174 | **1.94E-20*** | 0.1020299 | 0.0121601 | **2.84E-20*** | 0.1328097 | 0.0129469 | **1.94E-20*** | 0.1020909 | 0.0119722 | **1.94E-20*** |
|  | **MAE** | 0.1175445 | 0.0137048 | **1.94E-20*** | 0.0718131 | 0.0100349 | **2.84E-20*** | 0.096942 | 0.0100196 | **1.94E-20*** | 0.0752509 | 0.0089509 | **1.94E-20*** |
| **AMP vs. rSO_2__R** | **MSE** | 0.0421848 | 0.000465 | **6.07E-20*** | 0.0582723 | 0.0008621 | **6.07E-20*** | 0.0176384 | 0.0001632 | **6.07E-20*** | 0.0314939 | 0.00035 | **6.07E-20*** |
|  | **RMSE** | 0.1745558 | 0.0171398 | **6.07E-20*** | 0.1020299 | 0.0127818 | **6.07E-20*** | 0.1328097 | 0.0127759 | **6.07E-20*** | 0.1020909 | 0.0119218 | **6.07E-20*** |
|  | **MAE** | 0.1175445 | 0.0131311 | **6.07E-20*** | 0.0718131 | 0.0099344 | **6.07E-20*** | 0.096942 | 0.0101312 | **6.07E-20*** | 0.0752509 | 0.008662 | **6.07E-20*** |
|  | | **1-Hour Window Size and 1-Second Temporal Frequency** | | | | | | | | | | | |
| **ICP vs. rSO_2__L** | **MSE** | 0.1056377 | 0.0006641 | **1.94E-20*** | 0.1466504 | 0.0009619 | **6.15E-20*** | 0.0468586 | 0.000343 | **1.94E-20*** | 0.0783823 | 0.0005435 | **1.94E-20*** |
|  | **RMSE** | 0.280061 | 0.0218421 | **1.94E-20*** | 0.1698265 | 0.0140927 | **3.42E-20*** | 0.2164686 | 0.0185204 | **1.94E-20*** | 0.1689739 | 0.0138748 | **1.94E-20*** |
|  | **MAE** | 0.1903812 | 0.0170604 | **1.94E-20*** | 0.1168156 | 0.0111067 | **4.59E-20*** | 0.1641289 | 0.0136767 | **1.94E-20*** | 0.1087015 | 0.0102885 | **1.94E-20*** |
| **ICP vs. rSO_2__R** | **MSE** | 0.1056377 | 0.0006662 | **6.07E-20*** | 0.1466504 | 0.0011057 | **6.58E-20*** | 0.0468586 | 0.0003172 | **6.07E-20*** | 0.0783823 | 0.000549 | **6.07E-20*** |
|  | **RMSE** | 0.280061 | 0.0216247 | **6.07E-20*** | 0.1698265 | 0.0143664 | **6.24E-20*** | 0.2164686 | 0.0178098 | **6.07E-20*** | 0.1689739 | 0.0138885 | **6.07E-20*** |
|  | **MAE** | 0.1903812 | 0.0165936 | **6.07E-20*** | 0.1168156 | 0.0118298 | **6.24E-20*** | 0.1641289 | 0.0128422 | **6.07E-20*** | 0.1087015 | 0.0108722 | **6.07E-20*** |
| **AMP vs. rSO_2__L** | **MSE** | 0.0509163 | 0.0006641 | **1.94E-20*** | 0.0588465 | 0.0009619 | **1.22E-19*** | 0.0287535 | 0.000343 | **1.94E-20*** | 0.0344379 | 0.0005435 | **1.94E-20*** |
|  | **RMSE** | 0.1991537 | 0.0218421 | **1.94E-20*** | 0.0993382 | 0.0140927 | **3.81E-20*** | 0.1685543 | 0.0185204 | **1.94E-20*** | 0.1095155 | 0.0138748 | **1.94E-20*** |
|  | **MAE** | 0.1343246 | 0.0170604 | **1.94E-20*** | 0.0727823 | 0.0111067 | **5.68E-20*** | 0.1194738 | 0.0136767 | **1.94E-20*** | 0.0784842 | 0.0102885 | **1.94E-20*** |
| **AMP vs. rSO_2__R** | **MSE** | 0.0509163 | 0.0006662 | **6.07E-20*** | 0.0588465 | 0.0011057 | **6.24E-20*** | 0.0287535 | 0.0003172 | **6.07E-20*** | 0.0344379 | 0.000549 | **6.07E-20*** |
|  | **RMSE** | 0.1991537 | 0.0216247 | **6.07E-20*** | 0.0993382 | 0.0143664 | **6.24E-20*** | 0.1685543 | 0.0178098 | **6.07E-20*** | 0.1095155 | 0.0138885 | **6.07E-20*** |
|  | **MAE** | 0.1343246 | 0.0165936 | **6.07E-20*** | 0.0727823 | 0.0118298 | **6.24E-20*** | 0.1194738 | 0.0128422 | **6.07E-20*** | 0.0784842 | 0.0108722 | **6.07E-20*** |
| **Physiologic** | **Variance Metric** | **Wilcoxon Sign Rank Test Results** | | | | | | | | | | | |
| **Variable Comparison** |  | **1-Minute Window Size and 10 Second Temporal Frequency** | | | | | | | | | | | |
|  |  | **Mean** | | | **SD** | | | **Median** | | | **IQR** | | |
|  |  | **Median ICP/AMP** | **Median** rSO_2_ | **p-value** | **Median ICP/AMP** | **Median** rSO_2_ | **p-value** | **Median ICP/AMP** | **Median** rSO_2_ | **p-value** | **Median ICP/AMP** | **Median** rSO_2_ | **p-value** |
| **ICP vs. rSO_2__L** | **MSE** | 0.0143357 | 8.22E-05 | **1.94E-20*** | 0.043408 | 0.0010957 | **4.18E-20*** | 0.0038753 | 1.63E-05 | **1.94E-20*** | 0.0096329 | 2.98E-05 | **1.94E-20*** |
|  | **RMSE** | 0.0885076 | 0.0049715 | **1.94E-20*** | 0.0841628 | 0.0069247 | **2.10E-20*** | 0.0622519 | 0.0040355 | **1.94E-20*** | 0.0591369 | 0.0042646 | **1.94E-20*** |
|  | **MAE** | 0.0759816 | 0.0042386 | **1.94E-20*** | 0.0728591 | 0.0059164 | **2.10E-20*** | 0.0520839 | 0.0033419 | **1.94E-20*** | 0.0507551 | 0.0037293 | **1.94E-20*** |
| **ICP vs. rSO_2__R** | **MSE** | 0.0143357 | 9.09E-05 | **6.41E-20*** | 0.043408 | 0.0016787 | **2.22E-19*** | 0.0038753 | 1.35E-05 | **6.07E-20*** | 0.0096329 | 2.81E-05 | **6.07E-20*** |
|  | **RMSE** | 0.0885076 | 0.0046537 | **6.07E-20*** | 0.0841628 | 0.0078626 | **1.26E-19*** | 0.0622519 | 0.0036694 | **6.07E-20*** | 0.0591369 | 0.0042634 | **6.07E-20*** |
|  | **MAE** | 0.0759816 | 0.0039592 | **6.07E-20*** | 0.0728591 | 0.0068877 | **1.79E-19*** | 0.0520839 | 0.0031323 | **6.07E-20*** | 0.0507551 | 0.0036214 | **6.07E-20*** |
| **AMP vs. rSO_2__L** | **MSE** | 0.0136528 | 8.22E-05 | **1.94E-20*** | 0.05427 | 0.0010957 | **2.05E-20*** | 0.0030066 | 1.63E-05 | **2.00E-20*** | 0.0061974 | 2.98E-05 | **1.94E-20*** |
|  | **RMSE** | 0.0773604 | 0.0049715 | **1.94E-20*** | 0.0806452 | 0.0069247 | **1.94E-20*** | 0.0548328 | 0.0040355 | **2.00E-20*** | 0.0521938 | 0.0042646 | **1.94E-20*** |
|  | **MAE** | 0.0652705 | 0.0042386 | **1.94E-20*** | 0.0685629 | 0.0059164 | **1.94E-20*** | 0.0462178 | 0.0033419 | **2.00E-20*** | 0.0446621 | 0.0037293 | **1.94E-20*** |
| **AMP vs. rSO_2__R** | **MSE** | 0.0136528 | 9.09E-05 | **6.58E-20*** | 0.05427 | 0.0016787 | **6.07E-20*** | 0.0030066 | 1.35E-05 | **6.24E-20*** | 0.0061974 | 2.81E-05 | **6.07E-20*** |
|  | **RMSE** | 0.0773604 | 0.0046537 | **6.07E-20*** | 0.0806452 | 0.0078626 | **6.95E-20*** | 0.0548328 | 0.0036694 | **6.24E-20*** | 0.0521938 | 0.0042634 | **6.07E-20*** |
|  | **MAE** | 0.0652705 | 0.0039592 | **6.07E-20*** | 0.0685629 | 0.0068877 | **9.12E-20*** | 0.0462178 | 0.0031323 | **6.24E-20*** | 0.0446621 | 0.0036214 | **6.07E-20*** |
|  | | **5-Minute Window Size and 10 Second Temporal Frequency** | | | | | | | | | | | |
| **ICP vs. rSO_2__L** | **MSE** | 0.0304197 | 0.0002173 | **1.94E-20*** | 0.0762017 | 0.0008286 | **2.97E-20*** | 0.0083377 | 3.82E-05 | **1.94E-20*** | 0.0161419 | 5.72E-05 | **1.94E-20*** |
|  | **RMSE** | 0.121147 | 0.0089824 | **1.94E-20*** | 0.1168079 | 0.0111508 | **2.05E-20*** | 0.0913108 | 0.0061779 | **1.94E-20*** | 0.0780994 | 0.005072 | **1.94E-20*** |
|  | **MAE** | 0.097141 | 0.0072241 | **1.94E-20*** | 0.0953541 | 0.0084419 | **2.05E-20*** | 0.0727691 | 0.0051165 | **1.94E-20*** | 0.0644679 | 0.0046006 | **1.94E-20*** |
| **ICP vs. rSO_2__R** | **MSE** | 0.0304197 | 0.0002221 | **6.07E-20*** | 0.0762017 | 0.0014418 | **9.37E-20*** | 0.0083377 | 3.26E-05 | **6.07E-20*** | 0.0161419 | 5.82E-05 | **6.07E-20*** |
|  | **RMSE** | 0.121147 | 0.0090328 | **6.07E-20*** | 0.1168079 | 0.0116909 | **6.95E-20*** | 0.0913108 | 0.005708 | **6.07E-20*** | 0.0780994 | 0.0049089 | **6.07E-20*** |
|  | **MAE** | 0.097141 | 0.0070521 | **6.07E-20*** | 0.0953541 | 0.0082363 | **7.54E-20*** | 0.0727691 | 0.0049119 | **6.07E-20*** | 0.0644679 | 0.0043875 | **6.07E-20*** |
| **AMP vs. rSO_2__L** | **MSE** | 0.023302 | 0.0002173 | **1.94E-20*** | 0.0553488 | 0.0008286 | **2.16E-20*** | 0.0058995 | 3.82E-05 | **1.94E-20*** | 0.011651 | 5.72E-05 | **1.94E-20*** |
|  | **RMSE** | 0.110572 | 0.0089824 | **1.94E-20*** | 0.0944254 | 0.0111508 | **1.94E-20*** | 0.0768084 | 0.0061779 | **1.94E-20*** | 0.0669337 | 0.005072 | **1.94E-20*** |
|  | **MAE** | 0.0842031 | 0.0072241 | **1.94E-20*** | 0.0684006 | 0.0084419 | **1.94E-20*** | 0.0611725 | 0.0051165 | **1.94E-20*** | 0.0525635 | 0.0046006 | **1.94E-20*** |
| **AMP vs. rSO_2__R** | **MSE** | 0.023302 | 0.0002221 | **6.07E-20*** | 0.0553488 | 0.0014418 | **6.07E-20*** | 0.0058995 | 3.26E-05 | **6.07E-20*** | 0.011651 | 5.82E-05 | **6.07E-20*** |
|  | **RMSE** | 0.110572 | 0.0090328 | **6.07E-20*** | 0.0944254 | 0.0116909 | **6.77E-20*** | 0.0768084 | 0.005708 | **6.07E-20*** | 0.0669337 | 0.0049089 | **6.07E-20*** |
|  | **MAE** | 0.0842031 | 0.0070521 | **6.07E-20*** | 0.0684006 | 0.0082363 | **1.02E-19*** | 0.0611725 | 0.0049119 | **6.07E-20*** | 0.0525635 | 0.0043875 | **6.07E-20*** |
|  | | **15-Minute Window Size and 10 Second Temporal Frequency** | | | | | | | | | | | |
| **ICP vs. rSO_2__L** | **MSE** | 0.0492097 | 0.0003985 | **1.94E-20*** | 0.10067 | 0.0009943 | **2.67E-20*** | 0.0139242 | 7.27E-05 | **1.94E-20*** | 0.0293189 | 0.0001779 | **1.94E-20*** |
|  | **RMSE** | 0.1579728 | 0.0134672 | **1.94E-20*** | 0.1360902 | 0.0133842 | **2.00E-20*** | 0.1180007 | 0.0085277 | **1.94E-20*** | 0.1133327 | 0.0086196 | **1.94E-20*** |
|  | **MAE** | 0.1214951 | 0.0105763 | **1.94E-20*** | 0.1060963 | 0.0100262 | **2.00E-20*** | 0.0936475 | 0.0068878 | **1.94E-20*** | 0.0907798 | 0.0070477 | **1.94E-20*** |
| **ICP vs. rSO_2__R** | **MSE** | 0.0492097 | 0.0004081 | **6.07E-20*** | 0.10067 | 0.0011183 | **8.18E-20*** | 0.0139242 | 6.90E-05 | **6.07E-20*** | 0.0293189 | 0.0001666 | **6.07E-20*** |
|  | **RMSE** | 0.1579728 | 0.013261 | **6.07E-20*** | 0.1360902 | 0.014038 | **6.41E-20*** | 0.1180007 | 0.0083085 | **6.07E-20*** | 0.1133327 | 0.0083313 | **6.07E-20*** |
|  | **MAE** | 0.1214951 | 0.0100544 | **6.07E-20*** | 0.1060963 | 0.0103767 | **6.41E-20*** | 0.0936475 | 0.0066772 | **6.07E-20*** | 0.0907798 | 0.0064549 | **6.07E-20*** |
| **AMP vs. rSO_2__L** | **MSE** | 0.0321526 | 0.0003985 | **1.94E-20*** | 0.0497047 | 0.0009943 | **2.53E-20*** | 0.0101432 | 7.27E-05 | **1.94E-20*** | 0.0238573 | 0.0001779 | **1.94E-20*** |
|  | **RMSE** | 0.1434397 | 0.0134672 | **1.94E-20*** | 0.0975987 | 0.0133842 | **1.94E-20*** | 0.1007048 | 0.0085277 | **1.94E-20*** | 0.0982773 | 0.0086196 | **1.94E-20*** |
|  | **MAE** | 0.1022098 | 0.0105763 | **1.94E-20*** | 0.0699492 | 0.0100262 | **2.00E-20*** | 0.0795905 | 0.0068878 | **1.94E-20*** | 0.067408 | 0.0070477 | **1.94E-20*** |
| **AMP vs. rSO_2__R** | **MSE** | 0.0321526 | 0.0004081 | **6.07E-20*** | 0.0497047 | 0.0011183 | **6.07E-20*** | 0.0101432 | 6.90E-05 | **6.07E-20*** | 0.0238573 | 0.0001666 | **6.07E-20*** |
|  | **RMSE** | 0.1434397 | 0.013261 | **6.07E-20*** | 0.0975987 | 0.014038 | **6.24E-20*** | 0.1007048 | 0.0083085 | **6.07E-20*** | 0.0982773 | 0.0083313 | **6.07E-20*** |
|  | **MAE** | 0.1022098 | 0.0100544 | **6.07E-20*** | 0.0699492 | 0.0103767 | **6.77E-20*** | 0.0795905 | 0.0066772 | **6.07E-20*** | 0.067408 | 0.0064549 | **6.07E-20*** |
|  | | **30-Minute Window Size and 10 Second Temporal Frequency** | | | | | | | | | | | |
| **ICP vs. rSO_2__L** | **MSE** | 0.0645789 | 0.0005283 | **1.94E-20*** | 0.118076 | 0.0010437 | **4.97E-20*** | 0.0199938 | 0.000151 | **1.94E-20*** | 0.0494241 | 0.0003812 | **1.94E-20*** |
|  | **RMSE** | 0.1961808 | 0.0172002 | **1.94E-20*** | 0.1461813 | 0.0148358 | **3.00E-20*** | 0.1413994 | 0.012285 | **1.94E-20*** | 0.1416705 | 0.0126008 | **1.94E-20*** |
|  | **MAE** | 0.1447781 | 0.0132775 | **1.94E-20*** | 0.1032205 | 0.010857 | **3.33E-20*** | 0.1105076 | 0.0097018 | **1.94E-20*** | 0.102265 | 0.0095177 | **1.94E-20*** |
| **ICP vs. rSO_2__R** | **MSE** | 0.0645789 | 0.0005849 | **6.07E-20*** | 0.118076 | 0.0011575 | **7.96E-20*** | 0.0199938 | 0.0001421 | **6.07E-20*** | 0.0494241 | 0.0003591 | **6.07E-20*** |
|  | **RMSE** | 0.1961808 | 0.0173396 | **6.07E-20*** | 0.1461813 | 0.0147594 | **6.95E-20*** | 0.1413994 | 0.0119197 | **6.07E-20*** | 0.1416705 | 0.0130485 | **6.07E-20*** |
|  | **MAE** | 0.1447781 | 0.0133479 | **6.07E-20*** | 0.1032205 | 0.0109056 | **6.95E-20*** | 0.1105076 | 0.0095503 | **6.07E-20*** | 0.102265 | 0.009534 | **6.07E-20*** |
| **AMP vs. rSO_2__L** | **MSE** | 0.0403331 | 0.0005283 | **1.94E-20*** | 0.0553236 | 0.0010437 | **5.99E-20*** | 0.0169988 | 0.000151 | **1.94E-20*** | 0.0306465 | 0.0003812 | **1.94E-20*** |
|  | **RMSE** | 0.1665249 | 0.0172002 | **1.94E-20*** | 0.1011377 | 0.0148358 | **3.00E-20*** | 0.1303796 | 0.012285 | **1.94E-20*** | 0.1018872 | 0.0126008 | **1.94E-20*** |
|  | **MAE** | 0.116875 | 0.0132775 | **1.94E-20*** | 0.0701206 | 0.010857 | **3.33E-20*** | 0.0957549 | 0.0097018 | **1.94E-20*** | 0.0719824 | 0.0095177 | **1.94E-20*** |
| **AMP vs. rSO_2__R** | **MSE** | 0.0403331 | 0.0005849 | **6.07E-20*** | 0.0553236 | 0.0011575 | **7.54E-20*** | 0.0169988 | 0.0001421 | **6.07E-20*** | 0.0306465 | 0.0003591 | **6.07E-20*** |
|  | **RMSE** | 0.1665249 | 0.0173396 | **6.07E-20*** | 0.1011377 | 0.0147594 | **6.41E-20*** | 0.1303796 | 0.0119197 | **6.07E-20*** | 0.1018872 | 0.0130485 | **6.07E-20*** |
|  | **MAE** | 0.116875 | 0.0133479 | **6.07E-20*** | 0.0701206 | 0.0109056 | **6.95E-20*** | 0.0957549 | 0.0095503 | **6.07E-20*** | 0.0719824 | 0.009534 | **6.07E-20*** |
|  | | **1-Hour Window Size and 10 Second Temporal Frequency** | | | | | | | | | | | |
| **ICP vs. rSO_2__L** | **MSE** | 0.0850484 | 0.0007362 | **1.94E-20*** | 0.1277928 | 0.0011584 | **8.67E-20*** | 0.0371375 | 0.0003166 | **1.94E-20*** | 0.0696604 | 0.0006055 | **1.94E-20*** |
|  | **RMSE** | 0.2337659 | 0.0222199 | **1.94E-20*** | 0.1519565 | 0.0155641 | **4.13E-20*** | 0.1927023 | 0.017792 | **1.94E-20*** | 0.1530683 | 0.0154483 | **1.94E-20*** |
|  | **MAE** | 0.1756058 | 0.0169003 | **1.94E-20*** | 0.1153865 | 0.0112071 | **6.48E-20*** | 0.1450785 | 0.0132774 | **1.94E-20*** | 0.11529 | 0.0105448 | **1.94E-20*** |
| **ICP vs. rSO_2__R** | **MSE** | 0.0850484 | 0.0007853 | **6.07E-20*** | 0.1277928 | 0.0014358 | **8.18E-20*** | 0.0371375 | 0.000292 | **6.07E-20*** | 0.0696604 | 0.0005654 | **6.07E-20*** |
|  | **RMSE** | 0.2337659 | 0.022338 | **6.07E-20*** | 0.1519565 | 0.0164743 | **7.34E-20*** | 0.1927023 | 0.0170884 | **6.07E-20*** | 0.1530683 | 0.0152791 | **6.07E-20*** |
|  | **MAE** | 0.1756058 | 0.0171119 | **6.07E-20*** | 0.1153865 | 0.0130447 | **7.34E-20*** | 0.1450785 | 0.012576 | **6.07E-20*** | 0.11529 | 0.0111221 | **6.07E-20*** |
| **AMP vs. rSO_2__L** | **MSE** | 0.0495649 | 0.0007362 | **1.94E-20*** | 0.0517238 | 0.0011584 | **2.06E-19*** | 0.0276685 | 0.0003166 | **1.94E-20*** | 0.033968 | 0.0006055 | **1.94E-20*** |
|  | **RMSE** | 0.1884466 | 0.0222199 | **1.94E-20*** | 0.0975528 | 0.0155641 | **5.68E-20*** | 0.1663386 | 0.017792 | **1.94E-20*** | 0.1063377 | 0.0154483 | **1.94E-20*** |
|  | **MAE** | 0.1345517 | 0.0169003 | **1.94E-20*** | 0.0724144 | 0.0112071 | **1.10E-19*** | 0.1199197 | 0.0132774 | **1.94E-20*** | 0.0772688 | 0.0105448 | **1.94E-20*** |
| **AMP vs. rSO_2__R** | **MSE** | 0.0495649 | 0.0007853 | **6.07E-20*** | 0.0517238 | 0.0014358 | **6.95E-20*** | 0.0276685 | 0.000292 | **6.07E-20*** | 0.033968 | 0.0005654 | **6.07E-20*** |
|  | **RMSE** | 0.1884466 | 0.022338 | **6.07E-20*** | 0.0975528 | 0.0164743 | **6.41E-20*** | 0.1663386 | 0.0170884 | **6.07E-20*** | 0.1063377 | 0.0152791 | **6.07E-20*** |
|  | **MAE** | 0.1345517 | 0.0171119 | **6.07E-20*** | 0.0724144 | 0.0130447 | **6.95E-20*** | 0.1199197 | 0.012576 | **6.07E-20*** | 0.0772688 | 0.0111221 | **6.07E-20*** |
| **Physiologic** | **Variance Metric** | **Wilcoxon Sign Rank Test Results** | | | | | | | | | | | |
| **Variable Comparison** |  | **5-Minute Window Size and 1-Minute Temporal Frequency** | | | | | | | | | | | |
|  |  | **Mean** | | | **SD** | | | **Median** | | | **IQR** | | |
|  |  | **Median ICP/AMP** | **Median** rSO_2_ | **p-value** | **Median ICP/AMP** | **Median** rSO_2_ | **p-value** | **Median ICP/AMP** | **Median** rSO_2_ | **p-value** | **Median ICP/AMP** | **Median** rSO_2_ | **p-value** |
| **ICP vs. rSO_2__L** | **MSE** | 0.0143253 | 0.0001389 | **1.33E-20*** | 0.0465937 | 0.0005971 | **2.13E-20*** | 0.0016726 | 1.38E-05 | **1.33E-20*** | 0.0055124 | 3.56E-05 | **1.33E-20*** |
|  | **RMSE** | 0.0690259 | 0.0064767 | **1.33E-20*** | 0.0881664 | 0.0094647 | **1.33E-20*** | 0.0408974 | 0.0037094 | **1.33E-20*** | 0.0532455 | 0.0046643 | **1.33E-20*** |
|  | **MAE** | 0.0585994 | 0.0055901 | **1.33E-20*** | 0.0774239 | 0.0083339 | **1.37E-20*** | 0.0344796 | 0.0030711 | **1.33E-20*** | 0.0454613 | 0.0040335 | **1.33E-20*** |
| **ICP vs. rSO_2__R** | **MSE** | 0.0143253 | 0.000138 | **8.87E-20*** | 0.0465937 | 0.0006631 | **9.63E-20*** | 0.0016726 | 1.21E-05 | **8.87E-20*** | 0.0055124 | 3.33E-05 | **8.87E-20*** |
|  | **RMSE** | 0.0690259 | 0.0060228 | **8.87E-20*** | 0.0881664 | 0.0095067 | **8.87E-20*** | 0.0408974 | 0.0034811 | **8.87E-20*** | 0.0532455 | 0.0042499 | **8.87E-20*** |
|  | **MAE** | 0.0585994 | 0.0051772 | **8.87E-20*** | 0.0774239 | 0.0083641 | **8.87E-20*** | 0.0344796 | 0.002948 | **8.87E-20*** | 0.0454613 | 0.0037448 | **8.87E-20*** |
| **AMP vs. rSO_2__L** | **MSE** | 0.0084864 | 0.0001389 | **1.33E-20*** | 0.0283239 | 0.0005971 | **3.07E-20*** | 0.0011863 | 1.38E-05 | **1.37E-20*** | 0.0036046 | 3.56E-05 | **1.33E-20*** |
|  | **RMSE** | 0.0595455 | 0.0064767 | **1.33E-20*** | 0.068524 | 0.0094647 | **1.33E-20*** | 0.0344405 | 0.0037094 | **1.37E-20*** | 0.0439665 | 0.0046643 | **1.33E-20*** |
|  | **MAE** | 0.0516184 | 0.0055901 | **1.33E-20*** | 0.0603192 | 0.0083339 | **1.33E-20*** | 0.0291393 | 0.0030711 | **1.37E-20*** | 0.0378985 | 0.0040335 | **1.33E-20*** |
| **AMP vs. rSO_2__R** | **MSE** | 0.0084864 | 0.000138 | **8.87E-20*** | 0.0283239 | 0.0006631 | **1.10E-19*** | 0.0011863 | 1.21E-05 | **9.12E-20*** | 0.0036046 | 3.33E-05 | **8.87E-20*** |
|  | **RMSE** | 0.0595455 | 0.0060228 | **8.87E-20*** | 0.068524 | 0.0095067 | **9.37E-20*** | 0.0344405 | 0.0034811 | **9.12E-20*** | 0.0439665 | 0.0042499 | **8.87E-20*** |
|  | **MAE** | 0.0516184 | 0.0051772 | **8.87E-20*** | 0.0603192 | 0.0083641 | **9.37E-20*** | 0.0291393 | 0.002948 | **9.12E-20*** | 0.0378985 | 0.0037448 | **8.87E-20*** |
|  | | **15-Minute Window Size and 1-Minute Temporal Frequency** | | | | | | | | | | | |
| **ICP vs. rSO_2__L** | **MSE** | 0.0325411 | 0.0003391 | **1.33E-20*** | 0.0835228 | 0.0008215 | **2.19E-20*** | 0.0052902 | 4.65E-05 | **1.33E-20*** | 0.0183168 | 0.0001468 | **1.33E-20*** |
|  | **RMSE** | 0.1264845 | 0.0118425 | **1.33E-20*** | 0.1233043 | 0.0125883 | **1.37E-20*** | 0.0727335 | 0.0068209 | **1.33E-20*** | 0.0961802 | 0.0088641 | **1.33E-20*** |
|  | **MAE** | 0.0981788 | 0.0094988 | **1.33E-20*** | 0.0996919 | 0.0107688 | **1.37E-20*** | 0.0616893 | 0.0056062 | **1.33E-20*** | 0.0792345 | 0.007452 | **1.33E-20*** |
| **ICP vs. rSO_2__R** | **MSE** | 0.0325411 | 0.0003092 | **8.87E-20*** | 0.0835228 | 0.0008955 | **9.12E-20*** | 0.0052902 | 4.38E-05 | **8.87E-20*** | 0.0183168 | 0.0001259 | **8.87E-20*** |
|  | **RMSE** | 0.1264845 | 0.0111098 | **8.87E-20*** | 0.1233043 | 0.0127013 | **8.87E-20*** | 0.0727335 | 0.006615 | **8.87E-20*** | 0.0961802 | 0.0081049 | **8.87E-20*** |
|  | **MAE** | 0.0981788 | 0.0089995 | **8.87E-20*** | 0.0996919 | 0.0105301 | **8.87E-20*** | 0.0616893 | 0.0056274 | **8.87E-20*** | 0.0792345 | 0.0066073 | **8.87E-20*** |
| **AMP vs. rSO_2__L** | **MSE** | 0.0179323 | 0.0003391 | **1.33E-20*** | 0.0343109 | 0.0008215 | **3.50E-20*** | 0.0040338 | 4.65E-05 | **1.33E-20*** | 0.0129114 | 0.0001468 | **1.33E-20*** |
|  | **RMSE** | 0.0963099 | 0.0118425 | **1.33E-20*** | 0.0816416 | 0.0125883 | **1.33E-20*** | 0.0635115 | 0.0068209 | **1.33E-20*** | 0.0757496 | 0.0088641 | **1.33E-20*** |
|  | **MAE** | 0.0770125 | 0.0094988 | **1.33E-20*** | 0.0677471 | 0.0107688 | **1.37E-20*** | 0.0503393 | 0.0056062 | **1.33E-20*** | 0.0604186 | 0.007452 | **1.33E-20*** |
| **AMP vs. rSO_2__R** | **MSE** | 0.0179323 | 0.0003092 | **8.87E-20*** | 0.0343109 | 0.0008955 | **8.87E-20*** | 0.0040338 | 4.38E-05 | **8.87E-20*** | 0.0129114 | 0.0001259 | **8.87E-20*** |
|  | **RMSE** | 0.0963099 | 0.0111098 | **8.87E-20*** | 0.0816416 | 0.0127013 | **8.87E-20*** | 0.0635115 | 0.006615 | **8.87E-20*** | 0.0757496 | 0.0081049 | **8.87E-20*** |
|  | **MAE** | 0.0770125 | 0.0089995 | **8.87E-20*** | 0.0677471 | 0.0105301 | **8.87E-20*** | 0.0503393 | 0.0056274 | **8.87E-20*** | 0.0604186 | 0.0066073 | **8.87E-20*** |
|  | | **30-Minute Window Size and 1-Minute Temporal Frequency** | | | | | | | | | | | |
| **ICP vs. rSO_2__L** | **MSE** | 0.0434384 | 0.0004743 | **1.33E-20*** | 0.0944653 | 0.001026 | **3.86E-20*** | 0.0129284 | 0.0001245 | **1.33E-20*** | 0.0370494 | 0.0003492 | **1.33E-20*** |
|  | **RMSE** | 0.1602744 | 0.0152875 | **1.33E-20*** | 0.1353321 | 0.0139446 | **2.05E-20*** | 0.1137007 | 0.011157 | **1.33E-20*** | 0.1374665 | 0.0128556 | **1.33E-20*** |
|  | **MAE** | 0.1231083 | 0.012195 | **1.33E-20*** | 0.1033574 | 0.01114 | **2.16E-20*** | 0.0886608 | 0.0087295 | **1.33E-20*** | 0.1032432 | 0.0101648 | **1.33E-20*** |
| **ICP vs. rSO_2__R** | **MSE** | 0.0434384 | 0.0005185 | **8.87E-20*** | 0.0944653 | 0.0010358 | **9.63E-20*** | 0.0129284 | 0.0001082 | **8.87E-20*** | 0.0370494 | 0.0003171 | **8.87E-20*** |
|  | **RMSE** | 0.1602744 | 0.0158816 | **8.87E-20*** | 0.1353321 | 0.0141412 | **9.12E-20*** | 0.1137007 | 0.0103997 | **8.87E-20*** | 0.1374665 | 0.0125864 | **8.87E-20*** |
|  | **MAE** | 0.1231083 | 0.0125414 | **8.87E-20*** | 0.1033574 | 0.012045 | **8.87E-20*** | 0.0886608 | 0.0084027 | **8.87E-20*** | 0.1032432 | 0.009486 | **8.87E-20*** |
| **AMP vs. rSO_2__L** | **MSE** | 0.0240675 | 0.0004743 | **1.33E-20*** | 0.0377433 | 0.001026 | **8.69E-20*** | 0.0095797 | 0.0001245 | **1.33E-20*** | 0.0201426 | 0.0003492 | **1.33E-20*** |
|  | **RMSE** | 0.1247951 | 0.0152875 | **1.33E-20*** | 0.0875191 | 0.0139446 | **2.05E-20*** | 0.0977106 | 0.011157 | **1.33E-20*** | 0.0904845 | 0.0128556 | **1.33E-20*** |
|  | **MAE** | 0.0959982 | 0.012195 | **1.33E-20*** | 0.0675759 | 0.01114 | **2.16E-20*** | 0.0737994 | 0.0087295 | **1.33E-20*** | 0.0710167 | 0.0101648 | **1.33E-20*** |
| **AMP vs. rSO_2__R** | **MSE** | 0.0240675 | 0.0005185 | **8.87E-20*** | 0.0377433 | 0.0010358 | **9.63E-20*** | 0.0095797 | 0.0001082 | **8.87E-20*** | 0.0201426 | 0.0003171 | **8.87E-20*** |
|  | **RMSE** | 0.1247951 | 0.0158816 | **8.87E-20*** | 0.0875191 | 0.0141412 | **8.87E-20*** | 0.0977106 | 0.0103997 | **8.87E-20*** | 0.0904845 | 0.0125864 | **8.87E-20*** |
|  | **MAE** | 0.0959982 | 0.0125414 | **8.87E-20*** | 0.0675759 | 0.012045 | **8.87E-20*** | 0.0737994 | 0.0084027 | **8.87E-20*** | 0.0710167 | 0.009486 | **8.87E-20*** |
|  | | **1-Hour Window Size and 1-Minute Temporal Frequency** | | | | | | | | | | | |
| **ICP vs. rSO_2__L** | **MSE** | 0.0654427 | 0.000694 | **1.33E-20*** | 0.1142706 | 0.0011912 | **6.19E-20*** | 0.0274375 | 0.0002774 | **1.33E-20*** | 0.0557918 | 0.0005529 | **1.33E-20*** |
|  | **RMSE** | 0.2092904 | 0.0210129 | **1.33E-20*** | 0.152596 | 0.0157486 | **3.13E-20*** | 0.1656427 | 0.0166557 | **1.33E-20*** | 0.1606029 | 0.0153538 | **1.33E-20*** |
|  | **MAE** | 0.157743 | 0.0163043 | **1.33E-20*** | 0.118314 | 0.0122513 | **4.76E-20*** | 0.1260205 | 0.0126258 | **1.33E-20*** | 0.1163082 | 0.0114228 | **1.33E-20*** |
| **ICP vs. rSO_2__R** | **MSE** | 0.0654427 | 0.0007072 | **8.87E-20*** | 0.1142706 | 0.0014556 | **9.90E-20*** | 0.0274375 | 0.0002378 | **8.87E-20*** | 0.0557918 | 0.0005169 | **8.87E-20*** |
|  | **RMSE** | 0.2092904 | 0.0211176 | **8.87E-20*** | 0.152596 | 0.0172462 | **9.12E-20*** | 0.1656427 | 0.0152526 | **8.87E-20*** | 0.1606029 | 0.0148414 | **8.87E-20*** |
|  | **MAE** | 0.157743 | 0.0165036 | **8.87E-20*** | 0.118314 | 0.0132125 | **9.37E-20*** | 0.1260205 | 0.0116878 | **8.87E-20*** | 0.1163082 | 0.0116284 | **8.87E-20*** |
| **AMP vs. rSO_2__L** | **MSE** | 0.0330391 | 0.000694 | **1.33E-20*** | 0.0401166 | 0.0011912 | **2.10E-19*** | 0.0179576 | 0.0002774 | **1.33E-20*** | 0.02573 | 0.0005529 | **1.33E-20*** |
|  | **RMSE** | 0.1559099 | 0.0210129 | **1.33E-20*** | 0.0875801 | 0.0157486 | **4.40E-20*** | 0.1340056 | 0.0166557 | **1.33E-20*** | 0.0960683 | 0.0153538 | **1.33E-20*** |
|  | **MAE** | 0.1185144 | 0.0163043 | **1.33E-20*** | 0.0707073 | 0.0122513 | **7.05E-20*** | 0.0992302 | 0.0126258 | **1.33E-20*** | 0.0773096 | 0.0114228 | **1.33E-20*** |
| **AMP vs. rSO_2__R** | **MSE** | 0.0330391 | 0.0007072 | **8.87E-20*** | 0.0401166 | 0.0014556 | **9.63E-20*** | 0.0179576 | 0.0002378 | **8.87E-20*** | 0.02573 | 0.0005169 | **8.87E-20*** |
|  | **RMSE** | 0.1559099 | 0.0211176 | **8.87E-20*** | 0.0875801 | 0.0172462 | **9.12E-20*** | 0.1340056 | 0.0152526 | **8.87E-20*** | 0.0960683 | 0.0148414 | **8.87E-20*** |
|  | **MAE** | 0.1185144 | 0.0165036 | **8.87E-20*** | 0.0707073 | 0.0132125 | **9.12E-20*** | 0.0992302 | 0.0116878 | **8.87E-20*** | 0.0773096 | 0.0116284 | **8.87E-20*** |

**Appendix B.**

*ACF* autocorrelation function, *ADF* augmented dickey-fuller, *AIC* Akaike information criterion*, AMP* pulse amplitude of intracranial pressure, *BIC* Bayesian information criterion, *ICP* intracranial pressure, *KPSS* Kwiatkowski-phillips-schmidt-shin*, LL* log-likelihood, *PACF* partial autocorrelation function, *rSO_2__L* left sided regional oxygen saturation, rSO_2_*_R* right sided regional oxygen saturation.

**Table B.1.1**. Population summary of ADF results for non-differenced data.

| **Time Resolution** | **ICP** | | | **AMP** | | | **MAP** | | | rSO_2_**_L** | | | rSO_2_**_R** | | |
| --- | --- | --- | --- | --- | --- | --- | --- | --- | --- | --- | --- | --- | --- | --- | --- |
|  | **Stationary** | **Non- stationary** | **NA** | **Stationary** | **Non- stationary** | **NA** | **Stationary** | **Non- stationary** | **NA** | **Stationary** | **Non- stationary** | **NA** | **Stationary** | **Non- stationary** | **NA** |
| 1-second | 116 | 2 | 0 | 116 | 2 | 0 | 118 | 0 | 0 | 102 | 13 | 0 | 97 | 14 | 0 |
| 10-second | 112 | 6 | 0 | 110 | 8 | 0 | 117 | 1 | 0 | 94 | 21 | 0 | 91 | 20 | 0 |
| 1-minute | 101 | 17 | 0 | 96 | 22 | 0 | 114 | 4 | 0 | 81 | 34 | 0 | 80 | 30 | 0 |
| 5-minute | 87 | 31 | 0 | 80 | 38 | 0 | 106 | 12 | 0 | 64 | 50 | 0 | 63 | 48 | 0 |
| 10-minute | 81 | 37 | 0 | 68 | 50 | 0 | 92 | 26 | 0 | 54 | 60 | 0 | 51 | 60 | 0 |
| 15-minute | 76 | 41 | 0 | 60 | 57 | 0 | 78 | 40 | 0 | 43 | 71 | 0 | 42 | 68 | 0 |
| 30-minute | 61 | 54 | 0 | 40 | 75 | 0 | 59 | 58 | 0 | 25 | 89 | 0 | 26 | 84 | 0 |
| 1-hour | 33 | 80 | 0 | 33 | 81 | 0 | 34 | 81 | 0 | 23 | 89 | 0 | 24 | 82 | 0 |
| 2-hour | 20 | 86 | 0 | 22 | 86 | 0 | 19 | 90 | 0 | 19 | 85 | 0 | 14 | 85 | 0 |
| 3-hour | 14 | 78 | 0 | 15 | 78 | 0 | 10 | 82 | 0 | 10 | 78 | 0 | 10 | 76 | 0 |
| 4-hour | 12 | 74 | 0 | 15 | 71 | 0 | 8 | 79 | 0 | 10 | 71 | 0 | 6 | 76 | 0 |
| 5-hour | 7 | 74 | 0 | 10 | 71 | 0 | 7 | 75 | 0 | 8 | 66 | 0 | 7 | 69 | 0 |
| 6-hour | 7 | 66 | 0 | 7 | 67 | 0 | 7 | 65 | 0 | 7 | 61 | 0 | 5 | 62 | 0 |
| 12-hour | 7 | 108 | 0 | 7 | 108 | 0 | 8 | 107 | 0 | 6 | 106 | 0 | 8 | 99 | 0 |
| 24-hour | 7 | 91 | 0 | 7 | 92 | 0 | 4 | 93 | 0 | 1 | 93 | 0 | 3 | 89 | 0 |

**Table B.1.2**. Population summary of ADF results for first order differenced data.

| **Time Resolution** | **ICP** | | | **AMP** | | | **MAP** | | | rSO_2_**_L** | | | rSO_2_**_R** | | |
| --- | --- | --- | --- | --- | --- | --- | --- | --- | --- | --- | --- | --- | --- | --- | --- |
|  | **Stationary** | **Non- stationary** | **NA** | **Stationary** | **Non- stationary** | **NA** | **Stationary** | **Non- stationary** | **NA** | **Stationary** | **Non- stationary** | **NA** | **Stationary** | **Non- stationary** | **NA** |
| 1-second | 118 | 0 | 0 | 118 | 0 | 0 | 118 | 0 | 0 | 115 | 0 | 0 | 111 | 0 | 0 |
| 10-second | 118 | 0 | 0 | 118 | 0 | 0 | 118 | 0 | 0 | 115 | 0 | 0 | 111 | 0 | 0 |
| 1-minute | 118 | 0 | 0 | 118 | 0 | 0 | 118 | 0 | 0 | 114 | 1 | 0 | 110 | 0 | 0 |
| 5-minute | 114 | 4 | 0 | 116 | 2 | 0 | 118 | 0 | 0 | 111 | 3 | 0 | 109 | 2 | 0 |
| 10-minute | 113 | 4 | 1 | 114 | 3 | 1 | 116 | 2 | 0 | 110 | 4 | 0 | 109 | 2 | 0 |
| 15-minute | 109 | 8 | 0 | 111 | 6 | 0 | 116 | 2 | 0 | 105 | 9 | 0 | 105 | 5 | 0 |
| 30-minute | 102 | 11 | 2 | 104 | 11 | 0 | 109 | 8 | 0 | 96 | 18 | 0 | 97 | 13 | 0 |
| 1-hour | 85 | 28 | 0 | 85 | 29 | 0 | 95 | 20 | 0 | 78 | 34 | 0 | 79 | 27 | 0 |
| 2-hour | 61 | 42 | 3 | 57 | 49 | 2 | 70 | 37 | 2 | 57 | 45 | 2 | 55 | 42 | 2 |
| 3-hour | 52 | 37 | 3 | 49 | 40 | 4 | 54 | 34 | 4 | 37 | 47 | 4 | 35 | 48 | 3 |
| 4-hour | 36 | 46 | 4 | 35 | 47 | 4 | 44 | 39 | 4 | 28 | 48 | 5 | 31 | 49 | 2 |
| 5-hour | 28 | 50 | 3 | 33 | 45 | 3 | 22 | 55 | 5 | 18 | 52 | 4 | 21 | 49 | 6 |
| 6-hour | 25 | 44 | 4 | 24 | 46 | 4 | 20 | 50 | 2 | 19 | 46 | 3 | 23 | 41 | 3 |
| 12-hour | 15 | 82 | 18 | 10 | 88 | 17 | 12 | 85 | 18 | 11 | 83 | 18 | 15 | 77 | 15 |
| 24-hour | 5 | 77 | 16 | 6 | 76 | 17 | 4 | 77 | 16 | 0 | 74 | 20 | 4 | 72 | 16 |

**Table B.1.3**. Population summary of ADF results for second order differenced data.

| **Time Resolution** | **ICP** | | | **AMP** | | | **MAP** | | | rSO_2_**_L** | | | rSO_2_**_R** | | |
| --- | --- | --- | --- | --- | --- | --- | --- | --- | --- | --- | --- | --- | --- | --- | --- |
|  | **Stationary** | **Non- stationary** | **NA** | **Stationary** | **Non- stationary** | **NA** | **Stationary** | **Non- stationary** | **NA** | **Stationary** | **Non- stationary** | **NA** | **Stationary** | **Non- stationary** | **NA** |
| 1-second | 118 | 0 | 0 | 118 | 0 | 0 | 118 | 0 | 0 | 115 | 0 | 0 | 111 | 0 | 0 |
| 10-second | 118 | 0 | 0 | 118 | 0 | 0 | 118 | 0 | 0 | 115 | 0 | 0 | 110 | 1 | 0 |
| 1-minute | 118 | 0 | 0 | 118 | 0 | 0 | 118 | 0 | 0 | 114 | 1 | 0 | 110 | 0 | 0 |
| 5-minute | 118 | 0 | 0 | 118 | 0 | 0 | 118 | 0 | 0 | 114 | 0 | 0 | 110 | 1 | 0 |
| 10-minute | 114 | 3 | 1 | 115 | 2 | 1 | 116 | 2 | 0 | 114 | 0 | 0 | 111 | 0 | 0 |
| 15-minute | 112 | 4 | 1 | 114 | 2 | 1 | 116 | 2 | 0 | 112 | 2 | 0 | 110 | 0 | 0 |
| 30-minute | 111 | 2 | 2 | 113 | 2 | 0 | 115 | 2 | 0 | 108 | 6 | 0 | 105 | 4 | 1 |
| 1-hour | 101 | 10 | 2 | 102 | 12 | 0 | 107 | 8 | 0 | 93 | 18 | 1 | 94 | 12 | 0 |
| 2-hour | 82 | 16 | 8 | 82 | 18 | 8 | 80 | 21 | 8 | 71 | 23 | 10 | 77 | 17 | 5 |
| 3-hour | 66 | 22 | 4 | 70 | 18 | 5 | 72 | 16 | 4 | 61 | 21 | 6 | 61 | 22 | 3 |
| 4-hour | 53 | 29 | 4 | 51 | 31 | 4 | 55 | 28 | 4 | 44 | 30 | 7 | 47 | 31 | 4 |
| 5-hour | 48 | 22 | 11 | 45 | 26 | 10 | 43 | 28 | 11 | 42 | 24 | 8 | 39 | 27 | 10 |
| 6-hour | 42 | 26 | 5 | 38 | 31 | 5 | 43 | 25 | 4 | 29 | 32 | 7 | 35 | 27 | 5 |
| 12-hour | 14 | 74 | 27 | 12 | 76 | 27 | 12 | 75 | 28 | 10 | 72 | 30 | 20 | 62 | 25 |
| 24-hour | 6 | 56 | 36 | 4 | 58 | 37 | 2 | 58 | 37 | 5 | 50 | 39 | 2 | 54 | 36 |

**Table B.1.4**. Population summary of ADF results for third order differenced data.

| **Time Resolution** | **ICP** | | | **AMP** | | | **MAP** | | | rSO_2_**_L** | | | rSO_2_**_R** | | |
| --- | --- | --- | --- | --- | --- | --- | --- | --- | --- | --- | --- | --- | --- | --- | --- |
|  | **Stationary** | **Non- stationary** | **NA** | **Stationary** | **Non- stationary** | **NA** | **Stationary** | **Non- stationary** | **NA** | **Stationary** | **Non- stationary** | **NA** | **Stationary** | **Non- stationary** | **NA** |
| 1-second | 118 | 0 | 0 | 118 | 0 | 0 | 118 | 0 | 0 | 115 | 0 | 0 | 111 | 0 | 0 |
| 10-second | 118 | 0 | 0 | 118 | 0 | 0 | 118 | 0 | 0 | 115 | 0 | 0 | 111 | 0 | 0 |
| 1-minute | 118 | 0 | 0 | 118 | 0 | 0 | 118 | 0 | 0 | 114 | 1 | 0 | 110 | 0 | 0 |
| 5-minute | 118 | 0 | 0 | 117 | 1 | 0 | 118 | 0 | 0 | 114 | 0 | 0 | 111 | 0 | 0 |
| 10-minute | 114 | 3 | 1 | 115 | 2 | 1 | 117 | 1 | 0 | 114 | 0 | 0 | 110 | 1 | 0 |
| 15-minute | 112 | 4 | 1 | 115 | 1 | 1 | 116 | 2 | 0 | 114 | 0 | 0 | 110 | 0 | 0 |
| 30-minute | 111 | 2 | 2 | 115 | 0 | 0 | 115 | 1 | 1 | 111 | 2 | 1 | 105 | 2 | 3 |
| 1-hour | 103 | 8 | 2 | 104 | 10 | 0 | 110 | 5 | 0 | 103 | 7 | 2 | 100 | 6 | 0 |
| 2-hour | 87 | 7 | 12 | 87 | 10 | 11 | 86 | 9 | 14 | 77 | 14 | 13 | 81 | 8 | 10 |
| 3-hour | 74 | 12 | 6 | 73 | 13 | 7 | 75 | 12 | 5 | 69 | 12 | 7 | 71 | 11 | 4 |
| 4-hour | 58 | 23 | 5 | 65 | 16 | 5 | 64 | 16 | 7 | 54 | 19 | 8 | 55 | 19 | 8 |
| 5-hour | 54 | 15 | 12 | 52 | 18 | 11 | 53 | 17 | 12 | 46 | 18 | 10 | 42 | 22 | 12 |
| 6-hour | 42 | 19 | 12 | 46 | 15 | 13 | 49 | 11 | 12 | 41 | 13 | 14 | 38 | 18 | 11 |
| 12-hour | 15 | 66 | 34 | 20 | 61 | 34 | 15 | 66 | 34 | 11 | 62 | 39 | 15 | 61 | 31 |
| 24-hour | 3 | 45 | 50 | 4 | 44 | 51 | 4 | 45 | 48 | 7 | 35 | 52 | 4 | 41 | 47 |

**Table B.1.5**. Population summary of ADF results for fourth order differenced data.

| **Time Resolution** | **ICP** | | | **AMP** | | | **MAP** | | | rSO_2_**_L** | | | rSO_2_**_R** | | |
| --- | --- | --- | --- | --- | --- | --- | --- | --- | --- | --- | --- | --- | --- | --- | --- |
|  | **Stationary** | **Non- stationary** | **NA** | **Stationary** | **Non- stationary** | **NA** | **Stationary** | **Non- stationary** | **NA** | **Stationary** | **Non- stationary** | **NA** | **Stationary** | **Non- stationary** | **NA** |
| 1-second | 118 | 0 | 0 | 118 | 0 | 0 | 118 | 0 | 0 | 115 | 0 | 0 | 111 | 0 | 0 |
| 10-second | 118 | 0 | 0 | 118 | 0 | 0 | 118 | 0 | 0 | 115 | 0 | 0 | 111 | 0 | 0 |
| 1-minute | 118 | 0 | 0 | 118 | 0 | 0 | 118 | 0 | 0 | 115 | 0 | 0 | 110 | 0 | 0 |
| 5-minute | 118 | 0 | 0 | 118 | 0 | 0 | 118 | 0 | 0 | 114 | 0 | 0 | 111 | 0 | 0 |
| 10-minute | 115 | 2 | 1 | 116 | 1 | 1 | 117 | 1 | 0 | 114 | 0 | 0 | 110 | 0 | 1 |
| 15-minute | 112 | 4 | 1 | 115 | 0 | 2 | 117 | 0 | 1 | 114 | 0 | 0 | 109 | 1 | 0 |
| 30-minute | 112 | 1 | 2 | 115 | 0 | 0 | 115 | 1 | 1 | 112 | 1 | 1 | 106 | 1 | 3 |
| 1-hour | 102 | 8 | 3 | 107 | 5 | 2 | 110 | 4 | 1 | 105 | 3 | 4 | 98 | 6 | 2 |
| 2-hour | 88 | 5 | 13 | 87 | 8 | 13 | 86 | 8 | 15 | 80 | 9 | 15 | 80 | 7 | 12 |
| 3-hour | 74 | 10 | 8 | 76 | 8 | 9 | 77 | 7 | 8 | 71 | 9 | 8 | 69 | 13 | 4 |
| 4-hour | 65 | 11 | 10 | 68 | 8 | 10 | 65 | 8 | 14 | 56 | 15 | 10 | 57 | 12 | 13 |
| 5-hour | 57 | 12 | 12 | 54 | 16 | 11 | 56 | 13 | 13 | 46 | 16 | 12 | 45 | 18 | 13 |
| 6-hour | 48 | 9 | 16 | 47 | 10 | 17 | 48 | 8 | 16 | 40 | 10 | 18 | 36 | 15 | 16 |
| 12-hour | 16 | 54 | 45 | 14 | 57 | 44 | 13 | 57 | 45 | 14 | 52 | 46 | 13 | 52 | 42 |
| 24-hour | 1 | 36 | 61 | 3 | 34 | 62 | 0 | 34 | 63 | 2 | 30 | 62 | 1 | 32 | 59 |

**Table B.1.6**. Population summary of KPSS results for non-differenced data.

| **Time Resolution** | **ICP** | | | **AMP** | | | **MAP** | | | rSO_2_**_L** | | | rSO_2_**_R** | | |
| --- | --- | --- | --- | --- | --- | --- | --- | --- | --- | --- | --- | --- | --- | --- | --- |
|  | **Stationary** | **Non- stationary** | **NA** | **Stationary** | **Non- stationary** | **NA** | **Stationary** | **Non- stationary** | **NA** | **Stationary** | **Non- stationary** | **NA** | **Stationary** | **Non- stationary** | **NA** |
| 1-second | 0 | 118 | 0 | 0 | 118 | 0 | 0 | 118 | 0 | 0 | 115 | 0 | 0 | 111 | 0 |
| 10-second | 2 | 116 | 0 | 0 | 118 | 0 | 0 | 118 | 0 | 0 | 115 | 0 | 0 | 111 | 0 |
| 1-minute | 5 | 113 | 0 | 1 | 117 | 0 | 2 | 116 | 0 | 1 | 114 | 0 | 0 | 110 | 0 |
| 5-minute | 17 | 101 | 0 | 7 | 111 | 0 | 17 | 101 | 0 | 1 | 113 | 0 | 7 | 104 | 0 |
| 10-minute | 26 | 92 | 0 | 10 | 108 | 0 | 22 | 96 | 0 | 14 | 100 | 0 | 11 | 100 | 0 |
| 15-minute | 28 | 89 | 0 | 13 | 104 | 0 | 24 | 94 | 0 | 15 | 99 | 0 | 15 | 95 | 0 |
| 30-minute | 39 | 76 | 0 | 23 | 92 | 0 | 36 | 81 | 0 | 21 | 93 | 0 | 25 | 85 | 0 |
| 1-hour | 48 | 65 | 0 | 44 | 70 | 0 | 44 | 71 | 0 | 26 | 86 | 0 | 29 | 77 | 0 |
| 2-hour | 53 | 53 | 0 | 52 | 56 | 0 | 52 | 57 | 0 | 30 | 74 | 0 | 37 | 62 | 0 |
| 3-hour | 53 | 39 | 0 | 50 | 43 | 0 | 43 | 49 | 0 | 20 | 68 | 0 | 28 | 58 | 0 |
| 4-hour | 53 | 33 | 0 | 48 | 38 | 0 | 42 | 45 | 0 | 24 | 57 | 0 | 30 | 52 | 0 |
| 5-hour | 50 | 31 | 0 | 47 | 34 | 0 | 44 | 38 | 0 | 24 | 50 | 0 | 28 | 48 | 0 |
| 6-hour | 45 | 28 | 0 | 43 | 31 | 0 | 38 | 34 | 0 | 25 | 43 | 0 | 23 | 44 | 0 |
| 12-hour | 69 | 46 | 0 | 72 | 43 | 0 | 64 | 51 | 0 | 54 | 58 | 0 | 47 | 60 | 0 |
| 24-hour | 63 | 35 | 0 | 60 | 39 | 0 | 59 | 38 | 0 | 54 | 40 | 0 | 59 | 33 | 0 |

**Table B.1.7**. Population summary of KPSS results for first order differenced data.

| **Time Resolution** | **ICP** | | | **AMP** | | | **MAP** | | | rSO_2_**_L** | | | rSO_2_**_R** | | |
| --- | --- | --- | --- | --- | --- | --- | --- | --- | --- | --- | --- | --- | --- | --- | --- |
|  | **Stationary** | **Non- stationary** | **NA** | **Stationary** | **Non- stationary** | **NA** | **Stationary** | **Non- stationary** | **NA** | **Stationary** | **Non- stationary** | **NA** | **Stationary** | **Non- stationary** | **NA** |
| 1-second | 118 | 0 | 0 | 118 | 0 | 0 | 118 | 0 | 0 | 115 | 0 | 0 | 110 | 1 | 0 |
| 10-second | 118 | 0 | 0 | 118 | 0 | 0 | 118 | 0 | 0 | 111 | 4 | 0 | 109 | 2 | 0 |
| 1-minute | 118 | 0 | 0 | 118 | 0 | 0 | 118 | 0 | 0 | 110 | 5 | 0 | 106 | 4 | 0 |
| 5-minute | 117 | 1 | 0 | 117 | 1 | 0 | 118 | 0 | 0 | 108 | 6 | 0 | 106 | 5 | 0 |
| 10-minute | 117 | 0 | 1 | 116 | 1 | 1 | 118 | 0 | 0 | 107 | 7 | 0 | 108 | 3 | 0 |
| 15-minute | 117 | 0 | 0 | 116 | 1 | 0 | 118 | 0 | 0 | 107 | 7 | 0 | 106 | 4 | 0 |
| 30-minute | 112 | 1 | 2 | 112 | 3 | 0 | 117 | 0 | 0 | 107 | 7 | 0 | 105 | 5 | 0 |
| 1-hour | 111 | 2 | 0 | 109 | 5 | 0 | 112 | 3 | 0 | 107 | 5 | 0 | 97 | 9 | 0 |
| 2-hour | 100 | 3 | 3 | 101 | 5 | 2 | 104 | 3 | 2 | 97 | 5 | 2 | 92 | 5 | 2 |
| 3-hour | 85 | 4 | 3 | 85 | 4 | 4 | 86 | 2 | 4 | 79 | 5 | 4 | 78 | 5 | 3 |
| 4-hour | 77 | 5 | 4 | 78 | 4 | 4 | 80 | 3 | 4 | 70 | 6 | 5 | 74 | 6 | 2 |
| 5-hour | 71 | 7 | 3 | 70 | 8 | 3 | 74 | 3 | 5 | 65 | 5 | 4 | 64 | 6 | 6 |
| 6-hour | 63 | 6 | 4 | 64 | 6 | 4 | 67 | 3 | 2 | 57 | 8 | 3 | 58 | 6 | 3 |
| 12-hour | 74 | 23 | 18 | 79 | 19 | 17 | 80 | 17 | 18 | 73 | 21 | 18 | 71 | 21 | 15 |
| 24-hour | 55 | 27 | 16 | 57 | 25 | 17 | 51 | 30 | 16 | 53 | 21 | 20 | 47 | 29 | 16 |

**Table B.1.8**. Population summary of KPSS results for second order differenced data.

| **Time Resolution** | **ICP** | | | **AMP** | | | **MAP** | | | rSO_2_**_L** | | | rSO_2_**_R** | | |
| --- | --- | --- | --- | --- | --- | --- | --- | --- | --- | --- | --- | --- | --- | --- | --- |
|  | **Stationary** | **Non- stationary** | **NA** | **Stationary** | **Non- stationary** | **NA** | **Stationary** | **Non- stationary** | **NA** | **Stationary** | **Non- stationary** | **NA** | **Stationary** | **Non- stationary** | **NA** |
| 1-second | 118 | 0 | 0 | 118 | 0 | 0 | 118 | 0 | 0 | 115 | 0 | 0 | 111 | 0 | 0 |
| 10-second | 118 | 0 | 0 | 118 | 0 | 0 | 118 | 0 | 0 | 115 | 0 | 0 | 111 | 0 | 0 |
| 1-minute | 118 | 0 | 0 | 118 | 0 | 0 | 118 | 0 | 0 | 115 | 0 | 0 | 110 | 0 | 0 |
| 5-minute | 118 | 0 | 0 | 118 | 0 | 0 | 118 | 0 | 0 | 114 | 0 | 0 | 111 | 0 | 0 |
| 10-minute | 117 | 0 | 1 | 117 | 0 | 1 | 118 | 0 | 0 | 114 | 0 | 0 | 111 | 0 | 0 |
| 15-minute | 115 | 1 | 1 | 116 | 0 | 1 | 118 | 0 | 0 | 114 | 0 | 0 | 110 | 0 | 0 |
| 30-minute | 113 | 0 | 2 | 115 | 0 | 0 | 117 | 0 | 0 | 114 | 0 | 0 | 109 | 0 | 1 |
| 1-hour | 111 | 0 | 2 | 114 | 0 | 0 | 115 | 0 | 0 | 110 | 1 | 1 | 106 | 0 | 0 |
| 2-hour | 97 | 1 | 8 | 100 | 0 | 8 | 100 | 1 | 8 | 93 | 1 | 10 | 94 | 0 | 5 |
| 3-hour | 87 | 1 | 4 | 88 | 0 | 5 | 88 | 0 | 4 | 82 | 0 | 6 | 83 | 0 | 3 |
| 4-hour | 81 | 1 | 4 | 82 | 0 | 4 | 82 | 1 | 4 | 74 | 0 | 7 | 77 | 1 | 4 |
| 5-hour | 69 | 1 | 11 | 71 | 0 | 10 | 71 | 0 | 11 | 65 | 1 | 8 | 65 | 1 | 10 |
| 6-hour | 67 | 1 | 5 | 68 | 1 | 5 | 68 | 0 | 4 | 60 | 1 | 7 | 61 | 1 | 5 |
| 12-hour | 74 | 14 | 27 | 75 | 13 | 27 | 76 | 11 | 28 | 66 | 16 | 30 | 72 | 10 | 25 |
| 24-hour | 41 | 21 | 36 | 42 | 20 | 37 | 39 | 21 | 37 | 41 | 14 | 39 | 41 | 15 | 36 |

**Table B.1.9**. Population summary of KPSS results for third order differenced data.

| **Time Resolution** | **ICP** | | | **AMP** | | | **MAP** | | | rSO_2_**_L** | | | rSO_2_**_R** | | |
| --- | --- | --- | --- | --- | --- | --- | --- | --- | --- | --- | --- | --- | --- | --- | --- |
|  | **Stationary** | **Non- stationary** | **NA** | **Stationary** | **Non- stationary** | **NA** | **Stationary** | **Non- stationary** | **NA** | **Stationary** | **Non- stationary** | **NA** | **Stationary** | **Non- stationary** | **NA** |
| 1-second | 118 | 0 | 0 | 118 | 0 | 0 | 118 | 0 | 0 | 115 | 0 | 0 | 110 | 1 | 0 |
| 10-second | 118 | 0 | 0 | 118 | 0 | 0 | 118 | 0 | 0 | 115 | 0 | 0 | 110 | 1 | 0 |
| 1-minute | 118 | 0 | 0 | 118 | 0 | 0 | 118 | 0 | 0 | 115 | 0 | 0 | 110 | 0 | 0 |
| 5-minute | 118 | 0 | 0 | 118 | 0 | 0 | 118 | 0 | 0 | 114 | 0 | 0 | 111 | 0 | 0 |
| 10-minute | 117 | 0 | 1 | 117 | 0 | 1 | 118 | 0 | 0 | 114 | 0 | 0 | 111 | 0 | 0 |
| 15-minute | 116 | 0 | 1 | 116 | 0 | 1 | 118 | 0 | 0 | 114 | 0 | 0 | 110 | 0 | 0 |
| 30-minute | 113 | 0 | 2 | 115 | 0 | 0 | 116 | 0 | 1 | 113 | 0 | 1 | 107 | 0 | 3 |
| 1-hour | 111 | 0 | 2 | 114 | 0 | 0 | 115 | 0 | 0 | 110 | 0 | 2 | 105 | 1 | 0 |
| 2-hour | 94 | 0 | 12 | 97 | 0 | 11 | 95 | 0 | 14 | 91 | 0 | 13 | 89 | 0 | 10 |
| 3-hour | 86 | 0 | 6 | 86 | 0 | 7 | 87 | 0 | 5 | 81 | 0 | 7 | 81 | 1 | 4 |
| 4-hour | 80 | 1 | 5 | 81 | 0 | 5 | 79 | 1 | 7 | 73 | 0 | 8 | 73 | 1 | 8 |
| 5-hour | 68 | 1 | 12 | 70 | 0 | 11 | 70 | 0 | 12 | 63 | 1 | 10 | 64 | 0 | 12 |
| 6-hour | 60 | 1 | 12 | 60 | 1 | 13 | 60 | 0 | 12 | 54 | 0 | 14 | 56 | 0 | 11 |
| 12-hour | 66 | 15 | 34 | 68 | 13 | 34 | 66 | 15 | 34 | 61 | 12 | 39 | 58 | 18 | 31 |
| 24-hour | 36 | 12 | 50 | 34 | 14 | 51 | 29 | 20 | 48 | 29 | 13 | 52 | 27 | 18 | 47 |

**Table B.1.10**. Population summary of KPSS results for fourth order differenced data.

| **Time Resolution** | **ICP** | | | **AMP** | | | **MAP** | | | rSO_2_**_L** | | | rSO_2_**_R** | | |
| --- | --- | --- | --- | --- | --- | --- | --- | --- | --- | --- | --- | --- | --- | --- | --- |
|  | **Stationary** | **Non- stationary** | **NA** | **Stationary** | **Non- stationary** | **NA** | **Stationary** | **Non- stationary** | **NA** | **Stationary** | **Non- stationary** | **NA** | **Stationary** | **Non- stationary** | **NA** |
| 1-second | 118 | 0 | 0 | 118 | 0 | 0 | 118 | 0 | 0 | 115 | 0 | 0 | 111 | 0 | 0 |
| 10-second | 118 | 0 | 0 | 118 | 0 | 0 | 118 | 0 | 0 | 115 | 0 | 0 | 111 | 0 | 0 |
| 1-minute | 118 | 0 | 0 | 118 | 0 | 0 | 118 | 0 | 0 | 115 | 0 | 0 | 110 | 0 | 0 |
| 5-minute | 118 | 0 | 0 | 118 | 0 | 0 | 118 | 0 | 0 | 114 | 0 | 0 | 111 | 0 | 0 |
| 10-minute | 117 | 0 | 1 | 117 | 0 | 1 | 118 | 0 | 0 | 114 | 0 | 0 | 110 | 0 | 1 |
| 15-minute | 116 | 0 | 1 | 115 | 0 | 2 | 117 | 0 | 1 | 114 | 0 | 0 | 110 | 0 | 0 |
| 30-minute | 113 | 0 | 2 | 115 | 0 | 0 | 116 | 0 | 1 | 113 | 0 | 1 | 107 | 0 | 3 |
| 1-hour | 110 | 0 | 3 | 112 | 0 | 2 | 114 | 0 | 1 | 108 | 0 | 4 | 104 | 0 | 2 |
| 2-hour | 93 | 0 | 13 | 95 | 0 | 13 | 94 | 0 | 15 | 89 | 0 | 15 | 87 | 0 | 12 |
| 3-hour | 84 | 0 | 8 | 83 | 1 | 9 | 83 | 1 | 8 | 80 | 0 | 8 | 82 | 0 | 4 |
| 4-hour | 75 | 1 | 10 | 76 | 0 | 10 | 73 | 0 | 14 | 71 | 0 | 10 | 68 | 1 | 13 |
| 5-hour | 68 | 1 | 12 | 70 | 0 | 11 | 69 | 0 | 13 | 62 | 0 | 12 | 61 | 2 | 13 |
| 6-hour | 55 | 2 | 16 | 56 | 1 | 17 | 55 | 1 | 16 | 50 | 0 | 18 | 49 | 2 | 16 |
| 12-hour | 56 | 14 | 45 | 56 | 15 | 44 | 57 | 13 | 45 | 49 | 17 | 46 | 51 | 14 | 42 |
| 24-hour | 24 | 13 | 61 | 21 | 16 | 62 | 24 | 10 | 63 | 22 | 10 | 62 | 21 | 12 | 59 |

**Table B.2.1.** Patient Example – AIC, while fitting ARIMA models (1-minute resolution)

| **ARIMA Model** | **ICP_AIC** | **AMP_AIC** | **MAP_AIC** | rSO_2_**_L_AIC** | rSO_2_**_R_AIC** |
| --- | --- | --- | --- | --- | --- |
| (1,1,0) | 10286.83 | -2570.47 | 21145.14 | 9660.566 | 5727.25 |
| (1,1,1) | 10168.59 | -2703.89 | 20796.9 | 9609.707 | 5725.783 |
| (1,1,2) | 10167.79 | -2703.03 | 20798.25 | 9611.708 | 5612.029 |
| (1,1,3) | 10168.45 | -2702.59 | 20765.45 | 9607.364 | 5611.321 |
| (1,1,4) | 10170.45 | -2700.66 | 20757 | 9604.006 | 5612.848 |
| (1,1,5) | 10156.3 | -2708.64 | 20758.95 | 9606.009 | 5586.336 |
| (1,1,6) | 10152.5 | -2699.62 | 20810.06 | 9607.991 | 5581.885 |
| (1,1,7) | 10160.49 | -2700.69 | 20757.11 | 9609.648 | 5578.729 |
| (1,1,8) | 10160.12 | -2708.4 | 20758.32 | 9610.822 | 5580.719 |
| (1,1,9) | 10161.91 | -2706.72 | 20760.31 | 9535.323 | 5582.711 |
| (1,1,10) | 10153.14 | -2708.38 | 20760.38 | 9537.247 | 5583.941 |
| (2,1,0) | 10273.56 | -2657.2 | 21007.32 | 9624.967 | 5716.129 |
| (2,1,1) | 10167.64 | -2704.58 | 20798.47 | 9611.698 | 5609.745 |
| (2,1,2) | 10172.57 | -2702.78 | 20800.88 | 9608.941 | 5610.372 |
| (2,1,3) | 10167.29 | -2700.85 | 20757.51 | 9605.756 | 5601.19 |
| (2,1,4) | 10168.91 | -2698.83 | 20759.75 | 9606.009 | 5603.027 |
| (2,1,5) | 10158.23 | -2698.91 | 20756.97 | 9608.012 | 5581.066 |
| (2,1,6) | 10159.27 | -2704.89 | 20758.06 | 9601.803 | 5582.164 |
| (2,1,7) | 10153.73 | -2703.32 | 20760.92 | 9599.676 | 5580.68 |
| (2,1,8) | 10155.7 | -2709.16 | 20761.1 | 9610.033 | 5582.689 |
| (2,1,9) | 10157.97 | -2708.13 | 20759.39 | 9537.341 | 5584.676 |
| (2,1,10) | 10154.74 | -2706.38 | 20761.91 | 9538.718 | 5583.267 |
| (3,1,0) | 10251.77 | -2694.23 | 20933.17 | 9601.657 | 5704.678 |
| (3,1,1) | 10168.32 | -2702.6 | 20783.53 | 9601.931 | 5702.251 |
| (3,1,2) | 10167.49 | -2700.79 | 20757.11 | 9603.74 | 5601.727 |
| (3,1,3) | 10158.84 | -2705.84 | 20761.48 | 9605.589 | 5604.072 |
| (3,1,4) | 10160.75 | -2718.42 | 20756.42 | 9608.009 | 5604.961 |
| (3,1,5) | 10156.45 | -2714.38 | 20758.44 | 9609.993 | 5581.298 |
| (3,1,6) | 10157.19 | -2733.68 | 20761.38 | 9603.483 | 5585.063 |
| (3,1,7) | 10155.67 | -2732.14 | 20763.48 | 9603.399 | 5580.555 |
| (3,1,8) | 10156.29 | -2725.04 | 20762.51 | 9547.862 | 5582.253 |
| (3,1,9) | 10156.64 | -2722.39 | 20762.71 | 9539.038 | 5575.543 |
| (3,1,10) | 10150.27 | -2721.2 | 20764.93 | 9540.61 | 5581.659 |
| (4,1,0) | 10245.23 | -2697.53 | 20899.73 | 9601.807 | 5706.676 |
| (4,1,1) | 10170.32 | -2709.93 | 20770.19 | 9603.813 | 5699.131 |
| (4,1,2) | 10169.32 | -2707.84 | 20777.42 | 9605.832 | 5603.36 |
| (4,1,3) | 10160.73 | -2706.18 | 20756.42 | 9607.515 | 5608.704 |
| (4,1,4) | 10162.72 | -2722.93 | 20758.24 | 9609.101 | 5606.825 |
| (4,1,5) | 10157.94 | -2714.5 | 20762.26 | 9563.555 | 5583.547 |
| (4,1,6) | 10159.55 | -2732.99 | 20758.65 | 9562.817 | 5579.208 |
| (4,1,7) | 10156.97 | -2728.45 | 20759.44 | 9604.855 | 5582.066 |
| (4,1,8) | 10157.86 | -2726.07 | 20760.62 | 9605.429 | 5584.325 |
| (4,1,9) | 10150.85 | -2724.17 | 20762.11 | 9541.524 | 5585.738 |
| (4,1,10) | 10151.55 | -2723.05 | 20764.1 | 9542.551 | 5583.525 |
| (5,1,0) | 10190.27 | -2697.95 | 20854.23 | 9603.805 | 5662.699 |
| (5,1,1) | 10168.38 | -2708.2 | 20767.19 | 9605.8 | 5583.663 |
| (5,1,2) | 10165.11 | -2708.33 | 20771.29 | 9604.118 | 5585.645 |
| (5,1,3) | 10156.91 | -2722.36 | 20758.31 | 9561.752 | 5582.178 |
| (5,1,4) | 10158.86 | -2733.05 | 20764.33 | 9563.3 | 5579.59 |
| (5,1,5) | 10148.11 | -2730.24 | 20762.77 | 9598.78 | 5580.459 |
| (5,1,6) | 10149.05 | -2716.9 | 20759.74 | 9559.856 | 5579.774 |
| (5,1,7) | 10151.2 | -2730.15 | 20761.01 | 9556.098 | 5580.782 |
| (5,1,8) | 10152.04 | -2736.88 | 20762.43 | 9604.639 | 5579.022 |
| (5,1,9) | 10153.96 | -2734.67 | 20764.13 | 9538.711 | 5580.849 |
| (5,1,10) | 10153.41 | -2734.03 | 20766.15 | 9539.059 | 5583.866 |
| (6,1,0) | 10182.16 | -2700.74 | 20848.22 | 9605.707 | 5621.217 |
| (6,1,1) | 10154.32 | -2703.64 | 20753.91 | 9607.759 | 5585.622 |
| (6,1,2) | 10155.62 | -2706.83 | 20755.84 | 9609.783 | 5587.619 |
| (6,1,3) | 10158.67 | -2728.46 | 20758.41 | 9566.376 | 5581.287 |
| (6,1,4) | 10159.28 | -2732.38 | 20759.25 | 9566.224 | 5578.995 |
| (6,1,5) | 10149.05 | -2728.49 | 20758.95 | 9557.716 | 5580.918 |
| (6,1,6) | 10151.05 | -2731.48 | 20761.89 | 9569.688 | 5578.835 |
| (6,1,7) | 10153.01 | -2721.54 | 20762.3 | 9562.973 | 5581.366 |
| (6,1,8) | 10154.47 | -2728.73 | 20764.69 | 9604.203 | 5579.517 |
| (6,1,9) | 10155.82 | -2726.33 | 20766.67 | 9540.979 | 5571.313 |
| (6,1,10) | 10157.8 | -2727.39 | 20768.31 | 9542.393 | 5571.194 |
| (7,1,0) | 10182.1 | -2700.74 | 20845.2 | 9607.636 | 5598.047 |
| (7,1,1) | 10155.41 | -2702.21 | 20755.82 | 9609.275 | 5580.536 |
| (7,1,2) | 10156.04 | -2708.36 | 20757.82 | 9599.679 | 5584.915 |
| (7,1,3) | 10158.57 | -2726.8 | 20759.76 | 9602.306 | 5585.325 |
| (7,1,4) | 10157.41 | -2725.83 | 20758.83 | 9604.269 | 5579.401 |
| (7,1,5) | 10151.07 | -2729.48 | 20760.19 | 9592.023 | 5582.969 |
| (7,1,6) | 10153 | -2726.46 | 20762.12 | 9543.618 | 5580.067 |
| (7,1,7) | 10154.97 | -2725.91 | 20764.09 | 9603.764 | 5580.608 |
| (7,1,8) | 10156.38 | -2723.79 | 20766.65 | 9605.299 | 5581.447 |
| (7,1,9) | 10157.77 | -2722.12 | 20768.25 | 9539.355 | 5575.211 |
| (7,1,10) | 10153.43 | -2722.49 | 20766.97 | 9544.477 | 5585.16 |
| (8,1,0) | 10180.05 | -2705.56 | 20844.53 | 9606.809 | 5598.463 |
| (8,1,1) | 10157.08 | -2711.3 | 20757.78 | 9608.809 | 5570.435 |
| (8,1,2) | 10158.03 | -2710.99 | 20757.41 | 9600.139 | 5573.417 |
| (8,1,3) | 10157.44 | -2722.42 | 20761.42 | 9603.62 | 5571.885 |
| (8,1,4) | 10158.75 | -2723.59 | 20761.58 | 9602.688 | 5568.55 |
| (8,1,5) | 10152.3 | -2728.64 | 20762.49 | 9604.604 | 5573.535 |
| (8,1,6) | 10154.61 | -2732.84 | 20764.09 | 9606.194 | 5577.773 |
| (8,1,7) | 10156.51 | -2723.21 | 20766.11 | 9607.035 | 5577.986 |
| (8,1,8) | 10158.24 | -2721.56 | 20767.53 | 9538.31 | 5580.644 |
| (8,1,9) | 10159.66 | -2724.99 | 20768.49 | 9540.758 | 5576.632 |
| (8,1,10) | 10156.17 | -2729.54 | 20766.28 | 9546.421 | 5577.092 |
| (9,1,0) | 10182 | -2703.6 | 20836.6 | 9608.809 | 5595.667 |
| (9,1,1) | 10159.08 | -2710.25 | 20759.19 | 9549.094 | 5572.306 |
| (9,1,2) | 10159.81 | -2709.01 | 20760.14 | 9549.27 | 5573.779 |
| (9,1,3) | 10156.6 | -2724.13 | 20760.8 | 9544.551 | 5568.498 |
| (9,1,4) | 10152.54 | -2723.35 | 20761.45 | 9604.398 | 5575.667 |
| (9,1,5) | 10154.22 | -2727.05 | 20766.36 | 9553.61 | 5570.235 |
| (9,1,6) | 10156.08 | -2726.64 | 20765.84 | 9553.523 | 5571.765 |
| (9,1,7) | 10158.08 | -2724.15 | 20767.91 | 9548.269 | 5573.762 |
| (9,1,8) | 10159.95 | -2720.7 | 20766.84 | 9541.087 | 5576.123 |
| (9,1,9) | 10154.33 | -2729.06 | 20768.71 | 9540.361 | 5578.404 |
| (9,1,10) | 10155.55 | -2724.52 | 20768.57 | 9537.061 | 5579.855 |
| (10,1,0) | 10167.15 | -2704.64 | 20834.57 | 9606.753 | 5593.665 |
| (10,1,1) | 10157.66 | -2710.08 | 20760.47 | 9548.087 | 5572.387 |
| (10,1,2) | 10158.13 | -2708.08 | 20762 | 9550.176 | 5570.554 |
| (10,1,3) | 10151.38 | -2711.94 | 20762.8 | 9551.721 | 5578.24 |
| (10,1,4) | 10152.27 | -2721.46 | 20764.02 | 9544.013 | 5575.023 |
| (10,1,5) | 10154.24 | -2726.48 | 20765.16 | 9542.905 | 5570.403 |
| (10,1,6) | 10158.06 | -2724.78 | 20767.33 | 9539.025 | 5570.275 |
| (10,1,7) | 10158.49 | -2728.98 | 20767.62 | 9540.208 | 5575.489 |
| (10,1,8) | 10157.01 | -2716.69 | 20766.28 | 9542.777 | 5577.704 |
| (10,1,9) | 10155.5 | -2726.5 | 20768.2 | 9538.655 | 5578.396 |
| (10,1,10) | 10157.86 | -2724.3 | 20770.04 | 9536.26 | 5576.467 |

**Table B.2.2.** Patient Example – BIC, while fitting ARIMA models (1-minute resolution)

| **ARIMA Model** | **ICP_BIC** | **AMP_BIC** | **MAP_BIC** | rSO_2_**_L_BIC** | rSO_2_**_R_BIC** |
| --- | --- | --- | --- | --- | --- |
| (1,1,0) | 10305.65 | -2551.64 | 21164.08 | 9679.328 | 5746.188 |
| (1,1,1) | 10193.68 | -2678.78 | 20822.15 | 9634.723 | 5751.034 |
| (1,1,2) | 10199.16 | -2671.64 | 20829.82 | 9642.978 | 5643.592 |
| (1,1,3) | 10206.09 | -2664.93 | 20803.33 | 9644.888 | 5649.197 |
| (1,1,4) | 10214.36 | -2656.71 | 20801.19 | 9647.784 | 5657.036 |
| (1,1,5) | 10206.48 | -2658.42 | 20809.46 | 9656.041 | 5636.837 |
| (1,1,6) | 10208.96 | -2643.12 | 20866.88 | 9664.277 | 5638.699 |
| (1,1,7) | 10223.22 | -2637.91 | 20820.25 | 9672.188 | 5641.855 |
| (1,1,8) | 10229.12 | -2639.34 | 20827.78 | 9679.616 | 5650.158 |
| (1,1,9) | 10237.19 | -2631.39 | 20836.08 | 9610.372 | 5658.463 |
| (1,1,10) | 10234.7 | -2626.77 | 20842.46 | 9618.549 | 5666.005 |
| (2,1,0) | 10298.65 | -2632.09 | 21032.57 | 9649.983 | 5741.38 |
| (2,1,1) | 10199.01 | -2673.19 | 20830.03 | 9642.968 | 5641.308 |
| (2,1,2) | 10210.21 | -2665.12 | 20838.76 | 9646.465 | 5648.248 |
| (2,1,3) | 10211.21 | -2656.9 | 20801.71 | 9649.534 | 5645.378 |
| (2,1,4) | 10219.1 | -2648.6 | 20810.26 | 9656.041 | 5653.528 |
| (2,1,5) | 10214.69 | -2642.41 | 20813.79 | 9664.298 | 5637.88 |
| (2,1,6) | 10222 | -2642.11 | 20821.19 | 9664.343 | 5645.29 |
| (2,1,7) | 10222.74 | -2634.26 | 20830.38 | 9668.47 | 5650.119 |
| (2,1,8) | 10230.98 | -2633.82 | 20836.86 | 9685.081 | 5658.441 |
| (2,1,9) | 10239.52 | -2626.52 | 20841.47 | 9618.643 | 5666.74 |
| (2,1,10) | 10242.56 | -2618.49 | 20850.31 | 9626.274 | 5671.644 |
| (3,1,0) | 10283.14 | -2662.84 | 20964.74 | 9632.927 | 5736.241 |
| (3,1,1) | 10205.96 | -2664.93 | 20821.41 | 9639.455 | 5740.127 |
| (3,1,2) | 10211.41 | -2656.84 | 20801.3 | 9647.518 | 5645.915 |
| (3,1,3) | 10209.03 | -2655.62 | 20811.99 | 9655.621 | 5654.573 |
| (3,1,4) | 10217.21 | -2661.92 | 20813.24 | 9664.295 | 5661.774 |
| (3,1,5) | 10219.18 | -2651.6 | 20821.58 | 9672.533 | 5644.424 |
| (3,1,6) | 10226.19 | -2664.63 | 20830.83 | 9672.277 | 5654.502 |
| (3,1,7) | 10230.95 | -2656.8 | 20839.25 | 9678.447 | 5656.307 |
| (3,1,8) | 10237.85 | -2643.42 | 20844.59 | 9629.164 | 5664.317 |
| (3,1,9) | 10244.47 | -2634.5 | 20851.11 | 9626.594 | 5663.92 |
| (3,1,10) | 10244.37 | -2627.03 | 20859.64 | 9634.42 | 5676.349 |
| (4,1,0) | 10282.87 | -2659.87 | 20937.61 | 9639.331 | 5744.552 |
| (4,1,1) | 10214.24 | -2665.98 | 20814.39 | 9647.591 | 5743.32 |
| (4,1,2) | 10219.5 | -2657.62 | 20827.93 | 9655.864 | 5653.861 |
| (4,1,3) | 10217.19 | -2649.68 | 20813.25 | 9663.801 | 5665.518 |
| (4,1,4) | 10225.45 | -2660.15 | 20821.38 | 9671.641 | 5669.952 |
| (4,1,5) | 10226.95 | -2645.44 | 20831.71 | 9632.349 | 5652.986 |
| (4,1,6) | 10234.83 | -2657.66 | 20834.42 | 9637.865 | 5654.959 |
| (4,1,7) | 10238.53 | -2646.84 | 20841.52 | 9686.157 | 5664.13 |
| (4,1,8) | 10245.69 | -2638.18 | 20849.02 | 9692.985 | 5672.702 |
| (4,1,9) | 10244.95 | -2630 | 20856.82 | 9635.334 | 5680.428 |
| (4,1,10) | 10251.93 | -2622.61 | 20865.12 | 9642.615 | 5684.527 |
| (5,1,0) | 10234.18 | -2654 | 20898.43 | 9647.583 | 5706.887 |
| (5,1,1) | 10218.57 | -2657.98 | 20817.7 | 9655.832 | 5634.164 |
| (5,1,2) | 10221.57 | -2651.83 | 20828.11 | 9660.404 | 5642.458 |
| (5,1,3) | 10219.65 | -2659.58 | 20821.45 | 9624.292 | 5645.305 |
| (5,1,4) | 10227.87 | -2663.99 | 20833.78 | 9632.094 | 5649.029 |
| (5,1,5) | 10223.39 | -2654.91 | 20838.54 | 9673.828 | 5656.21 |
| (5,1,6) | 10230.6 | -2635.28 | 20841.82 | 9641.158 | 5661.838 |
| (5,1,7) | 10239.03 | -2642.26 | 20849.41 | 9643.654 | 5669.159 |
| (5,1,8) | 10246.14 | -2642.71 | 20857.14 | 9698.449 | 5673.711 |
| (5,1,9) | 10254.33 | -2634.23 | 20865.15 | 9638.776 | 5681.851 |
| (5,1,10) | 10260.05 | -2627.3 | 20873.49 | 9645.378 | 5691.181 |
| (6,1,0) | 10232.35 | -2650.52 | 20898.73 | 9655.739 | 5671.718 |
| (6,1,1) | 10210.78 | -2647.14 | 20810.73 | 9664.045 | 5642.435 |
| (6,1,2) | 10218.36 | -2644.05 | 20818.98 | 9672.323 | 5650.746 |
| (6,1,3) | 10227.68 | -2659.4 | 20827.86 | 9635.17 | 5650.726 |
| (6,1,4) | 10234.56 | -2657.04 | 20835.02 | 9641.272 | 5654.746 |
| (6,1,5) | 10230.61 | -2646.87 | 20841.03 | 9639.018 | 5662.983 |
| (6,1,6) | 10238.87 | -2643.59 | 20850.28 | 9657.244 | 5667.212 |
| (6,1,7) | 10247.11 | -2627.37 | 20857.01 | 9656.783 | 5676.056 |
| (6,1,8) | 10254.84 | -2628.28 | 20865.72 | 9704.267 | 5680.519 |
| (6,1,9) | 10262.46 | -2619.6 | 20874 | 9647.297 | 5678.628 |
| (6,1,10) | 10270.72 | -2614.39 | 20881.96 | 9654.966 | 5684.821 |
| (7,1,0) | 10238.56 | -2644.24 | 20902.03 | 9663.923 | 5654.86 |
| (7,1,1) | 10218.15 | -2639.43 | 20818.96 | 9671.815 | 5643.662 |
| (7,1,2) | 10225.04 | -2639.31 | 20827.27 | 9668.473 | 5654.354 |
| (7,1,3) | 10233.85 | -2651.46 | 20835.53 | 9677.355 | 5661.076 |
| (7,1,4) | 10238.96 | -2644.21 | 20840.91 | 9685.571 | 5661.466 |
| (7,1,5) | 10238.9 | -2641.59 | 20848.59 | 9679.579 | 5671.345 |
| (7,1,6) | 10247.1 | -2632.29 | 20856.82 | 9637.429 | 5674.756 |
| (7,1,7) | 10255.35 | -2625.46 | 20865.11 | 9703.828 | 5681.61 |
| (7,1,8) | 10263.03 | -2617.06 | 20873.99 | 9711.617 | 5688.762 |
| (7,1,9) | 10270.69 | -2609.12 | 20881.9 | 9651.927 | 5688.839 |
| (7,1,10) | 10272.62 | -2603.21 | 20886.94 | 9663.303 | 5705.1 |
| (8,1,0) | 10242.79 | -2642.78 | 20907.67 | 9669.349 | 5661.59 |
| (8,1,1) | 10226.09 | -2642.25 | 20827.23 | 9677.603 | 5639.874 |
| (8,1,2) | 10233.31 | -2635.65 | 20833.17 | 9675.187 | 5649.169 |
| (8,1,3) | 10239 | -2640.81 | 20843.5 | 9684.922 | 5653.949 |
| (8,1,4) | 10246.58 | -2635.7 | 20849.97 | 9690.244 | 5656.926 |
| (8,1,5) | 10246.4 | -2634.47 | 20857.2 | 9698.414 | 5668.224 |
| (8,1,6) | 10254.98 | -2632.4 | 20865.11 | 9706.258 | 5678.775 |
| (8,1,7) | 10263.15 | -2616.49 | 20873.45 | 9713.353 | 5685.3 |
| (8,1,8) | 10271.16 | -2608.56 | 20881.18 | 9650.882 | 5694.272 |
| (8,1,9) | 10278.85 | -2605.71 | 20888.45 | 9659.584 | 5696.572 |
| (8,1,10) | 10281.63 | -2603.98 | 20892.55 | 9671.501 | 5703.344 |
| (9,1,0) | 10251.01 | -2634.54 | 20906.06 | 9677.603 | 5665.106 |
| (9,1,1) | 10234.36 | -2634.91 | 20834.96 | 9624.142 | 5648.057 |
| (9,1,2) | 10241.36 | -2627.39 | 20842.22 | 9630.572 | 5655.843 |
| (9,1,3) | 10244.43 | -2636.24 | 20849.2 | 9632.107 | 5656.875 |
| (9,1,4) | 10246.64 | -2629.18 | 20856.16 | 9698.208 | 5670.356 |
| (9,1,5) | 10254.6 | -2626.6 | 20867.38 | 9653.674 | 5671.237 |
| (9,1,6) | 10262.72 | -2619.91 | 20873.17 | 9659.841 | 5679.08 |
| (9,1,7) | 10271 | -2611.14 | 20881.56 | 9660.841 | 5687.389 |
| (9,1,8) | 10279.14 | -2601.42 | 20886.8 | 9659.913 | 5696.062 |
| (9,1,9) | 10279.79 | -2603.5 | 20894.99 | 9665.441 | 5704.656 |
| (9,1,10) | 10287.29 | -2592.68 | 20901.16 | 9668.395 | 5712.42 |
| (10,1,0) | 10242.43 | -2629.31 | 20910.33 | 9681.801 | 5669.416 |
| (10,1,1) | 10239.21 | -2628.47 | 20842.55 | 9629.389 | 5654.451 |
| (10,1,2) | 10245.96 | -2620.19 | 20850.4 | 9637.732 | 5658.93 |
| (10,1,3) | 10245.48 | -2617.77 | 20857.51 | 9645.531 | 5672.929 |
| (10,1,4) | 10252.65 | -2621.01 | 20865.04 | 9644.078 | 5676.025 |
| (10,1,5) | 10260.89 | -2619.75 | 20872.49 | 9649.223 | 5677.718 |
| (10,1,6) | 10270.98 | -2611.77 | 20880.98 | 9651.597 | 5683.902 |
| (10,1,7) | 10277.68 | -2609.7 | 20887.59 | 9659.034 | 5695.429 |
| (10,1,8) | 10282.47 | -2591.13 | 20892.55 | 9667.857 | 5703.956 |
| (10,1,9) | 10287.24 | -2594.66 | 20900.8 | 9669.989 | 5710.961 |
| (10,1,10) | 10295.88 | -2586.19 | 20908.95 | 9673.848 | 5715.345 |

**Table B.2.3.** Patient Example – LL, while fitting ARIMA models (1-minute resolution)

| **ARIMA Model** | **ICP_LL** | **AMP_LL** | **MAP_LL** | rSO_2_**_L_LL** | rSO_2_**_R_LL** |
| --- | --- | --- | --- | --- | --- |
| (1,1,0) | -5140.42 | 1288.235 | -10569.6 | -4827.28 | -2860.62 |
| (1,1,1) | -5080.29 | 1355.945 | -10394.4 | -4800.85 | -2858.89 |
| (1,1,2) | -5078.9 | 1356.513 | -10394.1 | -4800.85 | -2801.01 |
| (1,1,3) | -5078.22 | 1357.297 | -10376.7 | -4797.68 | -2799.66 |
| (1,1,4) | -5078.22 | 1357.328 | -10371.5 | -4795 | -2799.42 |
| (1,1,5) | -5070.15 | 1362.322 | -10371.5 | -4795 | -2785.17 |
| (1,1,6) | -5067.25 | 1358.811 | -10396 | -4795 | -2781.94 |
| (1,1,7) | -5070.24 | 1360.347 | -10368.6 | -4794.82 | -2779.36 |
| (1,1,8) | -5069.06 | 1365.2 | -10368.2 | -4794.41 | -2779.36 |
| (1,1,9) | -5068.96 | 1365.362 | -10368.2 | -4755.66 | -2779.36 |
| (1,1,10) | -5063.57 | 1367.192 | -10367.2 | -4755.62 | -2778.97 |
| (2,1,0) | -5132.78 | 1332.601 | -10499.7 | -4808.48 | -2854.06 |
| (2,1,1) | -5078.82 | 1357.288 | -10394.2 | -4800.85 | -2799.87 |
| (2,1,2) | -5080.29 | 1357.392 | -10394.4 | -4798.47 | -2799.19 |
| (2,1,3) | -5076.65 | 1357.425 | -10371.8 | -4795.88 | -2793.59 |
| (2,1,4) | -5076.46 | 1357.413 | -10371.9 | -4795 | -2793.51 |
| (2,1,5) | -5070.12 | 1358.456 | -10369.5 | -4795.01 | -2781.53 |
| (2,1,6) | -5069.63 | 1362.445 | -10369 | -4790.9 | -2781.08 |
| (2,1,7) | -5065.86 | 1362.661 | -10369.5 | -4788.84 | -2779.34 |
| (2,1,8) | -5065.85 | 1366.58 | -10368.5 | -4793.02 | -2779.34 |
| (2,1,9) | -5065.98 | 1367.066 | -10366.7 | -4755.67 | -2779.34 |
| (2,1,10) | -5063.37 | 1367.191 | -10367 | -4755.36 | -2777.63 |
| (3,1,0) | -5120.89 | 1352.115 | -10461.6 | -4795.83 | -2847.34 |
| (3,1,1) | -5078.16 | 1357.299 | -10385.8 | -4794.97 | -2845.13 |
| (3,1,2) | -5076.75 | 1357.394 | -10371.6 | -4794.87 | -2793.86 |
| (3,1,3) | -5071.42 | 1360.92 | -10372.7 | -4794.79 | -2794.04 |
| (3,1,4) | -5071.37 | 1368.211 | -10369.2 | -4795 | -2793.48 |
| (3,1,5) | -5068.23 | 1367.191 | -10369.2 | -4795 | -2780.65 |
| (3,1,6) | -5067.59 | 1377.842 | -10369.7 | -4790.74 | -2781.53 |
| (3,1,7) | -5065.83 | 1378.069 | -10369.7 | -4789.7 | -2778.28 |
| (3,1,8) | -5065.15 | 1375.518 | -10368.3 | -4760.93 | -2778.13 |
| (3,1,9) | -5064.32 | 1375.196 | -10367.4 | -4755.52 | -2773.77 |
| (3,1,10) | -5060.13 | 1375.6 | -10367.5 | -4755.31 | -2775.83 |
| (4,1,0) | -5116.62 | 1354.767 | -10443.9 | -4794.9 | -2847.34 |
| (4,1,1) | -5078.16 | 1361.964 | -10378.1 | -4794.91 | -2842.57 |
| (4,1,2) | -5076.66 | 1361.922 | -10380.7 | -4794.92 | -2793.68 |
| (4,1,3) | -5071.37 | 1362.092 | -10369.2 | -4794.76 | -2795.35 |
| (4,1,4) | -5071.36 | 1371.465 | -10369.1 | -4794.55 | -2793.41 |
| (4,1,5) | -5067.97 | 1368.248 | -10370.1 | -4770.78 | -2780.77 |
| (4,1,6) | -5067.78 | 1378.496 | -10367.3 | -4769.41 | -2777.6 |
| (4,1,7) | -5065.49 | 1377.224 | -10366.7 | -4789.43 | -2778.03 |
| (4,1,8) | -5064.93 | 1377.037 | -10366.3 | -4788.71 | -2778.16 |
| (4,1,9) | -5060.42 | 1377.086 | -10366.1 | -4755.76 | -2777.87 |
| (4,1,10) | -5059.78 | 1377.526 | -10366 | -4755.28 | -2775.76 |
| (5,1,0) | -5088.13 | 1355.974 | -10420.1 | -4794.9 | -2824.35 |
| (5,1,1) | -5076.19 | 1362.101 | -10375.6 | -4794.9 | -2783.83 |
| (5,1,2) | -5073.55 | 1363.164 | -10376.6 | -4793.06 | -2783.82 |
| (5,1,3) | -5068.46 | 1371.178 | -10369.2 | -4770.88 | -2781.09 |
| (5,1,4) | -5068.43 | 1377.524 | -10371.2 | -4770.65 | -2778.79 |
| (5,1,5) | -5062.06 | 1377.122 | -10369.4 | -4787.39 | -2778.23 |
| (5,1,6) | -5061.53 | 1371.448 | -10366.9 | -4766.93 | -2776.89 |
| (5,1,7) | -5061.6 | 1379.075 | -10366.5 | -4764.05 | -2776.39 |
| (5,1,8) | -5061.02 | 1383.44 | -10366.2 | -4787.32 | -2774.51 |
| (5,1,9) | -5060.98 | 1383.336 | -10366.1 | -4753.36 | -2774.42 |
| (5,1,10) | -5059.7 | 1384.013 | -10366.1 | -4752.53 | -2774.93 |
| (6,1,0) | -5083.08 | 1358.37 | -10416.1 | -4794.85 | -2802.61 |
| (6,1,1) | -5068.16 | 1360.821 | -10368 | -4794.88 | -2783.81 |
| (6,1,2) | -5067.81 | 1363.413 | -10367.9 | -4794.89 | -2783.81 |
| (6,1,3) | -5068.34 | 1375.229 | -10368.2 | -4772.19 | -2779.64 |
| (6,1,4) | -5067.64 | 1378.189 | -10367.6 | -4771.11 | -2777.5 |
| (6,1,5) | -5061.53 | 1377.243 | -10366.5 | -4765.86 | -2777.46 |
| (6,1,6) | -5061.52 | 1379.74 | -10366.9 | -4770.84 | -2775.42 |
| (6,1,7) | -5061.5 | 1375.77 | -10366.2 | -4766.49 | -2775.68 |
| (6,1,8) | -5061.23 | 1380.363 | -10366.3 | -4786.1 | -2773.76 |
| (6,1,9) | -5060.91 | 1380.164 | -10366.3 | -4753.49 | -2768.66 |
| (6,1,10) | -5060.9 | 1381.694 | -10366.2 | -4753.2 | -2767.6 |
| (7,1,0) | -5082.05 | 1359.37 | -10413.6 | -4794.82 | -2790.02 |
| (7,1,1) | -5067.71 | 1361.106 | -10367.9 | -4794.64 | -2780.27 |
| (7,1,2) | -5067.02 | 1365.182 | -10367.9 | -4788.84 | -2781.46 |
| (7,1,3) | -5067.28 | 1375.398 | -10367.9 | -4789.15 | -2780.66 |
| (7,1,4) | -5065.71 | 1375.913 | -10366.4 | -4789.13 | -2776.7 |
| (7,1,5) | -5061.53 | 1378.739 | -10366.1 | -4782.01 | -2777.48 |
| (7,1,6) | -5061.5 | 1378.231 | -10366.1 | -4756.81 | -2775.03 |
| (7,1,7) | -5061.49 | 1378.953 | -10366 | -4785.88 | -2774.3 |
| (7,1,8) | -5061.19 | 1378.893 | -10366.3 | -4785.65 | -2773.72 |
| (7,1,9) | -5060.88 | 1379.06 | -10366.1 | -4751.68 | -2769.61 |
| (7,1,10) | -5057.71 | 1380.246 | -10364.5 | -4753.24 | -2773.58 |
| (8,1,0) | -5080.03 | 1362.778 | -10412.3 | -4793.4 | -2789.23 |
| (8,1,1) | -5067.54 | 1366.652 | -10367.9 | -4793.4 | -2774.22 |
| (8,1,2) | -5067.01 | 1367.494 | -10366.7 | -4788.07 | -2774.71 |
| (8,1,3) | -5065.72 | 1374.211 | -10367.7 | -4788.81 | -2772.94 |
| (8,1,4) | -5065.37 | 1375.796 | -10366.8 | -4787.34 | -2770.27 |
| (8,1,5) | -5061.15 | 1379.321 | -10366.2 | -4787.3 | -2771.77 |
| (8,1,6) | -5061.3 | 1382.422 | -10366 | -4787.1 | -2772.89 |
| (8,1,7) | -5061.25 | 1378.607 | -10366.1 | -4786.52 | -2771.99 |
| (8,1,8) | -5061.12 | 1378.779 | -10365.8 | -4751.15 | -2772.32 |
| (8,1,9) | -5060.83 | 1381.497 | -10365.2 | -4751.38 | -2769.32 |
| (8,1,10) | -5058.08 | 1384.771 | -10363.1 | -4753.21 | -2768.55 |
| (9,1,0) | -5080 | 1362.798 | -10407.3 | -4793.4 | -2786.83 |
| (9,1,1) | -5067.54 | 1367.123 | -10367.6 | -4762.55 | -2774.15 |
| (9,1,2) | -5066.91 | 1367.503 | -10367.1 | -4761.63 | -2773.89 |
| (9,1,3) | -5064.3 | 1376.064 | -10366.4 | -4758.28 | -2770.25 |
| (9,1,4) | -5061.27 | 1376.676 | -10365.7 | -4787.2 | -2772.83 |
| (9,1,5) | -5061.11 | 1379.524 | -10367.2 | -4760.8 | -2769.12 |
| (9,1,6) | -5061.04 | 1380.318 | -10365.9 | -4759.76 | -2768.88 |
| (9,1,7) | -5061.04 | 1380.073 | -10366 | -4756.13 | -2768.88 |
| (9,1,8) | -5060.97 | 1379.349 | -10364.4 | -4751.54 | -2769.06 |
| (9,1,9) | -5057.16 | 1384.528 | -10364.4 | -4750.18 | -2769.2 |
| (9,1,10) | -5056.77 | 1383.259 | -10363.3 | -4747.53 | -2768.93 |
| (10,1,0) | -5071.58 | 1364.322 | -10405.3 | -4791.38 | -2784.83 |
| (10,1,1) | -5065.83 | 1368.042 | -10367.2 | -4761.04 | -2773.19 |
| (10,1,2) | -5065.07 | 1368.042 | -10367 | -4761.09 | -2771.28 |
| (10,1,3) | -5060.69 | 1370.968 | -10366.4 | -4760.86 | -2774.12 |
| (10,1,4) | -5060.14 | 1376.729 | -10366 | -4756.01 | -2771.51 |
| (10,1,5) | -5060.12 | 1380.24 | -10365.6 | -4754.45 | -2768.2 |
| (10,1,6) | -5061.03 | 1380.389 | -10365.7 | -4751.51 | -2767.14 |
| (10,1,7) | -5060.24 | 1383.491 | -10364.8 | -4751.1 | -2768.74 |
| (10,1,8) | -5058.5 | 1378.343 | -10363.1 | -4751.39 | -2768.85 |
| (10,1,9) | -5056.75 | 1384.249 | -10363.1 | -4748.33 | -2768.2 |
| (10,1,10) | -5056.93 | 1384.15 | -10363 | -4746.13 | -2766.23 |

**Table B.2.4.** Patient example – Choosing ARIMA based on AIC, BIC, and LL at 1-minute Resolution

| **Model Selection Criteria** | **ICP** | **AMP** | **MAP** | rSO_2_**_L** | rSO_2_**_R** |
| --- | --- | --- | --- | --- | --- |
| AIC | (5,1,5) \| 10148.1142 | (5,1,8) \| -2736.8805 | (6,1,1) \| 20753.9066 | (1,1,9) \| 9535.32349 | (9,1,3) \| 5568.49814 |
| BIC | (1,1,1) \| 10193.6795 | (0,1,1) \| -2685.8823 | (1,1,4) \| 20801.1942 | (1,1,9) \| 9610.3716 | (0,1,7) \| 5633.66665 |
| LL | (10,1,9) \| -5056.7497 | (8,1,10) \| 1384.7713 | (10,1,10) \| -10363.021 | (10,1,10) \| -4746.13 | (10,1,10) \| -2766.2336 |

**Figure B.1.** Patient example of ACF and PACF for ICP before and after application of optimal ARIMA orders, primarily based on the AIC, with BIC and LL also recorded and used as secondary measures to confirm model suitability without unnecessary complexity.

**Figure B.2.** Patient example of ACF and PACF for AMP before and after application of optimal ARIMA orders, primarily based on the AIC, with BIC and LL also recorded and used as secondary measures to confirm model suitability without unnecessary complexity.

**rSO_2__L raw PACF (1-min)**

**rSO_2__L raw ACF (1-min)**

**rSO_2__L residual ACF after ARIMA (4,1,1)**

**rSO_2__L residual PACF after ARIMA (4,1,1)**

**Figure B.3.** Patient example of ACF and PACF for rSO_2_ L before and after application of optimal ARIMA orders, primarily based on the AIC, with BIC and LL also recorded and used as secondary measures to confirm model suitability without unnecessary complexity.

**rSO_2__R raw PACF (1-min)**

**rSO_2__R residual ACF after ARIMA (2,1,1)**

**rSO_2__R residual PACF after ARIMA (2,1,1)**

**rSO_2__R raw ACF (1-min)**

**Figure B.3.** Patient example of ACF and PACF for rSO_2_ R before and after application of optimal ARIMA orders, primarily based on the AIC, with BIC and LL also recorded and used as secondary measures to confirm model suitability without unnecessary complexity.

**Appendix C.**

*AMP* pulse amplitude of intracranial pressure, *CT* computed tomography, *GCS* Glasgow Coma Scale*, ICP* intracranial pressure, *IQR* interquartile range*, MAE* mean absolute error, *MSE* mean squared error, *N* number of subjects, *NIRS* near infrared spectroscopy, *RMSE* root mean squared error, *rSO_2__L* left sided regional oxygen saturation, rSO_2_*_R* right sided regional oxygen saturation.

**Table C.** Sub-group analysis for variance metrics across 1-second, 10-second, and 1-minute temporal resolutions. Bolded values denote significant p-values prior to Bonferroni analysis, and an asterisk denotes p-values which remain significant post Bonferroni analysis.

| **Physiologic** | **Variance Metric** | **Wilcoxon Signed Rank Test Results – 1-Second Sampling Frequency** | | | | | | | | | | | | | | | | | | | | |
| --- | --- | --- | --- | --- | --- | --- | --- | --- | --- | --- | --- | --- | --- | --- | --- | --- | --- | --- | --- | --- | --- | --- |
| **Variable Comparison** |  | **Total Population** | | | | | | | | | | | | | | | | | | | | |
|  |  | **5-Minute Window** | | | | **15-Minute Window** | | | **30-Minute Window** | | | | | **1-Hour Window** | | | | | | | | |
|  |  | **Median ICP/AMP** | | **Median** rSO_2_ | **p-value** | **Median ICP/AMP** | **Median** rSO_2_ | **p-value** | **Median ICP/AMP** | **Median** rSO_2_ | | **p-value** | | **Median ICP/AMP** | | | **Median** rSO_2_ | | | **p-value** | | |
| **ICP vs. rSO_2__L** | **MSE** | 1.078143296 | | 0.272822546 | **1.38E-14*** | 1.584417601 | 0.534135033 | **3.83E-12*** | 2.29131997 | 0.854580006 | | **8.03E-10*** | | 3.302973611 | | | 1.577422074 | | | **6.45E-08*** | | |
|  | **RMSE** | 1.038332724 | | 0.522324177 | **7.40E-14*** | 1.258732609 | 0.730845412 | **1.00E-11*** | 1.513705428 | 0.92443381 | | **1.82E-09*** | | 1.80137018 | | | 1.255954637 | | | **1.88E-07*** | | |
|  | **MAE** | 0.835911773 | | 0.467178 | **7.65E-13*** | 0.959503902 | 0.564224741 | **1.49E-10*** | 1.146649532 | 0.714103207 | | **2.09E-08*** | | 1.372322981 | | | 0.962279367 | | | **6.36E-06*** | | |
| **ICP vs. rSO_2__R** | **MSE** | 1.078143296 | | 0.242508944 | **2.04E-15*** | 1.584417601 | 0.45035396 | **5.06E-14*** | 2.29131997 | 0.809903668 | | **3.36E-11*** | | 3.302973611 | | | 1.449822332 | | | **6.95E-09*** | | |
|  | **RMSE** | 1.038332724 | | 0.492451964 | **5.28E-15*** | 1.258732609 | 0.671084168 | **7.75E-14*** | 1.513705428 | 0.899806332 | | **7.46E-11*** | | 1.80137018 | | | 1.204085683 | | | **6.95E-09*** | | |
|  | **MAE** | 0.835911773 | | 0.450683778 | **2.20E-14*** | 0.959503902 | 0.538888284 | **1.12E-12*** | 1.146649532 | 0.69886446 | | **7.84E-10*** | | 1.372322981 | | | 0.90877355 | | | **1.73E-07*** | | |
| **AMP vs. rSO_2__L** | **MSE** | 0.01288498 | | 0.272822546 | **4.93E-17*** | 0.023083298 | 0.534135033 | **4.79E-17*** | 0.037496565 | 0.854580006 | | **9.21E-18*** | | 0.059894637 | | | 1.577422074 | | | **3.09E-18*** | | |
|  | **RMSE** | 0.11349428 | | 0.522324177 | **1.97E-18*** | 0.151882727 | 0.730845412 | **2.08E-18*** | 0.193635323 | 0.92443381 | | **2.54E-18*** | | 0.244722553 | | | 1.255954637 | | | **1.57E-18*** | | |
|  | **MAE** | 0.0906628 | | 0.467178 | **1.86E-18*** | 0.115086862 | 0.564224741 | **1.44E-18*** | 0.14034556 | 0.714103207 | | **1.48E-18*** | | 0.16876757 | | | 0.962279367 | | | **1.21E-18*** | | |
| **AMP vs. rSO_2__R** | **MSE** | 0.01288498 | | 0.242508944 | **7.58E-16*** | 0.023083298 | 0.45035396 | **1.01E-15*** | 0.037496565 | 0.809903668 | | **2.85E-16*** | | 0.059894637 | | | 1.449822332 | | | **6.22E-17*** | | |
|  | **RMSE** | 0.11349428 | | 0.492451964 | **5.36E-17*** | 0.151882727 | 0.671084168 | **8.11E-17*** | 0.193635323 | 0.899806332 | | **5.05E-17*** | | 0.244722553 | | | 1.204085683 | | | **1.67E-17*** | | |
|  | **MAE** | 0.0906628 | | 0.450683778 | **5.86E-17*** | 0.115086862 | 0.538888284 | **5.05E-17*** | 0.14034556 | 0.69886446 | | **1.72E-17*** | | 0.16876757 | | | 0.90877355 | | | **1.35E-17*** | | |
|  | | **ICP Monitor Same Side as NIRS** | | | | | | | | | | | | | | | | | | | | |
| **ICP vs. rSO_2__L** | **MSE** | 1.08418 | | 0.27132 | **2.83e-15*** | 1.59231 | 0.45537 | **1.01e-12*** | 2.27927 | 0.80503 | | **1.89e-09*** | | 3.32339 | | | 1.51139 | | | **2.95e-07*** | | |
|  | **RMSE** | 1.04124 | | 0.52088 | **2.83e-15*** | 1.26187 | 0.67468 | **9.44e-13*** | 1.50972 | 0.89723 | | **1.89e-09*** | | 1.81177 | | | 1.22868 | | | **3.20e-07*** | | |
|  | **MAE** | 0.85652 | | 0.46296 | **1.30e-13*** | 0.96304 | 0.5374 | **3.39e-11*** | 1.13685 | 0.69441 | | **2.82e-08*** | | 1.37442 | | | 0.94047 | | | **7.28e-06*** | | |
| **ICP vs. rSO_2__R** | **MSE** | 1.08418 | | 0.24251 | **1.02e-15*** | 1.59231 | 0.45035 | **2.24e-13*** | 2.27927 | 0.8099 | | **1.33e-09*** | | 3.32339 | | | 1.44982 | | | **1.24e-07*** | | |
|  | **RMSE** | 1.04124 | | 0.49245 | **1.02e-15*** | 1.26187 | 0.67108 | **2.24e-13*** | 1.50972 | 0.89981 | | **1.31e-09*** | | 1.81177 | | | 1.20409 | | | **1.34e-07*** | | |
|  | **MAE** | 0.85652 | | 0.45068 | **1.21e-14*** | 0.96304 | 0.53889 | **6.56e-12*** | 1.13685 | 0.69886 | | **1.80e-08*** | | 1.37442 | | | 0.90877 | | | **1.94e-06*** | | |
| **AMP vs. rSO_2__L** | **MSE** | 0.01334 | | 0.27132 | **2.15e-32*** | 0.02199 | 0.45537 | **2.21e-32*** | 0.03697 | 0.80503 | | **6.14e-33*** | | 0.05927 | | | 1.51139 | | | **8.56e-34*** | | |
|  | **RMSE** | 0.1155 | | 0.52088 | **2.15e-32*** | 0.14828 | 0.67468 | **2.21e-32*** | 0.19227 | 0.89723 | | **4.70e-33*** | | 0.24346 | | | 1.22868 | | | **8.56e-34*** | | |
|  | **MAE** | 0.09155 | | 0.46296 | **8.75e-32*** | 0.1148 | 0.5374 | **1.08e-32*** | 0.13974 | 0.69441 | | **5.37e-33*** | | 0.16648 | | | 0.94047 | | | **9.81e-34*** | | |
| **AMP vs. rSO_2__R** | **MSE** | 0.01334 | | 0.24251 | **1.56e-30*** | 0.02199 | 0.45035 | **9.29e-31*** | 0.03697 | 0.8099 | | **1.30e-31*** | | 0.05927 | | | 1.44982 | | | **1.21e-33*** | | |
|  | **RMSE** | 0.1155 | | 0.49245 | **1.56e-30*** | 0.14828 | 0.67108 | **9.29e-31*** | 0.19227 | 0.89981 | | **1.11e-31*** | | 0.24346 | | | 1.20409 | | | **1.17e-33*** | | |
|  | **MAE** | 0.09155 | | 0.45068 | **1.20e-29*** | 0.1148 | 0.53889 | **3.44e-31*** | 0.13974 | 0.69886 | | **1.20e-31*** | | 0.16648 | | | 0.90877 | | | **5.68e-33*** | | |
|  | | **ICP Monitor Opposite Side as NIRS** | | | | | | | | | | | | | | | | | | | | |
| **ICP vs. rSO_2__L** | **MSE** | 1.07211 | | 0.27282 | **1.63e-14*** | 1.57653 | 0.53414 | **5.27e-12*** | 2.30337 | 0.85458 | | **6.25e-09*** | | 3.32339 | | | 1.57742 | | | **9.71e-07*** | | |
|  | **RMSE** | 1.03543 | | 0.52232 | **1.63e-14*** | 1.2556 | 0.73085 | **4.96e-12*** | 1.51769 | 0.92443 | | **6.25e-09*** | | 1.81177 | | | 1.25595 | | | **1.06e-06*** | | |
|  | **MAE** | 0.81531 | | 0.46718 | **7.60e-13*** | 0.95597 | 0.56422 | **1.51e-10*** | 1.13685 | 0.7141 | | **1.12e-07*** | | 1.37345 | | | 0.96228 | | | **3.17e-05*** | | |
| **ICP vs. rSO_2__R** | **MSE** | 1.07211 | | 0.2424 | **7.90e-15*** | 1.57653 | 0.45583 | **1.17e-12*** | 2.30337 | 0.8141 | | **2.81e-09*** | | 3.32339 | | | 1.46766 | | | **2.16e-07*** | | |
|  | **RMSE** | 1.03543 | | 0.49234 | **7.90e-15*** | 1.2556 | 0.67511 | **1.17e-12*** | 1.51769 | 0.9022 | | **2.78e-09*** | | 1.81177 | | | 1.21144 | | | **2.32e-07*** | | |
|  | **MAE** | 0.81531 | | 0.44836 | **7.39e-14*** | 0.95597 | 0.53916 | **3.28e-11*** | 1.13685 | 0.69924 | | **4.10e-08*** | | 1.37345 | | | 0.91258 | | | **4.66e-06*** | | |
| **AMP vs. rSO_2__L** | **MSE** | 0.01243 | | 0.27282 | **6.92e-34*** | 0.02199 | 0.53414 | **6.24e-34*** | 0.03697 | 0.85458 | | **1.68e-34*** | | 0.05927 | | | 1.57742 | | | **2.34e-35*** | | |
|  | **RMSE** | 0.11149 | | 0.52232 | **6.92e-34*** | 0.14828 | 0.73085 | **6.24e-34*** | 0.19227 | 0.92443 | | **1.30e-34*** | | 0.24346 | | | 1.25595 | | | **2.34e-35*** | | |
|  | **MAE** | 0.08978 | | 0.46718 | **2.80e-33*** | 0.1148 | 0.56422 | **3.29e-34*** | 0.13974 | 0.7141 | | **1.48e-34*** | | 0.16648 | | | 0.96228 | | | **2.53e-35*** | | |
| **AMP vs. rSO_2__R** | **MSE** | 0.01243 | | 0.2424 | **6.27e-31*** | 0.02199 | 0.45583 | **3.39e-31*** | 0.03697 | 0.8141 | | **4.97e-32*** | | 0.05927 | | | 1.46766 | | | **4.53e-34*** | | |
|  | **RMSE** | 0.11149 | | 0.49234 | **6.27e-31*** | 0.14828 | 0.67511 | **3.39e-31*** | 0.19227 | 0.9022 | | **4.25e-32*** | | 0.24346 | | | 1.21144 | | | **4.41e-34*** | | |
|  | **MAE** | 0.08978 | | 0.44836 | **5.32e-30*** | 0.1148 | 0.53916 | **1.41e-31*** | 0.13974 | 0.69924 | | **4.59e-32*** | | 0.16648 | | | 0.91258 | | | **2.21e-33*** | | |
|  | | **Primary Decompressive Craniectomy** | | | | | | | | | | | | | | | | | | | | |
| **ICP vs. rSO_2__L** | **MSE** | 1.07211 | | 0.24103 | **0.00104** | 1.39022 | 0.3972 | **0.00214** | 2.08787 | 0.67981 | | **0.00974** | | 2.9946 | | | 1.64255 | | | **0.02639** | | |
|  | **RMSE** | 1.03543 | | 0.49095 | **0.00104** | 1.17907 | 0.63024 | **0.00214** | 1.44494 | 0.8245 | | **0.00974** | | 1.72368 | | | 1.27967 | | | **0.02883** | | |
|  | **MAE** | 0.81361 | | 0.45674 | **0.00202** | 0.8797 | 0.51829 | **0.00795** | 1.10992 | 0.67319 | | **0.01831** | | 1.32181 | | | 0.96567 | | | **0.04239** | | |
| **ICP vs. rSO_2__R** | **MSE** | 1.07211 | | 0.24332 | **1.85e-04*** | 1.39022 | 0.44418 | **5.09e-04*** | 2.08787 | 0.68095 | | **0.00370** | | 2.9946 | | | 1.09332 | | | **0.01211** | | |
|  | **RMSE** | 1.03543 | | 0.49328 | **1.85e-04*** | 1.17907 | 0.66647 | **5.09e-04*** | 1.44494 | 0.8252 | | **0.00370** | | 1.72368 | | | 1.04562 | | | **0.01211** | | |
|  | **MAE** | 0.81361 | | 0.45893 | **2.25e-04*** | 0.8797 | 0.53785 | **0.00147** | 1.10992 | 0.64299 | | **0.00790** | | 1.32181 | | | 0.82139 | | | **0.04725** | | |
| **AMP vs. rSO_2__L** | **MSE** | 0.01036 | | 0.24103 | **1.14e-09*** | 0.01693 | 0.3972 | **1.75e-09*** | 0.04578 | 0.67981 | | **1.95e-09*** | | 0.05238 | | | 1.64255 | | | **1.75e-09*** | | |
|  | **RMSE** | 0.10177 | | 0.49095 | **1.14e-09*** | 0.13011 | 0.63024 | **1.75e-09*** | 0.21397 | 0.8245 | | **1.95e-09*** | | 0.22887 | | | 1.27967 | | | **1.75e-09*** | | |
|  | **MAE** | 0.08026 | | 0.45674 | **5.58e-09*** | 0.09835 | 0.51829 | **1.14e-09*** | 0.12687 | 0.67319 | | **1.75e-09*** | | 0.15779 | | | 0.96567 | | | **1.41e-09*** | | |
| **AMP vs. rSO_2__R** | **MSE** | 0.01036 | | 0.24332 | **7.03e-10*** | 0.01693 | 0.44418 | **1.06e-09*** | 0.04578 | 0.68095 | | **1.18e-09*** | | 0.05238 | | | 1.09332 | | | **9.58e-10*** | | |
|  | **RMSE** | 0.10177 | | 0.49328 | **7.03e-10*** | 0.13011 | 0.66647 | **1.06e-09*** | 0.21397 | 0.8252 | | **1.18e-09*** | | 0.22887 | | | 1.04562 | | | **9.58e-10*** | | |
|  | **MAE** | 0.08026 | | 0.45893 | **1.77e-09*** | 0.09835 | 0.53785 | **4.17e-10*** | 0.12687 | 0.64299 | | **1.06e-09*** | | 0.15779 | | | 0.82139 | | | **6.34e-10*** | | |
|  | | **Secondary Decompressive Craniectomy** | | | | | | | | | | | | | | | | | | | | |
| **ICP vs. rSO_2__L** | **MSE** | 1.02117 | | 0.29595 | **0.04091** | 1.76895 | 0.59668 | **0.02984** | 4.15015 | 1.04787 | | **0.02984** | | 6.77757 | | | 1.56012 | | | 0.07364 | | |
|  | **RMSE** | 1.01053 | | 0.54402 | **0.04091** | 1.33002 | 0.77245 | **0.02984** | 2.03675 | 1.02365 | | **0.02984** | | 2.60318 | | | 1.24902 | | | 0.07364 | | |
|  | **MAE** | 0.81531 | | 0.48569 | 0.15986 | 1.05314 | 0.60884 | **0.04091** | 1.38444 | 0.79746 | | **0.02984** | | 1.93538 | | | 0.93338 | | | 0.05528 | | |
| **ICP vs. rSO_2__R** | **MSE** | 1.02117 | | 0.34669 | 0.14385 | 1.76895 | 0.76572 | 0.07404 | 4.15015 | 1.6161 | | 0.07404 | | 6.77757 | | | 2.58144 | | | 0.19388 | | |
|  | **RMSE** | 1.01053 | | 0.58881 | 0.14385 | 1.33002 | 0.87502 | 0.07404 | 2.03675 | 1.27126 | | 0.07404 | | 2.60318 | | | 1.59965 | | | 0.19388 | | |
|  | **MAE** | 0.81531 | | 0.49903 | 0.19388 | 1.05314 | 0.69857 | 0.19388 | 1.38444 | 1.01695 | | 0.07404 | | 1.93538 | | | 1.23344 | | | 0.19388 | | |
| **AMP vs. rSO_2__L** | **MSE** | 0.01505 | | 0.29595 | **0.02984** | 0.03162 | 0.59668 | **0.02984** | 0.05148 | 1.04787 | | **0.00494** | | 0.08131 | | | 1.56012 | | | **0.00217** | | |
|  | **RMSE** | 0.12268 | | 0.54402 | **0.02984** | 0.17773 | 0.77245 | **0.02984** | 0.22689 | 1.02365 | | **0.00494** | | 0.28511 | | | 1.24902 | | | **0.00217** | | |
|  | **MAE** | 0.09245 | | 0.48569 | **0.02984** | 0.12668 | 0.60884 | **0.02984** | 0.16988 | 0.79746 | | **0.01519** | | 0.20146 | | | 0.93338 | | | **0.00729** | | |
| **AMP vs. rSO_2__R** | **MSE** | 0.01505 | | 0.34669 | 0.05132 | 0.03162 | 0.76572 | **0.02299** | 0.05148 | 1.6161 | | **0.01485** | | 0.08131 | | | 2.58144 | | | **0.00937** | | |
|  | **RMSE** | 0.12268 | | 0.58881 | 0.05132 | 0.17773 | 0.87502 | **0.02299** | 0.22689 | 1.27126 | | **0.01485** | | 0.28511 | | | 1.59965 | | | **0.00937** | | |
|  | **MAE** | 0.09245 | | 0.49903 | 0.05132 | 0.12668 | 0.69857 | **0.02299** | 0.16988 | 1.01695 | | **0.01485** | | 0.20146 | | | 1.23344 | | | **0.00937** | | |
|  | | **No Decompressive Craniectomy** | | | | | | | | | | | | | | | | | | | | |
| **ICP vs. rSO_2__L** | **MSE** | 1.11966 | | 0.27183 | **2.45e-11*** | 1.58442 | 0.53414 | **2.53e-09*** | 2.29132 | 0.87239 | | **6.81e-07*** | | 3.46495 | | | 1.57742 | | | **4.21e-05*** | | |
|  | **RMSE** | 1.05801 | | 0.52137 | **2.45e-11*** | 1.25873 | 0.73085 | **2.53e-09*** | 1.51371 | 0.93398 | | **6.81e-07*** | | 1.86015 | | | 1.25595 | | | **4.28e-05*** | | |
|  | **MAE** | 0.85776 | | 0.46718 | **2.59e-10*** | 0.9595 | 0.58494 | **1.02e-08*** | 1.13401 | 0.7141 | | **5.25e-06*** | | 1.37619 | | | 0.96228 | | | **6.74e-04*** | | |
| **ICP vs. rSO_2__R** | **MSE** | 1.11966 | | 0.24228 | **1.84e-11*** | 1.58442 | 0.48423 | **1.38e-09*** | 2.29132 | 0.89005 | | **4.87e-07*** | | 3.46495 | | | 1.65291 | | | **1.84e-05*** | | |
|  | **RMSE** | 1.05801 | | 0.49222 | **1.84e-11*** | 1.25873 | 0.69586 | **1.38e-09*** | 1.51371 | 0.94342 | | **4.87e-07*** | | 1.86015 | | | 1.28566 | | | **1.87e-05*** | | |
|  | **MAE** | 0.85776 | | 0.44603 | **9.09e-11*** | 0.9595 | 0.53944 | **4.75e-09*** | 1.13401 | 0.73444 | | **2.76e-06*** | | 1.37619 | | | 0.97779 | | | **7.77e-05*** | | |
| **AMP vs. rSO_2__L** | **MSE** | 0.01288 | | 0.27183 | **1.27e-24*** | 0.0218 | 0.53414 | **8.11e-25*** | 0.03646 | 0.87239 | | **4.30e-25*** | | 0.05989 | | | 1.57742 | | | **1.39e-25*** | | |
|  | **RMSE** | 0.11349 | | 0.52137 | **1.27e-24*** | 0.14765 | 0.73085 | **8.11e-25*** | 0.19093 | 0.93398 | | **3.19e-25*** | | 0.24472 | | | 1.25595 | | | **1.39e-25*** | | |
|  | **MAE** | 0.09066 | | 0.46718 | **1.05e-24*** | 0.11509 | 0.58494 | **9.07e-25*** | 0.14035 | 0.7141 | | **3.70e-25*** | | 0.16877 | | | 0.96228 | | | **2.03e-25*** | | |
| **AMP vs. rSO_2__R** | **MSE** | 0.01288 | | 0.24228 | **4.80e-22*** | 0.0218 | 0.48423 | **2.55e-22*** | 0.03646 | 0.89005 | | **1.07e-22*** | | 0.05989 | | | 1.65291 | | | **2.37e-24*** | | |
|  | **RMSE** | 0.11349 | | 0.49222 | **4.80e-22*** | 0.14765 | 0.69586 | **2.55e-22*** | 0.19093 | 0.94342 | | **9.56e-23*** | | 0.24472 | | | 1.28566 | | | **2.37e-24*** | | |
|  | **MAE** | 0.09066 | | 0.44603 | **1.09e-21*** | 0.11509 | 0.53944 | **3.07e-22*** | 0.14035 | 0.73444 | | **1.16e-22*** | | 0.16877 | | | 0.97779 | | | **1.21e-23*** | | |
|  | | **Glasgow Coma Scale of 8 or less** | | | | | | | | | | | | | | | | | | | | |
| **ICP vs. rSO_2__L** | **MSE** | 1.19513 | | 0.29972 | **9.40e-12*** | 1.72766 | 0.61825 | **2.27e-09*** | 2.59213 | 1.01696 | | **3.38e-07*** | | 4.06907 | | | 1.62932 | | | **4.35e-05*** | | |
|  | **RMSE** | 1.09321 | | 0.54745 | **9.40e-12*** | 1.3144 | 0.78627 | **2.13e-09*** | 1.60997 | 1.0084 | | **3.38e-07*** | | 2.01718 | | | 1.27466 | | | **4.42e-05*** | | |
|  | **MAE** | 0.86621 | | 0.47871 | **6.37e-11*** | 1.02082 | 0.6205 | **1.27e-08*** | 1.2005 | 0.7775 | | **2.47e-06*** | | 1.40462 | | | 0.99403 | | | **6.49e-04*** | | |
| **ICP vs. rSO_2__R** | **MSE** | 1.19513 | | 0.2639 | **2.53e-12*** | 1.72766 | 0.49909 | **6.80e-10*** | 2.59213 | 0.97423 | | **2.89e-07*** | | 4.06907 | | | 2.01092 | | | **3.28e-05*** | | |
|  | **RMSE** | 1.09321 | | 0.51371 | **2.53e-12*** | 1.3144 | 0.70646 | **6.80e-10*** | 1.60997 | 0.98702 | | **2.89e-07*** | | 2.01718 | | | 1.41806 | | | **3.28e-05*** | | |
|  | **MAE** | 0.86621 | | 0.46483 | **1.31e-11*** | 1.02082 | 0.56762 | **3.52e-09*** | 1.2005 | 0.75787 | | **1.54e-06*** | | 1.40462 | | | 1.01614 | | | **1.89e-04*** | | |
| **AMP vs. rSO_2__L** | **MSE** | 0.01288 | | 0.29972 | **5.15e-25*** | 0.02464 | 0.61825 | **4.80e-25*** | 0.03907 | 1.01696 | | **2.52e-25*** | | 0.05645 | | | 1.62932 | | | **4.60e-26*** | | |
|  | **RMSE** | 0.11349 | | 0.54745 | **5.15e-25*** | 0.15696 | 0.78627 | **4.80e-25*** | 0.19758 | 1.0084 | | **2.52e-25*** | | 0.23743 | | | 1.27466 | | | **4.60e-26*** | | |
|  | **MAE** | 0.09066 | | 0.47871 | **1.49e-24*** | 0.11711 | 0.6205 | **4.47e-25*** | 0.14417 | 0.7775 | | **2.18e-25*** | | 0.17548 | | | 0.99403 | | | **3.08e-26*** | | |
| **AMP vs. rSO_2__R** | **MSE** | 0.01288 | | 0.2639 | **4.34e-23*** | 0.02464 | 0.49909 | **2.95e-23*** | 0.03907 | 0.97423 | | **9.57e-24*** | | 0.05645 | | | 2.01092 | | | **9.67e-26*** | | |
|  | **RMSE** | 0.11349 | | 0.51371 | **4.34e-23*** | 0.15696 | 0.70646 | **2.95e-23*** | 0.19758 | 0.98702 | | **9.57e-24*** | | 0.23743 | | | 1.41806 | | | **9.67e-26*** | | |
|  | **MAE** | 0.09066 | | 0.46483 | **2.13e-22*** | 0.11711 | 0.56762 | **2.01e-23*** | 0.14417 | 0.75787 | | **9.57e-24*** | | 0.17548 | | | 1.01614 | | | **3.37e-25*** | | |
|  | | **Glasgow Coma Scale of 9 - 12** | | | | | | | | | | | | | | | | | | | | |
| **ICP vs. rSO_2__L** | **MSE** | 0.4475 | | 0.21719 | **0.04241** | 1.21993 | 0.34848 | **0.02460** | 1.50417 | 0.6984 | | 0.08472 | | 3.42058 | | | 1.29275 | | | **0.04241** | | |
|  | **RMSE** | 0.66895 | | 0.46604 | **0.04241** | 1.10451 | 0.59026 | **0.02460** | 1.22645 | 0.83563 | | 0.08472 | | 1.72368 | | | 1.13674 | | | 0.05212 | | |
|  | **MAE** | 0.53369 | | 0.40734 | 0.06362 | 0.68432 | 0.49094 | 0.08472 | 0.96078 | 0.64866 | | 0.11116 | | 1.23017 | | | 0.84004 | | | 0.14372 | | |
| **ICP vs. rSO_2__R** | **MSE** | 0.4475 | | 0.22582 | **0.04649** | 1.21993 | 0.4012 | **0.03440** | 1.50417 | 0.6018 | | 0.10574 | | 3.42058 | | | 1.0487 | | | **0.04211** | | |
|  | **RMSE** | 0.66895 | | 0.47521 | **0.04649** | 1.10451 | 0.63341 | **0.03440** | 1.22645 | 0.7756 | | 0.09709 | | 1.72368 | | | 1.02406 | | | **0.04211** | | |
|  | **MAE** | 0.53369 | | 0.42545 | 0.06800 | 0.68432 | 0.50723 | 0.10574 | 0.96078 | 0.61231 | | 0.17107 | | 1.23017 | | | 0.81874 | | | 0.15846 | | |
| **AMP vs. rSO_2__L** | **MSE** | 0.01036 | | 0.21719 | **1.12e-04*** | 0.01503 | 0.34848 | **9.38e-05*** | 0.04884 | 0.6984 | | **1.72e-05*** | | 0.06777 | | | 1.29275 | | | **9.43e-06*** | | |
|  | **RMSE** | 0.10177 | | 0.46604 | **1.12e-04*** | 0.12261 | 0.59026 | **9.38e-05*** | 0.22099 | 0.83563 | | **1.72e-05*** | | 0.26032 | | | 1.13674 | | | **9.43e-06*** | | |
|  | **MAE** | 0.08026 | | 0.40734 | **1.34e-04*** | 0.09547 | 0.49094 | **6.51e-05*** | 0.10928 | 0.64866 | | **2.54e-05*** | | 0.15779 | | | 0.84004 | | | **1.15e-05*** | | |
| **AMP vs. rSO_2__R** | **MSE** | 0.01036 | | 0.22582 | **1.15e-04*** | 0.01503 | 0.4012 | **5.74e-05*** | 0.04884 | 0.6018 | | **1.61e-05*** | | 0.06777 | | | 1.0487 | | | **7.48e-06*** | | |
|  | **RMSE** | 0.10177 | | 0.47521 | **1.15e-04*** | 0.12261 | 0.63341 | **5.74e-05*** | 0.22099 | 0.7756 | | **1.61e-05*** | | 0.26032 | | | 1.02406 | | | **7.48e-06*** | | |
|  | **MAE** | 0.08026 | | 0.42545 | **1.36e-04*** | 0.09547 | 0.50723 | **3.36e-05*** | 0.10928 | 0.61231 | | **1.94e-05*** | | 0.15779 | | | 0.81874 | | | **9.07e-06*** | | |
|  | | **Pupil Response Category – Bilateral Reactive** | | | | | | | | | | | | | | | | | | | | |
| **ICP vs. rSO_2__L** | **MSE** | 1.15514 | | 0.27282 | **1.02e-10*** | 1.73584 | 0.4732 | **1.04e-09*** | 2.46188 | 0.81586 | | **1.45e-07*** | | 3.8373 | | | 1.56012 | | | **1.25e-06*** | | |
|  | **RMSE** | 1.07478 | | 0.52232 | **1.02e-10*** | 1.31751 | 0.68789 | **1.02e-09*** | 1.56902 | 0.90325 | | **1.45e-07*** | | 1.9589 | | | 1.24902 | | | **1.40e-06*** | | |
|  | **MAE** | 0.859 | | 0.46927 | **1.29e-09*** | 1.02353 | 0.53766 | **1.50e-08*** | 1.19913 | 0.69157 | | **6.55e-07*** | | 1.39406 | | | 0.96419 | | | **1.94e-05*** | | |
| **ICP vs. rSO_2__R** | **MSE** | 1.15514 | | 0.24292 | **1.99e-10*** | 1.73584 | 0.44727 | **2.88e-09*** | 2.46188 | 0.80671 | | **4.26e-07*** | | 3.8373 | | | 1.50888 | | | **4.15e-06*** | | |
|  | **RMSE** | 1.07478 | | 0.49287 | **1.99e-10*** | 1.31751 | 0.66878 | **2.88e-09*** | 1.56902 | 0.8981 | | **4.16e-07*** | | 1.9589 | | | 1.22832 | | | **4.59e-06*** | | |
|  | **MAE** | 0.859 | | 0.45172 | **1.01e-09*** | 1.02353 | 0.53786 | **1.72e-08*** | 1.19913 | 0.68861 | | **1.70e-06*** | | 1.39406 | | | 0.91939 | | | **2.04e-05*** | | |
| **AMP vs. rSO_2__L** | **MSE** | 0.01505 | | 0.27282 | **2.99e-23*** | 0.03132 | 0.4732 | **2.28e-23*** | 0.04884 | 0.81586 | | **5.63e-24*** | | 0.07884 | | | 1.56012 | | | **1.60e-24*** | | |
|  | **RMSE** | 0.12268 | | 0.52232 | **2.99e-23*** | 0.17699 | 0.68789 | **2.28e-23*** | 0.22099 | 0.90325 | | **5.63e-24*** | | 0.27929 | | | 1.24902 | | | **1.60e-24*** | | |
|  | **MAE** | 0.09634 | | 0.46927 | **4.06e-23*** | 0.12668 | 0.53766 | **2.03e-23*** | 0.15318 | 0.69157 | | **7.69e-24*** | | 0.19986 | | | 0.96419 | | | **1.66e-24*** | | |
| **AMP vs. rSO_2__R** | **MSE** | 0.01505 | | 0.24292 | **1.82e-21*** | 0.03132 | 0.44727 | **8.03e-22*** | 0.04884 | 0.80671 | | **2.42e-22*** | | 0.07884 | | | 1.50888 | | | **6.33e-23*** | | |
|  | **RMSE** | 0.12268 | | 0.49287 | **1.82e-21*** | 0.17699 | 0.66878 | **8.03e-22*** | 0.22099 | 0.8981 | | **2.42e-22*** | | 0.27929 | | | 1.22832 | | | **6.07e-23*** | | |
|  | **MAE** | 0.09634 | | 0.45172 | **4.44e-21*** | 0.12668 | 0.53786 | **7.40e-22*** | 0.15318 | 0.68861 | | **2.52e-22*** | | 0.19986 | | | 0.91939 | | | **6.89e-23*** | | |
|  | | **Pupil Response Category – Bilateral Unreactive** | | | | | | | | | | | | | | | | | | | | |
| **ICP vs. rSO_2__L** | **MSE** | 0.77852 | | 0.24103 | **0.02563** | 1.31573 | 0.53552 | 0.05482 | 2.2162 | 0.85458 | | 0.13230 | | 2.77105 | | | 1.57742 | | | 0.37762 | | |
|  | **RMSE** | 0.88226 | | 0.49095 | **0.02563** | 1.14705 | 0.7318 | 0.05482 | 1.48869 | 0.92443 | | 0.13230 | | 1.66451 | | | 1.25595 | | | 0.37762 | | |
|  | **MAE** | 0.68654 | | 0.45674 | **0.02927** | 0.84465 | 0.56068 | 0.06929 | 1.04747 | 0.71671 | | 0.16112 | | 1.07401 | | | 0.9757 | | | 0.53342 | | |
| **ICP vs. rSO_2__R** | **MSE** | 0.77852 | | 0.28778 | **0.01543** | 1.31573 | 0.49046 | 0.06362 | 2.2162 | 0.70621 | | 0.23006 | | 2.77105 | | | 1.29112 | | | 0.44501 | | |
|  | **RMSE** | 0.88226 | | 0.53607 | **0.01543** | 1.14705 | 0.70031 | 0.06362 | 1.48869 | 0.84023 | | 0.23006 | | 1.66451 | | | 1.1352 | | | 0.44501 | | |
|  | **MAE** | 0.68654 | | 0.46945 | **0.02195** | 0.84465 | 0.56762 | 0.05212 | 1.04747 | 0.6708 | | 0.32611 | | 1.07401 | | | 0.90292 | | | 0.71066 | | |
| **AMP vs. rSO_2__L** | **MSE** | 0.00672 | | 0.24103 | **4.13e-05*** | 0.01322 | 0.53552 | **2.08e-05*** | 0.02459 | 0.85458 | | **2.08e-05*** | | 0.03694 | | | 1.57742 | | | **2.08e-05*** | | |
|  | **RMSE** | 0.08198 | | 0.49095 | **4.13e-05*** | 0.11497 | 0.7318 | **2.08e-05*** | 0.15681 | 0.92443 | | **2.08e-05*** | | 0.19219 | | | 1.25595 | | | **2.08e-05*** | | |
|  | **MAE** | 0.06228 | | 0.45674 | **8.00e-05*** | 0.08178 | 0.56068 | **2.08e-05*** | 0.1185 | 0.71671 | | **2.08e-05*** | | 0.13389 | | | 0.9757 | | | **2.08e-05*** | | |
| **AMP vs. rSO_2__R** | **MSE** | 0.00672 | | 0.28778 | **1.15e-05*** | 0.01322 | 0.49046 | **5.10e-06*** | 0.02459 | 0.70621 | | **5.10e-06*** | | 0.03694 | | | 1.29112 | | | **5.10e-06*** | | |
|  | **RMSE** | 0.08198 | | 0.53607 | **1.15e-05*** | 0.11497 | 0.70031 | **5.10e-06*** | 0.15681 | 0.84023 | | **5.10e-06*** | | 0.19219 | | | 1.1352 | | | **5.10e-06*** | | |
|  | **MAE** | 0.06228 | | 0.46945 | **2.09e-05*** | 0.08178 | 0.56762 | **5.10e-06*** | 0.1185 | 0.6708 | | **5.10e-06*** | | 0.13389 | | | 0.90292 | | | **5.10e-06*** | | |
|  | | **Pupil Response Category – Unilateral Unreactive** | | | | | | | | | | | | | | | | | | | | |
| **ICP vs. rSO_2__L** | **MSE** | 0.98344 | | 0.28713 | **2.99e-04*** | 1.35381 | 0.62706 | **0.00334** | 1.91444 | 0.91517 | | **0.02685** | | 2.98036 | | | 1.60136 | | | 0.11124 | | |
|  | **RMSE** | 0.98625 | | 0.53585 | **2.99e-04*** | 1.16265 | 0.79156 | **0.00334** | 1.38348 | 0.95665 | | **0.02685** | | 1.72519 | | | 1.26545 | | | 0.11124 | | |
|  | **MAE** | 0.77505 | | 0.47263 | **7.92e-04*** | 0.90925 | 0.61023 | **0.00625** | 1.0525 | 0.75838 | | 0.06387 | | 1.26729 | | | 0.95167 | | | 0.21793 | | |
| **ICP vs. rSO_2__R** | **MSE** | 0.98344 | | 0.23225 | **9.76e-05*** | 1.35381 | 0.36448 | **3.49e-04*** | 1.91444 | 0.89502 | | **0.00240** | | 2.98036 | | | 1.35895 | | | **0.01127** | | |
|  | **RMSE** | 0.98625 | | 0.48192 | **9.76e-05*** | 1.16265 | 0.60373 | **3.49e-04*** | 1.38348 | 0.94606 | | **0.00240** | | 1.72519 | | | 1.16574 | | | **0.01127** | | |
|  | **MAE** | 0.77505 | | 0.42696 | **1.87e-04*** | 0.90925 | 0.51253 | **0.00105** | 1.0525 | 0.73444 | | **0.00553** | | 1.26729 | | | 0.88256 | | | **0.02973** | | |
| **AMP vs. rSO_2__L** | **MSE** | 0.00887 | | 0.28713 | **4.27e-08*** | 0.01703 | 0.62706 | **4.79e-08*** | 0.02602 | 0.91517 | | **6.70e-08*** | | 0.03609 | | | 1.60136 | | | **2.71e-08*** | | |
|  | **RMSE** | 0.09412 | | 0.53585 | **4.27e-08*** | 0.13032 | 0.79156 | **4.79e-08*** | 0.16129 | 0.95665 | | **4.79e-08*** | | 0.18971 | | | 1.26545 | | | **2.71e-08*** | | |
|  | **MAE** | 0.07167 | | 0.47263 | **5.36e-08*** | 0.09378 | 0.61023 | **2.71e-08*** | 0.11799 | 0.75838 | | **4.27e-08*** | | 0.14537 | | | 0.95167 | | | **3.04e-08*** | | |
| **AMP vs. rSO_2__R** | **MSE** | 0.00887 | | 0.23225 | **7.88e-07*** | 0.01703 | 0.36448 | **1.45e-06*** | 0.02602 | 0.89502 | | **1.07e-06*** | | 0.03609 | | | 1.35895 | | | **5.36e-08*** | | |
|  | **RMSE** | 0.09412 | | 0.48192 | **7.88e-07*** | 0.13032 | 0.60373 | **1.45e-06*** | 0.16129 | 0.94606 | | **9.67e-07*** | | 0.18971 | | | 1.16574 | | | **5.36e-08*** | | |
|  | **MAE** | 0.07167 | | 0.42696 | **9.67e-07*** | 0.09378 | 0.51253 | **9.67e-07*** | 0.11799 | 0.73444 | | **7.11e-07*** | | 0.14537 | | | 0.88256 | | | **1.80e-07*** | | |
|  | | **Rotterdam CT Grade of 1** | | | | | | | | | | | | | | | | | | | | |
| **ICP vs. rSO_2__L** | **MSE** | 0.71822 | | 0.29595 | 1.00000 | 1.31472 | 0.58345 | 1.00000 | 2.10057 | 1.09076 | | 1.00000 | | 3.61103 | | | 2.1669 | | | 1.00000 | | |
|  | **RMSE** | 0.84748 | | 0.54402 | 1.00000 | 1.14661 | 0.76384 | 1.00000 | 1.44931 | 1.0444 | | 1.00000 | | 1.90027 | | | 1.47201 | | | 1.00000 | | |
|  | **MAE** | 0.65479 | | 0.48013 | 1.00000 | 0.88341 | 0.60884 | 1.00000 | 1.12163 | 0.80547 | | 1.00000 | | 1.32986 | | | 1.11126 | | | 1.00000 | | |
| **ICP vs. rSO_2__R** | **MSE** | 0.71822 | | 0.23098 | 1.00000 | 1.31472 | 0.38225 | 1.00000 | 2.10057 | 0.68907 | | 1.00000 | | 3.61103 | | | 1.15591 | | | 1.00000 | | |
|  | **RMSE** | 0.84748 | | 0.4806 | 1.00000 | 1.14661 | 0.61827 | 1.00000 | 1.44931 | 0.8301 | | 1.00000 | | 1.90027 | | | 1.07513 | | | 1.00000 | | |
|  | **MAE** | 0.65479 | | 0.43253 | 1.00000 | 0.88341 | 0.50031 | 1.00000 | 1.12163 | 0.64671 | | 1.00000 | | 1.32986 | | | 0.85854 | | | 1.00000 | | |
| **AMP vs. rSO_2__L** | **MSE** | 0.01505 | | 0.29595 | 1.00000 | 0.03162 | 0.58345 | 1.00000 | 0.05148 | 1.09076 | | 1.00000 | | 0.08131 | | | 2.1669 | | | 1.00000 | | |
|  | **RMSE** | 0.12268 | | 0.54402 | 1.00000 | 0.17773 | 0.76384 | 1.00000 | 0.22689 | 1.0444 | | 1.00000 | | 0.28511 | | | 1.47201 | | | 1.00000 | | |
|  | **MAE** | 0.09245 | | 0.48013 | 1.00000 | 0.12668 | 0.60884 | 1.00000 | 0.16988 | 0.80547 | | 1.00000 | | 0.20146 | | | 1.11126 | | | 1.00000 | | |
| **AMP vs. rSO_2__R** | **MSE** | 0.01505 | | 0.23098 | 1.00000 | 0.03162 | 0.38225 | 1.00000 | 0.05148 | 0.68907 | | 1.00000 | | 0.08131 | | | 1.15591 | | | 1.00000 | | |
|  | **RMSE** | 0.12268 | | 0.4806 | 1.00000 | 0.17773 | 0.61827 | 1.00000 | 0.22689 | 0.8301 | | 1.00000 | | 0.28511 | | | 1.07513 | | | 1.00000 | | |
|  | **MAE** | 0.09245 | | 0.43253 | 1.00000 | 0.12668 | 0.50031 | 1.00000 | 0.16988 | 0.64671 | | 1.00000 | | 0.20146 | | | 0.85854 | | | 1.00000 | | |
|  | | **Rotterdam CT Grade of 2** | | | | | | | | | | | | | | | | | | | | |
| **ICP vs. rSO_2__L** | **MSE** | 0.99464 | | 0.3409 | **0.02024** | 1.7201 | 0.74282 | **0.03064** | 3.46642 | 1.18561 | | **0.01307** | | 4.1027 | | | 2.13489 | | | 0.17349 | | |
|  | **RMSE** | 0.99723 | | 0.58365 | **0.02024** | 1.31139 | 0.85588 | **0.03064** | 1.86182 | 1.07912 | | **0.01307** | | 2.0096 | | | 1.44996 | | | 0.17349 | | |
|  | **MAE** | 0.795 | | 0.49683 | **0.03064** | 1.00455 | 0.67303 | **0.03064** | 1.29316 | 0.84001 | | **0.02024** | | 1.44682 | | | 1.11534 | | | 0.17349 | | |
| **ICP vs. rSO_2__R** | **MSE** | 0.99464 | | 0.33508 | **0.04283** | 1.7201 | 0.64622 | **0.04283** | 3.46642 | 1.28999 | | **0.04283** | | 4.1027 | | | 2.37069 | | | 0.59403 | | |
|  | **RMSE** | 0.99723 | | 0.57442 | **0.04283** | 1.31139 | 0.77378 | **0.04283** | 1.86182 | 1.07269 | | **0.04283** | | 2.0096 | | | 1.42145 | | | 0.59403 | | |
|  | **MAE** | 0.795 | | 0.48322 | **0.04283** | 1.00455 | 0.64894 | **0.04283** | 1.29316 | 0.84096 | | 0.06995 | | 1.44682 | | | 1.14677 | | | 0.74912 | | |
| **AMP vs. rSO_2__L** | **MSE** | 0.01168 | | 0.3409 | **0.00507** | 0.02464 | 0.74282 | **0.00507** | 0.03936 | 1.18561 | | **0.00507** | | 0.06735 | | | 2.13489 | | | **0.00507** | | |
|  | **RMSE** | 0.10803 | | 0.58365 | **0.00507** | 0.15696 | 0.85588 | **0.00507** | 0.19646 | 1.07912 | | **0.00507** | | 0.25671 | | | 1.44996 | | | **0.00507** | | |
|  | **MAE** | 0.08216 | | 0.49683 | **0.00507** | 0.11035 | 0.67303 | **0.00507** | 0.1498 | 0.84001 | | **0.00507** | | 0.1838 | | | 1.11534 | | | **0.00507** | | |
| **AMP vs. rSO_2__R** | **MSE** | 0.01168 | | 0.33508 | **0.01421** | 0.02464 | 0.64622 | **0.01421** | 0.03936 | 1.28999 | | **0.01421** | | 0.06735 | | | 2.37069 | | | **0.01421** | | |
|  | **RMSE** | 0.10803 | | 0.57442 | **0.01421** | 0.15696 | 0.77378 | **0.01421** | 0.19646 | 1.07269 | | **0.01421** | | 0.25671 | | | 1.42145 | | | **0.01421** | | |
|  | **MAE** | 0.08216 | | 0.48322 | **0.01421** | 0.11035 | 0.64894 | **0.01421** | 0.1498 | 0.84096 | | **0.01421** | | 0.1838 | | | 1.14677 | | | **0.01421** | | |
|  | | **Rotterdam CT Grade of 3** | | | | | | | | | | | | | | | | | | | | |
| **ICP vs. rSO_2__L** | **MSE** | 1.5329 | | 0.39135 | **2.52e-07*** | 2.24886 | 0.65613 | **1.09e-06*** | 3.22353 | 1.18351 | | **2.66e-05*** | | 4.90197 | | | 2.04236 | | | **9.96e-05*** | | |
|  | **RMSE** | 1.23792 | | 0.62558 | **2.52e-07*** | 1.49962 | 0.81002 | **1.09e-06*** | 1.79539 | 1.08789 | | **2.66e-05*** | | 2.21385 | | | 1.42902 | | | **9.96e-05*** | | |
|  | **MAE** | 0.98957 | | 0.50751 | **3.94e-07*** | 1.17275 | 0.64727 | **1.09e-06*** | 1.35336 | 0.79343 | | **2.66e-05*** | | 1.57856 | | | 1.06347 | | | **4.69e-04*** | | |
| **ICP vs. rSO_2__R** | **MSE** | 1.5329 | | 0.36124 | **2.11e-07*** | 2.24886 | 0.67185 | **3.93e-07*** | 3.22353 | 1.10324 | | **1.31e-06*** | | 4.90197 | | | 1.58792 | | | **7.20e-06*** | | |
|  | **RMSE** | 1.23792 | | 0.60102 | **2.11e-07*** | 1.49962 | 0.81953 | **3.93e-07*** | 1.79539 | 1.04883 | | **1.31e-06*** | | 2.21385 | | | 1.25993 | | | **7.20e-06*** | | |
|  | **MAE** | 0.98957 | | 0.48985 | **2.47e-07*** | 1.17275 | 0.64424 | **6.21e-07*** | 1.35336 | 0.8342 | | **1.52e-06*** | | 1.57856 | | | 1.00279 | | | **9.46e-06*** | | |
| **AMP vs. rSO_2__L** | **MSE** | 0.03527 | | 0.39135 | **1.01e-07*** | 0.05544 | 0.65613 | **1.01e-07*** | 0.08473 | 1.18351 | | **1.01e-07*** | | 0.11592 | | | 2.04236 | | | **1.01e-07*** | | |
|  | **RMSE** | 0.18779 | | 0.62558 | **1.01e-07*** | 0.23544 | 0.81002 | **1.01e-07*** | 0.29077 | 1.08789 | | **1.01e-07*** | | 0.34045 | | | 1.42902 | | | **1.01e-07*** | | |
|  | **MAE** | 0.14766 | | 0.50751 | **2.52e-07*** | 0.18412 | 0.64727 | **1.01e-07*** | 0.20547 | 0.79343 | | **1.01e-07*** | | 0.25212 | | | 1.06347 | | | **1.01e-07*** | | |
| **AMP vs. rSO_2__R** | **MSE** | 0.03527 | | 0.36124 | **1.54e-07*** | 0.05544 | 0.67185 | **1.54e-07*** | 0.08473 | 1.10324 | | **1.54e-07*** | | 0.11592 | | | 1.58792 | | | **1.54e-07*** | | |
|  | **RMSE** | 0.18779 | | 0.60102 | **1.54e-07*** | 0.23544 | 0.81953 | **1.54e-07*** | 0.29077 | 1.04883 | | **1.54e-07*** | | 0.34045 | | | 1.25993 | | | **1.54e-07*** | | |
|  | **MAE** | 0.14766 | | 0.48985 | **1.54e-07*** | 0.18412 | 0.64424 | **1.54e-07*** | 0.20547 | 0.8342 | | **1.54e-07*** | | 0.25212 | | | 1.00279 | | | **1.54e-07*** | | |
|  | | **Rotterdam CT Grade of 4** | | | | | | | | | | | | | | | | | | | | |
| **ICP vs. rSO_2__L** | **MSE** | 1.26683 | | 0.23643 | **1.54e-07*** | 1.99567 | 0.34634 | **1.79e-07*** | 3.18759 | 0.72501 | | **1.22e-06*** | | 5.27869 | | | 1.29032 | | | **4.54e-06*** | | |
|  | **RMSE** | 1.12551 | | 0.48624 | **1.54e-07*** | 1.41268 | 0.58851 | **1.79e-07*** | 1.78515 | 0.85148 | | **1.22e-06*** | | 2.29751 | | | 1.13592 | | | **4.54e-06*** | | |
|  | **MAE** | 0.90339 | | 0.43787 | **2.08e-07*** | 1.08786 | 0.49296 | **5.10e-07*** | 1.36426 | 0.62388 | | **3.46e-06*** | | 1.64362 | | | 0.88237 | | | **2.31e-05*** | | |
| **ICP vs. rSO_2__R** | **MSE** | 1.26683 | | 0.3093 | **8.21e-07*** | 1.99567 | 0.48423 | **6.60e-06*** | 3.18759 | 0.84171 | | **1.05e-04*** | | 5.27869 | | | 1.53225 | | | **8.73e-04*** | | |
|  | **RMSE** | 1.12551 | | 0.55614 | **8.21e-07*** | 1.41268 | 0.69586 | **6.60e-06*** | 1.78515 | 0.91745 | | **1.05e-04*** | | 2.29751 | | | 1.23784 | | | **8.73e-04*** | | |
|  | **MAE** | 0.90339 | | 0.4772 | **8.90e-07*** | 1.08786 | 0.55522 | **7.10e-06*** | 1.36426 | 0.71097 | | **1.28e-04*** | | 1.64362 | | | 0.91639 | | | **9.76e-04*** | | |
| **AMP vs. rSO_2__L** | **MSE** | 0.02347 | | 0.23643 | **1.97e-08*** | 0.03619 | 0.34634 | **1.20e-08*** | 0.05791 | 0.72501 | | **2.86e-09*** | | 0.09843 | | | 1.29032 | | | **4.11e-10*** | | |
|  | **RMSE** | 0.15319 | | 0.48624 | **1.97e-08*** | 0.19024 | 0.58851 | **1.20e-08*** | 0.24064 | 0.85148 | | **2.86e-09*** | | 0.31269 | | | 1.13592 | | | **4.11e-10*** | | |
|  | **MAE** | 0.11966 | | 0.43787 | **2.13e-08*** | 0.14359 | 0.49296 | **1.54e-08*** | 0.17335 | 0.62388 | | **5.20e-09*** | | 0.21619 | | | 0.88237 | | | **7.04e-10*** | | |
| **AMP vs. rSO_2__R** | **MSE** | 0.02347 | | 0.3093 | **7.22e-08*** | 0.03619 | 0.48423 | **2.98e-08*** | 0.05791 | 0.84171 | | **6.88e-09*** | | 0.09843 | | | 1.53225 | | | **1.01e-09*** | | |
|  | **RMSE** | 0.15319 | | 0.55614 | **7.22e-08*** | 0.19024 | 0.69586 | **2.98e-08*** | 0.24064 | 0.91745 | | **6.88e-09*** | | 0.31269 | | | 1.23784 | | | **1.01e-09*** | | |
|  | **MAE** | 0.11966 | | 0.4772 | **7.22e-08*** | 0.14359 | 0.55522 | **3.90e-08*** | 0.17335 | 0.71097 | | **9.10e-09*** | | 0.21619 | | | 0.91639 | | | **1.65e-09*** | | |
|  | | **Rotterdam CT Grade of 5** | | | | | | | | | | | | | | | | | | | | |
| **ICP vs. rSO_2__L** | **MSE** | 0.73502 | | 0.23102 | **5.39e-05*** | 1.1779 | 0.3535 | **6.74e-04*** | 1.47927 | 0.72198 | | **0.01621** | | 2.47112 | | | 1.38496 | | | **0.03499** | | |
|  | **RMSE** | 0.85695 | | 0.48062 | **5.39e-05*** | 1.0851 | 0.59454 | **6.74e-04*** | 1.2162 | 0.84933 | | **0.01621** | | 1.57176 | | | 1.1756 | | | **0.04074** | | |
|  | **MAE** | 0.66445 | | 0.43039 | **6.37e-04*** | 0.81649 | 0.49557 | **0.00303** | 0.9559 | 0.64741 | | **0.02000** | | 1.1779 | | | 0.88678 | | | 0.05084 | | |
| **ICP vs. rSO_2__R** | **MSE** | 0.73502 | | 0.23229 | **6.24e-05*** | 1.1779 | 0.41111 | **2.03e-04*** | 1.47927 | 0.67333 | | **0.00288** | | 2.47112 | | | 1.30508 | | | **0.00743** | | |
|  | **RMSE** | 0.85695 | | 0.48197 | **6.24e-05*** | 1.0851 | 0.64118 | **2.03e-04*** | 1.2162 | 0.82057 | | **0.00288** | | 1.57176 | | | 1.12247 | | | **0.00779** | | |
|  | **MAE** | 0.66445 | | 0.42545 | **2.77e-04*** | 0.81649 | 0.51228 | **6.83e-04*** | 0.9559 | 0.6322 | | **0.00479** | | 1.1779 | | | 0.86948 | | | **0.01238** | | |
| **AMP vs. rSO_2__L** | **MSE** | 0.00726 | | 0.23102 | **6.68e-11*** | 0.01433 | 0.3535 | **1.37e-10*** | 0.02368 | 0.72198 | | **1.69e-10*** | | 0.03894 | | | 1.38496 | | | **2.06e-10*** | | |
|  | **RMSE** | 0.08518 | | 0.48062 | **6.68e-11*** | 0.11971 | 0.59454 | **1.37e-10*** | 0.15383 | 0.84933 | | **1.12e-10*** | | 0.19415 | | | 1.1756 | | | **2.06e-10*** | | |
|  | **MAE** | 0.06892 | | 0.43039 | **8.22e-11*** | 0.09024 | 0.49557 | **1.12e-10*** | 0.11148 | 0.64741 | | **1.24e-10*** | | 0.14712 | | | 0.88678 | | | **3.09e-10*** | | |
| **AMP vs. rSO_2__R** | **MSE** | 0.00726 | | 0.23229 | **3.19e-09*** | 0.01433 | 0.41111 | **5.17e-09*** | 0.02368 | 0.67333 | | **5.69e-09*** | | 0.03894 | | | 1.30508 | | | **4.81e-10*** | | |
|  | **RMSE** | 0.08518 | | 0.48197 | **3.19e-09*** | 0.11971 | 0.64118 | **5.17e-09*** | 0.15383 | 0.82057 | | **5.17e-09*** | | 0.19415 | | | 1.12247 | | | **4.81e-10*** | | |
|  | **MAE** | 0.06892 | | 0.42545 | **7.58e-09*** | 0.09024 | 0.51228 | **4.70e-09*** | 0.11148 | 0.6322 | | **4.27e-09*** | | 0.14712 | | | 0.86948 | | | **1.46e-09*** | | |
|  | | **Rotterdam CT Grade of 6** | | | | | | | | | | | | | | | | | | | | |
| **ICP vs. rSO_2__L** | **MSE** | 0.32689 | | 0.28382 | 0.29574 | 0.48329 | 0.63587 | 0.91440 | 0.66133 | 1.03987 | | 0.82217 | | 1.32521 | | | 1.85608 | | | 0.44012 | | |
|  | **RMSE** | 0.57154 | | 0.53263 | 0.29574 | 0.69315 | 0.79739 | 0.91440 | 0.81315 | 1.01564 | | 0.82217 | | 1.14745 | | | 1.35914 | | | 0.44012 | | |
|  | **MAE** | 0.45237 | | 0.47331 | 0.40619 | 0.50741 | 0.61968 | 0.91440 | 0.60188 | 0.77744 | | 0.47562 | | 0.76049 | | | 1.03622 | | | 0.18065 | | |
| **ICP vs. rSO_2__R** | **MSE** | 0.32689 | | 0.21799 | 0.14961 | 0.48329 | 0.35522 | 0.36356 | 0.66133 | 0.85666 | | 0.99268 | | 1.32521 | | | 1.64489 | | | 0.79012 | | |
|  | **RMSE** | 0.57154 | | 0.46689 | 0.14961 | 0.69315 | 0.59601 | 0.36356 | 0.81315 | 0.92532 | | 0.99268 | | 1.14745 | | | 1.27764 | | | 0.79012 | | |
|  | **MAE** | 0.45237 | | 0.41482 | 0.21534 | 0.50741 | 0.50722 | 0.77602 | 0.60188 | 0.69618 | | 0.62667 | | 0.76049 | | | 0.96547 | | | 0.29122 | | |
| **AMP vs. rSO_2__L** | **MSE** | 0.00305 | | 0.28382 | **8.63e-09*** | 0.00621 | 0.63587 | **1.09e-08*** | 0.01025 | 1.03987 | | **9.68e-09*** | | 0.01635 | | | 1.85608 | | | **6.84e-09*** | | |
|  | **RMSE** | 0.05519 | | 0.53263 | **8.63e-09*** | 0.07864 | 0.79739 | **1.09e-08*** | 0.10096 | 1.01564 | | **9.68e-09*** | | 0.1274 | | | 1.35914 | | | **6.84e-09*** | | |
|  | **MAE** | 0.04349 | | 0.47331 | **1.22e-08*** | 0.05695 | 0.61968 | **6.09e-09*** | 0.0753 | 0.77744 | | **9.68e-09*** | | 0.09661 | | | 1.03622 | | | **5.41e-09*** | | |
| **AMP vs. rSO_2__R** | **MSE** | 0.00305 | | 0.21799 | **3.61e-09*** | 0.00621 | 0.35522 | **1.08e-08*** | 0.01025 | 0.85666 | | **5.03e-09*** | | 0.01635 | | | 1.64489 | | | **2.88e-09*** | | |
|  | **RMSE** | 0.05519 | | 0.46689 | **3.61e-09*** | 0.07864 | 0.59601 | **1.08e-08*** | 0.10096 | 0.92532 | | **5.03e-09*** | | 0.1274 | | | 1.27764 | | | **2.88e-09*** | | |
|  | **MAE** | 0.04349 | | 0.41482 | **5.61e-09*** | 0.05695 | 0.50722 | **3.23e-09*** | 0.0753 | 0.69618 | | **3.23e-09*** | | 0.09661 | | | 0.96547 | | | **2.30e-09*** | | |
|  | | **Marshall CT Grade of II** | | | | | | | | | | | | | | | | | | | | |
| **ICP vs. rSO_2__L** | **MSE** | 1.02117 | | 0.32208 | 0.08086 | 1.76895 | 0.91641 | 0.08086 | 3.32351 | 1.49882 | | 0.08086 | | 3.32339 | | | 3.03 | | | 0.66252 | | |
|  | **RMSE** | 1.01053 | | 0.56752 | 0.08086 | 1.33002 | 0.95729 | 0.08086 | 1.82305 | 1.22426 | | 0.08086 | | 1.82302 | | | 1.74069 | | | 0.66252 | | |
|  | **MAE** | 0.81531 | | 0.48944 | 0.08086 | 1.05314 | 0.74962 | 0.08086 | 1.36399 | 0.97657 | | 0.08086 | | 1.37345 | | | 1.37945 | | | 1.00000 | | |
| **ICP vs. rSO_2__R** | **MSE** | 1.02117 | | 0.41727 | 0.08086 | 1.76895 | 0.98342 | 0.08086 | 3.32351 | 1.52098 | | 0.08086 | | 3.32339 | | | 2.96954 | | | 0.38273 | | |
|  | **RMSE** | 1.01053 | | 0.64596 | 0.08086 | 1.33002 | 0.99168 | 0.08086 | 1.82305 | 1.23328 | | 0.08086 | | 1.82302 | | | 1.72319 | | | 0.38273 | | |
|  | **MAE** | 0.81531 | | 0.53893 | 0.08086 | 1.05314 | 0.7872 | 0.08086 | 1.36399 | 1.0198 | | 0.08086 | | 1.37345 | | | 1.37273 | | | 1.00000 | | |
| **AMP vs. rSO_2__L** | **MSE** | 0.00572 | | 0.32208 | 0.08086 | 0.01693 | 0.91641 | 0.08086 | 0.02299 | 1.49882 | | 0.08086 | | 0.02932 | | | 3.03 | | | 0.08086 | | |
|  | **RMSE** | 0.07561 | | 0.56752 | 0.08086 | 0.13011 | 0.95729 | 0.08086 | 0.15163 | 1.22426 | | 0.08086 | | 0.17123 | | | 1.74069 | | | 0.08086 | | |
|  | **MAE** | 0.06013 | | 0.48944 | 0.08086 | 0.09805 | 0.74962 | 0.08086 | 0.10714 | 0.97657 | | 0.08086 | | 0.12749 | | | 1.37945 | | | 0.08086 | | |
| **AMP vs. rSO_2__R** | **MSE** | 0.00572 | | 0.41727 | 0.08086 | 0.01693 | 0.98342 | 0.08086 | 0.02299 | 1.52098 | | 0.08086 | | 0.02932 | | | 2.96954 | | | 0.08086 | | |
|  | **RMSE** | 0.07561 | | 0.64596 | 0.08086 | 0.13011 | 0.99168 | 0.08086 | 0.15163 | 1.23328 | | 0.08086 | | 0.17123 | | | 1.72319 | | | 0.08086 | | |
|  | **MAE** | 0.06013 | | 0.53893 | 0.08086 | 0.09805 | 0.7872 | 0.08086 | 0.10714 | 1.0198 | | 0.08086 | | 0.12749 | | | 1.37273 | | | 0.08086 | | |
|  | | **Marshall CT Grade of III** | | | | | | | | | | | | | | | | | | | | |
| **ICP vs. rSO_2__L** | **MSE** | 1.28356 | | 0.34187 | **2.33e-12*** | 1.96238 | 0.61448 | **6.72e-11*** | 3.24386 | 0.97162 | | **1.70e-08*** | | 4.9578 | | | 1.96434 | | | **7.51e-07*** | | |
|  | **RMSE** | 1.13291 | | 0.58467 | **2.33e-12*** | 1.40079 | 0.78364 | **6.72e-11*** | 1.80107 | 0.98567 | | **1.70e-08*** | | 2.22655 | | | 1.40048 | | | **7.94e-07*** | | |
|  | **MAE** | 0.90314 | | 0.49239 | **4.64e-12*** | 1.07561 | 0.61945 | **7.77e-11*** | 1.32939 | 0.7845 | | **1.70e-08*** | | 1.60777 | | | 1.06004 | | | **2.00e-06*** | | |
| **ICP vs. rSO_2__R** | **MSE** | 1.28356 | | 0.34847 | **9.09e-12*** | 1.96238 | 0.65507 | **6.27e-10*** | 3.24386 | 1.19606 | | **1.41e-07*** | | 4.9578 | | | 2.17266 | | | **1.47e-05*** | | |
|  | **RMSE** | 1.13291 | | 0.59012 | **9.09e-12*** | 1.40079 | 0.80935 | **6.27e-10*** | 1.80107 | 1.0935 | | **1.41e-07*** | | 2.22655 | | | 1.47365 | | | **1.47e-05*** | | |
|  | **MAE** | 0.90314 | | 0.49307 | **1.26e-11*** | 1.07561 | 0.61638 | **6.27e-10*** | 1.32939 | 0.78415 | | **8.40e-08*** | | 1.60777 | | | 1.00279 | | | **1.85e-06*** | | |
| **AMP vs. rSO_2__L** | **MSE** | 0.02079 | | 0.34187 | **1.57e-13*** | 0.04149 | 0.61448 | **1.45e-13*** | 0.0599 | 0.97162 | | **1.45e-13*** | | 0.09455 | | | 1.96434 | | | **1.45e-13*** | | |
|  | **RMSE** | 0.1439 | | 0.58467 | **1.57e-13*** | 0.20354 | 0.78364 | **1.45e-13*** | 0.24473 | 0.98567 | | **1.45e-13*** | | 0.30742 | | | 1.40048 | | | **1.45e-13*** | | |
|  | **MAE** | 0.11215 | | 0.49239 | **2.55e-13*** | 0.14351 | 0.61945 | **1.45e-13*** | 0.18314 | 0.7845 | | **1.45e-13*** | | 0.2244 | | | 1.06004 | | | **1.45e-13*** | | |
| **AMP vs. rSO_2__R** | **MSE** | 0.02079 | | 0.34847 | **7.94e-13*** | 0.04149 | 0.65507 | **7.94e-13*** | 0.0599 | 1.19606 | | **7.94e-13*** | | 0.09455 | | | 2.17266 | | | **7.94e-13*** | | |
|  | **RMSE** | 0.1439 | | 0.59012 | **7.94e-13*** | 0.20354 | 0.80935 | **7.94e-13*** | 0.24473 | 1.0935 | | **7.94e-13*** | | 0.30742 | | | 1.47365 | | | **7.94e-13*** | | |
|  | **MAE** | 0.11215 | | 0.49307 | **7.94e-13*** | 0.14351 | 0.61638 | **7.94e-13*** | 0.18314 | 0.78415 | | **7.94e-13*** | | 0.2244 | | | 1.00279 | | | **7.94e-13*** | | |
|  | | **Marshall CT Grade of IV** | | | | | | | | | | | | | | | | | | | | |
| **ICP vs. rSO_2__L** | **MSE** | 1.48114 | | 0.22242 | **7.24e-04*** | 2.17369 | 0.31521 | **6.34e-04*** | 3.46472 | 0.60337 | | **0.00225** | | 6.30577 | | | 1.37217 | | | **0.00254** | | |
|  | **RMSE** | 1.21702 | | 0.47161 | **7.24e-04*** | 1.47422 | 0.56143 | **6.34e-04*** | 1.85967 | 0.77677 | | **0.00225** | | 2.48208 | | | 1.1714 | | | **0.00254** | | |
|  | **MAE** | 0.99372 | | 0.41386 | **8.25e-04*** | 1.17807 | 0.47741 | **0.00176** | 1.44938 | 0.62388 | | **0.00454** | | 1.6616 | | | 0.88924 | | | **0.00568** | | |
| **ICP vs. rSO_2__R** | **MSE** | 1.48114 | | 0.24228 | **9.58e-04*** | 2.17369 | 0.41303 | **0.00168** | 3.46472 | 0.7413 | | **0.00370** | | 6.30577 | | | 1.33809 | | | **0.00540** | | |
|  | **RMSE** | 1.21702 | | 0.49222 | **9.58e-04*** | 1.47422 | 0.64267 | **0.00168** | 1.85967 | 0.86099 | | **0.00370** | | 2.48208 | | | 1.15676 | | | **0.00540** | | |
|  | **MAE** | 0.99372 | | 0.4349 | **0.00146** | 1.17807 | 0.51228 | **0.00286** | 1.44938 | 0.67761 | | **0.00540** | | 1.6616 | | | 0.89708 | | | **0.01235** | | |
| **AMP vs. rSO_2__L** | **MSE** | 0.02221 | | 0.22242 | **1.58e-04*** | 0.03539 | 0.31521 | **1.37e-04*** | 0.0545 | 0.60337 | | **7.53e-05*** | | 0.09038 | | | 1.37217 | | | **2.15e-05*** | | |
|  | **RMSE** | 0.14891 | | 0.47161 | **1.58e-04*** | 0.18812 | 0.56143 | **1.37e-04*** | 0.23333 | 0.77677 | | **7.53e-05*** | | 0.29783 | | | 1.1714 | | | **2.15e-05*** | | |
|  | **MAE** | 0.11695 | | 0.41386 | **1.58e-04*** | 0.1403 | 0.47741 | **1.58e-04*** | 0.1817 | 0.62388 | | **1.18e-04*** | | 0.23918 | | | 0.88924 | | | **2.53e-05*** | | |
| **AMP vs. rSO_2__R** | **MSE** | 0.02221 | | 0.24228 | **6.20e-04*** | 0.03539 | 0.41303 | **3.40e-04*** | 0.0545 | 0.7413 | | **2.13e-04*** | | 0.09038 | | | 1.33809 | | | **8.04e-05*** | | |
|  | **RMSE** | 0.14891 | | 0.49222 | **6.20e-04*** | 0.18812 | 0.64267 | **3.40e-04*** | 0.23333 | 0.86099 | | **2.13e-04*** | | 0.29783 | | | 1.15676 | | | **8.04e-05*** | | |
|  | **MAE** | 0.11695 | | 0.4349 | **0.00127** | 0.1403 | 0.51228 | **3.96e-04*** | 0.1817 | 0.67761 | | **1.82e-04*** | | 0.23918 | | | 0.89708 | | | **4.84e-05*** | | |
|  | | **Marshall CT Grade of V** | | | | | | | | | | | | | | | | | | | | |
| **ICP vs. rSO_2__L** | **MSE** | 0.52766 | | 0.28302 | **9.17e-04*** | 0.83589 | 0.53414 | **0.01244** | 1.19536 | 0.81031 | | 0.07803 | | 2.06118 | | | 1.48688 | | | 0.23060 | | |
|  | **RMSE** | 0.72637 | | 0.53199 | **9.17e-04*** | 0.91427 | 0.73085 | **0.01202** | 1.09333 | 0.90017 | | 0.07803 | | 1.43567 | | | 1.21937 | | | 0.23530 | | |
|  | **MAE** | 0.57124 | | 0.46718 | **0.00806** | 0.68432 | 0.58494 | **0.04464** | 0.83998 | 0.70988 | | 0.21469 | | 1.0338 | | | 0.91168 | | | 0.67042 | | |
| **ICP vs. rSO_2__R** | **MSE** | 0.52766 | | 0.22006 | **1.28e-04*** | 0.83589 | 0.35524 | **6.77e-04*** | 1.19536 | 0.67333 | | **0.00912** | | 2.06118 | | | 1.25995 | | | **0.02782** | | |
|  | **RMSE** | 0.72637 | | 0.46911 | **1.28e-04*** | 0.91427 | 0.59602 | **6.77e-04*** | 1.09333 | 0.82057 | | **0.00912** | | 1.43567 | | | 1.1208 | | | **0.02782** | | |
|  | **MAE** | 0.57124 | | 0.41859 | **3.23e-04*** | 0.68432 | 0.50723 | **0.00572** | 0.83998 | 0.66129 | | **0.04999** | | 1.0338 | | | 0.87846 | | | 0.19542 | | |
| **AMP vs. rSO_2__L** | **MSE** | 0.00606 | | 0.28302 | **8.74e-18*** | 0.01414 | 0.53414 | **1.26e-17*** | 0.02275 | 0.81031 | | **1.02e-17*** | | 0.03384 | | | 1.48688 | | | **4.42e-18*** | | |
|  | **RMSE** | 0.07785 | | 0.53199 | **8.74e-18*** | 0.11892 | 0.73085 | **1.26e-17*** | 0.1508 | 0.90017 | | **7.47e-18*** | | 0.18395 | | | 1.21937 | | | **4.42e-18*** | | |
|  | **MAE** | 0.05939 | | 0.46718 | **1.90e-17*** | 0.07788 | 0.58494 | **5.18e-18*** | 0.09767 | 0.70988 | | **7.47e-18*** | | 0.13389 | | | 0.91168 | | | **4.91e-18*** | | |
| **AMP vs. rSO_2__R** | **MSE** | 0.00606 | | 0.22006 | **2.94e-16*** | 0.01414 | 0.35524 | **7.89e-16*** | 0.02275 | 0.67333 | | **2.29e-16*** | | 0.03384 | | | 1.25995 | | | **6.39e-18*** | | |
|  | **RMSE** | 0.07785 | | 0.46911 | **2.94e-16*** | 0.11892 | 0.59602 | **7.89e-16*** | 0.1508 | 0.82057 | | **1.97e-16*** | | 0.18395 | | | 1.1208 | | | **6.39e-18*** | | |
|  | **MAE** | 0.05939 | | 0.41859 | **7.15e-16*** | 0.07788 | 0.50723 | **2.07e-16*** | 0.09767 | 0.66129 | | **1.61e-16*** | | 0.13389 | | | 0.87846 | | | **2.11e-17*** | | |
|  | | **Under 40** | | | | | | | | | | | | | | | | | | | | |
| **ICP vs. rSO_2__L** | **MSE** | 1.20237 | | 0.28331 | **1.44e-08*** | 1.71948 | 0.61613 | **1.84e-06*** | 2.39555 | 1.09118 | | **1.20e-04*** | | 3.28256 | | | 2.05598 | | | **0.00201** | | |
|  | **RMSE** | 1.09653 | | 0.53214 | **1.44e-08*** | 1.31129 | 0.78492 | **1.84e-06*** | 1.54776 | 1.04366 | | **1.20e-04*** | | 1.81177 | | | 1.43382 | | | **0.00209** | | |
|  | **MAE** | 0.87106 | | 0.46828 | **4.04e-08*** | 1.02353 | 0.62873 | **3.56e-06*** | 1.18397 | 0.78836 | | **2.85e-04*** | | 1.39406 | | | 1.06663 | | | **0.00393** | | |
| **ICP vs. rSO_2__R** | **MSE** | 1.20237 | | 0.32524 | **4.46e-08*** | 1.71948 | 0.56882 | **3.58e-06*** | 2.39555 | 1.16961 | | **2.40e-04*** | | 3.28256 | | | 2.13253 | | | **0.00338** | | |
|  | **RMSE** | 1.09653 | | 0.5703 | **4.46e-08*** | 1.31129 | 0.75412 | **3.58e-06*** | 1.54776 | 1.0814 | | **2.40e-04*** | | 1.81177 | | | 1.45959 | | | **0.00331** | | |
|  | **MAE** | 0.87106 | | 0.4846 | **7.28e-08*** | 1.02353 | 0.60109 | **3.98e-06*** | 1.18397 | 0.83078 | | **2.92e-04*** | | 1.39406 | | | 1.08552 | | | **0.00468** | | |
| **AMP vs. rSO_2__L** | **MSE** | 0.01334 | | 0.28331 | **1.16e-18*** | 0.02199 | 0.61613 | **1.16e-18*** | 0.03523 | 1.09118 | | **1.16e-18*** | | 0.05238 | | | 2.05598 | | | **1.16e-18*** | | |
|  | **RMSE** | 0.1155 | | 0.53214 | **1.16e-18*** | 0.14828 | 0.78492 | **1.16e-18*** | 0.1877 | 1.04366 | | **1.16e-18*** | | 0.22887 | | | 1.43382 | | | **1.16e-18*** | | |
|  | **MAE** | 0.09155 | | 0.46828 | **1.30e-18*** | 0.11538 | 0.62873 | **1.16e-18*** | 0.14683 | 0.78836 | | **1.16e-18*** | | 0.17939 | | | 1.06663 | | | **1.16e-18*** | | |
| **AMP vs. rSO_2__R** | **MSE** | 0.01334 | | 0.32524 | **4.53e-17*** | 0.02199 | 0.56882 | **4.26e-17*** | 0.03523 | 1.16961 | | **4.26e-17*** | | 0.05238 | | | 2.13253 | | | **4.26e-17*** | | |
|  | **RMSE** | 0.1155 | | 0.5703 | **4.53e-17*** | 0.14828 | 0.75412 | **4.26e-17*** | 0.1877 | 1.0814 | | **4.26e-17*** | | 0.22887 | | | 1.45959 | | | **4.26e-17*** | | |
|  | **MAE** | 0.09155 | | 0.4846 | **7.43e-17*** | 0.11538 | 0.60109 | **4.53e-17*** | 0.14683 | 0.83078 | | **4.82e-17*** | | 0.17939 | | | 1.08552 | | | **4.26e-17*** | | |
|  | | **40 - 60** | | | | | | | | | | | | | | | | | | | | |
| **ICP vs. rSO_2__L** | **MSE** | 0.89047 | | 0.29154 | **2.35e-05*** | 1.31523 | 0.55879 | **8.59e-05*** | 2.13207 | 0.86552 | | **0.00119** | | 3.51581 | | | 1.58939 | | | **0.00694** | | |
|  | **RMSE** | 0.94363 | | 0.53993 | **2.35e-05*** | 1.14683 | 0.74734 | **8.59e-05*** | 1.46011 | 0.92995 | | **0.00119** | | 1.86164 | | | 1.2607 | | | **0.00739** | | |
|  | **MAE** | 0.75505 | | 0.48164 | **1.92e-04*** | 0.8957 | 0.59689 | **3.67e-04*** | 1.11096 | 0.73413 | | **0.00539** | | 1.32584 | | | 0.95698 | | | **0.03606** | | |
| **ICP vs. rSO_2__R** | **MSE** | 0.89047 | | 0.24194 | **3.95e-06*** | 1.31523 | 0.36875 | **9.12e-06*** | 2.13207 | 0.7798 | | **4.13e-04*** | | 3.51581 | | | 1.34852 | | | **0.00138** | | |
|  | **RMSE** | 0.94363 | | 0.49187 | **3.95e-06*** | 1.14683 | 0.60714 | **9.12e-06*** | 1.46011 | 0.88279 | | **3.97e-04*** | | 1.86164 | | | 1.16125 | | | **0.00148** | | |
|  | **MAE** | 0.75505 | | 0.43002 | **1.54e-05*** | 0.8957 | 0.50578 | **4.86e-05*** | 1.11096 | 0.68192 | | **0.00165** | | 1.32584 | | | 0.88995 | | | **0.00916** | | |
| **AMP vs. rSO_2__L** | **MSE** | 0.01169 | | 0.29154 | **1.84e-13*** | 0.02129 | 0.55879 | **5.84e-13*** | 0.03646 | 0.86552 | | **5.84e-13*** | | 0.05538 | | | 1.58939 | | | **3.41e-13*** | | |
|  | **RMSE** | 0.10809 | | 0.53993 | **1.84e-13*** | 0.1459 | 0.74734 | **5.84e-13*** | 0.19093 | 0.92995 | | **5.84e-13*** | | 0.23508 | | | 1.2607 | | | **3.41e-13*** | | |
|  | **MAE** | 0.07661 | | 0.48164 | **3.69e-13*** | 0.09782 | 0.59689 | **2.32e-13*** | 0.11974 | 0.73413 | | **3.41e-13*** | | 0.15076 | | | 0.95698 | | | **2.15e-13*** | | |
| **AMP vs. rSO_2__R** | **MSE** | 0.01169 | | 0.24194 | **3.54e-12*** | 0.02129 | 0.36875 | **1.22e-11*** | 0.03646 | 0.7798 | | **1.06e-11*** | | 0.05538 | | | 1.34852 | | | **3.69e-13*** | | |
|  | **RMSE** | 0.10809 | | 0.49187 | **3.54e-12*** | 0.1459 | 0.60714 | **1.22e-11*** | 0.19093 | 0.88279 | | **1.06e-11*** | | 0.23508 | | | 1.16125 | | | **3.69e-13*** | | |
|  | **MAE** | 0.07661 | | 0.43002 | **6.84e-12*** | 0.09782 | 0.50578 | **4.10e-12*** | 0.11974 | 0.68192 | | **6.36e-12*** | | 0.15076 | | | 0.88995 | | | **7.34e-13*** | | |
|  | | **Over 60** | | | | | | | | | | | | | | | | | | | | |
| **ICP vs. rSO_2__L** | **MSE** | 0.80475 | | 0.21725 | **0.00111** | 1.21993 | 0.35909 | **0.00188** | 2.44864 | 0.61229 | | **0.00465** | | 2.77105 | | | 1.14067 | | | **0.00205** | | |
|  | **RMSE** | 0.89708 | | 0.4661 | **0.00111** | 1.10451 | 0.59924 | **0.00188** | 1.56481 | 0.78249 | | **0.00465** | | 1.66451 | | | 1.06542 | | | **0.00205** | | |
|  | **MAE** | 0.72892 | | 0.40153 | **0.00286** | 0.82678 | 0.49142 | **0.00588** | 1.06633 | 0.63562 | | **0.00995** | | 1.23017 | | | 0.79717 | | | **0.02451** | | |
| **ICP vs. rSO_2__R** | **MSE** | 0.80475 | | 0.22582 | **9.23e-04*** | 1.21993 | 0.4012 | **0.00151** | 2.44864 | 0.58455 | | **0.00208** | | 2.77105 | | | 0.98444 | | | **0.00357** | | |
|  | **RMSE** | 0.89708 | | 0.47521 | **9.23e-04*** | 1.10451 | 0.63341 | **0.00151** | 1.56481 | 0.76456 | | **0.00208** | | 1.66451 | | | 0.99219 | | | **0.00357** | | |
|  | **MAE** | 0.72892 | | 0.41859 | **0.00192** | 0.82678 | 0.51291 | **0.00556** | 1.06633 | 0.61231 | | **0.00793** | | 1.23017 | | | 0.76455 | | | **0.01116** | | |
| **AMP vs. rSO_2__L** | **MSE** | 0.0162 | | 0.21725 | **8.97e-05*** | 0.03483 | 0.35909 | **5.87e-05*** | 0.08339 | 0.61229 | | **1.74e-05*** | | 0.09843 | | | 1.14067 | | | **6.85e-06*** | | |
|  | **RMSE** | 0.12726 | | 0.4661 | **8.97e-05*** | 0.18663 | 0.59924 | **5.87e-05*** | 0.28877 | 0.78249 | | **1.38e-05*** | | 0.31269 | | | 1.06542 | | | **6.85e-06*** | | |
|  | **MAE** | 0.09759 | | 0.40153 | **1.36e-04*** | 0.13616 | 0.49142 | **5.27e-05*** | 0.19641 | 0.63562 | | **3.80e-05*** | | 0.243 | | | 0.79717 | | | **1.10e-05*** | | |
| **AMP vs. rSO_2__R** | **MSE** | 0.0162 | | 0.22582 | **6.48e-05*** | 0.03483 | 0.4012 | **3.54e-05*** | 0.08339 | 0.58455 | | **1.54e-05*** | | 0.09843 | | | 0.98444 | | | **4.12e-06*** | | |
|  | **RMSE** | 0.12726 | | 0.47521 | **6.48e-05*** | 0.18663 | 0.63341 | **3.54e-05*** | 0.28877 | 0.76456 | | **1.54e-05*** | | 0.31269 | | | 0.99219 | | | **4.12e-06*** | | |
|  | **MAE** | 0.09759 | | 0.41859 | **1.41e-04*** | 0.13616 | 0.51291 | **3.20e-05*** | 0.19641 | 0.61231 | | **1.54e-05*** | | 0.243 | | | 0.76455 | | | **7.19e-06*** | | |
|  | | **Male** | | | | | | | | | | | | | | | | | | | | |
| **ICP vs. rSO_2__L** | **MSE** | 1.07814 | | 0.27396 | **7.37e-13*** | 1.58442 | 0.51737 | **1.21e-10*** | 2.24517 | 0.86349 | | **1.13e-07*** | | 3.37199 | | | 1.60667 | | | **6.51e-06*** | | |
|  | **RMSE** | 1.03833 | | 0.52341 | **7.37e-13*** | 1.25873 | 0.71917 | **1.21e-10*** | 1.49835 | 0.92921 | | **1.13e-07*** | | 1.81739 | | | 1.26754 | | | **7.07e-06*** | | |
|  | **MAE** | 0.83591 | | 0.46792 | **2.49e-11*** | 0.9595 | 0.56245 | **2.52e-09*** | 1.13904 | 0.71541 | | **1.00e-06*** | | 1.33901 | | | 0.9776 | | | **1.34e-04*** | | |
| **ICP vs. rSO_2__R** | **MSE** | 1.07814 | | 0.24358 | **6.87e-13*** | 1.58442 | 0.47086 | **1.74e-10*** | 2.24517 | 0.86108 | | **2.86e-07*** | | 3.37199 | | | 1.58792 | | | **1.41e-05*** | | |
|  | **RMSE** | 1.03833 | | 0.49354 | **6.87e-13*** | 1.25873 | 0.68619 | **1.74e-10*** | 1.49835 | 0.92793 | | **2.82e-07*** | | 1.81739 | | | 1.25993 | | | **1.47e-05*** | | |
|  | **MAE** | 0.83591 | | 0.45402 | **9.01e-12*** | 0.9595 | 0.54197 | **2.34e-09*** | 1.13904 | 0.71593 | | **1.59e-06*** | | 1.33901 | | | 0.96703 | | | **1.29e-04*** | | |
| **AMP vs. rSO_2__L** | **MSE** | 0.01389 | | 0.27396 | **3.90e-28*** | 0.02516 | 0.51737 | **3.90e-28*** | 0.04347 | 0.86349 | | **1.47e-28*** | | 0.06326 | | | 1.60667 | | | **5.01e-29*** | | |
|  | **RMSE** | 0.11784 | | 0.52341 | **3.90e-28*** | 0.15862 | 0.71917 | **3.90e-28*** | 0.20843 | 0.92921 | | **1.17e-28*** | | 0.25146 | | | 1.26754 | | | **5.01e-29*** | | |
|  | **MAE** | 0.09307 | | 0.46792 | **5.39e-28*** | 0.12089 | 0.56245 | **3.54e-28*** | 0.14711 | 0.71541 | | **1.43e-28*** | | 0.17548 | | | 0.9776 | | | **6.96e-29*** | | |
| **AMP vs. rSO_2__R** | **MSE** | 0.01389 | | 0.24358 | **4.03e-25*** | 0.02516 | 0.47086 | **2.81e-25*** | 0.04347 | 0.86108 | | **8.00e-26*** | | 0.06326 | | | 1.58792 | | | **1.25e-27*** | | |
|  | **RMSE** | 0.11784 | | 0.49354 | **4.03e-25*** | 0.15862 | 0.68619 | **2.81e-25*** | 0.20843 | 0.92793 | | **7.24e-26*** | | 0.25146 | | | 1.25993 | | | **1.21e-27*** | | |
|  | **MAE** | 0.09307 | | 0.45402 | **5.96e-25*** | 0.12089 | 0.54197 | **2.38e-25*** | 0.14711 | 0.71593 | | **7.73e-26*** | | 0.17548 | | | 0.96703 | | | **7.89e-27*** | | |
|  | | **Female** | | | | | | | | | | | | | | | | | | | | |
| **ICP vs. rSO_2__L** | **MSE** | 1.03797 | | 0.27282 | **0.00277** | 1.51761 | 0.53414 | **0.00751** | 2.61676 | 0.81031 | | **0.01119** | | 3.04584 | | | 1.42973 | | | 0.05747 | | |
|  | **RMSE** | 1.01611 | | 0.52232 | **0.00277** | 1.22917 | 0.73085 | **0.00751** | 1.61764 | 0.90017 | | **0.01119** | | 1.74517 | | | 1.19325 | | | 0.05747 | | |
|  | **MAE** | 0.80151 | | 0.46718 | **0.00430** | 0.95595 | 0.58494 | **0.01357** | 1.16041 | 0.70988 | | **0.03534** | | 1.3849 | | | 0.94755 | | | 0.09449 | | |
| **ICP vs. rSO_2__R** | **MSE** | 1.03797 | | 0.2335 | **0.00226** | 1.51761 | 0.35521 | **0.00279** | 2.61676 | 0.6472 | | **0.00513** | | 3.04584 | | | 1.0243 | | | **0.00858** | | |
|  | **RMSE** | 1.01611 | | 0.48322 | **0.00226** | 1.22917 | 0.59599 | **0.00279** | 1.61764 | 0.80449 | | **0.00513** | | 1.74517 | | | 1.01203 | | | **0.00858** | | |
|  | **MAE** | 0.80151 | | 0.41859 | **0.00158** | 0.95595 | 0.50191 | **0.00585** | 1.16041 | 0.6322 | | **0.01099** | | 1.3849 | | | 0.76558 | | | **0.01979** | | |
| **AMP vs. rSO_2__L** | **MSE** | 0.01013 | | 0.27282 | **2.56e-07*** | 0.01792 | 0.53414 | **2.01e-07*** | 0.02802 | 0.81031 | | **2.01e-07*** | | 0.04444 | | | 1.42973 | | | **7.43e-08*** | | |
|  | **RMSE** | 0.1 | | 0.52232 | **2.56e-07*** | 0.13381 | 0.73085 | **2.01e-07*** | 0.16716 | 0.90017 | | **2.01e-07*** | | 0.21066 | | | 1.19325 | | | **7.43e-08*** | | |
|  | **MAE** | 0.07344 | | 0.46718 | **5.26e-07*** | 0.09797 | 0.58494 | **1.57e-07*** | 0.12654 | 0.70988 | | **1.39e-07*** | | 0.1495 | | | 0.94755 | | | **6.54e-08*** | | |
| **AMP vs. rSO_2__R** | **MSE** | 0.01013 | | 0.2335 | **2.19e-07*** | 0.01792 | 0.35521 | **3.45e-07*** | 0.02802 | 0.6472 | | **1.23e-07*** | | 0.04444 | | | 1.0243 | | | **4.25e-08*** | | |
|  | **RMSE** | 0.1 | | 0.48322 | **2.19e-07*** | 0.13381 | 0.59599 | **3.45e-07*** | 0.16716 | 0.80449 | | **1.23e-07*** | | 0.21066 | | | 1.01203 | | | **4.25e-08*** | | |
|  | **MAE** | 0.07344 | | 0.41859 | **6.72e-07*** | 0.09797 | 0.50191 | **1.75e-07*** | 0.12654 | 0.6322 | | **1.23e-07*** | | 0.1495 | | | 0.76558 | | | **6.86e-08*** | | |
| **Physiologic** | **Variance Metric** | **Wilcoxon Signed Rank Test Results – 10 Second Sampling Frequency** | | | | | | | | | | | | | | | | | | | | |
| **Variable Comparison** |  | **Total Population** | | | | | | | | | | | | | | | | | | | | |
|  |  | **5-Minute Window** | | | | **15-Minute Window** | | | **30-Minute Window** | | | | | | **1-Hour Window** | | | | | | | |
|  |  | **Median ICP/AMP** | | **Median** rSO_2_ | **p-value** | **Median ICP/AMP** | **Median** rSO_2_ | **p-value** | **Median ICP/AMP** | | **Median** rSO_2_ | | **p-value** | | **Median ICP/AMP** | | | **Median** rSO_2_ | | | **p-value** | |
| **ICP vs. rSO_2__L** | **MSE** | 0.515081683 | | 0.190518222 | **3.74E-12*** | 0.950175392 | 0.393862654 | **4.05E-09*** | 1.616446431 | | 0.755364122 | | **6.88E-07*** | | 2.676749521 | | | 1.47592275 | | | **1.09E-05*** | |
|  | **RMSE** | 0.717691727 | | 0.436483931 | **5.87E-11*** | 0.972246255 | 0.62748381 | **1.07E-08*** | 1.271394862 | | 0.869116837 | | **2.13E-06*** | | 1.636061615 | | | 1.214875611 | | | **3.33E-05*** | |
|  | **MAE** | 0.560629545 | | 0.376333333 | **2.56E-10*** | 0.763314815 | 0.502118519 | **6.34E-08*** | 0.971853068 | | 0.666222222 | | **8.34E-06*** | | 1.262236745 | | | 0.92968876 | | | **1.42E-04*** | |
| **ICP vs. rSO_2__R** | **MSE** | 0.515081683 | | 0.172213889 | **2.53E-13*** | 0.950175392 | 0.343833216 | **2.19E-11*** | 1.616446431 | | 0.701147235 | | **1.83E-07*** | | 2.676749521 | | | 1.449240004 | | | **4.78E-06*** | |
|  | **RMSE** | 0.717691727 | | 0.414986613 | **1.01E-12*** | 0.972246255 | 0.586370907 | **6.19E-11*** | 1.271394862 | | 0.837344251 | | **3.32E-07*** | | 1.636061615 | | | 1.203842722 | | | **6.03E-06*** | |
|  | **MAE** | 0.560629545 | | 0.347866667 | **2.56E-12*** | 0.763314815 | 0.479764198 | **8.37E-10*** | 0.971853068 | | 0.661775309 | | **1.17E-06*** | | 1.262236745 | | | 0.886548611 | | | **1.57E-05*** | |
| **AMP vs. rSO_2__L** | **MSE** | 0.012245591 | | 0.190518222 | **1.09E-17*** | 0.021323645 | 0.393862654 | **1.00E-17*** | 0.036609859 | | 0.755364122 | | **1.52E-18*** | | 0.061659338 | | | 1.47592275 | | | **9.37E-19*** | |
|  | **RMSE** | 0.110649973 | | 0.436483931 | **5.89E-18*** | 0.146022011 | 0.62748381 | **1.52E-18*** | 0.191311448 | | 0.869116837 | | **8.12E-19*** | | 0.246822755 | | | 1.214875611 | | | **7.45E-19*** | |
|  | **MAE** | 0.087900556 | | 0.376333333 | **7.58E-18*** | 0.114828642 | 0.502118519 | **1.86E-18*** | 0.142355336 | | 0.666222222 | | **7.45E-19*** | | 0.170089547 | | | 0.92968876 | | | **8.60E-19*** | |
| **AMP vs. rSO_2__R** | **MSE** | 0.012245591 | | 0.172213889 | **1.12E-16*** | 0.021323645 | 0.343833216 | **2.93E-16*** | 0.036609859 | | 0.701147235 | | **7.65E-17*** | | 0.061659338 | | | 1.449240004 | | | **1.57E-17*** | |
|  | **RMSE** | 0.110649973 | | 0.414986613 | **7.88E-17*** | 0.146022011 | 0.586370907 | **3.98E-17*** | 0.191311448 | | 0.837344251 | | **2.26E-17*** | | 0.246822755 | | | 1.203842722 | | | **9.13E-18*** | |
|  | **MAE** | 0.087900556 | | 0.347866667 | **9.40E-17*** | 0.114828642 | 0.479764198 | **2.47E-17*** | 0.142355336 | | 0.661775309 | | **9.42E-18*** | | 0.170089547 | | | 0.886548611 | | | **1.06E-17*** | |
|  | | **ICP Monitor Same Side as NIRS** | | | | | | | | | | | | | | | | | | | | |
| **ICP vs. rSO_2__L** | **MSE** | 0.51522 | | 0.18356 | **3.52e-10*** | 0.95801 | 0.36195 | **3.73e-08*** | 1.6133 | | 0.71405 | | **8.60e-06*** | | 2.67853 | | | 1.43952 | | | **1.27e-04*** | |
|  | **RMSE** | 0.71779 | | 0.42843 | **3.52e-10*** | 0.97375 | 0.60149 | **3.82e-08*** | 1.27016 | | 0.84501 | | **8.87e-06*** | | 1.63659 | | | 1.19979 | | | **1.40e-04*** | |
|  | **MAE** | 0.56141 | | 0.36905 | **1.29e-09*** | 0.76735 | 0.4934 | **3.60e-07*** | 0.95745 | | 0.64939 | | **3.94e-05*** | | 1.26888 | | | 0.89577 | | | **3.09e-04*** | |
| **ICP vs. rSO_2__R** | **MSE** | 0.51522 | | 0.17221 | **4.50e-11*** | 0.95801 | 0.34383 | **4.40e-09*** | 1.6133 | | 0.70115 | | **5.87e-06*** | | 2.67853 | | | 1.44924 | | | **7.19e-05*** | |
|  | **RMSE** | 0.71779 | | 0.41499 | **4.50e-11*** | 0.97375 | 0.58637 | **4.65e-09*** | 1.27016 | | 0.83734 | | **6.06e-06*** | | 1.63659 | | | 1.20384 | | | **7.47e-05*** | |
|  | **MAE** | 0.56141 | | 0.34787 | **1.07e-10*** | 0.76735 | 0.47976 | **5.34e-08*** | 0.95745 | | 0.66178 | | **2.11e-05*** | | 1.26888 | | | 0.88655 | | | **1.43e-04*** | |
| **AMP vs. rSO_2__L** | **MSE** | 0.01193 | | 0.18356 | **2.59e-32*** | 0.02124 | 0.36195 | **3.21e-32*** | 0.03541 | | 0.71405 | | **4.96e-33*** | | 0.06098 | | | 1.43952 | | | **6.01e-34*** | |
|  | **RMSE** | 0.10925 | | 0.42843 | **2.59e-32*** | 0.14574 | 0.60149 | **3.12e-32*** | 0.18818 | | 0.84501 | | **3.79e-33*** | | 0.2467 | | | 1.19979 | | | **6.01e-34*** | |
|  | **MAE** | 0.08663 | | 0.36905 | **4.07e-32*** | 0.11439 | 0.4934 | **2.15e-32*** | 0.14061 | | 0.64939 | | **1.36e-33*** | | 0.16315 | | | 0.89577 | | | **5.85e-34*** | |
| **AMP vs. rSO_2__R** | **MSE** | 0.01193 | | 0.17221 | **1.65e-30*** | 0.02124 | 0.34383 | **7.54e-31*** | 0.03541 | | 0.70115 | | **7.63e-32*** | | 0.06098 | | | 1.44924 | | | **4.63e-34*** | |
|  | **RMSE** | 0.10925 | | 0.41499 | **1.65e-30*** | 0.14574 | 0.58637 | **7.16e-31*** | 0.18818 | | 0.83734 | | **6.17e-32*** | | 0.2467 | | | 1.20384 | | | **4.63e-34*** | |
|  | **MAE** | 0.08663 | | 0.34787 | **7.74e-30*** | 0.11439 | 0.47976 | **4.83e-31*** | 0.14061 | | 0.66178 | | **3.82e-32*** | | 0.16315 | | | 0.88655 | | | **8.94e-34*** | |
|  | | **ICP Monitor Opposite Side as NIRS** | | | | | | | | | | | | | | | | | | | | |
| **ICP vs. rSO_2__L** | **MSE** | 0.51494 | | 0.19052 | **2.13e-09*** | 0.94234 | 0.39386 | **1.71e-07*** | 1.6133 | | 0.75536 | | **2.50e-05*** | | 2.67853 | | | 1.47592 | | | **4.38e-04*** | |
|  | **RMSE** | 0.7176 | | 0.43648 | **2.13e-09*** | 0.97074 | 0.62748 | **1.77e-07*** | 1.27016 | | 0.86912 | | **2.57e-05*** | | 1.63659 | | | 1.21488 | | | **4.78e-04*** | |
|  | **MAE** | 0.55985 | | 0.37633 | **6.82e-09*** | 0.75928 | 0.50212 | **1.47e-06*** | 0.95745 | | 0.66622 | | **1.24e-04*** | | 1.26673 | | | 0.92969 | | | **0.00115** | |
| **ICP vs. rSO_2__R** | **MSE** | 0.51494 | | 0.17233 | **2.64e-10*** | 0.94234 | 0.34534 | **1.76e-08*** | 1.6133 | | 0.70572 | | **1.07e-05*** | | 2.67853 | | | 1.45459 | | | **1.32e-04*** | |
|  | **RMSE** | 0.7176 | | 0.41512 | **2.64e-10*** | 0.97074 | 0.58765 | **1.85e-08*** | 1.27016 | | 0.84005 | | **1.10e-05*** | | 1.63659 | | | 1.20479 | | | **1.37e-04*** | |
|  | **MAE** | 0.55985 | | 0.35031 | **6.42e-10*** | 0.75928 | 0.47994 | **1.90e-07*** | 0.95745 | | 0.67118 | | **4.26e-05*** | | 1.26673 | | | 0.89391 | | | **3.18e-04*** | |
| **AMP vs. rSO_2__L** | **MSE** | 0.01193 | | 0.19052 | **8.93e-34*** | 0.02124 | 0.39386 | **1.07e-33*** | 0.03541 | | 0.75536 | | **1.48e-34*** | | 0.06098 | | | 1.47592 | | | **1.75e-35*** | |
|  | **RMSE** | 0.10925 | | 0.43648 | **8.93e-34*** | 0.14574 | 0.62748 | **1.04e-33*** | 0.18818 | | 0.86912 | | **1.14e-34*** | | 0.2467 | | | 1.21488 | | | **1.75e-35*** | |
|  | **MAE** | 0.08663 | | 0.37633 | **1.56e-33*** | 0.11439 | 0.50212 | **7.47e-34*** | 0.14061 | | 0.66622 | | **3.74e-35*** | | 0.16315 | | | 0.92969 | | | **1.50e-35*** | |
| **AMP vs. rSO_2__R** | **MSE** | 0.01193 | | 0.17233 | **8.11e-31*** | 0.02124 | 0.34534 | **3.66e-31*** | 0.03541 | | 0.70572 | | **3.19e-32*** | | 0.06098 | | | 1.45459 | | | **1.86e-34*** | |
|  | **RMSE** | 0.10925 | | 0.41512 | **8.11e-31*** | 0.14574 | 0.58765 | **3.47e-31*** | 0.18818 | | 0.84005 | | **2.58e-32*** | | 0.2467 | | | 1.20479 | | | **1.86e-34*** | |
|  | **MAE** | 0.08663 | | 0.35031 | **3.83e-30*** | 0.11439 | 0.47994 | **2.02e-31*** | 0.14061 | | 0.67118 | | **1.49e-32*** | | 0.16315 | | | 0.89391 | | | **3.37e-34*** | |
|  | | **Primary Decompressive Craniectomy** | | | | | | | | | | | | | | | | | | | | |
| **ICP vs. rSO_2__L** | **MSE** | 0.44099 | | 0.15904 | **0.01077** | 0.87833 | 0.29055 | **0.00837** | 1.0922 | | 0.58663 | | **0.02883** | | 2.57849 | | | 1.61451 | | | 0.06099 | |
|  | **RMSE** | 0.66407 | | 0.39879 | **0.01077** | 0.93719 | 0.53902 | **0.00837** | 1.04509 | | 0.76592 | | **0.03012** | | 1.60577 | | | 1.25504 | | | 0.06343 | |
|  | **MAE** | 0.53493 | | 0.32427 | **0.00837** | 0.74141 | 0.43271 | **0.02009** | 0.84218 | | 0.62388 | | 0.05636 | | 1.28661 | | | 0.94169 | | | **0.04066** | |
| **ICP vs. rSO_2__R** | **MSE** | 0.44099 | | 0.1593 | **0.00184** | 0.87833 | 0.3362 | **0.00242** | 1.0922 | | 0.53251 | | **0.01329** | | 2.57849 | | | 1.04638 | | | **0.04040** | |
|  | **RMSE** | 0.66407 | | 0.39913 | **0.00184** | 0.93719 | 0.57983 | **0.00255** | 1.04509 | | 0.72973 | | **0.01593** | | 1.60577 | | | 1.02293 | | | **0.04203** | |
|  | **MAE** | 0.53493 | | 0.33524 | **0.00205** | 0.74141 | 0.47102 | **0.00559** | 0.84218 | | 0.57153 | | **0.02920** | | 1.28661 | | | 0.77612 | | | **0.03583** | |
| **AMP vs. rSO_2__L** | **MSE** | 0.00987 | | 0.15904 | **4.08e-09*** | 0.01531 | 0.29055 | **5.90e-10*** | 0.043 | | 0.58663 | | **1.27e-09*** | | 0.06234 | | | 1.61451 | | | **1.41e-09*** | |
|  | **RMSE** | 0.09936 | | 0.39879 | **4.08e-09*** | 0.12374 | 0.53902 | **5.90e-10*** | 0.20736 | | 0.76592 | | **1.27e-09*** | | 0.243 | | | 1.25504 | | | **1.41e-09*** | |
|  | **MAE** | 0.07869 | | 0.32427 | **5.58e-09*** | 0.09835 | 0.43271 | **4.23e-10*** | 0.11553 | | 0.62388 | | **6.58e-10*** | | 0.1534 | | | 0.94169 | | | **1.02e-09*** | |
| **AMP vs. rSO_2__R** | **MSE** | 0.00987 | | 0.1593 | **9.58e-10*** | 0.01531 | 0.3362 | **1.44e-10*** | 0.043 | | 0.53251 | | **1.99e-10*** | | 0.06234 | | | 1.04638 | | | **1.79e-10*** | |
|  | **RMSE** | 0.09936 | | 0.39913 | **9.58e-10*** | 0.12374 | 0.57983 | **1.44e-10*** | 0.20736 | | 0.72973 | | **1.99e-10*** | | 0.243 | | | 1.02293 | | | **1.79e-10*** | |
|  | **MAE** | 0.07869 | | 0.33524 | **2.92e-09*** | 0.09835 | 0.47102 | **1.44e-10*** | 0.11553 | | 0.57153 | | **1.44e-10*** | | 0.1534 | | | 0.77612 | | | **1.79e-10*** | |
|  | | **Secondary Decompressive Craniectomy** | | | | | | | | | | | | | | | | | | | | |
| **ICP vs. rSO_2__L** | **MSE** | 0.55748 | | 0.21677 | 0.09670 | 1.19465 | 0.50527 | **0.02984** | 2.50601 | | 0.98114 | | **0.02984** | | 5.46021 | | | 1.45638 | | | 0.09670 | |
|  | **RMSE** | 0.74664 | | 0.46559 | 0.09670 | 1.093 | 0.71083 | **0.02984** | 1.58303 | | 0.99052 | | **0.02984** | | 2.33671 | | | 1.2068 | | | 0.09670 | |
|  | **MAE** | 0.58011 | | 0.39693 | 0.15986 | 0.85025 | 0.56579 | **0.02984** | 1.20187 | | 0.74294 | | **0.02984** | | 1.79905 | | | 0.87728 | | | 0.05528 | |
| **ICP vs. rSO_2__R** | **MSE** | 0.55748 | | 0.20698 | 0.10438 | 1.19465 | 0.60274 | 0.10438 | 2.50601 | | 1.52978 | | 0.19388 | | 5.46021 | | | 2.45129 | | | 0.19388 | |
|  | **RMSE** | 0.74664 | | 0.45495 | 0.10438 | 1.093 | 0.77634 | 0.10438 | 1.58303 | | 1.23684 | | 0.19388 | | 2.33671 | | | 1.55918 | | | 0.19388 | |
|  | **MAE** | 0.58011 | | 0.36964 | 0.25562 | 0.85025 | 0.64506 | 0.19388 | 1.20187 | | 1.00222 | | 0.19388 | | 1.79905 | | | 1.20681 | | | 0.19388 | |
| **AMP vs. rSO_2__L** | **MSE** | 0.01366 | | 0.21677 | **0.04091** | 0.03336 | 0.50527 | **0.02984** | 0.0509 | | 0.98114 | | **0.00729** | | 0.0762 | | | 1.45638 | | | **0.00494** | |
|  | **RMSE** | 0.11686 | | 0.46559 | **0.04091** | 0.18264 | 0.71083 | **0.02984** | 0.2256 | | 0.99052 | | **0.00729** | | 0.27595 | | | 1.2068 | | | **0.00494** | |
|  | **MAE** | 0.09124 | | 0.39693 | **0.04091** | 0.12628 | 0.56579 | **0.02984** | 0.17111 | | 0.74294 | | **0.01519** | | 0.19815 | | | 0.87728 | | | **0.00729** | |
| **AMP vs. rSO_2__R** | **MSE** | 0.01366 | | 0.20698 | 0.10438 | 0.03336 | 0.60274 | 0.05132 | 0.0509 | | 1.52978 | | **0.01485** | | 0.0762 | | | 2.45129 | | | **0.00937** | |
|  | **RMSE** | 0.11686 | | 0.45495 | 0.10438 | 0.18264 | 0.77634 | 0.05132 | 0.2256 | | 1.23684 | | **0.01485** | | 0.27595 | | | 1.55918 | | | **0.00937** | |
|  | **MAE** | 0.09124 | | 0.36964 | 0.07404 | 0.12628 | 0.64506 | 0.05132 | 0.17111 | | 1.00222 | | **0.01485** | | 0.19815 | | | 1.20681 | | | **0.01485** | |
|  | | **No Decompressive Craniectomy** | | | | | | | | | | | | | | | | | | | | |
| **ICP vs. rSO_2__L** | **MSE** | 0.51508 | | 0.19634 | **1.96e-07*** | 0.92339 | 0.42816 | **2.14e-05*** | 1.52524 | | 0.76976 | | **8.40e-04*** | | 2.62275 | | | 1.47592 | | | **0.00607** | |
|  | **RMSE** | 0.71769 | | 0.4431 | **1.96e-07*** | 0.9609 | 0.65434 | **2.14e-05*** | 1.23449 | | 0.87736 | | **8.40e-04*** | | 1.61937 | | | 1.21488 | | | **0.00641** | |
|  | **MAE** | 0.56063 | | 0.37721 | **3.82e-07*** | 0.75257 | 0.51554 | **9.54e-05*** | 0.92915 | | 0.67293 | | **0.00230** | | 1.16804 | | | 0.92969 | | | **0.02067** | |
| **ICP vs. rSO_2__R** | **MSE** | 0.51508 | | 0.17244 | **8.71e-08*** | 0.92339 | 0.34685 | **4.54e-06*** | 1.52524 | | 0.77635 | | **5.05e-04*** | | 2.62275 | | | 1.70316 | | | **0.00444** | |
|  | **RMSE** | 0.71769 | | 0.41525 | **8.71e-08*** | 0.9609 | 0.58894 | **4.54e-06*** | 1.23449 | | 0.88104 | | **4.91e-04*** | | 1.61937 | | | 1.30505 | | | **0.00444** | |
|  | **MAE** | 0.56063 | | 0.35276 | **1.35e-07*** | 0.75257 | 0.48159 | **1.78e-05*** | 0.92915 | | 0.69536 | | **0.00101*** | | 1.16804 | | | 0.95025 | | | **0.00604** | |
| **AMP vs. rSO_2__L** | **MSE** | 0.01225 | | 0.19634 | **2.19e-25*** | 0.02132 | 0.42816 | **2.05e-24*** | 0.03428 | | 0.76976 | | **7.25e-25*** | | 0.06055 | | | 1.47592 | | | **1.68e-25*** | |
|  | **RMSE** | 0.11065 | | 0.4431 | **2.19e-25*** | 0.14602 | 0.65434 | **2.05e-24*** | 0.18511 | | 0.87736 | | **5.38e-25*** | | 0.24603 | | | 1.21488 | | | **1.68e-25*** | |
|  | **MAE** | 0.0879 | | 0.37721 | **2.27e-25*** | 0.11483 | 0.51554 | **1.97e-24*** | 0.14236 | | 0.67293 | | **3.19e-25*** | | 0.1701 | | | 0.92969 | | | **1.95e-25*** | |
| **AMP vs. rSO_2__R** | **MSE** | 0.01225 | | 0.17244 | **1.75e-22*** | 0.02132 | 0.34685 | **7.78e-22*** | 0.03428 | | 0.77635 | | **1.89e-22*** | | 0.06055 | | | 1.70316 | | | **2.02e-24*** | |
|  | **RMSE** | 0.11065 | | 0.41525 | **1.75e-22*** | 0.14602 | 0.58894 | **7.50e-22*** | 0.18511 | | 0.88104 | | **1.50e-22*** | | 0.24603 | | | 1.30505 | | | **2.02e-24*** | |
|  | **MAE** | 0.0879 | | 0.35276 | **3.07e-22*** | 0.11483 | 0.48159 | **5.57e-22*** | 0.14236 | | 0.69536 | | **9.21e-23*** | | 0.1701 | | | 0.95025 | | | **3.78e-24*** | |
|  | | **Glasgow Coma Scale of 8 or less** | | | | | | | | | | | | | | | | | | | | |
| **ICP vs. rSO_2__L** | **MSE** | 0.56106 | | 0.21366 | **2.36e-08*** | 1.01644 | 0.48394 | **2.55e-06*** | 1.76209 | | 0.91196 | | **1.70e-04*** | | 3.05512 | | | 1.65977 | | | **0.00357** | |
|  | **RMSE** | 0.74903 | | 0.46223 | **2.36e-08*** | 1.00806 | 0.69553 | **2.64e-06*** | 1.32743 | | 0.95479 | | **1.77e-04*** | | 1.74789 | | | 1.2865 | | | **0.00373** | |
|  | **MAE** | 0.59506 | | 0.38299 | **4.00e-08*** | 0.79394 | 0.5432 | **9.72e-06*** | 1.02628 | | 0.73443 | | **5.37e-04*** | | 1.34105 | | | 0.94947 | | | **0.00628** | |
| **ICP vs. rSO_2__R** | **MSE** | 0.56106 | | 0.19437 | **3.59e-09*** | 1.01644 | 0.37959 | **3.06e-07*** | 1.76209 | | 0.87701 | | **1.33e-04*** | | 3.05512 | | | 1.81822 | | | **0.00229** | |
|  | **RMSE** | 0.74903 | | 0.44086 | **3.59e-09*** | 1.00806 | 0.61611 | **3.29e-07*** | 1.32743 | | 0.93637 | | **1.37e-04*** | | 1.74789 | | | 1.34839 | | | **0.00232** | |
|  | **MAE** | 0.59506 | | 0.37581 | **6.47e-09*** | 0.79394 | 0.49011 | **1.54e-06*** | 1.02628 | | 0.73911 | | **3.43e-04*** | | 1.34105 | | | 1.02679 | | | **0.00378** | |
| **AMP vs. rSO_2__L** | **MSE** | 0.01225 | | 0.21366 | **1.76e-25*** | 0.02161 | 0.48394 | **2.80e-25*** | 0.03885 | | 0.91196 | | **1.06e-25*** | | 0.06523 | | | 1.65977 | | | **2.29e-26*** | |
|  | **RMSE** | 0.11065 | | 0.46223 | **1.76e-25*** | 0.14699 | 0.69553 | **2.71e-25*** | 0.19691 | | 0.95479 | | **1.06e-25*** | | 0.25534 | | | 1.2865 | | | **2.29e-26*** | |
|  | **MAE** | 0.0879 | | 0.38299 | **3.48e-25*** | 0.11636 | 0.5432 | **2.91e-25*** | 0.14593 | | 0.73443 | | **4.43e-26*** | | 0.17796 | | | 0.94947 | | | **1.48e-26*** | |
| **AMP vs. rSO_2__R** | **MSE** | 0.01225 | | 0.19437 | **1.57e-23*** | 0.02161 | 0.37959 | **1.41e-23*** | 0.03885 | | 0.87701 | | **3.29e-24*** | | 0.06523 | | | 1.81822 | | | **3.97e-26*** | |
|  | **RMSE** | 0.11065 | | 0.44086 | **1.57e-23*** | 0.14699 | 0.61611 | **1.32e-23*** | 0.19691 | | 0.93637 | | **3.29e-24*** | | 0.25534 | | | 1.34839 | | | **3.97e-26*** | |
|  | **MAE** | 0.0879 | | 0.37581 | **6.59e-23*** | 0.11636 | 0.49011 | **1.14e-23*** | 0.14593 | | 0.73911 | | **1.85e-24*** | | 0.17796 | | | 1.02679 | | | **5.55e-26*** | |
|  | | **Glasgow Coma Scale of 9 - 12** | | | | | | | | | | | | | | | | | | | | |
| **ICP vs. rSO_2__L** | **MSE** | 0.21492 | | 0.14478 | 0.19793 | 0.62467 | 0.30721 | 0.07011 | 0.99808 | | 0.6041 | | 0.19793 | | 1.9995 | | | 1.20216 | | | 0.13214 | |
|  | **RMSE** | 0.46359 | | 0.38047 | 0.19793 | 0.79036 | 0.55404 | 0.07011 | 0.99904 | | 0.77716 | | 0.19793 | | 1.414 | | | 1.09598 | | | 0.14372 | |
|  | **MAE** | 0.37329 | | 0.31205 | 0.24745 | 0.58594 | 0.45109 | 0.16920 | 0.76864 | | 0.63473 | | 0.30507 | | 1.06669 | | | 0.82316 | | | 0.21356 | |
| **ICP vs. rSO_2__R** | **MSE** | 0.21492 | | 0.16808 | 0.17107 | 0.62467 | 0.3175 | 0.14658 | 0.99808 | | 0.53538 | | 0.26275 | | 1.9995 | | | 1.07955 | | | 0.14658 | |
|  | **RMSE** | 0.46359 | | 0.40998 | 0.17107 | 0.79036 | 0.56347 | 0.14658 | 0.99904 | | 0.73169 | | 0.26275 | | 1.414 | | | 1.03901 | | | 0.15846 | |
|  | **MAE** | 0.37329 | | 0.34453 | 0.19851 | 0.58594 | 0.45208 | 0.26275 | 0.76864 | | 0.5705 | | 0.31951 | | 1.06669 | | | 0.8148 | | | 0.36150 | |
| **AMP vs. rSO_2__L** | **MSE** | 0.00987 | | 0.14478 | **4.43e-04*** | 0.01531 | 0.30721 | **1.60e-04*** | 0.043 | | 0.6041 | | **2.54e-05*** | | 0.06633 | | | 1.20216 | | | **1.15e-05*** | |
|  | **RMSE** | 0.09936 | | 0.38047 | **4.43e-04*** | 0.12374 | 0.55404 | **1.60e-04*** | 0.20736 | | 0.77716 | | **2.54e-05*** | | 0.24695 | | | 1.09598 | | | **1.15e-05*** | |
|  | **MAE** | 0.07869 | | 0.31205 | **3.17e-04*** | 0.09301 | 0.45109 | **1.34e-04*** | 0.11703 | | 0.63473 | | **2.09e-05*** | | 0.15047 | | | 0.82316 | | | **1.15e-05*** | |
| **AMP vs. rSO_2__R** | **MSE** | 0.00987 | | 0.16808 | **3.08e-04*** | 0.01531 | 0.3175 | **9.66e-05*** | 0.043 | | 0.53538 | | **2.33e-05*** | | 0.06633 | | | 1.07955 | | | **7.48e-06*** | |
|  | **RMSE** | 0.09936 | | 0.40998 | **3.08e-04*** | 0.12374 | 0.56347 | **9.66e-05*** | 0.20736 | | 0.73169 | | **2.33e-05*** | | 0.24695 | | | 1.03901 | | | **7.48e-06*** | |
|  | **MAE** | 0.07869 | | 0.34453 | **1.60e-04*** | 0.09301 | 0.45208 | **5.74e-05*** | 0.11703 | | 0.5705 | | **1.94e-05*** | | 0.15047 | | | 0.8148 | | | **9.07e-06*** | |
|  | | **Pupil Response Category – Bilateral Reactive** | | | | | | | | | | | | | | | | | | | | |
| **ICP vs. rSO_2__L** | **MSE** | 0.56464 | | 0.18811 | **3.89e-07*** | 1.05419 | 0.38836 | **1.98e-06*** | 1.84324 | | 0.74282 | | **2.68e-05*** | | 3.06251 | | | 1.47592 | | | **1.28e-04*** | |
|  | **RMSE** | 0.75142 | | 0.43372 | **3.89e-07*** | 1.02674 | 0.62318 | **1.87e-06*** | 1.35766 | | 0.86186 | | **2.68e-05*** | | 1.75 | | | 1.21488 | | | **1.45e-04*** | |
|  | **MAE** | 0.61002 | | 0.37721 | **1.40e-06*** | 0.8072 | 0.50144 | **7.49e-06*** | 1.03825 | | 0.66622 | | **6.25e-05*** | | 1.34779 | | | 0.93854 | | | **3.42e-04*** | |
| **ICP vs. rSO_2__R** | **MSE** | 0.56464 | | 0.17233 | **2.27e-07*** | 1.05419 | 0.34208 | **1.77e-06*** | 1.84324 | | 0.69771 | | **7.11e-05*** | | 3.06251 | | | 1.50593 | | | **2.37e-04*** | |
|  | **RMSE** | 0.75142 | | 0.41512 | **2.27e-07*** | 1.02674 | 0.58487 | **1.77e-06*** | 1.35766 | | 0.83529 | | **7.11e-05*** | | 1.75 | | | 1.22706 | | | **2.45e-04*** | |
|  | **MAE** | 0.61002 | | 0.35588 | **4.75e-07*** | 0.8072 | 0.47982 | **7.58e-06*** | 1.03825 | | 0.66031 | | **9.77e-05*** | | 1.34779 | | | 0.90438 | | | **5.32e-04*** | |
| **AMP vs. rSO_2__L** | **MSE** | 0.01366 | | 0.18811 | **2.19e-23*** | 0.03 | 0.38836 | **5.52e-23*** | 0.04925 | | 0.74282 | | **1.38e-23*** | | 0.07263 | | | 1.47592 | | | **2.67e-24*** | |
|  | **RMSE** | 0.11686 | | 0.43372 | **2.19e-23*** | 0.17321 | 0.62318 | **5.31e-23*** | 0.22192 | | 0.86186 | | **1.38e-23*** | | 0.2695 | | | 1.21488 | | | **2.67e-24*** | |
|  | **MAE** | 0.09217 | | 0.37721 | **2.28e-23*** | 0.12628 | 0.50144 | **3.35e-23*** | 0.15838 | | 0.66622 | | **5.00e-24*** | | 0.19677 | | | 0.93854 | | | **2.11e-24*** | |
| **AMP vs. rSO_2__R** | **MSE** | 0.01366 | | 0.17233 | **1.37e-21*** | 0.03 | 0.34208 | **1.90e-21*** | 0.04925 | | 0.69771 | | **3.98e-22*** | | 0.07263 | | | 1.50593 | | | **6.60e-23*** | |
|  | **RMSE** | 0.11686 | | 0.41512 | **1.37e-21*** | 0.17321 | 0.58487 | **1.82e-21*** | 0.22192 | | 0.83529 | | **3.98e-22*** | | 0.2695 | | | 1.22706 | | | **6.60e-23*** | |
|  | **MAE** | 0.09217 | | 0.35588 | **2.14e-21*** | 0.12628 | 0.47982 | **1.26e-21*** | 0.15838 | | 0.66031 | | **2.14e-22*** | | 0.19677 | | | 0.90438 | | | **4.91e-23*** | |
|  | | **Pupil Response Category – Bilateral Unreactive** | | | | | | | | | | | | | | | | | | | | |
| **ICP vs. rSO_2__L** | **MSE** | 0.4336 | | 0.17497 | 0.07763 | 0.74589 | 0.34552 | 0.10764 | 1.0109 | | 0.59729 | | 0.29927 | | 1.35801 | | | 1.30332 | | | 0.56807 | |
|  | **RMSE** | 0.65848 | | 0.41829 | 0.07763 | 0.85807 | 0.58714 | 0.11947 | 1.00544 | | 0.77281 | | 0.32408 | | 1.16529 | | | 1.14162 | | | 0.56807 | |
|  | **MAE** | 0.52018 | | 0.35387 | 0.06171 | 0.68528 | 0.48849 | 0.11947 | 0.79848 | | 0.62388 | | 0.43627 | | 0.90592 | | | 0.91686 | | | 0.56807 | |
| **ICP vs. rSO_2__R** | **MSE** | 0.4336 | | 0.19621 | 0.07011 | 0.74589 | 0.36947 | 0.08472 | 1.0109 | | 0.5754 | | 0.49874 | | 1.35801 | | | 1.27386 | | | 0.67842 | |
|  | **RMSE** | 0.65848 | | 0.44279 | 0.07011 | 0.85807 | 0.60779 | 0.09290 | 1.00544 | | 0.75802 | | 0.55573 | | 1.16529 | | | 1.12596 | | | 0.67842 | |
|  | **MAE** | 0.52018 | | 0.37 | 0.05763 | 0.68528 | 0.48638 | 0.15607 | 0.79848 | | 0.60712 | | 0.67842 | | 0.90592 | | | 0.83735 | | | 0.74342 | |
| **AMP vs. rSO_2__L** | **MSE** | 0.00601 | | 0.17497 | **6.43e-05*** | 0.01197 | 0.34552 | **2.08e-05*** | 0.02179 | | 0.59729 | | **2.08e-05*** | | 0.03934 | | | 1.30332 | | | **2.08e-05*** | |
|  | **RMSE** | 0.07752 | | 0.41829 | **6.43e-05*** | 0.1094 | 0.58714 | **2.08e-05*** | 0.1476 | | 0.77281 | | **2.08e-05*** | | 0.19834 | | | 1.14162 | | | **2.08e-05*** | |
|  | **MAE** | 0.06135 | | 0.35387 | **6.43e-05*** | 0.07995 | 0.48849 | **2.08e-05*** | 0.10813 | | 0.62388 | | **2.08e-05*** | | 0.13456 | | | 0.91686 | | | **2.08e-05*** | |
| **AMP vs. rSO_2__R** | **MSE** | 0.00601 | | 0.19621 | **1.41e-05*** | 0.01197 | 0.36947 | **5.10e-06*** | 0.02179 | | 0.5754 | | **5.10e-06*** | | 0.03934 | | | 1.27386 | | | **5.10e-06*** | |
|  | **RMSE** | 0.07752 | | 0.44279 | **1.41e-05*** | 0.1094 | 0.60779 | **5.10e-06*** | 0.1476 | | 0.75802 | | **5.10e-06*** | | 0.19834 | | | 1.12596 | | | **5.10e-06*** | |
|  | **MAE** | 0.06135 | | 0.37 | **2.54e-05*** | 0.07995 | 0.48638 | **5.10e-06*** | 0.10813 | | 0.60712 | | **5.10e-06*** | | 0.13456 | | | 0.83735 | | | **5.10e-06*** | |
|  | | **Pupil Response Category – Unilateral Unreactive** | | | | | | | | | | | | | | | | | | | | |
| **ICP vs. rSO_2__L** | **MSE** | 0.46555 | | 0.1987 | **0.00334** | 0.79084 | 0.50204 | 0.06983 | 1.2609 | | 0.76976 | | 0.24935 | | 2.13144 | | | 1.49182 | | | 0.46464 | |
|  | **RMSE** | 0.6823 | | 0.44576 | **0.00334** | 0.88899 | 0.70855 | 0.06983 | 1.12279 | | 0.87736 | | 0.24935 | | 1.45949 | | | 1.21712 | | | 0.47697 | |
|  | **MAE** | 0.54258 | | 0.37987 | **0.00431** | 0.71001 | 0.56001 | 0.14638 | 0.88009 | | 0.67293 | | 0.39454 | | 1.06675 | | | 0.88885 | | | 0.59550 | |
| **ICP vs. rSO_2__R** | **MSE** | 0.46555 | | 0.16704 | **0.00121** | 0.79084 | 0.32915 | **0.00894** | 1.2609 | | 0.80422 | | **0.04201** | | 2.13144 | | | 1.3443 | | | 0.15203 | |
|  | **RMSE** | 0.6823 | | 0.4087 | **0.00121** | 0.88899 | 0.57372 | **0.00894** | 1.12279 | | 0.89678 | | **0.04201** | | 1.45949 | | | 1.15944 | | | 0.15785 | |
|  | **MAE** | 0.54258 | | 0.3392 | **0.00130** | 0.71001 | 0.46968 | **0.01760** | 0.88009 | | 0.69536 | | 0.07962 | | 1.06675 | | | 0.86186 | | | 0.16382 | |
| **AMP vs. rSO_2__L** | **MSE** | 0.00808 | | 0.1987 | **4.27e-08*** | 0.01617 | 0.50204 | **4.27e-08*** | 0.02467 | | 0.76976 | | **2.41e-08*** | | 0.03572 | | | 1.49182 | | | **1.52e-08*** | |
|  | **RMSE** | 0.0899 | | 0.44576 | **4.27e-08*** | 0.12709 | 0.70855 | **4.27e-08*** | 0.15701 | | 0.87736 | | **1.71e-08*** | | 0.18862 | | | 1.21712 | | | **1.52e-08*** | |
|  | **MAE** | 0.07164 | | 0.37987 | **4.79e-08*** | 0.09355 | 0.56001 | **5.36e-08*** | 0.11824 | | 0.67293 | | **1.71e-08*** | | 0.1463 | | | 0.88885 | | | **3.04e-08*** | |
| **AMP vs. rSO_2__R** | **MSE** | 0.00808 | | 0.16704 | **9.67e-07*** | 0.01617 | 0.32915 | **7.88e-07*** | 0.02467 | | 0.80422 | | **4.69e-07*** | | 0.03572 | | | 1.3443 | | | **2.41e-08*** | |
|  | **RMSE** | 0.0899 | | 0.4087 | **9.67e-07*** | 0.12709 | 0.57372 | **7.88e-07*** | 0.15701 | | 0.89678 | | **4.23e-07*** | | 0.18862 | | | 1.15944 | | | **2.41e-08*** | |
|  | **MAE** | 0.07164 | | 0.3392 | **1.19e-06*** | 0.09355 | 0.46968 | **7.88e-07*** | 0.11824 | | 0.69536 | | **3.42e-07*** | | 0.1463 | | | 0.86186 | | | **4.27e-08*** | |
|  | | **Rotterdam CT Grade of 1** | | | | | | | | | | | | | | | | | | | | |
| **ICP vs. rSO_2__L** | **MSE** | 0.51941 | | 0.21677 | 1.00000 | 1.01441 | 0.50527 | 1.00000 | 1.59166 | | 1.02646 | | 1.00000 | | 3.33017 | | | 2.10793 | | | 1.00000 | |
|  | **RMSE** | 0.7207 | | 0.46559 | 1.00000 | 1.00718 | 0.71083 | 1.00000 | 1.26161 | | 1.01314 | | 1.00000 | | 1.82487 | | | 1.45187 | | | 1.00000 | |
|  | **MAE** | 0.5665 | | 0.39304 | 1.00000 | 0.78246 | 0.56579 | 1.00000 | 1.02142 | | 0.79874 | | 1.00000 | | 1.26888 | | | 1.08825 | | | 1.00000 | |
| **ICP vs. rSO_2__R** | **MSE** | 0.51941 | | 0.16017 | 1.00000 | 1.01441 | 0.31647 | 1.00000 | 1.59166 | | 0.61613 | | 1.00000 | | 3.33017 | | | 1.0895 | | | 1.00000 | |
|  | **RMSE** | 0.7207 | | 0.40021 | 1.00000 | 1.00718 | 0.56256 | 1.00000 | 1.26161 | | 0.78494 | | 1.00000 | | 1.82487 | | | 1.04379 | | | 1.00000 | |
|  | **MAE** | 0.5665 | | 0.33862 | 1.00000 | 0.78246 | 0.45227 | 1.00000 | 1.02142 | | 0.63358 | | 1.00000 | | 1.26888 | | | 0.83269 | | | 1.00000 | |
| **AMP vs. rSO_2__L** | **MSE** | 0.01366 | | 0.21677 | 1.00000 | 0.03336 | 0.50527 | 1.00000 | 0.0509 | | 1.02646 | | 1.00000 | | 0.0762 | | | 2.10793 | | | 1.00000 | |
|  | **RMSE** | 0.11686 | | 0.46559 | 1.00000 | 0.18264 | 0.71083 | 1.00000 | 0.2256 | | 1.01314 | | 1.00000 | | 0.27595 | | | 1.45187 | | | 1.00000 | |
|  | **MAE** | 0.09124 | | 0.39304 | 1.00000 | 0.12628 | 0.56579 | 1.00000 | 0.17111 | | 0.79874 | | 1.00000 | | 0.19815 | | | 1.08825 | | | 1.00000 | |
| **AMP vs. rSO_2__R** | **MSE** | 0.01366 | | 0.16017 | 1.00000 | 0.03336 | 0.31647 | 1.00000 | 0.0509 | | 0.61613 | | 1.00000 | | 0.0762 | | | 1.0895 | | | 1.00000 | |
|  | **RMSE** | 0.11686 | | 0.40021 | 1.00000 | 0.18264 | 0.56256 | 1.00000 | 0.2256 | | 0.78494 | | 1.00000 | | 0.27595 | | | 1.04379 | | | 1.00000 | |
|  | **MAE** | 0.09124 | | 0.33862 | 1.00000 | 0.12628 | 0.45227 | 1.00000 | 0.17111 | | 0.63358 | | 1.00000 | | 0.19815 | | | 0.83269 | | | 1.00000 | |
|  | | **Rotterdam CT Grade of 2** | | | | | | | | | | | | | | | | | | | | |
| **ICP vs. rSO_2__L** | **MSE** | 0.5254 | | 0.23513 | **0.03064** | 1.04561 | 0.58758 | 0.06555 | 2.23 | | 1.08859 | | **0.04533** | | 3.80891 | | | 2.12177 | | | 0.29795 | |
|  | **RMSE** | 0.72451 | | 0.4847 | **0.03064** | 1.01994 | 0.76058 | 0.06555 | 1.4905 | | 1.03209 | | **0.04533** | | 1.94943 | | | 1.43925 | | | 0.29795 | |
|  | **MAE** | 0.5678 | | 0.39837 | **0.03064** | 0.79583 | 0.60642 | 0.12821 | 1.14278 | | 0.81136 | | 0.06555 | | 1.44261 | | | 1.05566 | | | 0.22977 | |
| **ICP vs. rSO_2__R** | **MSE** | 0.5254 | | 0.24422 | **0.04283** | 1.04561 | 0.57908 | 0.24095 | 2.23 | | 1.21983 | | 0.45555 | | 3.80891 | | | 2.27182 | | | 0.59403 | |
|  | **RMSE** | 0.72451 | | 0.48652 | **0.04283** | 1.01994 | 0.7231 | 0.24095 | 1.4905 | | 1.03702 | | 0.45555 | | 1.94943 | | | 1.384 | | | 0.59403 | |
|  | **MAE** | 0.5678 | | 0.40017 | **0.04283** | 0.79583 | 0.59022 | 0.24095 | 1.14278 | | 0.83011 | | 0.59403 | | 1.44261 | | | 1.15735 | | | 1.00000 | |
| **AMP vs. rSO_2__L** | **MSE** | 0.01098 | | 0.23513 | **0.00507** | 0.02199 | 0.58758 | **0.00507** | 0.03909 | | 1.08859 | | **0.00507** | | 0.0594 | | | 2.12177 | | | **0.00507** | |
|  | **RMSE** | 0.10451 | | 0.4847 | **0.00507** | 0.14816 | 0.76058 | **0.00507** | 0.19573 | | 1.03209 | | **0.00507** | | 0.24219 | | | 1.43925 | | | **0.00507** | |
|  | **MAE** | 0.07974 | | 0.39837 | **0.00507** | 0.11223 | 0.60642 | **0.00507** | 0.14228 | | 0.81136 | | **0.00507** | | 0.18535 | | | 1.05566 | | | **0.00507** | |
| **AMP vs. rSO_2__R** | **MSE** | 0.01098 | | 0.24422 | **0.01421** | 0.02199 | 0.57908 | **0.01421** | 0.03909 | | 1.21983 | | **0.01421** | | 0.0594 | | | 2.27182 | | | **0.01421** | |
|  | **RMSE** | 0.10451 | | 0.48652 | **0.01421** | 0.14816 | 0.7231 | **0.01421** | 0.19573 | | 1.03702 | | **0.01421** | | 0.24219 | | | 1.384 | | | **0.01421** | |
|  | **MAE** | 0.07974 | | 0.40017 | **0.01421** | 0.11223 | 0.59022 | **0.01421** | 0.14228 | | 0.83011 | | **0.01421** | | 0.18535 | | | 1.15735 | | | **0.01421** | |
|  | | **Rotterdam CT Grade of 3** | | | | | | | | | | | | | | | | | | | | |
| **ICP vs. rSO_2__L** | **MSE** | 0.93589 | | 0.29416 | **3.32e-06*** | 1.55632 | 0.52815 | **1.61e-05*** | 2.30457 | | 1.08049 | | **5.78e-04*** | | 3.75266 | | | 2.40872 | | | **0.00277** | |
|  | **RMSE** | 0.96709 | | 0.54236 | **3.32e-06*** | 1.24742 | 0.72674 | **2.35e-05*** | 1.51792 | | 1.03946 | | **5.78e-04*** | | 1.93421 | | | 1.552 | | | **0.00277** | |
|  | **MAE** | 0.78396 | | 0.43489 | **3.32e-06*** | 0.93609 | 0.58271 | **3.85e-05*** | 1.13036 | | 0.79365 | | **7.10e-04*** | | 1.43504 | | | 1.05176 | | | **0.00277** | |
| **ICP vs. rSO_2__R** | **MSE** | 0.93589 | | 0.25248 | **8.39e-07*** | 1.55632 | 0.50159 | **4.75e-06*** | 2.30457 | | 0.9722 | | **2.73e-05*** | | 3.75266 | | | 1.60983 | | | **1.72e-04*** | |
|  | **RMSE** | 0.96709 | | 0.50242 | **8.39e-07*** | 1.24742 | 0.70815 | **4.75e-06*** | 1.51792 | | 0.98563 | | **4.00e-05*** | | 1.93421 | | | 1.26755 | | | **1.72e-04*** | |
|  | **MAE** | 0.78396 | | 0.41107 | **1.13e-06*** | 0.93609 | 0.55085 | **5.47e-06*** | 1.13036 | | 0.76658 | | **4.00e-05*** | | 1.43504 | | | 0.94428 | | | **5.82e-05*** | |
| **AMP vs. rSO_2__L** | **MSE** | 0.03357 | | 0.29416 | **2.52e-07*** | 0.05524 | 0.52815 | **1.18e-07*** | 0.07569 | | 1.08049 | | **1.18e-07*** | | 0.11207 | | | 2.40872 | | | **1.18e-07*** | |
|  | **RMSE** | 0.18321 | | 0.54236 | **2.52e-07*** | 0.23497 | 0.72674 | **1.18e-07*** | 0.2751 | | 1.03946 | | **1.18e-07*** | | 0.3346 | | | 1.552 | | | **1.18e-07*** | |
|  | **MAE** | 0.14315 | | 0.43489 | **2.93e-07*** | 0.18471 | 0.58271 | **1.01e-07*** | 0.20566 | | 0.79365 | | **1.01e-07*** | | 0.24779 | | | 1.05176 | | | **1.01e-07*** | |
| **AMP vs. rSO_2__R** | **MSE** | 0.03357 | | 0.25248 | **1.54e-07*** | 0.05524 | 0.50159 | **1.54e-07*** | 0.07569 | | 0.9722 | | **1.54e-07*** | | 0.11207 | | | 1.60983 | | | **1.54e-07*** | |
|  | **RMSE** | 0.18321 | | 0.50242 | **1.54e-07*** | 0.23497 | 0.70815 | **1.54e-07*** | 0.2751 | | 0.98563 | | **1.54e-07*** | | 0.3346 | | | 1.26755 | | | **1.54e-07*** | |
|  | **MAE** | 0.14315 | | 0.41107 | **1.54e-07*** | 0.18471 | 0.55085 | **1.54e-07*** | 0.20566 | | 0.76658 | | **1.54e-07*** | | 0.24779 | | | 0.94428 | | | **1.54e-07*** | |
|  | | **Rotterdam CT Grade of 4** | | | | | | | | | | | | | | | | | | | | |
| **ICP vs. rSO_2__L** | **MSE** | 0.66517 | | 0.1775 | **1.86e-06*** | 1.16723 | 0.28504 | **2.29e-06*** | 2.39079 | | 0.57451 | | **3.16e-05*** | | 4.07685 | | | 1.20345 | | | **9.87e-05*** | |
|  | **RMSE** | 0.81558 | | 0.42131 | **1.86e-06*** | 1.08038 | 0.5339 | **2.13e-06*** | 1.54622 | | 0.75796 | | **3.16e-05*** | | 2.01911 | | | 1.097 | | | **9.87e-05*** | |
|  | **MAE** | 0.64498 | | 0.35995 | **3.96e-06*** | 0.84399 | 0.43789 | **3.96e-06*** | 1.15402 | | 0.59411 | | **3.57e-05*** | | 1.42397 | | | 0.83737 | | | **2.19e-04*** | |
| **ICP vs. rSO_2__R** | **MSE** | 0.66517 | | 0.20699 | **1.37e-05*** | 1.16723 | 0.36016 | **5.83e-05*** | 2.39079 | | 0.69427 | | **0.00136** | | 4.07685 | | | 1.42057 | | | **0.00874** | |
|  | **RMSE** | 0.81558 | | 0.45496 | **1.37e-05*** | 1.08038 | 0.60014 | **5.83e-05*** | 1.54622 | | 0.83323 | | **0.00136** | | 2.01911 | | | 1.19188 | | | **0.00874** | |
|  | **MAE** | 0.64498 | | 0.37671 | **1.37e-05*** | 0.84399 | 0.48208 | **9.86e-05*** | 1.15402 | | 0.6832 | | **0.00169** | | 1.42397 | | | 0.90749 | | | **0.01629** | |
| **AMP vs. rSO_2__L** | **MSE** | 0.01877 | | 0.1775 | **3.40e-09*** | 0.03351 | 0.28504 | **1.81e-08*** | 0.05543 | | 0.57451 | | **5.66e-09*** | | 0.09475 | | | 1.20345 | | | **5.38e-10*** | |
|  | **RMSE** | 0.137 | | 0.42131 | **3.40e-09*** | 0.18306 | 0.5339 | **1.81e-08*** | 0.23545 | | 0.75796 | | **5.66e-09*** | | 0.30781 | | | 1.097 | | | **5.38e-10*** | |
|  | **MAE** | 0.10575 | | 0.35995 | **3.40e-09*** | 0.14455 | 0.43789 | **1.81e-08*** | 0.17068 | | 0.59411 | | **4.03e-09*** | | 0.21471 | | | 0.83737 | | | **5.38e-10*** | |
| **AMP vs. rSO_2__R** | **MSE** | 0.01877 | | 0.20699 | **1.73e-08*** | 0.03351 | 0.36016 | **4.26e-08*** | 0.05543 | | 0.69427 | | **9.98e-09*** | | 0.09475 | | | 1.42057 | | | **1.11e-09*** | |
|  | **RMSE** | 0.137 | | 0.45496 | **1.73e-08*** | 0.18306 | 0.60014 | **4.26e-08*** | 0.23545 | | 0.83323 | | **9.98e-09*** | | 0.30781 | | | 1.19188 | | | **1.11e-09*** | |
|  | **MAE** | 0.10575 | | 0.37671 | **1.90e-08*** | 0.14455 | 0.48208 | **4.26e-08*** | 0.17068 | | 0.6832 | | **8.29e-09*** | | 0.21471 | | | 0.90749 | | | **1.49e-09*** | |
|  | | **Rotterdam CT Grade of 5** | | | | | | | | | | | | | | | | | | | | |
| **ICP vs. rSO_2__L** | **MSE** | 0.30773 | | 0.15209 | **0.00548** | 0.77845 | 0.27217 | **0.01840** | 1.10382 | | 0.6487 | | 0.13727 | | 2.04072 | | | 1.30127 | | | 0.18338 | |
|  | **RMSE** | 0.55473 | | 0.38995 | **0.00548** | 0.88179 | 0.52141 | **0.01840** | 1.05048 | | 0.80449 | | 0.13727 | | 1.42853 | | | 1.13678 | | | 0.18338 | |
|  | **MAE** | 0.45039 | | 0.32066 | **0.01197** | 0.69223 | 0.42968 | **0.02880** | 0.83151 | | 0.63753 | | 0.14569 | | 1.10832 | | | 0.85045 | | | 0.21034 | |
| **ICP vs. rSO_2__R** | **MSE** | 0.30773 | | 0.16808 | **0.00319** | 0.77845 | 0.3362 | **0.00583** | 1.10382 | | 0.63477 | | **0.03701** | | 2.04072 | | | 1.26129 | | | 0.07218 | |
|  | **RMSE** | 0.55473 | | 0.40998 | **0.00319** | 0.88179 | 0.57983 | **0.00583** | 1.05048 | | 0.79672 | | **0.03701** | | 1.42853 | | | 1.11149 | | | 0.07218 | |
|  | **MAE** | 0.45039 | | 0.34453 | **0.00479** | 0.69223 | 0.47281 | **0.01080** | 0.83151 | | 0.61373 | | **0.04841** | | 1.10832 | | | 0.85707 | | | 0.05824 | |
| **AMP vs. rSO_2__L** | **MSE** | 0.00787 | | 0.15209 | **1.69e-10*** | 0.0141 | 0.27217 | **4.17e-10*** | 0.02294 | | 0.6487 | | **2.79e-10*** | | 0.03713 | | | 1.30127 | | | **2.53e-10*** | |
|  | **RMSE** | 0.08869 | | 0.38995 | **1.69e-10*** | 0.1187 | 0.52141 | **4.17e-10*** | 0.15132 | | 0.80449 | | **1.69e-10*** | | 0.19253 | | | 1.13678 | | | **2.53e-10*** | |
|  | **MAE** | 0.06781 | | 0.32066 | **1.69e-10*** | 0.09022 | 0.42968 | **3.09e-10*** | 0.11072 | | 0.63753 | | **1.52e-10*** | | 0.1438 | | | 0.85045 | | | **3.41e-10*** | |
| **AMP vs. rSO_2__R** | **MSE** | 0.00787 | | 0.16808 | **7.58e-09*** | 0.0141 | 0.3362 | **1.11e-08*** | 0.02294 | | 0.63477 | | **1.11e-08*** | | 0.03713 | | | 1.26129 | | | **4.35e-10*** | |
|  | **RMSE** | 0.08869 | | 0.40998 | **7.58e-09*** | 0.1187 | 0.57983 | **1.11e-08*** | 0.15132 | | 0.79672 | | **8.33e-09*** | | 0.19253 | | | 1.11149 | | | **4.35e-10*** | |
|  | **MAE** | 0.06781 | | 0.34453 | **1.21e-08*** | 0.09022 | 0.47281 | **8.33e-09*** | 0.11072 | | 0.61373 | | **7.58e-09*** | | 0.1438 | | | 0.85707 | | | **7.22e-10*** | |
|  | | **Rotterdam CT Grade of 6** | | | | | | | | | | | | | | | | | | | | |
| **ICP vs. rSO_2__L** | **MSE** | 0.1471 | | 0.21468 | 0.89891 | 0.22354 | 0.47735 | 0.51264 | 0.62202 | | 0.85441 | | 0.36346 | | 1.11976 | | | 1.82092 | | | 0.23704 | |
|  | **RMSE** | 0.3835 | | 0.46331 | 0.89891 | 0.47279 | 0.68934 | 0.51264 | 0.78859 | | 0.91353 | | 0.36346 | | 1.05534 | | | 1.34625 | | | 0.23704 | |
|  | **MAE** | 0.30594 | | 0.38638 | 0.80700 | 0.36619 | 0.54779 | 0.30486 | 0.52961 | | 0.72586 | | 0.19371 | | 0.71595 | | | 0.9921 | | | 0.12986 | |
| **ICP vs. rSO_2__R** | **MSE** | 0.1471 | | 0.13797 | 0.69310 | 0.22354 | 0.31392 | 0.99268 | 0.62202 | | 0.78189 | | 0.46842 | | 1.11976 | | | 1.62913 | | | 0.33521 | |
|  | **RMSE** | 0.3835 | | 0.37141 | 0.69310 | 0.47279 | 0.56026 | 0.99268 | 0.78859 | | 0.88415 | | 0.46842 | | 1.05534 | | | 1.27337 | | | 0.33521 | |
|  | **MAE** | 0.30594 | | 0.31776 | 0.69310 | 0.36619 | 0.46924 | 0.62667 | 0.52961 | | 0.68797 | | 0.23644 | | 0.71595 | | | 0.93406 | | | 0.25129 | |
| **AMP vs. rSO_2__L** | **MSE** | 0.00293 | | 0.21468 | **7.68e-09*** | 0.00536 | 0.47735 | **4.28e-09*** | 0.01 | | 0.85441 | | **4.28e-09*** | | 0.0158 | | | 1.82092 | | | **3.80e-09*** | |
|  | **RMSE** | 0.05413 | | 0.46331 | **7.68e-09*** | 0.07322 | 0.68934 | **4.28e-09*** | 0.09987 | | 0.91353 | | **4.28e-09*** | | 0.12529 | | | 1.34625 | | | **3.80e-09*** | |
|  | **MAE** | 0.04314 | | 0.38638 | **9.68e-09*** | 0.05596 | 0.54779 | **2.66e-09*** | 0.0765 | | 0.72586 | | **3.38e-09*** | | 0.09453 | | | 0.9921 | | | **3.38e-09*** | |
| **AMP vs. rSO_2__R** | **MSE** | 0.00293 | | 0.13797 | **1.08e-08*** | 0.00536 | 0.31392 | **1.84e-09*** | 0.01 | | 0.78189 | | **1.47e-09*** | | 0.0158 | | | 1.62913 | | | **9.26e-10*** | |
|  | **RMSE** | 0.05413 | | 0.37141 | **1.08e-08*** | 0.07322 | 0.56026 | **1.84e-09*** | 0.09987 | | 0.88415 | | **1.47e-09*** | | 0.12529 | | | 1.27337 | | | **9.26e-10*** | |
|  | **MAE** | 0.04314 | | 0.31776 | **9.69e-09*** | 0.05596 | 0.46924 | **1.31e-09*** | 0.0765 | | 0.68797 | | **9.26e-10*** | | 0.09453 | | | 0.93406 | | | **9.26e-10*** | |
|  | | **Marshall CT Grade of II** | | | | | | | | | | | | | | | | | | | | |
| **ICP vs. rSO_2__L** | **MSE** | 0.55748 | | 0.24879 | 0.08086 | 1.19465 | 0.73261 | 0.19043 | 2.3911 | | 1.40416 | | 0.08086 | | 3.06251 | | | 2.86183 | | | 1.00000 | |
|  | **RMSE** | 0.74664 | | 0.49879 | 0.08086 | 1.093 | 0.85593 | 0.19043 | 1.54595 | | 1.18497 | | 0.08086 | | 1.75 | | | 1.6915 | | | 1.00000 | |
|  | **MAE** | 0.58011 | | 0.419 | 0.08086 | 0.85025 | 0.69042 | 0.19043 | 1.07107 | | 0.93488 | | 0.19043 | | 1.34779 | | | 1.33323 | | | 1.00000 | |
| **ICP vs. rSO_2__R** | **MSE** | 0.55748 | | 0.33065 | 0.08086 | 1.19465 | 0.9236 | 0.38273 | 2.3911 | | 1.45997 | | 0.19043 | | 3.06251 | | | 2.73861 | | | 1.00000 | |
|  | **RMSE** | 0.74664 | | 0.57502 | 0.08086 | 1.093 | 0.96104 | 0.38273 | 1.54595 | | 1.20829 | | 0.19043 | | 1.75 | | | 1.65485 | | | 1.00000 | |
|  | **MAE** | 0.58011 | | 0.47107 | 0.08086 | 0.85025 | 0.77221 | 0.38273 | 1.07107 | | 1.01694 | | 0.66252 | | 1.34779 | | | 1.321 | | | 1.00000 | |
| **AMP vs. rSO_2__L** | **MSE** | 0.00599 | | 0.24879 | 0.08086 | 0.01522 | 0.73261 | 0.08086 | 0.02144 | | 1.40416 | | 0.08086 | | 0.03117 | | | 2.86183 | | | 0.08086 | |
|  | **RMSE** | 0.07741 | | 0.49879 | 0.08086 | 0.12337 | 0.85593 | 0.08086 | 0.14643 | | 1.18497 | | 0.08086 | | 0.17656 | | | 1.6915 | | | 0.08086 | |
|  | **MAE** | 0.06137 | | 0.419 | 0.08086 | 0.09684 | 0.69042 | 0.08086 | 0.0985 | | 0.93488 | | 0.08086 | | 0.13026 | | | 1.33323 | | | 0.08086 | |
| **AMP vs. rSO_2__R** | **MSE** | 0.00599 | | 0.33065 | 0.08086 | 0.01522 | 0.9236 | 0.08086 | 0.02144 | | 1.45997 | | 0.08086 | | 0.03117 | | | 2.73861 | | | 0.08086 | |
|  | **RMSE** | 0.07741 | | 0.57502 | 0.08086 | 0.12337 | 0.96104 | 0.08086 | 0.14643 | | 1.20829 | | 0.08086 | | 0.17656 | | | 1.65485 | | | 0.08086 | |
|  | **MAE** | 0.06137 | | 0.47107 | 0.08086 | 0.09684 | 0.77221 | 0.08086 | 0.0985 | | 1.01694 | | 0.08086 | | 0.13026 | | | 1.321 | | | 0.08086 | |
|  | | **Marshall CT Grade of III** | | | | | | | | | | | | | | | | | | | | |
| **ICP vs. rSO_2__L** | **MSE** | 0.74608 | | 0.2225 | **1.54e-09*** | 1.25997 | 0.48394 | **4.87e-08*** | 2.15437 | | 0.81445 | | **6.33e-06*** | | 4.29129 | | | 1.98239 | | | **4.25e-05*** | |
|  | **RMSE** | 0.86373 | | 0.47166 | **1.54e-09*** | 1.12248 | 0.69553 | **5.84e-08*** | 1.46751 | | 0.90245 | | **6.66e-06*** | | 2.07134 | | | 1.40659 | | | **4.67e-05*** | |
|  | **MAE** | 0.68461 | | 0.39312 | **2.15e-09*** | 0.86432 | 0.5432 | **1.00e-07*** | 1.14155 | | 0.72098 | | **8.15e-06*** | | 1.45262 | | | 1.05164 | | | **4.89e-05*** | |
| **ICP vs. rSO_2__R** | **MSE** | 0.74608 | | 0.24771 | **3.14e-09*** | 1.25997 | 0.50873 | **2.07e-07*** | 2.15437 | | 1.04738 | | **3.41e-05*** | | 4.29129 | | | 2.14788 | | | **3.62e-04*** | |
|  | **RMSE** | 0.86373 | | 0.4977 | **3.14e-09*** | 1.12248 | 0.71325 | **2.07e-07*** | 1.46751 | | 1.02335 | | **3.98e-05*** | | 2.07134 | | | 1.46173 | | | **3.62e-04*** | |
|  | **MAE** | 0.68461 | | 0.40827 | **3.62e-09*** | 0.86432 | 0.55085 | **3.63e-07*** | 1.14155 | | 0.77187 | | **4.41e-05*** | | 1.45262 | | | 1.05404 | | | **2.19e-04*** | |
| **AMP vs. rSO_2__L** | **MSE** | 0.02067 | | 0.2225 | **3.25e-13*** | 0.04126 | 0.48394 | **1.45e-13*** | 0.05981 | | 0.81445 | | **1.45e-13*** | | 0.09034 | | | 1.98239 | | | **1.45e-13*** | |
|  | **RMSE** | 0.14336 | | 0.47166 | **3.25e-13*** | 0.20302 | 0.69553 | **1.45e-13*** | 0.24451 | | 0.90245 | | **1.45e-13*** | | 0.30048 | | | 1.40659 | | | **1.45e-13*** | |
|  | **MAE** | 0.11191 | | 0.39312 | **4.47e-13*** | 0.14856 | 0.5432 | **1.45e-13*** | 0.18174 | | 0.72098 | | **1.45e-13*** | | 0.22197 | | | 1.05164 | | | **1.45e-13*** | |
| **AMP vs. rSO_2__R** | **MSE** | 0.02067 | | 0.24771 | **8.66e-13*** | 0.04126 | 0.50873 | **7.94e-13*** | 0.05981 | | 1.04738 | | **7.94e-13*** | | 0.09034 | | | 2.14788 | | | **7.94e-13*** | |
|  | **RMSE** | 0.14336 | | 0.4977 | **8.66e-13*** | 0.20302 | 0.71325 | **7.94e-13*** | 0.24451 | | 1.02335 | | **7.94e-13*** | | 0.30048 | | | 1.46173 | | | **7.94e-13*** | |
|  | **MAE** | 0.11191 | | 0.40827 | **8.66e-13*** | 0.14856 | 0.55085 | **7.94e-13*** | 0.18174 | | 0.77187 | | **7.94e-13*** | | 0.22197 | | | 1.05404 | | | **7.94e-13*** | |
|  | | **Marshall CT Grade of IV** | | | | | | | | | | | | | | | | | | | | |
| **ICP vs. rSO_2__L** | **MSE** | 0.69758 | | 0.15238 | **0.00254** | 1.23539 | 0.24951 | **0.00361** | 2.6187 | | 0.55534 | | **0.00634** | | 4.18554 | | | 1.34367 | | | **0.01198** | |
|  | **RMSE** | 0.83499 | | 0.39036 | **0.00254** | 1.11132 | 0.49951 | **0.00361** | 1.61774 | | 0.74521 | | **0.00634** | | 2.03069 | | | 1.15157 | | | **0.01198** | |
|  | **MAE** | 0.65109 | | 0.32793 | **0.00286** | 0.89378 | 0.42587 | **0.00454** | 1.23061 | | 0.59411 | | **0.00707** | | 1.45954 | | | 0.86718 | | | **0.01198** | |
| **ICP vs. rSO_2__R** | **MSE** | 0.69758 | | 0.16269 | **0.00370** | 1.23539 | 0.3409 | **0.00540** | 2.6187 | | 0.67573 | | **0.00874** | | 4.18554 | | | 1.26129 | | | **0.01102** | |
|  | **RMSE** | 0.83499 | | 0.40334 | **0.00370** | 1.11132 | 0.58386 | **0.00540** | 1.61774 | | 0.8219 | | **0.00874** | | 2.03069 | | | 1.12307 | | | **0.01102** | |
|  | **MAE** | 0.65109 | | 0.33687 | **0.00421** | 0.89378 | 0.47953 | **0.00776** | 1.23061 | | 0.61379 | | **0.00982** | | 1.45954 | | | 0.85751 | | | **0.01544** | |
| **AMP vs. rSO_2__L** | **MSE** | 0.01612 | | 0.15238 | **2.97e-05*** | 0.03168 | 0.24951 | **2.43e-04*** | 0.05377 | | 0.55534 | | **1.02e-04*** | | 0.09266 | | | 1.34367 | | | **1.83e-05*** | |
|  | **RMSE** | 0.12652 | | 0.39036 | **2.97e-05*** | 0.17793 | 0.49951 | **2.43e-04*** | 0.2318 | | 0.74521 | | **1.02e-04*** | | 0.3023 | | | 1.15157 | | | **1.83e-05*** | |
|  | **MAE** | 0.0995 | | 0.32793 | **2.53e-05*** | 0.13871 | 0.42587 | **2.11e-04*** | 0.17862 | | 0.59411 | | **7.53e-05*** | | 0.23712 | | | 0.86718 | | | **1.32e-05*** | |
| **AMP vs. rSO_2__R** | **MSE** | 0.01612 | | 0.16269 | **1.12e-04*** | 0.03168 | 0.3409 | **3.96e-04*** | 0.05377 | | 0.67573 | | **2.49e-04*** | | 0.09266 | | | 1.26129 | | | **5.74e-05*** | |
|  | **RMSE** | 0.12652 | | 0.40334 | **1.12e-04*** | 0.17793 | 0.58386 | **3.96e-04*** | 0.2318 | | 0.8219 | | **2.49e-04*** | | 0.3023 | | | 1.12307 | | | **5.74e-05*** | |
|  | **MAE** | 0.0995 | | 0.33687 | **2.49e-04*** | 0.13871 | 0.47953 | **5.35e-04*** | 0.17862 | | 0.61379 | | **2.13e-04*** | | 0.23712 | | | 0.85751 | | | **4.84e-05*** | |
|  | | **Marshall CT Grade of V** | | | | | | | | | | | | | | | | | | | | |
| **ICP vs. rSO_2__L** | **MSE** | 0.27771 | | 0.19634 | 0.05586 | 0.54492 | 0.3771 | 0.16801 | 0.89491 | | 0.74282 | | 0.55291 | | 1.51328 | | | 1.41275 | | | 0.81517 | |
|  | **RMSE** | 0.52698 | | 0.4431 | 0.05586 | 0.73819 | 0.61408 | 0.16074 | 0.946 | | 0.86186 | | 0.55291 | | 1.23016 | | | 1.18859 | | | 0.81982 | |
|  | **MAE** | 0.42808 | | 0.37633 | 0.07905 | 0.57178 | 0.50212 | 0.40475 | 0.73994 | | 0.66543 | | 0.85729 | | 0.90323 | | | 0.87308 | | | 0.87143 | |
| **ICP vs. rSO_2__R** | **MSE** | 0.27771 | | 0.14529 | **0.00594** | 0.54492 | 0.32035 | **0.01819** | 0.89491 | | 0.57796 | | 0.13873 | | 1.51328 | | | 1.27076 | | | 0.29420 | |
|  | **RMSE** | 0.52698 | | 0.38117 | **0.00594** | 0.73819 | 0.56599 | **0.01819** | 0.946 | | 0.76023 | | 0.13714 | | 1.23016 | | | 1.11149 | | | 0.29975 | |
|  | **MAE** | 0.42808 | | 0.31702 | **0.00821** | 0.57178 | 0.46881 | 0.06752 | 0.73994 | | 0.60259 | | 0.28872 | | 0.90323 | | | 0.8544 | | | 0.45733 | |
| **AMP vs. rSO_2__L** | **MSE** | 0.00648 | | 0.19634 | **2.22e-17*** | 0.00976 | 0.3771 | **1.02e-17*** | 0.02296 | | 0.74282 | | **4.66e-18*** | | 0.03405 | | | 1.41275 | | | **2.90e-18*** | |
|  | **RMSE** | 0.08049 | | 0.4431 | **2.22e-17*** | 0.09876 | 0.61408 | **1.02e-17*** | 0.15151 | | 0.86186 | | **3.40e-18*** | | 0.18453 | | | 1.18859 | | | **2.90e-18*** | |
|  | **MAE** | 0.05752 | | 0.37633 | **3.91e-17*** | 0.0774 | 0.50212 | **9.21e-18*** | 0.097 | | 0.66543 | | **2.61e-18*** | | 0.13456 | | | 0.87308 | | | **2.75e-18*** | |
| **AMP vs. rSO_2__R** | **MSE** | 0.00648 | | 0.14529 | **1.29e-15*** | 0.00976 | 0.32035 | **3.41e-16*** | 0.02296 | | 0.57796 | | **1.32e-16*** | | 0.03405 | | | 1.27076 | | | **2.00e-18*** | |
|  | **RMSE** | 0.08049 | | 0.38117 | **1.29e-15*** | 0.09876 | 0.56599 | **3.41e-16*** | 0.15151 | | 0.76023 | | **9.76e-17*** | | 0.18453 | | | 1.11149 | | | **2.00e-18*** | |
|  | **MAE** | 0.05752 | | 0.31702 | **1.80e-15*** | 0.0774 | 0.46881 | **2.29e-16*** | 0.097 | | 0.60259 | | **7.58e-17*** | | 0.13456 | | | 0.8544 | | | **4.20e-18*** | |
|  | | **Under 40** | | | | | | | | | | | | | | | | | | | | |
| **ICP vs. rSO_2__L** | **MSE** | 0.60793 | | 0.21155 | **1.42e-05*** | 1.14629 | 0.4542 | **5.50e-04*** | 1.79854 | | 1.03066 | | **0.00658** | | 2.73602 | | | 2.10688 | | | **0.03975** | |
|  | **RMSE** | 0.7797 | | 0.45987 | **1.42e-05*** | 1.07065 | 0.67387 | **5.77e-04*** | 1.34109 | | 1.01492 | | **0.00671** | | 1.65395 | | | 1.45124 | | | **0.03975** | |
|  | **MAE** | 0.61867 | | 0.37747 | **1.96e-05*** | 0.8186 | 0.52561 | **0.00129** | 1.03045 | | 0.77324 | | **0.01268** | | 1.31215 | | | 1.03765 | | | **0.02939** | |
| **ICP vs. rSO_2__R** | **MSE** | 0.60793 | | 0.22363 | **1.60e-05*** | 1.14629 | 0.47836 | **6.83e-04*** | 1.79854 | | 0.99611 | | **0.01239** | | 2.73602 | | | 2.09334 | | | **0.04808** | |
|  | **RMSE** | 0.7797 | | 0.47283 | **1.60e-05*** | 1.07065 | 0.69144 | **7.01e-04*** | 1.34109 | | 0.99714 | | **0.01317** | | 1.65395 | | | 1.4441 | | | **0.04726** | |
|  | **MAE** | 0.61867 | | 0.38849 | **1.60e-05*** | 0.8186 | 0.53818 | **0.00124** | 1.03045 | | 0.77866 | | **0.01488** | | 1.31215 | | | 1.07422 | | | **0.02713** | |
| **AMP vs. rSO_2__L** | **MSE** | 0.01323 | | 0.21155 | **2.29e-18*** | 0.02141 | 0.4542 | **1.45e-18*** | 0.03541 | | 1.03066 | | **1.37e-18*** | | 0.06234 | | | 2.10688 | | | **1.16e-18*** | |
|  | **RMSE** | 0.115 | | 0.45987 | **2.29e-18*** | 0.1463 | 0.67387 | **1.45e-18*** | 0.18818 | | 1.01492 | | **1.37e-18*** | | 0.24967 | | | 1.45124 | | | **1.16e-18*** | |
|  | **MAE** | 0.09188 | | 0.37747 | **2.05e-18*** | 0.11527 | 0.52561 | **1.30e-18*** | 0.14492 | | 0.77324 | | **1.16e-18*** | | 0.17889 | | | 1.03765 | | | **1.16e-18*** | |
| **AMP vs. rSO_2__R** | **MSE** | 0.01323 | | 0.22363 | **9.50e-17*** | 0.02141 | 0.47836 | **6.17e-17*** | 0.03541 | | 0.99611 | | **5.13e-17*** | | 0.06234 | | | 2.09334 | | | **4.26e-17*** | |
|  | **RMSE** | 0.115 | | 0.47283 | **9.50e-17*** | 0.1463 | 0.69144 | **6.17e-17*** | 0.18818 | | 0.99714 | | **5.13e-17*** | | 0.24967 | | | 1.4441 | | | **4.26e-17*** | |
|  | **MAE** | 0.09188 | | 0.38849 | **1.14e-16*** | 0.11527 | 0.53818 | **5.13e-17*** | 0.14492 | | 0.77866 | | **4.82e-17*** | | 0.17889 | | | 1.07422 | | | **4.26e-17*** | |
|  | | **40 - 60** | | | | | | | | | | | | | | | | | | | | |
| **ICP vs. rSO_2__L** | **MSE** | 0.51732 | | 0.21391 | **6.82e-04*** | 0.92339 | 0.45766 | **0.00124** | 1.60248 | | 0.76256 | | **0.01696** | | 2.67297 | | | 1.54757 | | | **0.04868** | |
|  | **RMSE** | 0.71924 | | 0.4625 | **6.82e-04*** | 0.9609 | 0.67571 | **0.00124** | 1.26588 | | 0.87324 | | **0.01696** | | 1.63485 | | | 1.23608 | | | **0.04988** | |
|  | **MAE** | 0.56063 | | 0.39312 | **8.24e-04*** | 0.75257 | 0.53668 | **0.00474** | 0.98367 | | 0.66958 | | **0.02779** | | 1.19661 | | | 0.90286 | | | 0.12749 | |
| **ICP vs. rSO_2__R** | **MSE** | 0.51732 | | 0.16 | **6.91e-05*** | 0.92339 | 0.32253 | **2.88e-04*** | 1.60248 | | 0.70296 | | **0.00458** | | 2.67297 | | | 1.30753 | | | **0.01514** | |
|  | **RMSE** | 0.71924 | | 0.40001 | **6.91e-05*** | 0.9609 | 0.56789 | **2.88e-04*** | 1.26588 | | 0.83821 | | **0.00444** | | 1.63485 | | | 1.14126 | | | **0.01649** | |
|  | **MAE** | 0.56063 | | 0.3356 | **1.32e-04*** | 0.75257 | 0.45963 | **0.00111** | 0.98367 | | 0.64768 | | **0.01034** | | 1.19661 | | | 0.86098 | | | 0.06041 | |
| **AMP vs. rSO_2__L** | **MSE** | 0.01047 | | 0.21391 | **5.41e-13*** | 0.01904 | 0.45766 | **2.32e-13*** | 0.03206 | | 0.76256 | | **2.32e-13*** | | 0.05654 | | | 1.54757 | | | **2.32e-13*** | |
|  | **RMSE** | 0.10218 | | 0.4625 | **5.41e-13*** | 0.13793 | 0.67571 | **2.32e-13*** | 0.17904 | | 0.87324 | | **2.32e-13*** | | 0.23648 | | | 1.23608 | | | **2.32e-13*** | |
|  | **MAE** | 0.07494 | | 0.39312 | **7.92e-13*** | 0.09665 | 0.53668 | **1.84e-13*** | 0.11927 | | 0.66958 | | **1.24e-13*** | | 0.1438 | | | 0.90286 | | | **1.24e-13*** | |
| **AMP vs. rSO_2__R** | **MSE** | 0.01047 | | 0.16 | **1.75e-11*** | 0.01904 | 0.32253 | **3.81e-12*** | 0.03206 | | 0.70296 | | **2.63e-12*** | | 0.05654 | | | 1.30753 | | | **9.07e-14*** | |
|  | **RMSE** | 0.10218 | | 0.40001 | **1.75e-11*** | 0.13793 | 0.56789 | **3.81e-12*** | 0.17904 | | 0.83821 | | **2.63e-12*** | | 0.23648 | | | 1.14126 | | | **9.07e-14*** | |
|  | **MAE** | 0.07494 | | 0.3356 | **2.17e-11*** | 0.09665 | 0.45963 | **2.44e-12*** | 0.11927 | | 0.64768 | | **2.27e-12*** | | 0.1438 | | | 0.86098 | | | **1.84e-13*** | |
|  | | **Over 60** | | | | | | | | | | | | | | | | | | | | |
| **ICP vs. rSO_2__L** | **MSE** | 0.32763 | | 0.14287 | **0.00859** | 0.74589 | 0.2899 | **0.00859** | 1.0109 | | 0.55534 | | **0.01877** | | 1.9995 | | | 1.08119 | | | **0.02147** | |
|  | **RMSE** | 0.57239 | | 0.37798 | **0.00859** | 0.85807 | 0.53843 | **0.00797** | 1.00544 | | 0.74521 | | **0.01877** | | 1.414 | | | 1.03682 | | | **0.02147** | |
|  | **MAE** | 0.44971 | | 0.30963 | **0.00995** | 0.68528 | 0.43271 | **0.00995** | 0.79848 | | 0.60663 | | **0.02976** | | 1.06669 | | | 0.80062 | | | **0.04589** | |
| **ICP vs. rSO_2__R** | **MSE** | 0.32763 | | 0.14213 | **0.00689** | 0.74589 | 0.3175 | **0.00481** | 1.0109 | | 0.47517 | | **0.01275** | | 1.9995 | | | 0.87136 | | | **0.01764** | |
|  | **RMSE** | 0.57239 | | 0.377 | **0.00689** | 0.85807 | 0.56347 | **0.00481** | 1.00544 | | 0.68933 | | **0.01275** | | 1.414 | | | 0.93347 | | | **0.01764** | |
|  | **MAE** | 0.44971 | | 0.30118 | **0.00850** | 0.68528 | 0.46061 | **0.01043** | 0.79848 | | 0.56683 | | **0.03445** | | 1.06669 | | | 0.69125 | | | **0.02130** | |
| **AMP vs. rSO_2__L** | **MSE** | 0.01493 | | 0.14287 | **1.38e-05*** | 0.0353 | 0.2899 | **1.10e-04*** | 0.06809 | | 0.55534 | | **4.24e-05*** | | 0.09638 | | | 1.08119 | | | **1.10e-05*** | |
|  | **RMSE** | 0.12217 | | 0.37798 | **1.38e-05*** | 0.18789 | 0.53843 | **1.10e-04*** | 0.26095 | | 0.74521 | | **3.05e-05*** | | 0.30975 | | | 1.03682 | | | **1.10e-05*** | |
|  | **MAE** | 0.09632 | | 0.30963 | **1.74e-05*** | 0.13813 | 0.43271 | **1.22e-04*** | 0.17733 | | 0.60663 | | **2.73e-05*** | | 0.24343 | | | 0.80062 | | | **8.68e-06*** | |
| **AMP vs. rSO_2__R** | **MSE** | 0.01493 | | 0.14213 | **1.38e-05*** | 0.0353 | 0.3175 | **4.80e-05*** | 0.06809 | | 0.47517 | | **1.90e-05*** | | 0.09638 | | | 0.87136 | | | **4.12e-06*** | |
|  | **RMSE** | 0.12217 | | 0.377 | **1.38e-05*** | 0.18789 | 0.56347 | **4.80e-05*** | 0.26095 | | 0.68933 | | **1.54e-05*** | | 0.30975 | | | 0.93347 | | | **4.12e-06*** | |
|  | **MAE** | 0.09632 | | 0.30118 | **2.11e-05*** | 0.13813 | 0.46061 | **4.80e-05*** | 0.17733 | | 0.56683 | | **8.03e-06*** | | 0.24343 | | | 0.69125 | | | **7.19e-06*** | |
|  | | **Male** | | | | | | | | | | | | | | | | | | | | |
| **ICP vs. rSO_2__L** | **MSE** | 0.52131 | | 0.19341 | **2.16e-08*** | 0.95018 | 0.41101 | **1.05e-06*** | 1.61645 | | 0.74909 | | **1.31e-04*** | | 2.70727 | | | 1.60892 | | | **0.00114** | |
|  | **RMSE** | 0.72201 | | 0.43974 | **2.16e-08*** | 0.97225 | 0.64091 | **1.16e-06*** | 1.27139 | | 0.86549 | | **1.36e-04*** | | 1.64527 | | | 1.26064 | | | **0.00123** | |
|  | **MAE** | 0.57304 | | 0.37677 | **5.46e-08*** | 0.77178 | 0.50883 | **5.52e-06*** | 0.99807 | | 0.66958 | | **3.89e-04*** | | 1.26224 | | | 0.9277 | | | **0.00250** | |
| **ICP vs. rSO_2__R** | **MSE** | 0.52131 | | 0.17581 | **5.30e-09*** | 0.95018 | 0.36415 | **7.17e-07*** | 1.61645 | | 0.74488 | | **2.93e-04*** | | 2.70727 | | | 1.57786 | | | **0.00272** | |
|  | **RMSE** | 0.72201 | | 0.41928 | **5.30e-09*** | 0.97225 | 0.60344 | **7.53e-07*** | 1.27139 | | 0.86302 | | **3.04e-04*** | | 1.64527 | | | 1.25606 | | | **0.00283** | |
|  | **MAE** | 0.57304 | | 0.36114 | **1.70e-08*** | 0.77178 | 0.48368 | **4.30e-06*** | 0.99807 | | 0.68504 | | **5.69e-04*** | | 1.26224 | | | 0.93618 | | | **0.00411** | |
| **AMP vs. rSO_2__L** | **MSE** | 0.01326 | | 0.19341 | **3.21e-28*** | 0.02289 | 0.41101 | **2.14e-27*** | 0.04104 | | 0.74909 | | **5.56e-28*** | | 0.06433 | | | 1.60892 | | | **1.10e-28*** | |
|  | **RMSE** | 0.11516 | | 0.43974 | **3.21e-28*** | 0.15124 | 0.64091 | **2.07e-27*** | 0.20255 | | 0.86549 | | **4.16e-28*** | | 0.24831 | | | 1.26064 | | | **1.10e-28*** | |
|  | **MAE** | 0.09156 | | 0.37677 | **3.21e-28*** | 0.12145 | 0.50883 | **1.41e-27*** | 0.14459 | | 0.66958 | | **1.25e-28*** | | 0.17797 | | | 0.9277 | | | **1.03e-28*** | |
| **AMP vs. rSO_2__R** | **MSE** | 0.01326 | | 0.17581 | **3.31e-25*** | 0.02289 | 0.36415 | **9.71e-25*** | 0.04104 | | 0.74488 | | **1.60e-25*** | | 0.06433 | | | 1.57786 | | | **1.25e-27*** | |
|  | **RMSE** | 0.11516 | | 0.41928 | **3.31e-25*** | 0.15124 | 0.60344 | **9.10e-25*** | 0.20255 | | 0.86302 | | **1.45e-25*** | | 0.24831 | | | 1.25606 | | | **1.25e-27*** | |
|  | **MAE** | 0.09156 | | 0.36114 | **4.44e-25*** | 0.12145 | 0.48368 | **5.40e-25*** | 0.14459 | | 0.68504 | | **8.00e-26*** | | 0.17797 | | | 0.93618 | | | **2.56e-27*** | |
|  | | **Female** | | | | | | | | | | | | | | | | | | | | |
| **ICP vs. rSO_2__L** | **MSE** | 0.45002 | | 0.19052 | **0.01854** | 0.96123 | 0.38836 | **0.04651** | 1.59695 | | 0.75733 | | 0.07787 | | 2.44163 | | | 1.36066 | | | 0.15505 | |
|  | **RMSE** | 0.6708 | | 0.43648 | **0.01854** | 0.98033 | 0.62318 | **0.04173** | 1.26211 | | 0.87025 | | 0.07787 | | 1.56195 | | | 1.16646 | | | 0.15505 | |
|  | **MAE** | 0.53683 | | 0.3704 | **0.01970** | 0.75035 | 0.49831 | 0.05747 | 0.94632 | | 0.66543 | | 0.14855 | | 1.22399 | | | 0.92969 | | | 0.20671 | |
| **ICP vs. rSO_2__R** | **MSE** | 0.45002 | | 0.15984 | **0.00913** | 0.96123 | 0.32035 | **0.01034** | 1.59695 | | 0.47937 | | **0.01482** | | 2.44163 | | | 0.87667 | | | **0.02342** | |
|  | **RMSE** | 0.6708 | | 0.3998 | **0.00913** | 0.98033 | 0.56599 | **0.01034** | 1.26211 | | 0.69236 | | **0.01482** | | 1.56195 | | | 0.93621 | | | **0.02342** | |
|  | **MAE** | 0.53683 | | 0.33433 | **0.00624** | 0.75035 | 0.45985 | **0.01979** | 0.94632 | | 0.5422 | | **0.04001** | | 1.22399 | | | 0.7412 | | | **0.04212** | |
| **AMP vs. rSO_2__L** | **MSE** | 0.00934 | | 0.19052 | **3.26e-07*** | 0.01632 | 0.38836 | **6.54e-08*** | 0.02741 | | 0.75733 | | **5.07e-08*** | | 0.04387 | | | 1.36066 | | | **2.66e-08*** | |
|  | **RMSE** | 0.09598 | | 0.43648 | **3.26e-07*** | 0.12766 | 0.62318 | **6.54e-08*** | 0.1652 | | 0.87025 | | **5.07e-08*** | | 0.20939 | | | 1.16646 | | | **2.66e-08*** | |
|  | **MAE** | 0.07297 | | 0.3704 | **4.67e-07*** | 0.09451 | 0.49831 | **5.76e-08*** | 0.12164 | | 0.66543 | | **4.46e-08*** | | 0.15 | | | 0.92969 | | | **3.03e-08*** | |
| **AMP vs. rSO_2__R** | **MSE** | 0.00934 | | 0.15984 | **1.96e-07*** | 0.01632 | 0.32035 | **4.80e-08*** | 0.02741 | | 0.47937 | | **3.34e-08*** | | 0.04387 | | | 0.87667 | | | **1.25e-08*** | |
|  | **RMSE** | 0.09598 | | 0.3998 | **1.96e-07*** | 0.12766 | 0.56599 | **4.80e-08*** | 0.1652 | | 0.69236 | | **3.34e-08*** | | 0.20939 | | | 0.93621 | | | **1.25e-08*** | |
|  | **MAE** | 0.07297 | | 0.33433 | **6.72e-07*** | 0.09451 | 0.45985 | **4.80e-08*** | 0.12164 | | 0.5422 | | **2.62e-08*** | | 0.15 | | | 0.7412 | | | **1.81e-08*** | |
| **Physiologic** | **Variance Metric** | **Wilcoxon Signed Rank Test Results – 1 Minute Sampling Frequency** | | | | | | | | | | | | | | | | | | | | |
| **Variable Comparison** |  | **Total Population** | | | | | | | | | | | | | | | | | | | | |
|  |  | **5 Min Window** | | | | **15 Min Window** | | | **30 Min Window** | | | | | | | **1 Hour Window** | | | | | | |
|  |  | **Median ICP/AMP (IQR)** | **Median rSO2 (IQR)** | | **p-value (IQR)** | **Median ICP/AMP (IQR)** | **Median rSO2 (IQR)** | **p-value (IQR)** | **Median ICP/AMP (IQR)** | | **Median rSO2 (IQR)** | | **p-value (IQR)** | | | **Median ICP/AMP (IQR)** | | | **Median rSO2 (IQR)** | | | **p-value (IQR)** |
| **ICP vs. rSO2_L** | **MSE** | 0.115598881 | 0.064903333 | | **1.38E-05*** | 0.403432219 | 0.227976469 | **3.69E-04*** | 0.881415067 | | 0.563578508 | | **0.0016** | | | 1.788570113 | | | 1.265273037 | | | **0.0053** |
|  | **RMSE** | 0.33996753 | 0.254761162 | | **2.98E-05*** | 0.635001768 | 0.477468815 | **0.0012** | 0.93883044 | | 0.750693861 | | **0.0052** | | | 1.337337305 | | | 1.118554925 | | | **0.0085** |
|  | **MAE** | 0.2894 | 0.213466667 | | **3.02E-05*** | 0.50602037 | 0.388792593 | **0.0018** | 0.749219333 | | 0.61295 | | **0.0073** | | | 1.027667978 | | | 0.875813889 | | | **0.0221** |
| **ICP vs. rSO2_R** | **MSE** | 0.115598881 | 0.059072222 | | **1.90E-07*** | 0.403432219 | 0.211450877 | **2.33E-05*** | 0.881415067 | | 0.52773013 | | **9.95E-04*** | | | 1.788570113 | | | 1.24852304 | | | **0.0029** |
|  | **RMSE** | 0.33996753 | 0.243046574 | | **5.43E-07*** | 0.635001768 | 0.459831689 | **8.01E-05*** | 0.93883044 | | 0.726386879 | | **0.0015** | | | 1.337337305 | | | 1.107508273 | | | **0.0026** |
|  | **MAE** | 0.2894 | 0.207233333 | | **5.13E-07*** | 0.50602037 | 0.387725926 | **2.07E-04*** | 0.749219333 | | 0.592214815 | | **0.0032** | | | 1.027667978 | | | 0.833421852 | | | **0.0032** |
| **AMP vs. rSO2_L** | **MSE** | 0.002558817 | 0.064903333 | | **3.27E-18*** | 0.009273641 | 0.227976469 | **4.33E-18*** | 0.018179246 | | 0.563578508 | | **1.11E-18*** | | | 0.033599309 | | | 1.265273037 | | | **1.08E-18*** |
|  | **RMSE** | 0.050579466 | 0.254761162 | | **1.14E-18*** | 0.096296228 | 0.477468815 | **8.60E-19*** | 0.134830096 | | 0.750693861 | | **7.03E-19*** | | | 0.183293274 | | | 1.118554925 | | | **6.84E-19*** |
|  | **MAE** | 0.042623667 | 0.213466667 | | **1.18E-18*** | 0.078132074 | 0.388792593 | **1.52E-18*** | 0.106796454 | | 0.61295 | | **7.03E-19*** | | | 0.139875844 | | | 0.875813889 | | | **8.36E-19*** |
| **AMP vs. rSO2_R** | **MSE** | 0.002558817 | 0.059072222 | | **2.55E-17*** | 0.009273641 | 0.211450877 | **1.37E-16*** | 0.018179246 | | 0.52773013 | | **3.78E-17*** | | | 0.033599309 | | | 1.24852304 | | | **2.33E-17*** |
|  | **RMSE** | 0.050579466 | 0.243046574 | | **1.43E-17*** | 0.096296228 | 0.459831689 | **4.81E-17*** | 0.134830096 | | 0.726386879 | | **1.19E-17*** | | | 0.183293274 | | | 1.107508273 | | | **1.12E-17*** |
|  | **MAE** | 0.042623667 | 0.207233333 | | **1.38E-17*** | 0.078132074 | 0.387725926 | **6.50E-17*** | 0.106796454 | | 0.592214815 | | **1.12E-17*** | | | 0.139875844 | | | 0.833421852 | | | **1.71E-17*** |
|  | | **ICP Monitor Same Side as NIRS** | | | | | | | | | | | | | | | | | | | | |
| **ICP vs. rSO2_L** | **MSE** | 0.11871 | 0.06212 | | **2.18e-04*** | 0.41908 | 0.20025 | **0.00724** | 0.88805 | | 0.5517 | | **0.01772** | | | 1.79385 | | | 1.23066 | | | **0.02132** |
|  | **RMSE** | 0.34455 | 0.24925 | | **2.32e-04*** | 0.64736 | 0.44744 | **0.00705** | 0.94236 | | 0.74275 | | **0.01783** | | | 1.33935 | | | 1.10767 | | | **0.02209** |
|  | **MAE** | 0.29387 | 0.21243 | | **2.49e-04*** | 0.50916 | 0.37939 | **0.00811** | 0.74953 | | 0.59426 | | **0.02524** | | | 1.04058 | | | 0.84802 | | | **0.04606** |
| **ICP vs. rSO2_R** | **MSE** | 0.11871 | 0.05907 | | **2.62e-05*** | 0.41908 | 0.21145 | **9.07e-04*** | 0.88805 | | 0.52773 | | **0.00787** | | | 1.79385 | | | 1.24852 | | | **0.01175** |
|  | **RMSE** | 0.34455 | 0.24305 | | **2.76e-05*** | 0.64736 | 0.45983 | **9.92e-04*** | 0.94236 | | 0.72639 | | **0.00787** | | | 1.33935 | | | 1.10751 | | | **0.01197** |
|  | **MAE** | 0.29387 | 0.20723 | | **2.76e-05*** | 0.50916 | 0.38773 | **0.00223** | 0.74953 | | 0.59221 | | **0.01351** | | | 1.04058 | | | 0.83342 | | | **0.01660** |
| **AMP vs. rSO2_L** | **MSE** | 0.00248 | 0.06212 | | **2.97e-33*** | 0.00926 | 0.20025 | **4.70e-33*** | 0.01816 | | 0.5517 | | **9.04e-34*** | | | 0.0333 | | | 1.23066 | | | **1.41e-34*** |
|  | **RMSE** | 0.04985 | 0.24925 | | **2.97e-33*** | 0.0962 | 0.44744 | **4.70e-33*** | 0.13477 | | 0.74275 | | **6.52e-34*** | | | 0.18248 | | | 1.10767 | | | **1.41e-34*** |
|  | **MAE** | 0.04234 | 0.21243 | | **4.00e-33*** | 0.07764 | 0.37939 | **4.82e-33*** | 0.10639 | | 0.59426 | | **7.27e-34*** | | | 0.13978 | | | 0.84802 | | | **1.41e-34*** |
| **AMP vs. rSO2_R** | **MSE** | 0.00248 | 0.05907 | | **3.15e-31*** | 0.00926 | 0.21145 | **1.40e-31*** | 0.01816 | | 0.52773 | | **1.43e-32*** | | | 0.0333 | | | 1.24852 | | | **1.20e-34*** |
|  | **RMSE** | 0.04985 | 0.24305 | | **3.15e-31*** | 0.0962 | 0.45983 | **1.40e-31*** | 0.13477 | | 0.72639 | | **1.09e-32*** | | | 0.18248 | | | 1.10751 | | | **1.16e-34*** |
|  | **MAE** | 0.04234 | 0.20723 | | **4.10e-31*** | 0.07764 | 0.38773 | **1.13e-31*** | 0.10639 | | 0.59221 | | **1.03e-32*** | | | 0.13978 | | | 0.83342 | | | **1.77e-34*** |
|  | | **ICP Monitor Opposite Side as NIRS** | | | | | | | | | | | | | | | | | | | | |
| **ICP vs. rSO2_L** | **MSE** | 0.11249 | 0.0649 | | **7.76e-04*** | 0.38779 | 0.22798 | **0.01733** | 0.88805 | | 0.56358 | | **0.03471** | | | 1.79385 | | | 1.26527 | | | 0.05496 |
|  | **RMSE** | 0.33539 | 0.25476 | | **8.18e-04*** | 0.62264 | 0.47747 | **0.01694** | 0.94236 | | 0.75069 | | **0.03489** | | | 1.33935 | | | 1.11855 | | | 0.05684 |
|  | **MAE** | 0.28493 | 0.21347 | | **8.12e-04*** | 0.50573 | 0.38879 | **0.01855** | 0.74953 | | 0.61295 | | 0.05061 | | | 1.04058 | | | 0.87581 | | | 0.10661 |
| **ICP vs. rSO2_R** | **MSE** | 0.11249 | 0.05919 | | **8.05e-05*** | 0.38779 | 0.20926 | **0.00183** | 0.88805 | | 0.5367 | | **0.01056** | | | 1.79385 | | | 1.25438 | | | **0.01692** |
|  | **RMSE** | 0.33539 | 0.24328 | | **8.43e-05*** | 0.62264 | 0.45745 | **0.00199** | 0.94236 | | 0.7326 | | **0.01056** | | | 1.33935 | | | 1.11948 | | | **0.01733** |
|  | **MAE** | 0.28493 | 0.20813 | | **7.90e-05*** | 0.50573 | 0.38723 | **0.00432** | 0.74953 | | 0.59518 | | **0.01723** | | | 1.04058 | | | 0.83528 | | | **0.02598** |
| **AMP vs. rSO2_L** | **MSE** | 0.00248 | 0.0649 | | **8.60e-35*** | 0.00926 | 0.22798 | **1.33e-34*** | 0.01816 | | 0.56358 | | **2.74e-35*** | | | 0.0333 | | | 1.26527 | | | **4.13e-36*** |
|  | **RMSE** | 0.04985 | 0.25476 | | **8.60e-35*** | 0.0962 | 0.47747 | **1.33e-34*** | 0.13477 | | 0.75069 | | **1.95e-35*** | | | 0.18248 | | | 1.11855 | | | **4.13e-36*** |
|  | **MAE** | 0.04234 | 0.21347 | | **1.14e-34*** | 0.07764 | 0.38879 | **1.44e-34*** | 0.10639 | | 0.61295 | | **2.16e-35*** | | | 0.13978 | | | 0.87581 | | | **4.02e-36*** |
| **AMP vs. rSO2_R** | **MSE** | 0.00248 | 0.05919 | | **1.32e-31*** | 0.00926 | 0.20926 | **5.94e-32*** | 0.01816 | | 0.5367 | | **6.09e-33*** | | | 0.0333 | | | 1.25438 | | | **4.87e-35*** |
|  | **RMSE** | 0.04985 | 0.24328 | | **1.32e-31*** | 0.0962 | 0.45745 | **5.94e-32*** | 0.13477 | | 0.7326 | | **4.79e-33*** | | | 0.18248 | | | 1.11948 | | | **4.73e-35*** |
|  | **MAE** | 0.04234 | 0.20813 | | **1.71e-31*** | 0.07764 | 0.38723 | **4.69e-32*** | 0.10639 | | 0.59518 | | **4.66e-33*** | | | 0.13978 | | | 0.83528 | | | **6.96e-35*** |
|  | | **Primary Decompressive Craniectomy** | | | | | | | | | | | | | | | | | | | | |
| **ICP vs. rSO2_L** | **MSE** | 0.11017 | 0.05181 | | 0.05636 | 0.38494 | 0.17143 | 0.09935 | 0.75459 | | 0.46861 | | 0.11442 | | | 1.43455 | | | 1.43671 | | | 0.28996 |
|  | **RMSE** | 0.33192 | 0.22762 | | 0.06099 | 0.62043 | 0.41404 | 0.09935 | 0.86867 | | 0.68455 | | 0.11846 | | | 1.19761 | | | 1.19841 | | | 0.28213 |
|  | **MAE** | 0.28 | 0.19093 | | 0.07121 | 0.50916 | 0.34147 | 0.09935 | 0.70494 | | 0.55524 | | 0.19323 | | | 0.91043 | | | 0.88199 | | | 0.21807 |
| **ICP vs. rSO2_R** | **MSE** | 0.11017 | 0.04587 | | **0.01593** | 0.38494 | 0.15157 | 0.05102 | 0.75459 | | 0.34504 | | 0.09123 | | | 1.43455 | | | 0.873 | | | 0.20674 |
|  | **RMSE** | 0.33192 | 0.21417 | | **0.01743** | 0.62043 | 0.38931 | 0.05300 | 0.86867 | | 0.5874 | | 0.08813 | | | 1.19761 | | | 0.93435 | | | 0.20674 |
|  | **MAE** | 0.28 | 0.18253 | | **0.01391** | 0.50916 | 0.34159 | 0.08512 | 0.70494 | | 0.47114 | | 0.14447 | | | 0.91043 | | | 0.73695 | | | 0.20674 |
| **AMP vs. rSO2_L** | **MSE** | 0.00243 | 0.05181 | | **4.53e-09*** | 0.01067 | 0.17143 | **8.20e-10*** | 0.02166 | | 0.46861 | | **1.14e-09*** | | | 0.0333 | | | 1.43671 | | | **1.14e-09*** |
|  | **RMSE** | 0.04927 | 0.22762 | | **4.53e-09*** | 0.10331 | 0.41404 | **8.20e-10*** | 0.14675 | | 0.68455 | | **1.14e-09*** | | | 0.18248 | | | 1.19841 | | | **1.14e-09*** |
|  | **MAE** | 0.04131 | 0.19093 | | **4.53e-09*** | 0.08016 | 0.34147 | **7.35e-10*** | 0.10409 | | 0.55524 | | **1.14e-09*** | | | 0.13978 | | | 0.88199 | | | **1.14e-09*** |
| **AMP vs. rSO2_R** | **MSE** | 0.00243 | 0.04587 | | **9.58e-10*** | 0.01067 | 0.15157 | **1.61e-10*** | 0.02166 | | 0.34504 | | **1.44e-10*** | | | 0.0333 | | | 0.873 | | | **1.61e-10*** |
|  | **RMSE** | 0.04927 | 0.21417 | | **9.58e-10*** | 0.10331 | 0.38931 | **1.61e-10*** | 0.14675 | | 0.5874 | | **1.44e-10*** | | | 0.18248 | | | 0.93435 | | | **1.61e-10*** |
|  | **MAE** | 0.04131 | 0.18253 | | **9.58e-10*** | 0.08016 | 0.34159 | **1.44e-10*** | 0.10409 | | 0.47114 | | **1.44e-10*** | | | 0.13978 | | | 0.73695 | | | **1.61e-10*** |
|  | | **Secondary Decompressive Craniectomy** | | | | | | | | | | | | | | | | | | | | |
| **ICP vs. rSO2_L** | **MSE** | 0.23253 | 0.09848 | | 0.15986 | 0.78128 | 0.33629 | 0.25015 | 1.88089 | | 0.81441 | | 0.12520 | | | 3.30031 | | | 1.21776 | | | 0.12520 |
|  | **RMSE** | 0.48222 | 0.31382 | | 0.15986 | 0.88387 | 0.5799 | 0.20134 | 1.37146 | | 0.90245 | | 0.12520 | | | 1.81665 | | | 1.10344 | | | 0.12520 |
|  | **MAE** | 0.40984 | 0.26604 | | 0.15986 | 0.68832 | 0.47757 | 0.09670 | 1.07585 | | 0.67438 | | 0.09670 | | | 1.39308 | | | 0.82361 | | | 0.07364 |
| **ICP vs. rSO2_R** | **MSE** | 0.23253 | 0.08496 | | 0.32986 | 0.78128 | 0.4616 | 0.32986 | 1.88089 | | 1.47368 | | 0.32986 | | | 3.30031 | | | 2.37 | | | 0.32986 |
|  | **RMSE** | 0.48222 | 0.29148 | | 0.32986 | 0.88387 | 0.67924 | 0.32986 | 1.37146 | | 1.21395 | | 0.32986 | | | 1.81665 | | | 1.53313 | | | 0.32986 |
|  | **MAE** | 0.40984 | 0.2412 | | 0.32986 | 0.68832 | 0.55829 | 0.62612 | 1.07585 | | 0.97479 | | 0.32986 | | | 1.39308 | | | 1.18944 | | | 0.62612 |
| **AMP vs. rSO2_L** | **MSE** | 0.00358 | 0.09848 | | **0.01519** | 0.01392 | 0.33629 | **0.00217** | 0.03178 | | 0.81441 | | **0.00217** | | | 0.05103 | | | 1.21776 | | | **0.00217** |
|  | **RMSE** | 0.05986 | 0.31382 | | **0.01519** | 0.11797 | 0.5799 | **0.00217** | 0.17828 | | 0.90245 | | **0.00217** | | | 0.22585 | | | 1.10344 | | | **0.00217** |
|  | **MAE** | 0.05148 | 0.26604 | | **0.02145** | 0.09644 | 0.47757 | **0.00217** | 0.13622 | | 0.67438 | | **0.00217** | | | 0.17478 | | | 0.82361 | | | **0.00217** |
| **AMP vs. rSO2_R** | **MSE** | 0.00358 | 0.08496 | | **0.03476** | 0.01392 | 0.4616 | **0.01485** | 0.03178 | | 1.47368 | | **0.00937** | | | 0.05103 | | | 2.37 | | | **0.00577** |
|  | **RMSE** | 0.05986 | 0.29148 | | **0.03476** | 0.11797 | 0.67924 | **0.01485** | 0.17828 | | 1.21395 | | **0.00937** | | | 0.22585 | | | 1.53313 | | | **0.00577** |
|  | **MAE** | 0.05148 | 0.2412 | | **0.03476** | 0.09644 | 0.55829 | **0.00937** | 0.13622 | | 0.97479 | | **0.00937** | | | 0.17478 | | | 1.18944 | | | **0.00577** |
|  | | **No Decompressive Craniectomy** | | | | | | | | | | | | | | | | | | | | |
| **ICP vs. rSO2_L** | **MSE** | 0.1156 | 0.06819 | | **0.00883** | 0.3808 | 0.21236 | 0.09733 | 0.85445 | | 0.57036 | | 0.16699 | | | 1.76355 | | | 1.26527 | | | 0.19625 |
|  | **RMSE** | 0.33997 | 0.26113 | | **0.00883** | 0.61702 | 0.46082 | 0.09733 | 0.9243 | | 0.75522 | | 0.16699 | | | 1.3275 | | | 1.11855 | | | 0.20253 |
|  | **MAE** | 0.2894 | 0.22153 | | **0.00778** | 0.48611 | 0.38982 | 0.12019 | 0.74922 | | 0.61749 | | 0.20000 | | | 1.01252 | | | 0.87581 | | | 0.42510 |
| **ICP vs. rSO2_R** | **MSE** | 0.1156 | 0.06379 | | **0.00251** | 0.3808 | 0.22098 | **0.02096** | 0.85445 | | 0.58944 | | 0.07072 | | | 1.76355 | | | 1.37144 | | | 0.09073 |
|  | **RMSE** | 0.33997 | 0.25256 | | **0.00251** | 0.61702 | 0.47006 | **0.02096** | 0.9243 | | 0.76772 | | 0.07132 | | | 1.3275 | | | 1.17109 | | | 0.09371 |
|  | **MAE** | 0.2894 | 0.222 | | **0.00226** | 0.48611 | 0.39004 | **0.03181** | 0.74922 | | 0.61196 | | 0.07889 | | | 1.01252 | | | 0.86648 | | | 0.10644 |
| **AMP vs. rSO2_L** | **MSE** | 0.00242 | 0.06819 | | **4.99e-26*** | 0.00866 | 0.21236 | **9.77e-25*** | 0.01799 | | 0.57036 | | **4.46e-25*** | | | 0.03261 | | | 1.26527 | | | **1.11e-25*** |
|  | **RMSE** | 0.04915 | 0.26113 | | **4.99e-26*** | 0.09306 | 0.46082 | **9.77e-25*** | 0.13414 | | 0.75522 | | **3.31e-25*** | | | 0.18059 | | | 1.11855 | | | **1.11e-25*** |
|  | **MAE** | 0.04253 | 0.22153 | | **4.99e-26*** | 0.07526 | 0.38982 | **1.31e-24*** | 0.1068 | | 0.61749 | | **3.70e-25*** | | | 0.13569 | | | 0.87581 | | | **1.44e-25*** |
| **AMP vs. rSO2_R** | **MSE** | 0.00242 | 0.06379 | | **1.06e-22*** | 0.00866 | 0.22098 | **6.65e-22*** | 0.01799 | | 0.58944 | | **1.47e-22*** | | | 0.03261 | | | 1.37144 | | | **1.88e-24*** |
|  | **RMSE** | 0.04915 | 0.25256 | | **1.06e-22*** | 0.09306 | 0.47006 | **6.65e-22*** | 0.13414 | | 0.76772 | | **1.12e-22*** | | | 0.18059 | | | 1.17109 | | | **1.80e-24*** |
|  | **MAE** | 0.04253 | 0.222 | | **1.10e-22*** | 0.07526 | 0.39004 | **7.17e-22*** | 0.1068 | | 0.61196 | | **1.21e-22*** | | | 0.13569 | | | 0.86648 | | | **2.90e-24*** |
|  | | **Glasgow Coma Scale of 8 or less** | | | | | | | | | | | | | | | | | | | | |
| **ICP vs. rSO2_L** | **MSE** | 0.12464 | 0.06994 | | **0.00108** | 0.43026 | 0.25841 | **0.03203** | 0.91328 | | 0.66491 | | 0.06788 | | | 1.92286 | | | 1.47561 | | | 0.14440 |
|  | **RMSE** | 0.35303 | 0.26443 | | **0.00114** | 0.65592 | 0.50818 | **0.03095** | 0.95556 | | 0.81514 | | 0.06788 | | | 1.38666 | | | 1.21366 | | | 0.14725 |
|  | **MAE** | 0.30192 | 0.22417 | | **0.00105** | 0.51053 | 0.40661 | **0.03762** | 0.76747 | | 0.64961 | | 0.08242 | | | 1.08823 | | | 0.92794 | | | 0.23435 |
| **ICP vs. rSO2_R** | **MSE** | 0.12464 | 0.06241 | | **2.46e-04*** | 0.43026 | 0.23755 | **0.00709** | 0.91328 | | 0.63237 | | **0.04132** | | | 1.92286 | | | 1.59578 | | | 0.09423 |
|  | **RMSE** | 0.35303 | 0.24982 | | **2.57e-04*** | 0.65592 | 0.48738 | **0.00755** | 0.95556 | | 0.79521 | | **0.04063** | | | 1.38666 | | | 1.26324 | | | 0.09633 |
|  | **MAE** | 0.30192 | 0.2216 | | **2.75e-04*** | 0.51053 | 0.39556 | **0.01577** | 0.76747 | | 0.63444 | | 0.05393 | | | 1.08823 | | | 0.9486 | | | 0.12113 |
| **AMP vs. rSO2_L** | **MSE** | 0.00256 | 0.06994 | | **8.23e-26*** | 0.00927 | 0.25841 | **9.51e-26*** | 0.01818 | | 0.66491 | | **3.43e-26*** | | | 0.03425 | | | 1.47561 | | | **6.55e-27*** |
|  | **RMSE** | 0.05061 | 0.26443 | | **8.23e-26*** | 0.0963 | 0.50818 | **9.51e-26*** | 0.13483 | | 0.81514 | | **3.56e-26*** | | | 0.18507 | | | 1.21366 | | | **6.31e-27*** |
|  | **MAE** | 0.04308 | 0.22417 | | **1.06e-25*** | 0.07867 | 0.40661 | **1.27e-25*** | 0.10762 | | 0.64961 | | **3.08e-26*** | | | 0.14617 | | | 0.92794 | | | **7.05e-27*** |
| **AMP vs. rSO2_R** | **MSE** | 0.00256 | 0.06241 | | **1.04e-23*** | 0.00927 | 0.23755 | **6.21e-24*** | 0.01818 | | 0.63237 | | **1.41e-24*** | | | 0.03425 | | | 1.59578 | | | **1.69e-26*** |
|  | **RMSE** | 0.05061 | 0.24982 | | **1.04e-23*** | 0.0963 | 0.48738 | **6.21e-24*** | 0.13483 | | 0.79521 | | **1.41e-24*** | | | 0.18507 | | | 1.26324 | | | **1.69e-26*** |
|  | **MAE** | 0.04308 | 0.2216 | | **1.25e-23*** | 0.07867 | 0.39556 | **5.99e-24*** | 0.10762 | | 0.63444 | | **1.36e-24*** | | | 0.14617 | | | 0.9486 | | | **2.05e-26*** |
|  | | **Glasgow Coma Scale of 9 - 12** | | | | | | | | | | | | | | | | | | | | |
| **ICP vs. rSO2_L** | **MSE** | 0.08039 | 0.06518 | | 0.47145 | 0.31476 | 0.2035 | 0.44501 | 0.63537 | | 0.50643 | | 0.49874 | | | 1.04773 | | | 1.08234 | | | 0.44501 |
|  | **RMSE** | 0.28353 | 0.2553 | | 0.47145 | 0.56103 | 0.45101 | 0.44501 | 0.79708 | | 0.71063 | | 0.49874 | | | 1.02337 | | | 1.03997 | | | 0.44501 |
|  | **MAE** | 0.24481 | 0.21507 | | 0.49874 | 0.46936 | 0.37343 | 0.41943 | 0.61339 | | 0.56899 | | 0.55573 | | | 0.77943 | | | 0.79349 | | | 0.64677 |
| **ICP vs. rSO2_R** | **MSE** | 0.08039 | 0.06171 | | 0.56145 | 0.31476 | 0.18657 | 0.67830 | 0.63537 | | 0.41911 | | 0.74002 | | | 1.04773 | | | 0.96721 | | | 0.38373 |
|  | **RMSE** | 0.28353 | 0.24841 | | 0.56145 | 0.56103 | 0.43183 | 0.70892 | 0.79708 | | 0.64738 | | 0.74002 | | | 1.02337 | | | 0.98347 | | | 0.36150 |
|  | **MAE** | 0.24481 | 0.21873 | | 0.53383 | 0.46936 | 0.38159 | 0.67830 | 0.61339 | | 0.51915 | | 0.90097 | | | 0.77943 | | | 0.768 | | | 0.56145 |
| **AMP vs. rSO2_L** | **MSE** | 0.00182 | 0.06518 | | **7.82e-05*** | 0.00524 | 0.2035 | **2.54e-05*** | 0.01778 | | 0.50643 | | **5.10e-06*** | | | 0.03646 | | | 1.08234 | | | **5.10e-06*** |
|  | **RMSE** | 0.04261 | 0.2553 | | **7.82e-05*** | 0.07236 | 0.45101 | **2.54e-05*** | 0.13333 | | 0.71063 | | **5.10e-06*** | | | 0.19096 | | | 1.03997 | | | **5.10e-06*** |
|  | **MAE** | 0.03626 | 0.21507 | | **7.82e-05*** | 0.05782 | 0.37343 | **1.72e-05*** | 0.09602 | | 0.56899 | | **5.10e-06*** | | | 0.12454 | | | 0.79349 | | | **5.10e-06*** |
| **AMP vs. rSO2_R** | **MSE** | 0.00182 | 0.06171 | | **2.80e-05*** | 0.00524 | 0.18657 | **9.07e-06*** | 0.01778 | | 0.41911 | | **3.39e-06*** | | | 0.03646 | | | 0.96721 | | | **3.39e-06*** |
|  | **RMSE** | 0.04261 | 0.24841 | | **2.80e-05*** | 0.07236 | 0.43183 | **9.07e-06*** | 0.13333 | | 0.64738 | | **3.39e-06*** | | | 0.19096 | | | 0.98347 | | | **3.39e-06*** |
|  | **MAE** | 0.03626 | 0.21873 | | **3.36e-05*** | 0.05782 | 0.38159 | **6.15e-06*** | 0.09602 | | 0.51915 | | **3.39e-06*** | | | 0.12454 | | | 0.768 | | | **3.39e-06*** |
|  | | **Pupil Response Category – Bilateral Reactive** | | | | | | | | | | | | | | | | | | | | |
| **ICP vs. rSO2_L** | **MSE** | 0.12776 | 0.0649 | | **0.00117** | 0.45559 | 0.22798 | **0.00440** | 1.07736 | | 0.57453 | | **0.00445** | | | 2.36343 | | | 1.29924 | | | **0.00484** |
|  | **RMSE** | 0.35743 | 0.25476 | | **0.00117** | 0.67487 | 0.47747 | **0.00395** | 1.03796 | | 0.75798 | | **0.00445** | | | 1.53734 | | | 1.13984 | | | **0.00496** |
|  | **MAE** | 0.30321 | 0.21347 | | **0.00106** | 0.53341 | 0.38879 | **0.00430** | 0.82321 | | 0.62144 | | **0.00520** | | | 1.16995 | | | 0.87911 | | | **0.01047** |
| **ICP vs. rSO2_R** | **MSE** | 0.12776 | 0.05936 | | **4.05e-04*** | 0.45559 | 0.21659 | **0.00197** | 1.07736 | | 0.55494 | | **0.00351** | | | 2.36343 | | | 1.25738 | | | **0.00381** |
|  | **RMSE** | 0.35743 | 0.24365 | | **4.05e-04*** | 0.67487 | 0.46539 | **0.00202** | 1.03796 | | 0.74494 | | **0.00351** | | | 1.53734 | | | 1.12133 | | | **0.00386** |
|  | **MAE** | 0.30321 | 0.2102 | | **3.27e-04*** | 0.53341 | 0.39298 | **0.00332** | 0.82321 | | 0.60127 | | **0.00351** | | | 1.16995 | | | 0.85946 | | | **0.00554** |
| **AMP vs. rSO2_L** | **MSE** | 0.00272 | 0.0649 | | **4.11e-24*** | 0.01127 | 0.22798 | **1.55e-23*** | 0.02317 | | 0.57453 | | **2.28e-24*** | | | 0.05001 | | | 1.29924 | | | **8.80e-25*** |
|  | **RMSE** | 0.05218 | 0.25476 | | **4.11e-24*** | 0.10616 | 0.47747 | **1.55e-23*** | 0.15221 | | 0.75798 | | **2.28e-24*** | | | 0.22362 | | | 1.13984 | | | **8.46e-25*** |
|  | **MAE** | 0.04523 | 0.21347 | | **4.81e-24*** | 0.08674 | 0.38879 | **1.18e-23*** | 0.11504 | | 0.62144 | | **2.78e-24*** | | | 0.16232 | | | 0.87911 | | | **6.66e-25*** |
| **AMP vs. rSO2_R** | **MSE** | 0.00272 | 0.05936 | | **5.55e-22*** | 0.01127 | 0.21659 | **7.43e-22*** | 0.02317 | | 0.55494 | | **1.17e-22*** | | | 0.05001 | | | 1.25738 | | | **2.62e-23*** |
|  | **RMSE** | 0.05218 | 0.24365 | | **5.55e-22*** | 0.10616 | 0.46539 | **7.43e-22*** | 0.15221 | | 0.74494 | | **1.17e-22*** | | | 0.22362 | | | 1.12133 | | | **2.51e-23*** |
|  | **MAE** | 0.04523 | 0.2102 | | **6.83e-22*** | 0.08674 | 0.39298 | **4.89e-22*** | 0.11504 | | 0.60127 | | **1.07e-22*** | | | 0.16232 | | | 0.85946 | | | **2.98e-23*** |
| **c** | | **Pupil Response Category – Bilateral Unreactive** | | | | | | | | | | | | | | | | | | | | |
| **ICP vs. rSO2_L** | **MSE** | 0.08022 | 0.06015 | | 0.32408 | 0.27458 | 0.18575 | 0.71638 | 0.53665 | | 0.44947 | | 0.79525 | | | 0.93022 | | | 1.06479 | | | 0.83554 |
|  | **RMSE** | 0.28323 | 0.24525 | | 0.32408 | 0.48915 | 0.43098 | 0.71638 | 0.73244 | | 0.67043 | | 0.79525 | | | 0.96336 | | | 1.03187 | | | 0.83554 |
|  | **MAE** | 0.23827 | 0.212 | | 0.40631 | 0.39721 | 0.3537 | 0.71638 | 0.5816 | | 0.5065 | | 0.71638 | | | 0.82114 | | | 0.80448 | | | 1.00000 |
| **ICP vs. rSO2_R** | **MSE** | 0.08022 | 0.05515 | | 0.44501 | 0.27458 | 0.20596 | 0.77665 | 0.53665 | | 0.41122 | | 0.84430 | | | 0.93022 | | | 0.89115 | | | 0.61574 |
|  | **RMSE** | 0.28323 | 0.2344 | | 0.47145 | 0.48915 | 0.45376 | 0.77665 | 0.73244 | | 0.64113 | | 0.84430 | | | 0.96336 | | | 0.94392 | | | 0.61574 |
|  | **MAE** | 0.23827 | 0.19687 | | 0.47145 | 0.39721 | 0.37362 | 0.94780 | 0.5816 | | 0.49983 | | 0.84430 | | | 0.82114 | | | 0.73824 | | | 0.77665 |
| **AMP vs. rSO2_L** | **MSE** | 0.00118 | 0.06015 | | **6.43e-05*** | 0.0052 | 0.18575 | **2.08e-05*** | 0.01196 | | 0.44947 | | **4.13e-05*** | | | 0.02483 | | | 1.06479 | | | **2.08e-05*** |
|  | **RMSE** | 0.03441 | 0.24525 | | **6.43e-05*** | 0.07209 | 0.43098 | **2.08e-05*** | 0.10935 | | 0.67043 | | **4.13e-05*** | | | 0.15757 | | | 1.03187 | | | **2.08e-05*** |
|  | **MAE** | 0.02937 | 0.212 | | **6.43e-05*** | 0.05771 | 0.3537 | **2.62e-05*** | 0.08819 | | 0.5065 | | **4.13e-05*** | | | 0.11788 | | | 0.80448 | | | **2.08e-05*** |
| **AMP vs. rSO2_R** | **MSE** | 0.00118 | 0.05515 | | **1.15e-05*** | 0.0052 | 0.20596 | **5.10e-06*** | 0.01196 | | 0.41122 | | **5.10e-06*** | | | 0.02483 | | | 0.89115 | | | **5.10e-06*** |
|  | **RMSE** | 0.03441 | 0.2344 | | **1.15e-05*** | 0.07209 | 0.45376 | **5.10e-06*** | 0.10935 | | 0.64113 | | **5.10e-06*** | | | 0.15757 | | | 0.94392 | | | **5.10e-06*** |
|  | **MAE** | 0.02937 | 0.19687 | | **1.15e-05*** | 0.05771 | 0.37362 | **5.10e-06*** | 0.08819 | | 0.49983 | | **5.10e-06*** | | | 0.11788 | | | 0.73824 | | | **5.10e-06*** |
|  | | **Pupil Response Category – Unilateral Unreactive** | | | | | | | | | | | | | | | | | | | | |
| **ICP vs. rSO2_L** | **MSE** | 0.10512 | 0.07998 | | 0.27491 | 0.32824 | 0.24331 | 0.84906 | 0.66308 | | 0.57036 | | 0.92817 | | | 1.33921 | | | 1.20702 | | | 0.97603 |
|  | **RMSE** | 0.32414 | 0.2828 | | 0.27491 | 0.5729 | 0.49327 | 0.84906 | 0.8141 | | 0.75522 | | 0.92817 | | | 1.15719 | | | 1.09865 | | | 0.94410 |
|  | **MAE** | 0.27105 | 0.2318 | | 0.29291 | 0.46149 | 0.38982 | 0.84906 | 0.63616 | | 0.60153 | | 0.78681 | | | 0.89976 | | | 0.84031 | | | 0.72590 |
| **ICP vs. rSO2_R** | **MSE** | 0.10512 | 0.05896 | | 0.06387 | 0.32824 | 0.20592 | 0.21054 | 0.66308 | | 0.58028 | | 0.56803 | | | 1.33921 | | | 1.37132 | | | 0.74098 |
|  | **RMSE** | 0.32414 | 0.24281 | | 0.06387 | 0.5729 | 0.45378 | 0.21054 | 0.8141 | | 0.76175 | | 0.56803 | | | 1.15719 | | | 1.17103 | | | 0.75617 |
|  | **MAE** | 0.27105 | 0.20633 | | 0.07962 | 0.46149 | 0.36667 | 0.26620 | 0.63616 | | 0.61895 | | 0.74098 | | | 0.89976 | | | 0.85535 | | | 0.86479 |
| **AMP vs. rSO2_L** | **MSE** | 0.00202 | 0.07998 | | **1.35e-08*** | 0.00687 | 0.24331 | **1.71e-08*** | 0.01545 | | 0.57036 | | **1.20e-08*** | | | 0.0233 | | | 1.20702 | | | **1.35e-08*** |
|  | **RMSE** | 0.04491 | 0.2828 | | **1.35e-08*** | 0.08266 | 0.49327 | **1.71e-08*** | 0.12409 | | 0.75522 | | **8.43e-09*** | | | 0.15253 | | | 1.09865 | | | **1.35e-08*** |
|  | **MAE** | 0.03808 | 0.2318 | | **1.35e-08*** | 0.06331 | 0.38982 | **1.71e-08*** | 0.09878 | | 0.60153 | | **9.49e-09*** | | | 0.11998 | | | 0.84031 | | | **1.71e-08*** |
| **AMP vs. rSO2_R** | **MSE** | 0.00202 | 0.05896 | | **5.78e-07*** | 0.00687 | 0.20592 | **4.23e-07*** | 0.01545 | | 0.58028 | | **3.08e-07*** | | | 0.0233 | | | 1.37132 | | | **1.07e-08*** |
|  | **RMSE** | 0.04491 | 0.24281 | | **5.78e-07*** | 0.08266 | 0.45378 | **4.23e-07*** | 0.12409 | | 0.76175 | | **3.08e-07*** | | | 0.15253 | | | 1.17103 | | | **1.07e-08*** |
|  | **MAE** | 0.03808 | 0.20633 | | **5.21e-07*** | 0.06331 | 0.36667 | **4.69e-07*** | 0.09878 | | 0.61895 | | **2.77e-07*** | | | 0.11998 | | | 0.85535 | | | **1.71e-08*** |
|  | | **Rotterdam CT Grade of 1** | | | | | | | | | | | | | | | | | | | | |
| **ICP vs. rSO2_L** | **MSE** | 0.13625 | 0.08901 | | 1.00000 | 0.43615 | 0.33629 | 1.00000 | 1.13395 | | 0.81441 | | 1.00000 | | | 2.56041 | | | 1.93441 | | | 1.00000 |
|  | **RMSE** | 0.36912 | 0.29834 | | 1.00000 | 0.66042 | 0.5799 | 1.00000 | 1.06487 | | 0.90245 | | 1.00000 | | | 1.60013 | | | 1.39078 | | | 1.00000 |
|  | **MAE** | 0.30911 | 0.25353 | | 1.00000 | 0.5318 | 0.47757 | 1.00000 | 0.82321 | | 0.67438 | | 1.00000 | | | 1.13327 | | | 1.0427 | | | 1.00000 |
| **ICP vs. rSO2_R** | **MSE** | 0.13625 | 0.05548 | | 1.00000 | 0.43615 | 0.21596 | 1.00000 | 1.13395 | | 0.5367 | | 1.00000 | | | 2.56041 | | | 0.94724 | | | 1.00000 |
|  | **RMSE** | 0.36912 | 0.23554 | | 1.00000 | 0.66042 | 0.46472 | 1.00000 | 1.06487 | | 0.7326 | | 1.00000 | | | 1.60013 | | | 0.97326 | | | 1.00000 |
|  | **MAE** | 0.30911 | 0.19907 | | 1.00000 | 0.5318 | 0.37487 | 1.00000 | 0.82321 | | 0.58925 | | 1.00000 | | | 1.13327 | | | 0.78854 | | | 1.00000 |
| **AMP vs. rSO2_L** | **MSE** | 0.00358 | 0.08901 | | 1.00000 | 0.01392 | 0.33629 | 1.00000 | 0.03178 | | 0.81441 | | 1.00000 | | | 0.05103 | | | 1.93441 | | | 1.00000 |
|  | **RMSE** | 0.05986 | 0.29834 | | 1.00000 | 0.11797 | 0.5799 | 1.00000 | 0.17828 | | 0.90245 | | 1.00000 | | | 0.22585 | | | 1.39078 | | | 1.00000 |
|  | **MAE** | 0.05148 | 0.25353 | | 1.00000 | 0.09644 | 0.47757 | 1.00000 | 0.13622 | | 0.67438 | | 1.00000 | | | 0.17478 | | | 1.0427 | | | 1.00000 |
| **AMP vs. rSO2_R** | **MSE** | 0.00358 | 0.05548 | | 1.00000 | 0.01392 | 0.21596 | 1.00000 | 0.03178 | | 0.5367 | | 1.00000 | | | 0.05103 | | | 0.94724 | | | 1.00000 |
|  | **RMSE** | 0.05986 | 0.23554 | | 1.00000 | 0.11797 | 0.46472 | 1.00000 | 0.17828 | | 0.7326 | | 1.00000 | | | 0.22585 | | | 0.97326 | | | 1.00000 |
|  | **MAE** | 0.05148 | 0.19907 | | 1.00000 | 0.09644 | 0.37487 | 1.00000 | 0.13622 | | 0.58925 | | 1.00000 | | | 0.17478 | | | 0.78854 | | | 1.00000 |
|  | | **Rotterdam CT Grade of 2** | | | | | | | | | | | | | | | | | | | | |
| **ICP vs. rSO2_L** | **MSE** | 0.17402 | 0.07989 | | 0.12821 | 0.61623 | 0.38341 | 0.12821 | 1.51364 | | 0.83824 | | 0.12821 | | | 2.77428 | | | 1.85298 | | | 0.37848 |
|  | **RMSE** | 0.4119 | 0.27779 | | 0.12821 | 0.78488 | 0.61218 | 0.12821 | 1.22966 | | 0.90138 | | 0.12821 | | | 1.66265 | | | 1.3456 | | | 0.37848 |
|  | **MAE** | 0.35123 | 0.23287 | | 0.06555 | 0.63806 | 0.49045 | 0.09270 | 0.93637 | | 0.73406 | | 0.17349 | | | 1.27291 | | | 1.0126 | | | 0.29795 |
| **ICP vs. rSO2_R** | **MSE** | 0.17402 | 0.09479 | | 0.33736 | 0.61623 | 0.42333 | 0.45555 | 1.51364 | | 1.03107 | | 0.74912 | | | 2.77428 | | | 2.04975 | | | 0.91511 |
|  | **RMSE** | 0.4119 | 0.29982 | | 0.33736 | 0.78488 | 0.61151 | 0.45555 | 1.22966 | | 0.94324 | | 0.74912 | | | 1.66265 | | | 1.29044 | | | 0.91511 |
|  | **MAE** | 0.35123 | 0.25013 | | 0.24095 | 0.63806 | 0.506 | 0.45555 | 0.93637 | | 0.75501 | | 0.74912 | | | 1.27291 | | | 1.11344 | | | 1.00000 |
| **AMP vs. rSO2_L** | **MSE** | 0.00298 | 0.07989 | | **0.00507** | 0.01103 | 0.38341 | **0.00507** | 0.02614 | | 0.83824 | | **0.00507** | | | 0.04596 | | | 1.85298 | | | **0.00507** |
|  | **RMSE** | 0.0545 | 0.27779 | | **0.00507** | 0.10502 | 0.61218 | **0.00507** | 0.16087 | | 0.90138 | | **0.00507** | | | 0.21229 | | | 1.3456 | | | **0.00507** |
|  | **MAE** | 0.04579 | 0.23287 | | **0.00507** | 0.08429 | 0.49045 | **0.00507** | 0.11637 | | 0.73406 | | **0.00507** | | | 0.1659 | | | 1.0126 | | | **0.00507** |
| **AMP vs. rSO2_R** | **MSE** | 0.00298 | 0.09479 | | **0.01421** | 0.01103 | 0.42333 | **0.01421** | 0.02614 | | 1.03107 | | **0.01421** | | | 0.04596 | | | 2.04975 | | | **0.01421** |
|  | **RMSE** | 0.0545 | 0.29982 | | **0.01421** | 0.10502 | 0.61151 | **0.01421** | 0.16087 | | 0.94324 | | **0.01421** | | | 0.21229 | | | 1.29044 | | | **0.01421** |
|  | **MAE** | 0.04579 | 0.25013 | | **0.01421** | 0.08429 | 0.506 | **0.01421** | 0.11637 | | 0.75501 | | **0.01421** | | | 0.1659 | | | 1.11344 | | | **0.01421** |
|  | | **Rotterdam CT Grade of 3** | | | | | | | | | | | | | | | | | | | | |
| **ICP vs. rSO2_L** | **MSE** | 0.17473 | 0.08708 | | **0.00364** | 0.59846 | 0.3083 | **0.00935** | 1.19194 | | 0.8608 | | **0.04164** | | | 2.78544 | | | 1.68801 | | | **0.04760** |
|  | **RMSE** | 0.41799 | 0.29509 | | **0.00435** | 0.77309 | 0.55525 | **0.00935** | 1.09175 | | 0.92779 | | **0.04164** | | | 1.6659 | | | 1.29904 | | | **0.04760** |
|  | **MAE** | 0.35853 | 0.24707 | | **0.00364** | 0.61162 | 0.44793 | **0.01395** | 0.87829 | | 0.69319 | | **0.02202** | | | 1.24838 | | | 1.03315 | | | 0.07439 |
| **ICP vs. rSO2_R** | **MSE** | 0.17473 | 0.07786 | | **6.59e-04*** | 0.59846 | 0.2234 | **0.00204** | 1.19194 | | 0.58106 | | **0.00627** | | | 2.78544 | | | 1.21832 | | | **0.00300** |
|  | **RMSE** | 0.41799 | 0.279 | | **6.59e-04*** | 0.77309 | 0.47241 | **0.00204** | 1.09175 | | 0.75957 | | **0.00627** | | | 1.6659 | | | 1.10334 | | | **0.00300** |
|  | **MAE** | 0.35853 | 0.23747 | | **5.31e-04*** | 0.61162 | 0.38501 | **0.00185** | 0.87829 | | 0.58159 | | **0.00398** | | | 1.24838 | | | 0.84737 | | | **0.00151** |
| **AMP vs. rSO2_L** | **MSE** | 0.00569 | 0.08708 | | **1.87e-07*** | 0.01833 | 0.3083 | **1.01e-07*** | 0.03527 | | 0.8608 | | **1.01e-07*** | | | 0.06256 | | | 1.68801 | | | **1.01e-07*** |
|  | **RMSE** | 0.07545 | 0.29509 | | **1.87e-07*** | 0.13537 | 0.55525 | **1.01e-07*** | 0.1878 | | 0.92779 | | **1.01e-07*** | | | 0.25012 | | | 1.29904 | | | **1.01e-07*** |
|  | **MAE** | 0.06393 | 0.24707 | | **1.87e-07*** | 0.10708 | 0.44793 | **1.01e-07*** | 0.14965 | | 0.69319 | | **1.01e-07*** | | | 0.19813 | | | 1.03315 | | | **1.01e-07*** |
| **AMP vs. rSO2_R** | **MSE** | 0.00569 | 0.07786 | | **1.54e-07*** | 0.01833 | 0.2234 | **1.54e-07*** | 0.03527 | | 0.58106 | | **1.54e-07*** | | | 0.06256 | | | 1.21832 | | | **1.54e-07*** |
|  | **RMSE** | 0.07545 | 0.279 | | **1.54e-07*** | 0.13537 | 0.47241 | **1.54e-07*** | 0.1878 | | 0.75957 | | **1.54e-07*** | | | 0.25012 | | | 1.10334 | | | **1.54e-07*** |
|  | **MAE** | 0.06393 | 0.23747 | | **1.54e-07*** | 0.10708 | 0.38501 | **1.54e-07*** | 0.14965 | | 0.58159 | | **1.54e-07*** | | | 0.19813 | | | 0.84737 | | | **1.54e-07*** |
|  | | **Rotterdam CT Grade of 4** | | | | | | | | | | | | | | | | | | | | |
| **ICP vs. rSO2_L** | **MSE** | 0.12776 | 0.05951 | | **2.88e-04*** | 0.48482 | 0.18555 | **3.98e-04*** | 1.2356 | | 0.50479 | | **0.00101*** | | | 2.86829 | | | 1.10086 | | | **0.00150** |
|  | **RMSE** | 0.35743 | 0.24395 | | **2.88e-04*** | 0.69629 | 0.43075 | **3.39e-04*** | 1.11158 | | 0.71049 | | **0.00101*** | | | 1.6936 | | | 1.04922 | | | **0.00150** |
|  | **MAE** | 0.30328 | 0.20427 | | **3.78e-04*** | 0.56457 | 0.35572 | **6.74e-04*** | 0.88334 | | 0.55655 | | **0.00118** | | | 1.23404 | | | 0.80392 | | | **0.00158** |
| **ICP vs. rSO2_R** | **MSE** | 0.12776 | 0.0582 | | **0.00149** | 0.48482 | 0.20985 | **0.00530** | 1.2356 | | 0.5354 | | **0.01310** | | | 2.86829 | | | 1.2396 | | | **0.03634** |
|  | **RMSE** | 0.35743 | 0.24124 | | **0.00149** | 0.69629 | 0.45805 | **0.00530** | 1.11158 | | 0.73123 | | **0.01252** | | | 1.6936 | | | 1.11294 | | | **0.03634** |
|  | **MAE** | 0.30328 | 0.20723 | | **0.00134** | 0.56457 | 0.38 | **0.00994** | 0.88334 | | 0.58466 | | **0.01252** | | | 1.23404 | | | 0.83689 | | | **0.03781** |
| **AMP vs. rSO2_L** | **MSE** | 0.00323 | 0.05951 | | **1.00e-09*** | 0.01475 | 0.18555 | **2.86e-09*** | 0.02327 | | 0.50479 | | **5.89e-10*** | | | 0.05829 | | | 1.10086 | | | **1.14e-10*** |
|  | **RMSE** | 0.05685 | 0.24395 | | **1.00e-09*** | 0.12144 | 0.43075 | **2.86e-09*** | 0.15256 | | 0.71049 | | **5.89e-10*** | | | 0.24143 | | | 1.04922 | | | **1.04e-10*** |
|  | **MAE** | 0.04819 | 0.20427 | | **1.10e-09*** | 0.09015 | 0.35572 | **3.40e-09*** | 0.119 | | 0.55655 | | **5.89e-10*** | | | 0.17522 | | | 0.80392 | | | **1.14e-10*** |
| **AMP vs. rSO2_R** | **MSE** | 0.00323 | 0.0582 | | **8.31e-09*** | 0.01475 | 0.20985 | **1.01e-08*** | 0.02327 | | 0.5354 | | **2.59e-09*** | | | 0.05829 | | | 1.2396 | | | **5.15e-10*** |
|  | **RMSE** | 0.05685 | 0.24124 | | **8.31e-09*** | 0.12144 | 0.45805 | **1.01e-08*** | 0.15256 | | 0.73123 | | **2.59e-09*** | | | 0.24143 | | | 1.11294 | | | **4.64e-10*** |
|  | **MAE** | 0.04819 | 0.20723 | | **8.31e-09*** | 0.09015 | 0.38 | **6.86e-09*** | 0.119 | | 0.58466 | | **2.12e-09*** | | | 0.17522 | | | 0.83689 | | | **5.70e-10*** |
|  | | **Rotterdam CT Grade of 5** | | | | | | | | | | | | | | | | | | | | |
| **ICP vs. rSO2_L** | **MSE** | 0.08758 | 0.05205 | | 0.12156 | 0.31513 | 0.17965 | 0.43198 | 0.71481 | | 0.56534 | | 0.65742 | | | 1.40184 | | | 1.17138 | | | 0.51843 |
|  | **RMSE** | 0.29592 | 0.22815 | | 0.12156 | 0.56137 | 0.42384 | 0.43198 | 0.84534 | | 0.75188 | | 0.65742 | | | 1.18385 | | | 1.08217 | | | 0.51843 |
|  | **MAE** | 0.25665 | 0.193 | | 0.11072 | 0.46656 | 0.35056 | 0.35452 | 0.68722 | | 0.60724 | | 0.65742 | | | 0.88936 | | | 0.81571 | | | 0.72625 |
| **ICP vs. rSO2_R** | **MSE** | 0.08758 | 0.05436 | | 0.07739 | 0.31513 | 0.21365 | 0.16198 | 0.71481 | | 0.49608 | | 0.25315 | | | 1.40184 | | | 1.17218 | | | 0.25985 |
|  | **RMSE** | 0.29592 | 0.23315 | | 0.07739 | 0.56137 | 0.46222 | 0.16683 | 0.84534 | | 0.70422 | | 0.25985 | | | 1.18385 | | | 1.08267 | | | 0.25985 |
|  | **MAE** | 0.25665 | 0.20013 | | 0.05216 | 0.46656 | 0.40274 | 0.13096 | 0.68722 | | 0.57763 | | 0.28787 | | | 0.88936 | | | 0.78265 | | | 0.30261 |
| **AMP vs. rSO2_L** | **MSE** | 0.00215 | 0.05205 | | **1.69e-10*** | 0.00733 | 0.17965 | **3.41e-10*** | 0.01618 | | 0.56534 | | **2.53e-10*** | | | 0.02477 | | | 1.17138 | | | **3.09e-10*** |
|  | **RMSE** | 0.04628 | 0.22815 | | **1.69e-10*** | 0.08503 | 0.42384 | **3.41e-10*** | 0.12707 | | 0.75188 | | **2.28e-10*** | | | 0.15739 | | | 1.08217 | | | **3.41e-10*** |
|  | **MAE** | 0.0391 | 0.193 | | **1.69e-10*** | 0.06948 | 0.35056 | **3.77e-10*** | 0.09208 | | 0.60724 | | **2.53e-10*** | | | 0.1233 | | | 0.81571 | | | **3.41e-10*** |
| **AMP vs. rSO2_R** | **MSE** | 0.00215 | 0.05436 | | **1.11e-08*** | 0.00733 | 0.21365 | **1.76e-08*** | 0.01618 | | 0.49608 | | **1.33e-08*** | | | 0.02477 | | | 1.17218 | | | **5.90e-10*** |
|  | **RMSE** | 0.04628 | 0.23315 | | **1.11e-08*** | 0.08503 | 0.46222 | **1.76e-08*** | 0.12707 | | 0.70422 | | **1.01e-08*** | | | 0.15739 | | | 1.08267 | | | **5.90e-10*** |
|  | **MAE** | 0.0391 | 0.20013 | | **1.33e-08*** | 0.06948 | 0.40274 | **1.94e-08*** | 0.09208 | | 0.57763 | | **1.01e-08*** | | | 0.1233 | | | 0.78265 | | | **7.22e-10*** |
|  | | **Rotterdam CT Grade of 6** | | | | | | | | | | | | | | | | | | | | |
| **ICP vs. rSO2_L** | **MSE** | 0.03098 | 0.07658 | | 0.18709 | 0.10224 | 0.26756 | 0.05422 | 0.36571 | | 0.60676 | | 0.10688 | | | 0.80632 | | | 1.57001 | | | 0.06764 |
|  | **RMSE** | 0.17599 | 0.27658 | | 0.18709 | 0.31972 | 0.51537 | 0.05182 | 0.60456 | | 0.77592 | | 0.10688 | | | 0.89779 | | | 1.25231 | | | 0.06476 |
|  | **MAE** | 0.1489 | 0.2293 | | 0.18065 | 0.26267 | 0.41532 | **0.04515** | 0.43682 | | 0.62884 | | **0.04309** | | | 0.61358 | | | 0.95034 | | | **0.04729** |
| **ICP vs. rSO2_R** | **MSE** | 0.03098 | 0.05399 | | 0.66621 | 0.10224 | 0.19654 | 0.13466 | 0.36571 | | 0.63534 | | 0.08612 | | | 0.80632 | | | 1.50464 | | | 0.06508 |
|  | **RMSE** | 0.17599 | 0.23228 | | 0.66621 | 0.31972 | 0.44321 | 0.11655 | 0.60456 | | 0.797 | | 0.08612 | | | 0.89779 | | | 1.22543 | | | 0.06508 |
|  | **MAE** | 0.1489 | 0.1971 | | 0.52656 | 0.26267 | 0.3868 | 0.08612 | 0.43682 | | 0.63972 | | 0.05509 | | | 0.61358 | | | 0.91391 | | | 0.05280 |
| **AMP vs. rSO2_L** | **MSE** | 0.00051 | 0.07658 | | **6.84e-09*** | 0.00205 | 0.26756 | **3.80e-09*** | 0.00477 | | 0.60676 | | **5.41e-09*** | | | 0.00919 | | | 1.57001 | | | **3.38e-09*** |
|  | **RMSE** | 0.02261 | 0.27658 | | **6.84e-09*** | 0.04521 | 0.51537 | **3.80e-09*** | 0.06904 | | 0.77592 | | **5.41e-09*** | | | 0.09583 | | | 1.25231 | | | **3.38e-09*** |
|  | **MAE** | 0.01916 | 0.2293 | | **6.84e-09*** | 0.0366 | 0.41532 | **3.38e-09*** | 0.05333 | | 0.62884 | | **4.81e-09*** | | | 0.07677 | | | 0.95034 | | | **3.38e-09*** |
| **AMP vs. rSO2_R** | **MSE** | 0.00051 | 0.05399 | | **2.58e-09*** | 0.00205 | 0.19654 | **1.04e-09*** | 0.00477 | | 0.63534 | | **7.35e-10*** | | | 0.00919 | | | 1.50464 | | | **8.26e-10*** |
|  | **RMSE** | 0.02261 | 0.23228 | | **2.58e-09*** | 0.04521 | 0.44321 | **1.04e-09*** | 0.06904 | | 0.797 | | **7.35e-10*** | | | 0.09583 | | | 1.22543 | | | **8.26e-10*** |
|  | **MAE** | 0.01916 | 0.1971 | | **3.23e-09*** | 0.0366 | 0.3868 | **1.04e-09*** | 0.05333 | | 0.63972 | | **9.26e-10*** | | | 0.07677 | | | 0.91391 | | | **8.26e-10*** |
|  | | **Marshall CT Grade of II** | | | | | | | | | | | | | | | | | | | | |
| **ICP vs. rSO2_L** | **MSE** | 0.19094 | 0.13382 | | 0.38273 | 0.65973 | 0.61227 | 0.08086 | 1.42436 | | 1.0568 | | 0.08086 | | | 2.24262 | | | 2.4349 | | | 1.00000 |
|  | **RMSE** | 0.43697 | 0.36581 | | 0.38273 | 0.81224 | 0.78246 | 0.08086 | 1.19327 | | 1.02801 | | 0.08086 | | | 1.49754 | | | 1.55972 | | | 1.00000 |
|  | **MAE** | 0.37368 | 0.309 | | 0.38273 | 0.67341 | 0.61415 | 0.19043 | 0.87182 | | 0.84899 | | 0.66252 | | | 1.26425 | | | 1.25811 | | | 1.00000 |
| **ICP vs. rSO2_R** | **MSE** | 0.19094 | 0.14644 | | 0.38273 | 0.65973 | 0.71845 | 1.00000 | 1.42436 | | 1.21778 | | 0.38273 | | | 2.24262 | | | 2.25396 | | | 1.00000 |
|  | **RMSE** | 0.43697 | 0.38267 | | 0.38273 | 0.81224 | 0.84761 | 1.00000 | 1.19327 | | 1.10353 | | 0.38273 | | | 1.49754 | | | 1.50132 | | | 1.00000 |
|  | **MAE** | 0.37368 | 0.3373 | | 0.38273 | 0.67341 | 0.68504 | 1.00000 | 0.87182 | | 0.88528 | | 1.00000 | | | 1.26425 | | | 1.14557 | | | 0.66252 |
| **AMP vs. rSO2_L** | **MSE** | 0.00133 | 0.13382 | | 0.08086 | 0.00929 | 0.61227 | 0.08086 | 0.01797 | | 1.0568 | | 0.08086 | | | 0.02058 | | | 2.4349 | | | 0.08086 |
|  | **RMSE** | 0.03645 | 0.36581 | | 0.08086 | 0.09639 | 0.78246 | 0.08086 | 0.13405 | | 1.02801 | | 0.08086 | | | 0.14345 | | | 1.55972 | | | 0.08086 |
|  | **MAE** | 0.03087 | 0.309 | | 0.08086 | 0.07131 | 0.61415 | 0.08086 | 0.08861 | | 0.84899 | | 0.08086 | | | 0.11432 | | | 1.25811 | | | 0.08086 |
| **AMP vs. rSO2_R** | **MSE** | 0.00133 | 0.14644 | | 0.08086 | 0.00929 | 0.71845 | 0.08086 | 0.01797 | | 1.21778 | | 0.08086 | | | 0.02058 | | | 2.25396 | | | 0.08086 |
|  | **RMSE** | 0.03645 | 0.38267 | | 0.08086 | 0.09639 | 0.84761 | 0.08086 | 0.13405 | | 1.10353 | | 0.08086 | | | 0.14345 | | | 1.50132 | | | 0.08086 |
|  | **MAE** | 0.03087 | 0.3373 | | 0.08086 | 0.07131 | 0.68504 | 0.08086 | 0.08861 | | 0.88528 | | 0.08086 | | | 0.11432 | | | 1.14557 | | | 0.08086 |
|  | | **Marshall CT Grade of III** | | | | | | | | | | | | | | | | | | | | |
| **ICP vs. rSO2_L** | **MSE** | 0.1291 | 0.07009 | | **3.87e-05*** | 0.50078 | 0.25302 | **1.64e-04*** | 1.20879 | | 0.56906 | | **0.00251** | | | 3.09067 | | | 1.70609 | | | **0.00162** |
|  | **RMSE** | 0.3593 | 0.26471 | | **4.67e-05*** | 0.70764 | 0.50292 | **1.64e-04*** | 1.09945 | | 0.75434 | | **0.00251** | | | 1.75555 | | | 1.30606 | | | **0.00168** |
|  | **MAE** | 0.30506 | 0.22353 | | **4.89e-05*** | 0.59139 | 0.39746 | **5.95e-04*** | 0.8902 | | 0.62494 | | **0.00134** | | | 1.27006 | | | 1.02092 | | | **0.00168** |
| **ICP vs. rSO2_R** | **MSE** | 0.1291 | 0.07515 | | **1.00e-04*** | 0.50078 | 0.21659 | **0.00136** | 1.20879 | | 0.6198 | | **0.00386** | | | 3.09067 | | | 1.51638 | | | **0.00372** |
|  | **RMSE** | 0.3593 | 0.27413 | | **1.05e-04*** | 0.70764 | 0.46539 | **0.00136** | 1.09945 | | 0.78728 | | **0.00372** | | | 1.75555 | | | 1.23141 | | | **0.00358** |
|  | **MAE** | 0.30506 | 0.23 | | **1.00e-04*** | 0.59139 | 0.39329 | **0.00168** | 0.8902 | | 0.61047 | | **0.00214** | | | 1.27006 | | | 0.94627 | | | **0.00214** |
| **AMP vs. rSO2_L** | **MSE** | 0.0038 | 0.07009 | | **2.35e-13*** | 0.01435 | 0.25302 | **1.45e-13*** | 0.03161 | | 0.56906 | | **1.45e-13*** | | | 0.05922 | | | 1.70609 | | | **1.45e-13*** |
|  | **RMSE** | 0.06167 | 0.26471 | | **2.35e-13*** | 0.11977 | 0.50292 | **1.45e-13*** | 0.1778 | | 0.75434 | | **1.45e-13*** | | | 0.24333 | | | 1.30606 | | | **1.45e-13*** |
|  | **MAE** | 0.05233 | 0.22353 | | **2.35e-13*** | 0.0947 | 0.39746 | **1.45e-13*** | 0.13247 | | 0.62494 | | **1.45e-13*** | | | 0.18188 | | | 1.02092 | | | **1.45e-13*** |
| **AMP vs. rSO2_R** | **MSE** | 0.0038 | 0.07515 | | **1.25e-12*** | 0.01435 | 0.21659 | **1.25e-12*** | 0.03161 | | 0.6198 | | **1.25e-12*** | | | 0.05922 | | | 1.51638 | | | **1.25e-12*** |
|  | **RMSE** | 0.06167 | 0.27413 | | **1.25e-12*** | 0.11977 | 0.46539 | **1.25e-12*** | 0.1778 | | 0.78728 | | **1.25e-12*** | | | 0.24333 | | | 1.23141 | | | **1.25e-12*** |
|  | **MAE** | 0.05233 | 0.23 | | **1.25e-12*** | 0.0947 | 0.39329 | **1.25e-12*** | 0.13247 | | 0.61047 | | **1.25e-12*** | | | 0.18188 | | | 0.94627 | | | **1.25e-12*** |
|  | | **Marshall CT Grade of IV** | | | | | | | | | | | | | | | | | | | | |
| **ICP vs. rSO2_L** | **MSE** | 0.16663 | 0.05744 | | **0.01198** | 0.60678 | 0.18255 | **0.02618** | 1.55726 | | 0.49213 | | **0.02871** | | | 3.07523 | | | 1.21776 | | | **0.02168** |
|  | **RMSE** | 0.40821 | 0.23967 | | **0.01198** | 0.77822 | 0.42726 | **0.02618** | 1.24116 | | 0.70146 | | **0.02871** | | | 1.75206 | | | 1.10344 | | | **0.02168** |
|  | **MAE** | 0.353 | 0.20233 | | **0.01467** | 0.63728 | 0.3517 | **0.02384** | 1.00437 | | 0.55655 | | **0.02168** | | | 1.30196 | | | 0.81551 | | | **0.03146** |
| **ICP vs. rSO2_R** | **MSE** | 0.16663 | 0.05045 | | **0.01919** | 0.60678 | 0.19226 | **0.02630** | 1.55726 | | 0.48744 | | **0.02913** | | | 3.07523 | | | 0.99759 | | | **0.02913** |
|  | **RMSE** | 0.40821 | 0.22461 | | **0.01919** | 0.77822 | 0.43847 | **0.02630** | 1.24116 | | 0.69814 | | **0.02913** | | | 1.75206 | | | 0.9988 | | | **0.02913** |
|  | **MAE** | 0.353 | 0.18587 | | **0.01722** | 0.63728 | 0.37064 | **0.02913** | 1.00437 | | 0.55946 | | **0.03222** | | | 1.30196 | | | 0.78265 | | | **0.03924** |
| **AMP vs. rSO2_L** | **MSE** | 0.0034 | 0.05744 | | **2.15e-05*** | 0.01315 | 0.18255 | **7.53e-05*** | 0.03104 | | 0.49213 | | **1.83e-05*** | | | 0.06438 | | | 1.21776 | | | **2.84e-06*** |
|  | **RMSE** | 0.05804 | 0.23967 | | **2.15e-05*** | 0.11441 | 0.42726 | **7.53e-05*** | 0.17478 | | 0.70146 | | **1.83e-05*** | | | 0.25016 | | | 1.10344 | | | **2.84e-06*** |
|  | **MAE** | 0.04961 | 0.20233 | | **2.53e-05*** | 0.09351 | 0.3517 | **5.55e-05*** | 0.13775 | | 0.55655 | | **1.83e-05*** | | | 0.19452 | | | 0.81551 | | | **4.03e-06*** |
| **AMP vs. rSO2_R** | **MSE** | 0.0034 | 0.05045 | | **8.04e-05*** | 0.01315 | 0.19226 | **1.82e-04*** | 0.03104 | | 0.48744 | | **4.07e-05*** | | | 0.06438 | | | 0.99759 | | | **1.68e-05*** |
|  | **RMSE** | 0.05804 | 0.22461 | | **8.04e-05*** | 0.11441 | 0.43847 | **1.82e-04*** | 0.17478 | | 0.69814 | | **4.07e-05*** | | | 0.25016 | | | 0.9988 | | | **1.40e-05*** |
|  | **MAE** | 0.04961 | 0.18587 | | **8.04e-05*** | 0.09351 | 0.37064 | **1.32e-04*** | 0.13775 | | 0.55946 | | **4.07e-05*** | | | 0.19452 | | | 0.78265 | | | **2.01e-05*** |
|  | | **Marshall CT Grade of V** | | | | | | | | | | | | | | | | | | | | |
| **ICP vs. rSO2_L** | **MSE** | 0.07651 | 0.0649 | | 0.97609 | 0.25434 | 0.24726 | 0.39469 | 0.50519 | | 0.57036 | | 0.37180 | | | 0.96175 | | | 1.19712 | | | 0.28062 |
|  | **RMSE** | 0.27661 | 0.25476 | | 0.97609 | 0.48915 | 0.49716 | 0.39136 | 0.71077 | | 0.75522 | | 0.37180 | | | 0.97359 | | | 1.0938 | | | 0.27268 |
|  | **MAE** | 0.24333 | 0.21287 | | 0.96175 | 0.39721 | 0.40375 | 0.41840 | 0.55277 | | 0.62144 | | 0.21030 | | | 0.73689 | | | 0.84031 | | | 0.18330 |
| **ICP vs. rSO2_R** | **MSE** | 0.07651 | 0.05436 | | 0.21469 | 0.25434 | 0.20267 | 0.67479 | 0.50519 | | 0.48778 | | 0.99044 | | | 0.96175 | | | 1.24267 | | | 0.58133 |
|  | **RMSE** | 0.27661 | 0.23315 | | 0.21469 | 0.48915 | 0.45007 | 0.69682 | 0.71077 | | 0.69841 | | 0.98087 | | | 0.97359 | | | 1.08905 | | | 0.58133 |
|  | **MAE** | 0.24333 | 0.19933 | | 0.21030 | 0.39721 | 0.38175 | 0.85729 | 0.55277 | | 0.56805 | | 0.71464 | | | 0.73689 | | | 0.81777 | | | 0.50584 |
| **AMP vs. rSO2_L** | **MSE** | 0.00136 | 0.0649 | | **8.30e-18*** | 0.00524 | 0.24726 | **3.98e-18*** | 0.0135 | | 0.57036 | | **6.06e-18*** | | | 0.0208 | | | 1.19712 | | | **2.75e-18*** |
|  | **RMSE** | 0.03692 | 0.25476 | | **8.30e-18*** | 0.07236 | 0.49716 | **3.98e-18*** | 0.11618 | | 0.75522 | | **4.91e-18*** | | | 0.14418 | | | 1.0938 | | | **2.75e-18*** |
|  | **MAE** | 0.03033 | 0.21287 | | **1.08e-17*** | 0.05769 | 0.40375 | **4.66e-18*** | 0.08807 | | 0.62144 | | **5.18e-18*** | | | 0.11652 | | | 0.84031 | | | **2.47e-18*** |
| **AMP vs. rSO2_R** | **MSE** | 0.00136 | 0.05436 | | **2.80e-16*** | 0.00524 | 0.20267 | **8.82e-17*** | 0.0135 | | 0.48778 | | **4.79e-17*** | | | 0.0208 | | | 1.24267 | | | **9.99e-19*** |
|  | **RMSE** | 0.03692 | 0.23315 | | **2.80e-16*** | 0.07236 | 0.45007 | **8.82e-17*** | 0.11618 | | 0.69841 | | **4.11e-17*** | | | 0.14418 | | | 1.08905 | | | **9.99e-19*** |
|  | **MAE** | 0.03033 | 0.19933 | | **3.25e-16*** | 0.05769 | 0.38175 | **7.97e-17*** | 0.08807 | | 0.56805 | | **4.33e-17*** | | | 0.11652 | | | 0.81777 | | | **1.38e-18*** |
|  | | **Under 40** | | | | | | | | | | | | | | | | | | | | |
| **ICP vs. rSO2_L** | **MSE** | 0.12601 | 0.0683 | | **0.03085** | 0.42327 | 0.28893 | 0.18733 | 0.98061 | | 0.76301 | | 0.20739 | | | 2.09056 | | | 1.69526 | | | 0.23905 |
|  | **RMSE** | 0.35498 | 0.26126 | | **0.03237** | 0.65059 | 0.53722 | 0.18733 | 0.99025 | | 0.87302 | | 0.20739 | | | 1.44481 | | | 1.30197 | | | 0.24162 |
|  | **MAE** | 0.30321 | 0.22243 | | **0.03562** | 0.52422 | 0.4287 | 0.20739 | 0.77373 | | 0.67731 | | 0.24682 | | | 1.11753 | | | 1.02092 | | | 0.31224 |
| **ICP vs. rSO2_R** | **MSE** | 0.12601 | 0.08215 | | **0.04975** | 0.42327 | 0.32261 | 0.16994 | 0.98061 | | 0.81255 | | 0.20915 | | | 2.09056 | | | 1.83916 | | | 0.25150 |
|  | **RMSE** | 0.35498 | 0.28629 | | 0.05147 | 0.65059 | 0.56784 | 0.17221 | 0.99025 | | 0.90043 | | 0.20652 | | | 1.44481 | | | 1.35315 | | | 0.25150 |
|  | **MAE** | 0.30321 | 0.2485 | | **0.04487** | 0.52422 | 0.4656 | 0.21990 | 0.77373 | | 0.69793 | | 0.19878 | | | 1.11753 | | | 0.99601 | | | 0.23105 |
| **AMP vs. rSO2_L** | **MSE** | 0.00263 | 0.0683 | | **1.22e-18*** | 0.00926 | 0.28893 | **1.16e-18*** | 0.0187 | | 0.76301 | | **1.16e-18*** | | | 0.04317 | | | 1.69526 | | | **1.16e-18*** |
|  | **RMSE** | 0.05131 | 0.26126 | | **1.22e-18*** | 0.0962 | 0.53722 | **1.16e-18*** | 0.13673 | | 0.87302 | | **1.16e-18*** | | | 0.20774 | | | 1.30197 | | | **1.16e-18*** |
|  | **MAE** | 0.04383 | 0.22243 | | **1.22e-18*** | 0.07863 | 0.4287 | **1.16e-18*** | 0.11309 | | 0.67731 | | **1.16e-18*** | | | 0.15222 | | | 1.02092 | | | **1.16e-18*** |
| **AMP vs. rSO2_R** | **MSE** | 0.00263 | 0.08215 | | **6.17e-17*** | 0.00926 | 0.32261 | **6.17e-17*** | 0.0187 | | 0.81255 | | **6.57e-17*** | | | 0.04317 | | | 1.83916 | | | **4.26e-17*** |
|  | **RMSE** | 0.05131 | 0.28629 | | **6.17e-17*** | 0.0962 | 0.56784 | **6.17e-17*** | 0.13673 | | 0.90043 | | **6.57e-17*** | | | 0.20774 | | | 1.35315 | | | **4.26e-17*** |
|  | **MAE** | 0.04383 | 0.2485 | | **6.57e-17*** | 0.07863 | 0.4656 | **5.80e-17*** | 0.11309 | | 0.69793 | | **6.57e-17*** | | | 0.15222 | | | 0.99601 | | | **4.26e-17*** |
|  | | **40 - 60** | | | | | | | | | | | | | | | | | | | | |
| **ICP vs. rSO2_L** | **MSE** | 0.11648 | 0.07927 | | **0.03992** | 0.4367 | 0.21898 | 0.16489 | 0.86108 | | 0.56534 | | 0.33590 | | | 1.71483 | | | 1.24737 | | | 0.43220 |
|  | **RMSE** | 0.34117 | 0.28154 | | **0.03992** | 0.66083 | 0.46723 | 0.16489 | 0.92783 | | 0.75188 | | 0.33590 | | | 1.30904 | | | 1.11522 | | | 0.45071 |
|  | **MAE** | 0.29007 | 0.23403 | | **0.03426** | 0.50881 | 0.37176 | 0.14671 | 0.74958 | | 0.57548 | | 0.37937 | | | 0.9945 | | | 0.84802 | | | 0.63597 |
| **ICP vs. rSO2_R** | **MSE** | 0.11648 | 0.05428 | | **0.00421** | 0.4367 | 0.19041 | **0.02470** | 0.86108 | | 0.48744 | | 0.10764 | | | 1.71483 | | | 0.99759 | | | 0.14423 |
|  | **RMSE** | 0.34117 | 0.23298 | | **0.00421** | 0.66083 | 0.43636 | **0.02470** | 0.92783 | | 0.69814 | | 0.10764 | | | 1.30904 | | | 0.9988 | | | 0.15626 |
|  | **MAE** | 0.29007 | 0.19907 | | **0.00421** | 0.50881 | 0.37447 | **0.02470** | 0.74958 | | 0.55946 | | 0.12230 | | | 0.9945 | | | 0.78854 | | | 0.23219 |
| **AMP vs. rSO2_L** | **MSE** | 0.00246 | 0.07927 | | **3.16e-13*** | 0.00796 | 0.21898 | **1.98e-13*** | 0.01698 | | 0.56534 | | **3.16e-13*** | | | 0.0261 | | | 1.24737 | | | **1.84e-13*** |
|  | **RMSE** | 0.04956 | 0.28154 | | **3.16e-13*** | 0.08901 | 0.46723 | **1.98e-13*** | 0.13026 | | 0.75188 | | **3.41e-13*** | | | 0.16151 | | | 1.11522 | | | **1.84e-13*** |
|  | **MAE** | 0.04173 | 0.23403 | | **3.41e-13*** | 0.06827 | 0.37176 | **2.32e-13*** | 0.0884 | | 0.57548 | | **3.16e-13*** | | | 0.12388 | | | 0.84802 | | | **1.45e-13*** |
| **AMP vs. rSO2_R** | **MSE** | 0.00246 | 0.05428 | | **1.11e-11*** | 0.00796 | 0.19041 | **3.92e-12*** | 0.01698 | | 0.48744 | | **3.37e-12*** | | | 0.0261 | | | 0.99759 | | | **1.29e-13*** |
|  | **RMSE** | 0.04956 | 0.23298 | | **1.11e-11*** | 0.08901 | 0.43636 | **3.92e-12*** | 0.13026 | | 0.69814 | | **3.37e-12*** | | | 0.16151 | | | 0.9988 | | | **1.29e-13*** |
|  | **MAE** | 0.04173 | 0.19907 | | **1.20e-11*** | 0.06827 | 0.37447 | **3.37e-12*** | 0.0884 | | 0.55946 | | **3.37e-12*** | | | 0.12388 | | | 0.78854 | | | **1.51e-13*** |
|  | | **Over 60** | | | | | | | | | | | | | | | | | | | | |
| **ICP vs. rSO2_L** | **MSE** | 0.08039 | 0.05494 | | 0.06884 | 0.27458 | 0.18555 | 0.12946 | 0.63537 | | 0.50053 | | 0.12946 | | | 1.04773 | | | 0.96486 | | | 0.17237 |
|  | **RMSE** | 0.28353 | 0.23438 | | 0.06884 | 0.50433 | 0.43075 | 0.12946 | 0.79708 | | 0.70748 | | 0.12946 | | | 1.02337 | | | 0.98077 | | | 0.17237 |
|  | **MAE** | 0.24444 | 0.19093 | | 0.09534 | 0.41655 | 0.37141 | 0.16456 | 0.60122 | | 0.58855 | | 0.20640 | | | 0.82114 | | | 0.77299 | | | 0.21562 |
| **ICP vs. rSO2_R** | **MSE** | 0.08039 | 0.05259 | | **0.03867** | 0.27458 | 0.20267 | 0.05701 | 0.63537 | | 0.34504 | | 0.07802 | | | 1.04773 | | | 0.71689 | | | 0.10496 |
|  | **RMSE** | 0.28353 | 0.22933 | | **0.03867** | 0.50433 | 0.45007 | 0.06684 | 0.79708 | | 0.5874 | | 0.08207 | | | 1.02337 | | | 0.84628 | | | 0.10496 |
|  | **MAE** | 0.24444 | 0.19487 | | **0.04331** | 0.41655 | 0.37277 | 0.13263 | 0.60122 | | 0.47349 | | 0.18823 | | | 0.82114 | | | 0.6253 | | | 0.07802 |
| **AMP vs. rSO2_L** | **MSE** | 0.00301 | 0.05494 | | **1.38e-05*** | 0.01475 | 0.18555 | **2.44e-05*** | 0.01834 | | 0.50053 | | **8.68e-06*** | | | 0.03646 | | | 0.96486 | | | **2.29e-06*** |
|  | **RMSE** | 0.0549 | 0.23438 | | **1.38e-05*** | 0.12144 | 0.43075 | **2.44e-05*** | 0.13544 | | 0.70748 | | **4.79e-06*** | | | 0.19096 | | | 0.98077 | | | **2.29e-06*** |
|  | **MAE** | 0.04742 | 0.19093 | | **1.74e-05*** | 0.09125 | 0.37141 | **2.44e-05*** | 0.10768 | | 0.58855 | | **4.79e-06*** | | | 0.13997 | | | 0.77299 | | | **2.03e-06*** |
| **AMP vs. rSO2_R** | **MSE** | 0.00301 | 0.05259 | | **1.24e-05*** | 0.01475 | 0.20267 | **8.96e-06*** | 0.01834 | | 0.34504 | | **1.84e-06*** | | | 0.03646 | | | 0.71689 | | | **6.31e-07*** |
|  | **RMSE** | 0.0549 | 0.22933 | | **1.24e-05*** | 0.12144 | 0.45007 | **8.96e-06*** | 0.13544 | | 0.5874 | | **1.64e-06*** | | | 0.19096 | | | 0.84628 | | | **6.31e-07*** |
|  | **MAE** | 0.04742 | 0.19487 | | **1.24e-05*** | 0.09125 | 0.37277 | **8.03e-06*** | 0.10768 | | 0.47349 | | **1.46e-06*** | | | 0.13997 | | | 0.6253 | | | **6.31e-07*** |
|  | | **Male** | | | | | | | | | | | | | | | | | | | | |
| **ICP vs. rSO2_L** | **MSE** | 0.1156 | 0.06519 | | **0.00113** | 0.38636 | 0.23612 | **0.02030** | 0.85445 | | 0.56906 | | 0.05765 | | | 1.78857 | | | 1.34879 | | | 0.07478 |
|  | **RMSE** | 0.33997 | 0.25531 | | **0.00119** | 0.62154 | 0.48587 | **0.02126** | 0.9243 | | 0.75434 | | 0.05804 | | | 1.33734 | | | 1.16118 | | | 0.07622 |
|  | **MAE** | 0.2894 | 0.21767 | | **0.00105** | 0.50602 | 0.39436 | **0.02077** | 0.74277 | | 0.61946 | | 0.07152 | | | 1.02767 | | | 0.87746 | | | 0.16412 |
| **ICP vs. rSO2_R** | **MSE** | 0.1156 | 0.06179 | | **3.73e-04*** | 0.38636 | 0.21596 | **0.01162** | 0.85445 | | 0.58028 | | 0.06258 | | | 1.78857 | | | 1.27767 | | | 0.09956 |
|  | **RMSE** | 0.33997 | 0.24857 | | **3.87e-04*** | 0.62154 | 0.46472 | **0.01260** | 0.9243 | | 0.76175 | | 0.06258 | | | 1.33734 | | | 1.13034 | | | 0.10153 |
|  | **MAE** | 0.2894 | 0.21873 | | **3.78e-04*** | 0.50602 | 0.39329 | **0.02281** | 0.74277 | | 0.60996 | | 0.08208 | | | 1.02767 | | | 0.87351 | | | 0.13324 |
| **AMP vs. rSO2_L** | **MSE** | 0.0027 | 0.06519 | | **8.46e-29*** | 0.0098 | 0.23612 | **1.09e-27*** | 0.01827 | | 0.56906 | | **3.01e-28*** | | | 0.03554 | | | 1.34879 | | | **5.71e-29*** |
|  | **RMSE** | 0.05196 | 0.25531 | | **8.46e-29*** | 0.09898 | 0.48587 | **1.09e-27*** | 0.13516 | | 0.75434 | | **2.04e-28*** | | | 0.1885 | | | 1.16118 | | | **5.53e-29*** |
|  | **MAE** | 0.04388 | 0.21767 | | **9.04e-29*** | 0.07917 | 0.39436 | **1.16e-27*** | 0.10762 | | 0.61946 | | **2.65e-28*** | | | 0.1461 | | | 0.87746 | | | **5.01e-29*** |
| **AMP vs. rSO2_R** | **MSE** | 0.0027 | 0.06179 | | **1.82e-25*** | 0.0098 | 0.21596 | **8.20e-25*** | 0.01827 | | 0.58028 | | **1.20e-25*** | | | 0.03554 | | | 1.27767 | | | **7.92e-28*** |
|  | **RMSE** | 0.05196 | 0.24857 | | **1.82e-25*** | 0.09898 | 0.46472 | **8.20e-25*** | 0.13516 | | 0.76175 | | **8.58e-26*** | | | 0.1885 | | | 1.13034 | | | **7.65e-28*** |
|  | **MAE** | 0.04388 | 0.21873 | | **2.38e-25*** | 0.07917 | 0.39329 | **5.52e-25*** | 0.10762 | | 0.60996 | | **8.30e-26*** | | | 0.1461 | | | 0.87351 | | | **1.33e-27*** |
|  | | **Female** | | | | | | | | | | | | | | | | | | | | |
| **ICP vs. rSO2_L** | **MSE** | 0.11474 | 0.05786 | | 0.26985 | 0.43777 | 0.17801 | 0.44597 | 0.96989 | | 0.49213 | | 0.41929 | | | 1.82725 | | | 1.20702 | | | 0.50213 |
|  | **RMSE** | 0.33866 | 0.24054 | | 0.26985 | 0.66162 | 0.42191 | 0.41929 | 0.98481 | | 0.70146 | | 0.41929 | | | 1.35116 | | | 1.09865 | | | 0.50213 |
|  | **MAE** | 0.29023 | 0.20513 | | 0.26010 | 0.5023 | 0.3517 | 0.48775 | 0.77551 | | 0.60153 | | 0.45966 | | | 1.06391 | | | 0.86678 | | | 0.48775 |
| **ICP vs. rSO2_R** | **MSE** | 0.11474 | 0.05295 | | 0.05965 | 0.43777 | 0.16629 | **0.04661** | 0.96989 | | 0.32191 | | 0.05965 | | | 1.82725 | | | 0.74718 | | | 0.06259 |
|  | **RMSE** | 0.33866 | 0.22999 | | 0.05965 | 0.66162 | 0.40779 | **0.04661** | 0.98481 | | 0.5673 | | 0.05965 | | | 1.35116 | | | 0.86439 | | | 0.06259 |
|  | **MAE** | 0.29023 | 0.19587 | | 0.05411 | 0.5023 | 0.34159 | 0.05682 | 0.77551 | | 0.46352 | | 0.07914 | | | 1.06391 | | | 0.71076 | | | 0.07914 |
| **AMP vs. rSO2_L** | **MSE** | 0.00181 | 0.05786 | | **1.39e-07*** | 0.00704 | 0.17801 | **2.33e-08*** | 0.01653 | | 0.49213 | | **1.79e-08*** | | | 0.02909 | | | 1.20702 | | | **1.20e-08*** |
|  | **RMSE** | 0.04254 | 0.24054 | | **1.39e-07*** | 0.08361 | 0.42191 | **2.33e-08*** | 0.12854 | | 0.70146 | | **1.79e-08*** | | | 0.17027 | | | 1.09865 | | | **1.20e-08*** |
|  | **MAE** | 0.03628 | 0.20513 | | **1.57e-07*** | 0.06719 | 0.3517 | **2.05e-08*** | 0.10207 | | 0.60153 | | **1.79e-08*** | | | 0.13301 | | | 0.86678 | | | **1.38e-08*** |
| **AMP vs. rSO2_R** | **MSE** | 0.00181 | 0.05295 | | **9.77e-08*** | 0.00704 | 0.16629 | **9.73e-09*** | 0.01653 | | 0.32191 | | **5.17e-09*** | | | 0.02909 | | | 0.74718 | | | **5.17e-09*** |
|  | **RMSE** | 0.04254 | 0.22999 | | **9.77e-08*** | 0.08361 | 0.40779 | **9.73e-09*** | 0.12854 | | 0.5673 | | **5.17e-09*** | | | 0.17027 | | | 0.86439 | | | **5.17e-09*** |
|  | **MAE** | 0.03628 | 0.19587 | | **9.77e-08*** | 0.06719 | 0.34159 | **8.58e-09*** | 0.10207 | | 0.46352 | | **5.17e-09*** | | | 0.13301 | | | 0.71076 | | | **5.17e-09*** |

**Appendix D.**

*AMP* pulse amplitude of intracranial pressure, *ICP* intracranial pressure, *MAE* mean absolute error, *MSE* mean squared error, *RMSE* root mean squared error, *rSO_2__L* left sided regional oxygen saturation, rSO_2_*_R* right sided regional oxygen saturation.

**Table D.** Population summary of Pearson correlation analysis across different window sizes and temporal resolutions. Bolded values denote significant p-values prior to Bonferroni analysis, and an asterisk denotes p-values which remain significant post Bonferroni analysis.

|  | |  | | **Median (IQR)** | | | | | | |
| --- | --- | --- | --- | --- | --- | --- | --- | --- | --- | --- |
|  |  | | **1-Second Temporal Resolution** | | | | | | | |
| **Physiologic Relationship** | **Value** | | **1min** | | **5min** | **15min** | **30min** | **1hr** | **3hr** | **6hr** |
| ICP vs rSO_2__L | r | | 0.039 (-0.161–0.253) | | 0.045 (-0.097–0.207) | 0.046 (-0.119–0.228) | 0.048 (-0.149–0.263) | 0.058 (-0.171–0.306) | 0.077 (-0.148–0.320) | 0.090 (-0.139–0.309) |
|  | p | | 0.120 (0.005–0.478) | | **0.010** (0.000–0.259) | **0.000*** (0.000–0.032) | **0.000*** (0.000–0.000) | **0.000*** (0.000–0.000) | **0.000*** (0.000–0.000) | **0.000*** (0.000–0.000) |
| ICP vs rSO_2__R | r | | 0.042 (-0.156–0.256) | | 0.049 (-0.093–0.214) | 0.050 (-0.114–0.237) | 0.058 (-0.148–0.270) | 0.069 (-0.172–0.317) | 0.098 (-0.143–0.349) | 0.098 (-0.129–0.337) |
|  | p | | 0.120 (0.005–0.479) | | **0.009** (0.000–0.254) | **0.000*** (0.000–0.029) | **0.000*** (0.000–0.000) | **0.000*** (0.000–0.000) | **0.000*** (0.000–0.000) | **0.000*** (0.000–0.000) |
| AMP vs rSO_2__L | r | | 0.186 (-0.105–0.459) | | 0.125 (-0.072–0.339) | 0.088 (-0.117–0.309) | 0.063 (-0.164–0.303) | 0.045 (-0.205–0.300) | 0.041 (-0.202–0.273) | 0.048 (-0.188–0.287) |
|  | p | | **0.017** (0.000–0.262) | | **0.000*** (0.000–0.095) | **0.000*** (0.000–0.006) | **0.000*** (0.000–0.000) | **0.000*** (0.000–0.000) | **0.000*** (0.000–0.000) | **0.000*** (0.000–0.000) |
| AMP vs rSO_2__R | r | | 0.191 (-0.097–0.463) | | 0.131 (-0.067–0.349) | 0.093 (-0.110–0.321) | 0.069 (-0.161–0.312) | 0.051 (-0.202–0.305) | 0.048 (-0.194–0.298) | 0.063 (-0.176–0.301) |
|  | p | | **0.017** (0.000–0.260) | | **0.000*** (0.000–0.088) | **0.000*** (0.000–0.007) | **0.000*** (0.000–0.000) | **0.000*** (0.000–0.000) | **0.000*** (0.000–0.000) | **0.000*** (0.000–0.000) |
|  |  | | **10-Second Temporal Resolution** | | | | | | | |
| ICP vs rSO_2__L | r | | 0.157 (-0.273–0.527) | | 0.115 (-0.129–0.349) | 0.095 (-0.158–0.336) | 0.081 (-0.189–0.350) | 0.080 (-0.206–0.365) | 0.083 (-0.161–0.349) | 0.092 (-0.139–0.324) |
|  | p | | 0.409 (0.167–0.693) | | 0.182 (0.019–0.534) | **0.018** (0.000–0.282) | **0.000*** (0.000–0.109) | **0.000*** (0.000–0.025) | **0.000*** (0.000–0.001) | **0.000*** (0.000–0.000) |
| ICP vs rSO_2__R | r | | 0.164 (-0.263–0.530) | | 0.124 (-0.121–0.358) | 0.103 (-0.153–0.348) | 0.094 (-0.191–0.360) | 0.087 (-0.206–0.373) | 0.100 (-0.159–0.375) | 0.103 (-0.137–0.349) |
|  | p | | 0.411 (0.169–0.694) | | 0.175 (0.017–0.528) | **0.015** (0.000–0.271) | **0.000*** (0.000–0.096) | **0.000*** (0.000–0.016) | **0.000*** (0.000–0.000) | **0.000*** (0.000–0.000) |
| AMP vs rSO_2__L | r | | 0.109 (-0.302–0.481) | | 0.078 (-0.147–0.294) | 0.053 (-0.166–0.271) | 0.040 (-0.203–0.273) | 0.029 (-0.236–0.281) | 0.028 (-0.209–0.262) | 0.040 (-0.193–0.278) |
|  | p | | 0.435 (0.188–0.709) | | 0.244 (0.038–0.584) | **0.047** (0.000–0.373) | **0.003** (0.000–0.196) | **0.000*** (0.000–0.063) | **0.000*** (0.000–0.003) | **0.000*** (0.000–0.000) |
| AMP vs rSO_2__R | r | | 0.114 (-0.295–0.483) | | 0.081 (-0.140–0.300) | 0.060 (-0.162–0.277) | 0.039 (-0.206–0.279) | 0.031 (-0.230–0.280) | 0.035 (-0.209–0.277) | 0.050 (-0.179–0.280) |
|  | p | | 0.437 (0.191–0.712) | | 0.241 (0.036–0.589) | **0.045** (0.000–0.372) | **0.002** (0.000–0.184) | **0.000*** (0.000–0.067) | **0.000*** (0.000–0.003) | **0.000*** (0.000–0.000) |
|  |  | | **1-Minute Temporal Resolution** | | | | | | | |
| ICP vs rSO_2__L | r | | - | | 0.149 (-0.430–0.626) | 0.088 (-0.356–0.492) | 0.072 (-0.344–0.462) | 0.069 (-0.303–0.449) | 0.083 (-0.205–0.389) | 0.097 (-0.164–0.355) |
|  | p | | - | | 0.349 (0.121–0.653) | 0.121 (0.009–0.459) | **0.033** (0.000–0.320) | **0.005** (0.000–0.207) | **0.000*** (0.000–0.115) | **0.000*** (0.000–0.048) |
| ICP vs rSO_2__R | r | | - | | 0.156 (-0.428–0.634) | 0.091 (-0.350–0.500) | 0.073 (-0.345–0.474) | 0.078 (-0.311–0.443) | 0.098 (-0.208–0.406) | 0.099 (-0.161–0.373) |
|  | p | | - | | 0.342 (0.119–0.647) | 0.116 (0.008–0.459) | **0.029** (0.000–0.316) | **0.004** (0.000–0.191) | **0.000*** (0.000–0.075) | **0.000*** (0.000–0.034) |
| AMP vs rSO_2__L | r | | - | | 0.109 (-0.445–0.593) | 0.050 (-0.366–0.441) | 0.022 (-0.363–0.408) | 0.016 (-0.351–0.378) | 0.026 (-0.276–0.313) | 0.039 (-0.228–0.308) |
|  | p | | - | | 0.364 (0.133–0.665) | 0.148 (0.013–0.497) | **0.047** (0.001–0.366) | **0.009** (0.000–0.244) | **0.000*** (0.000–0.115) | **0.000*** (0.000–0.048) |
| AMP vs rSO_2__R | r | | - | | 0.114 (-0.441–0.604) | 0.054 (-0.365–0.449) | 0.020 (-0.373–0.409) | 0.014 (-0.357–0.372) | 0.030 (-0.274–0.329) | 0.052 (-0.216–0.320) |
|  | p | | - | | 0.361 (0.132–0.661) | 0.145 (0.012–0.496) | **0.044** (0.000–0.347) | **0.009** (0.000–0.238) | **0.000*** (0.000–0.089) | **0.000*** (0.000–0.038) |
|  |  | | **10-Minute Temporal Resolution** | | | | | | | |
| ICP vs rSO_2__L | r | | - | | - | - | 0.106 (-0.791–0.857) | 0.110 (-0.490–0.637) | 0.118 (-0.266–0.481) | 0.108 (-0.210–0.412) |
|  | p | | - | | - | - | 0.376 (0.171–0.655) | 0.248 (0.062–0.572) | 0.145 (0.011–0.500) | 0.081 (0.002–0.424) |
| ICP vs rSO_2__R | r | | - | | - | - | 0.102 (-0.793–0.865) | 0.107 (-0.501–0.638) | 0.139 (-0.282–0.512) | 0.123 (-0.213–0.448) |
|  | p | | - | | - | - | 0.372 (0.169–0.647) | 0.246 (0.058–0.576) | 0.123 (0.008–0.458) | 0.062 (0.002–0.397) |
| AMP vs rSO_2__L | r | | - | | - | - | 0.039 (-0.817–0.846) | 0.038 (-0.547–0.614) | 0.062 (-0.346–0.442) | 0.064 (-0.264–0.403) |
|  | p | | - | | - | - | 0.375 (0.170–0.652) | 0.246 (0.060–0.565) | 0.131 (0.011–0.469) | 0.070 (0.002–0.400) |
| AMP vs rSO_2__R | r | | - | | - | - | 0.009 (-0.822–0.837) | 0.025 (-0.564–0.598) | 0.064 (-0.348–0.451) | 0.076 (-0.261–0.400) |
|  | p | | - | | - | - | 0.378 (0.169–0.649) | 0.246 (0.062–0.574) | 0.127 (0.012–0.472) | 0.073 (0.003–0.366) |

**Appendix F.**

*AMP* pulse amplitude of intracranial pressure, *ICP* intracranial pressure, *MAE* mean absolute error, *MSE* mean squared error, *RMSE* root mean squared error, *rSO_2__L* left sided regional oxygen saturation, rSO_2_*_R* right sided regional oxygen saturation.

**Table F.** Population summary of cross correlation function analysis across different window sizes and temporal resolutions.

|  |  | **Median (IQR)** | | | | | | | | | | | | | |
| --- | --- | --- | --- | --- | --- | --- | --- | --- | --- | --- | --- | --- | --- | --- | --- |
|  |  | | **1-Second Temporal Resolution** | | | | | | | | | | | | |
| **Physiologic Relationship** | **Value** | **1min** | | **5min** | | **15min** | | **30min** | | **1hr** | | **3hr** | | **6hr** | |
| ICP vs rSO_2__L | r (max CCF) | | 0.157 (-0.270–0.279) | | 0.102 (-0.146–0.162) | | 0.078 (-0.094–0.117) | | 0.065 (-0.069–0.099) | | 0.054 (-0.051–0.087) | | 0.042 (-0.033–0.070) | | 0.037 (-0.024–0.062) |
|  | Lag (s) | | -1.000 (-11.000–11.000) | | -5.000 (-28.000–30.000) | | -6.000 (-38.000–40.000) | | -6.000 (-29.000–25.000) | | -6.000 (-19.000–8.000) | | -6.000 (-11.000–-1.000) | | -6.000 (-9.000–-4.000) |
| ICP vs rSO_2__R | r (max CCF) | | 0.161 (-0.270–0.280) | | 0.103 (-0.145–0.162) | | 0.079 (-0.093–0.118) | | 0.066 (-0.069–0.100) | | 0.056 (-0.050–0.088) | | 0.045 (-0.030–0.073) | | 0.038 (-0.021–0.064) |
|  | Lag (s) | | -1.000 (-11.000–11.000) | | -6.000 (-27.000–30.000) | | -6.000 (-36.000–36.000) | | -6.000 (-24.000–23.000) | | -6.000 (-15.000–7.000) | | -6.000 (-9.000–-1.000) | | -6.000 (-9.000–-3.000) |
| AMP vs rSO_2__L | r (max CCF) | | 0.273 (-0.342–0.375) | | 0.175 (-0.196–0.239) | | 0.126 (-0.127–0.186) | | 0.101 (-0.095–0.163) | | 0.081 (-0.072–0.143) | | 0.059 (-0.046–0.116) | | 0.049 (-0.035–0.104) |
|  | Lag (s) | | -1.000 (-10.000–9.000) | | -2.000 (-23.000–22.000) | | -2.000 (-24.000–23.000) | | -2.000 (-19.000–15.000) | | -2.000 (-13.000–8.000) | | -2.000 (-9.000–4.000) | | -2.000 (-8.000–1.000) |
| AMP vs rSO_2__R | r (max CCF) | | 0.275 (-0.341–0.376) | | 0.177 (-0.195–0.242) | | 0.127 (-0.127–0.190) | | 0.103 (-0.095–0.168) | | 0.083 (-0.070–0.149) | | 0.063 (-0.043–0.124) | | 0.055 (-0.031–0.111) |
|  | Lag (s) | | -1.000 (-9.000–9.000) | | -2.000 (-22.000–23.000) | | -2.000 (-22.000–23.000) | | -2.000 (-15.000–13.000) | | -2.000 (-11.000–7.000) | | -2.000 (-7.000–3.000) | | -2.000 (-7.000–0.000) |
|  |  | | **10-Second Temporal Resolution** | | | | | | | | | | | | |
| ICP vs rSO_2__L | r (max CCF) | | - | | 0.354 (-0.419–0.487) | | 0.269 (-0.287–0.394) | | 0.226 (-0.222–0.365) | | 0.197 (-0.171–0.341) | | 0.174 (-0.105–0.308) | | 0.159 (-0.084–0.289) |
|  | Lag (s) | | - | | -10.000 (-20.000–20.000) | | -10.000 (-20.000–10.000) | | -10.000 (-20.000–0.000) | | -10.000 (-20.000–0.000) | | -10.000 (-20.000–0.000) | | -10.000 (-10.000–-10.000) |
| ICP vs rSO_2__R | r (max CCF) | | - | | 0.359 (-0.419–0.492) | | 0.272 (-0.287–0.407) | | 0.232 (-0.220–0.381) | | 0.204 (-0.170–0.356) | | 0.186 (-0.113–0.331) | | 0.160 (-0.085–0.316) |
|  | Lag (s) | | - | | -10.000 (-20.000–20.000) | | -10.000 (-20.000–10.000) | | -10.000 (-20.000–0.000) | | -10.000 (-20.000–0.000) | | -10.000 (-10.000–0.000) | | -10.000 (-10.000–0.000) |
| AMP vs rSO_2__L | r (max CCF) | | - | | 0.335 (-0.423–0.468) | | 0.245 (-0.291–0.357) | | 0.199 (-0.222–0.317) | | 0.160 (-0.173–0.290) | | 0.119 (-0.119–0.249) | | 0.095 (-0.094–0.224) |
|  | Lag (s) | | - | | -10.000 (-30.000–30.000) | | -10.000 (-30.000–30.000) | | -10.000 (-20.000–10.000) | | -10.000 (-20.000–0.000) | | -10.000 (-20.000–0.000) | | -10.000 (-20.000–0.000) |
| AMP vs rSO_2__R | r (max CCF) | | - | | 0.341 (-0.421–0.473) | | 0.250 (-0.288–0.367) | | 0.204 (-0.221–0.330) | | 0.163 (-0.172–0.306) | | 0.122 (-0.115–0.269) | | 0.103 (-0.089–0.242) |
|  | Lag (s) | | - | | -10.000 (-30.000–30.000) | | -10.000 (-30.000–30.000) | | -10.000 (-20.000–10.000) | | -10.000 (-20.000–10.000) | | -10.000 (-20.000–0.000) | | -10.000 (-20.000–0.000) |
|  |  | | **1-Minute Temporal Resolution** | | | | | | | | | | | | |
| ICP vs rSO_2__L | r (max CCF) | | - | | - | | 0.335 (-0.513–0.559) | | 0.258 (-0.409–0.474) | | 0.218 (-0.342–0.420) | | 0.189 (-0.271–0.366) | | 0.162 (-0.230–0.323) |
|  | Lag (s) | | - | | - | | 0.000 (-60.000–60.000) | | 0.000 (-60.000–60.000) | | 0.000 (-60.000–60.000) | | 0.000 (-60.000–60.000) | | 0.000 (-60.000–0.000) |
| ICP vs rSO_2__R | r (max CCF) | | - | | - | | 0.356 (-0.511–0.569) | | 0.281 (-0.406–0.484) | | 0.246 (-0.333–0.437) | | 0.196 (-0.259–0.385) | | 0.171 (-0.216–0.335) |
|  | Lag (s) | | - | | - | | 0.000 (-60.000–60.000) | | 0.000 (-60.000–60.000) | | 0.000 (-60.000–60.000) | | 0.000 (-60.000–60.000) | | 0.000 (-60.000–60.000) |
| AMP vs rSO_2__L | r (max CCF) | | - | | - | | 0.270 (-0.517–0.536) | | 0.185 (-0.415–0.432) | | -0.143 (-0.345–0.361) | | -0.113 (-0.272–0.271) | | -0.109 (-0.233–0.223) |
|  | Lag (s) | | - | | - | | 0.000 (-60.000–60.000) | | 0.000 (-60.000–60.000) | | 0.000 (-60.000–60.000) | | 0.000 (-60.000–60.000) | | 0.000 (-60.000–60.000) |
| AMP vs rSO_2__R | r (max CCF) | | - | | - | | 0.296 (-0.516–0.541) | | 0.208 (-0.417–0.439) | | 0.149 (-0.343–0.366) | | -0.120 (-0.275–0.281) | | -0.079 (-0.234–0.235) |
|  | Lag (s) | | - | | - | | 0.000 (-60.000–60.000) | | 0.000 (-60.000–60.000) | | 0.000 (-60.000–60.000) | | 0.000 (-60.000–60.000) | | 0.000 (-60.000–60.000) |
|  |  | | **10-Minute Temporal Resolution** | | | | | | | | | | | | |
| ICP vs rSO_2__L | r (max CCF) | | - | | - | | - | | - | | - | | 0.277 (-0.390–0.526) | | 0.227 (-0.304–0.440) |
|  | Lag (s) | | - | | - | | - | | - | | - | | 0.000 (-600.000–600.000) | | 0.000 (-600.000–0.000) |
| ICP vs rSO_2__R | r (max CCF) | | - | | - | | - | | - | | - | | 0.279 (-0.397–0.526) | | 0.237 (-0.296–0.453) |
|  | Lag (s) | | - | | - | | - | | - | | - | | 0.000 (-600.000–0.000) | | 0.000 (-600.000–0.000) |
| AMP vs rSO_2__L | r (max CCF) | | - | | - | | - | | - | | - | | 0.175 (-0.457–0.468) | | 0.174 (-0.344–0.377) |
|  | Lag (s) | | - | | - | | - | | - | | - | | 0.000 (-600.000–600.000) | | 0.000 (-600.000–600.000) |
| AMP vs rSO_2__R | r (max CCF) | | - | | - | | - | | - | | - | | 0.159 (-0.460–0.478) | | 0.152 (-0.354–0.396) |
|  | Lag (s) | | - | | - | | - | | - | | - | | 0.000 (-600.000–600.000) | | 0.000 (-600.000–600.000) |
